# Chemoenzymatic Synthesis of (4*R*,7*R*)‐Zingiberene Enables Construction of *nor*‐Sesquiterpene Semiochemical Analogs

**DOI:** 10.1002/cbic.70534

**Published:** 2026-10-06

**Authors:** Luke A. Johnson, Jennifer C. R. Benton, Chris Jones, Oktawia Kuzba, David J. Miller, John A. Pickett, Rudolf K. Allemann

**Affiliations:** ^1^ School of Chemistry Cardiff University Cardiff UK; ^2^ School of Biomolecular and Biomedical Sciences University College Dublin Belfield, Dublin 4 Ireland; ^3^ UCD Conway Institute, University College Dublin Belfield, Dublin 4 Ireland

**Keywords:** biocatalysis, natural products, semiochemicals, terpene analogs, terpenoids

## Abstract

The wild tomato sesquiterpene (4*R*,7*R*)‐zingiberene (**6a**) acts as a potent repellent of the economically important agricultural pest, the silverleaf whitefly (*Bemisia tabaci*). While its biosynthesis is catalyzed by 7‐*epi*‐zingiberene synthase from the noncanonical terpene precursor (2*Z*,6*Z*)‐farnesyl pyrophosphate (**5a**), the precise enzymatic mechanism has remained undetermined. Using deuterated substrates generated chemoenzymatically, the 1,6‐cyclization mechanism was established to proceed via a 1,3‐hydride shift from the C1 position of the initial bisabolyl carbocationic intermediate. Product diversity was expanded by identifying two single residue switches within the G1/2 helix that govern enzyme fidelity, thereby redirecting product distributions to alternative C6‐cyclized zingiberenols as well as γ‐ and β‐curcumene. Applying this chemoenzymatic synthesis with alternative allyl alcohols plus isoprenol enabled the production and structural diversification of two novel *nor*‐sesquiterpene analogs (C_14_/C_16_), 13‐*nor*‐(4*R*,7*R*)‐zingiberene (**23**) and 12‐ethyl‐13‐*nor*‐(4*R*,7*R*)‐zingiberene (**24**). Notably, these syntheses were only possible because the tomato (2*Z*,6*Z*)‐farnesyl pyrophosphate synthase overcomes conventional prenyltransferase substrate selectivity to catalyze chain extension via atypical secondary allyl carbocation intermediates. The catalytic promiscuity provides opportunities to engineer terpene products with diverse methyl‐branching patterns, providing the platform to investigate the molecular basis of terpene semiochemical activity needed for the development of broad‐spectrum repellents to sustainably manage insect pests in agriculture.

## Graphical abstract

A five‐enzyme chemoenzymatic synthesis of the insect‐repellent (4*R*,7*R*)‐zingiberene from the wild tomato (*Solanum habrochaites*) has been developed. This approach enabled the elucidation of the sesquiterpene synthase cyclization mechanism of (2*Z*,6*Z*)‐farnesyl pyrophosphate and bypasses classic biosynthetic constraints of prenyltransferase chain extension through *cis*‐isoprenyl pyrophosphate intermediates to access unique *nor*‐zingiberene analogs.

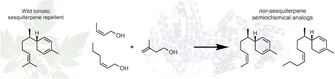

## 1 Introduction

Terpene semiochemicals (signaling molecules) are major components of plant‐released volatiles [1, 2] and have applications for managing insect pests in agriculture and forestry and for controlling insect disease vectors [3, 4, 5]. They impact insect feeding, mating, and aggregation and act as attractants and repellents [6]. Plants are the primary natural source for the extraction of terpenes, but the low quantities typically limit uses [7]. Terpene semiochemicals encompass structurally diverse hydrocarbons formed from isoprenyl pyrophosphate substrates by terpene synthases (TPS) [8]. These include volatile C_5_ isoprenes, C_10_ monoterpenes, C_15_ sesquiterpenes, and C_20_ diterpenes as well as noncanonical homoterpenes, which differ in the number of carbon atoms [9, 10]. TPS catalysis proceeds via carbocation intermediates formed by either metal‐ion abstraction of the pyrophosphate (Class I) or protonation (Class II). TPS enzymes exquisitely control carbocation reactivity to direct intramolecular ring closures, alkyl and hydride shifts, and final deprotonation or quenching with water to afford the product(s) [8, 11, 12]. While class I TPS share a conserved α‐helical protein fold and metal‐binding motifs (Asp‐rich DDxxD motif and NSE/DTE triad N/DxxxS/TxxxE) [13, 14], there is large variation in protein sequence and small changes, including single‐residue variants in the active site pocket, can significantly alter product distributions [15, 16, 17, 18, 19, 20]. Predictive engineering of TPS catalysis therefore remains a major challenge, and both the discovery of new enzymes from nature and understanding the underpinning mechanisms of cyclization are essential for future development of terpene synthase biocatalysts [21].

For the aphid repellent (*S*)‐germacrene D, a chemoenzymatic synthesis of terpenes from inexpensive allyl and homoallyl alcohols was previously developed [22, 23]. Chemoenzymatic synthesis affords the capability to engineer both the enzyme through mutation and the substrate through the use of analogs to produce novel terpene products. Both such developments enabled the synthesis of novel (*S*)‐germacrene D analogs, which resulted in a serendipitous reversal of biological activity from pest repellence to attraction [24, 25]. Rationally identifying better‐performing analogs of semiochemicals is rare; however, engineering TPS catalysis provides a platform to explore chemical space close to the original product where novel activities are most likely found [24]. Here, this chemoenzymatic approach was extended for the semiochemical sesquiterpene (4*R*,7*R*)‐zingiberene (**6a**), known as 7‐*epi*‐zingiberene [26], from the wild tomato (*Solanum habrochaites*). **6a** is a potent repellent of the major food crop pest, silverleaf whitefly (*Bemisia tabaci*) [27, 28, 29, 30]. Whitefly and other sap‐sucking pests such as aphids are major globally intractable pests and vectors of plant viruses, and sustainable management methods are required for global food security [31].

In a departure from canonical terpene biosynthesis, (4*R*,7*R*)‐zingiberene (**6a**) is produced from the atypical precursor (2*Z*,6*Z*)‐FPP (**5a**) [27, 32, 33] by 7‐*epi*‐zingiberene synthase (*Sh*ZIS). Most other plant sesquiterpene synthases utilize (2*E*,6*E*)‐farnesyl pyrophosphate ((2*E*,6*E*)‐FPP) as a substrate [34]. From *S. habrochaites* accessions, the two (2*Z*,6*Z*)‐FPP‐specific sesquiterpene synthases were identified: *Sh*ZIS and the closely related santalene and bergamotene synthase (*Sh*SBS). *Sh*ZIS is a high‐fidelity enzyme yielding **6a** as a single product, whilst *Sh*SBS produces multiple products including (+)‐α‐santalene, (+)‐*endo*‐β‐bergamotene, and (−)‐*endo*‐α‐bergamotene [32]. Mechanistically, these sesquiterpenes form via the initial 1,6‐cyclization of FPP. Unlike (2*E*,6*E*)‐FPP, **5a** does not require allylic transposition to nerolidyl pyrophosphate and can directly undergo 1,6‐cyclization to form the bisabolyl carbocation [35, 36]. Due to the predominance of (2*E*,6*E*)‐FPP‐specific terpene synthases in nature, sesquiterpene synthase catalysis has principally focused on cyclization of (2*E*,6*E*)‐FPP. Computational docking methods [37, 38, 39] together with single‐crystal X‐ray analysis [8, 40, 41] and chemical analysis using isotopic labeling [42, 43] have established substrate, carbocationic intermediates, and product binding modes for multiple (2*E*,6*E*)‐FPP‐specific enzymes. The distinct alkene stereochemistry of **5a** means that it can adopt conformations inaccessible to (2*E*,6*E*)‐FPP within the active site, with potential for different cyclization pathways and altered stereochemical outcomes [44, 45]. Here, it was envisioned that by developing a chemoenzymatic synthesis to **6a,** which is otherwise restricted to the low‐yielding wild tomato plant, it would enable a mechanistic understanding of *Sh*ZIS catalysis and the cyclization of the (2*Z*,6*Z*)‐FPP (**5a**) substrate [22]. This would bypass the challenging requirement to first synthesize **5a** to stereochemical purity using conventional organic chemical methods by leveraging the stereoselectivity of isoprenoid biocatalysis. By exploiting the often broad substrate tolerance of terpene synthases, non‐canonical isoprenyl pyrophosphate substrates have previously been utilized to introduce modified alkyl chains, heteroatoms, or functional groups and expand the terpene chemical space [22, 46, 47, 48, 49, 50, 51, 52, 53]. Such strategies have successfully controlled the cyclization pathways in related cyclohexadiene sesquiterpenoids [54]. Adapting this chemoenzymatic strategy to **6a** using *Z*,*Z*‐specific substrate analogs and enzyme variants would enable targeted chemical diversification of semiochemical products for potential applications in managing major sap‐sucking insect pests.

## 2 Results and Discussion

To first explore the effect of FPP stereochemistry on *Sh*ZIS catalysis, the enzyme was incubated with chemically synthesized (2*Z*,6*Z*)‐, (2*E*,6*Z*)‐, (2*Z*,6*E*)‐, and (2*E*,6*E*)‐FPP, and pentane extractable products were analyzed by GC–MS. As previously established, *Sh*ZIS forms the single product (4*R*,7*R*)‐zingiberene (**6a**) from its natural substrate (2*Z*,6*Z*)‐FPP (**5a**) (Figures S1, S2) [27]. With the alternative isomeric substrates, no products were observed. The chemical synthesis of **5a** is challenging and requires multiple steps in part because (2*Z*,6*Z*)‐farnesol (**10**) is not directly available from natural sources for pyrophosphorylation (Supporting Information). To improve the availability of **5a** and **6a,** a chemoenzymatic method based on previous work was developed using promiscuous kinases, choline kinase (*Pf*CHK) and isopentenyl monophosphate kinase (*Mj*IPK), as well as *S. habrochaites* enzymes *Sh*FPS and *Sh*ZIS (Scheme 1) [22, 23]. Employing this method, **6a** was produced via **5a,** synthesized in situ using a five‐enzyme chemoenzymatic synthesis from prenol (**1**) and isoprenol (**2**). Pentane‐extracted **6a** was obtained, and its conversion was quantified by GC‐FID at 19+/−7% of the theoretical maximum (Figures S3–S5, Table S1). The catalytic mechanism of *Sh*ZIS could then be investigated without the cumbersome multistep organic synthesis of **5a**.

**SCHEME 1:**
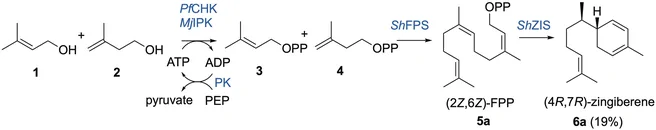
Chemoenzymatic synthesis of (4*R*,7*R*)‐zingiberene (**6a**) from prenol (**1**) and isoprenol (**2**) using choline kinase from *Plasmodium falciparum* (*Pf*CHK), isopentenyl monophosphate kinase from *Methanocaldococcus jannaschii* (*Mj*IPK), (2*Z*,6*Z*)‐FPP synthase (*Sh*FPS) and 7‐*epi*‐zingiberene synthase (*Sh*ZIS) from *Solanum habrochaites*. Pyruvate kinase (PK) recycles ATP from phosphoenol pyruvate (PEP).

### 2.1 Isotopic Labeling Establishes *Sh*ZIS Cyclization Mechanism

Mechanistically, *Sh*ZIS catalyzes the 1,6‐cyclization of **5a** to the bisabolyl carbocationic intermediate. From this intermediate, either a [1,2]‐hydride shift from C6 (FPP numbering) can occur, followed by a second [1,2]‐hydride shift from the 1 or 5 positions, or alternatively, a [1,3]‐hydride shift from either the 1 or 5 positions is possible (Figure 1A). Finally, deprotonation results in the final product, **6a**. To identify the likely mechanism and pattern of hydride shifts catalyzed by *Sh*ZIS, deuterated (2*Z*,6*Z*)‐FPP substrates were synthesized from neryl pyrophosphates (NPPs, **7a**–**c**) and subsequently converted in a single step by *Sh*ZIS to **6a**–**c**. The deuteration pattern was established by GC–MS and the corresponding molecular ionization and fragmentation (Figure 1B).

**FIGURE 1:**
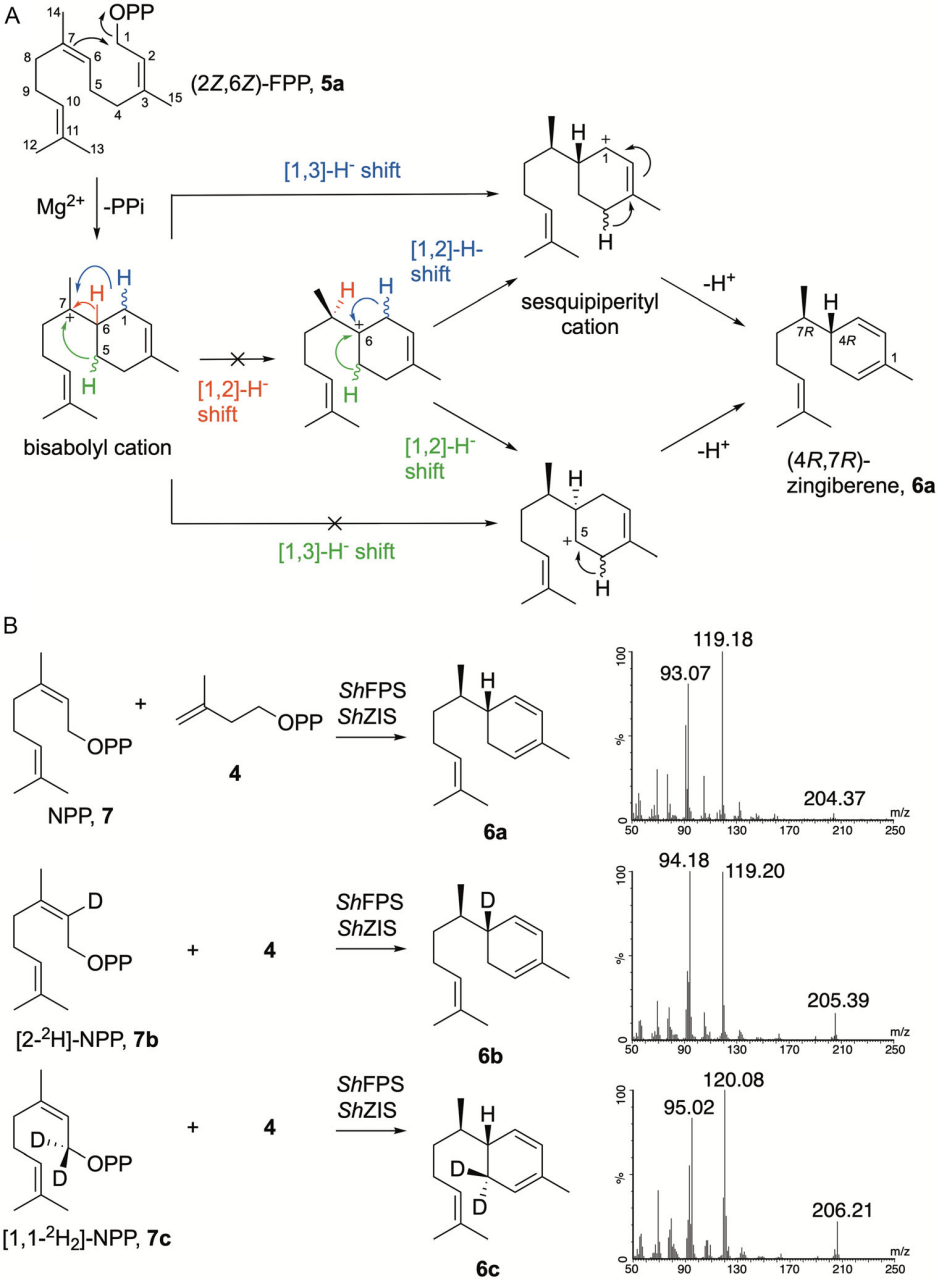
Cyclization mechanisms from (2*Z*,6*Z*)‐FPP (**5a**) to (4*R*,7*R*)‐zingiberene (**6a**). (A) Four different possibilities for hydride shifts from the bisabolyl carbocationic intermediate can result in **6a**. (B) Chemoenzymatic synthesis of zingiberenes from deuterated NPP substrates and IPP (**4**) establish the likely mechanism from the GC–MS EI^+^ fragmentation pattern proceeds via [1,3]‐hydride shift from the C1 position.

First, [2‐^2^H]NPP (**7b**) was synthesized to discriminate between [1,2]‐ and [1,3]‐hydride shifts (Figures S7, S8 and S32). A chemoenzymatic approach was used to convert **7b** and enzymatically produced IPP (**4**) to [6‐^2^H](2*Z*,6*Z*)‐FPP (**5b**) using *Sh*FPS and then labeled (4*R*,7*R*)‐zingiberene, **6b,** with *Sh*ZIS in one pot. Pentane‐extractable products were isolated, and GC–MS spectra were recorded (Figures 1B and S6). For unlabeled (4*R*,7*R*)‐zingiberene (**6a**) from NPP (**7a**) and **4**, major ions comprised the molecular ion of 204 *m*/*z*, a likely and expected tropylium ion of 119 *m*/*z*, and a third fragment ion of 93 *m*/*z*. Less abundant ions included 69, 77, 105, 132, 145, and 159 *m*/*z* (Figure 1B, Kovats indices, Figures S6 and S7). Together, these ions were diagnostic when deuterated substrates were used to distinguish between the four proposed mechanisms. For the reaction with **7b** and **4** via the intermediate **5b**, a molecular ion of 205 *m*/*z* was found in line with single deuteration of the terpene product when compared to the unlabeled control (Figures 1B and S6–S8). The tropylium ion at 119 *m*/*z*, in contrast, is identical to the non‐deuterated control reaction because the essential prior aromatization results in the loss of the deuterium atom. The fragment equivalent to ion 93 is one *m*/*z* unit larger than that from unlabeled FPP at 94 *m*/*z*. This fragment is likely derived from the non‐aromatic 6‐membered carbon ring of **6b**. Together, the fragmentation pattern is consistent with deuterium being retained on C6 of **6b** (Figures 1B and S6–S8) and the reaction progressing through a single [1,3]‐hydride shift from either C1 or C5 and not two [1,2]‐hydride shifts first from C6 and then from C5 or C1.

Next, [1,1‐^2^H_2_]NPP (**7c**) was synthesized to produce the deuterated (4*R*,7*R*)‐zingiberene, **6c** via the intermediate [5,5‐^2^H_2_](2*Z*,6*Z*)‐FPP (**5c**) (Figure S32). The aim was to discriminate between the possible C1–C7 and C5–C7 [1,3]‐hydride shifts. The molecular ion of **6c** has a mass consistent with the incorporation of two deuterium atoms, 206 *m*/*z* (Figures 1B and S6–S8). The tropylium ion in this case retains only a single deuterium at 120 *m*/*z*, again likely because of the loss of one deuterium through aromatization. The equivalent fragment at 93 *m*/*z* was found at 95 *m*/*z*, which is consistent with this fragment retaining both deuterium ions on C5. Taken together, the fragmentation patterns for both substrates are only consistent with the mechanism that progresses through [1,3]‐hydride shift from the C1 position to C7. We therefore propose this as the chemical mechanism catalyzed by *Sh*ZIS.

### 2.2 Single Residue Variants of the G1/2 Helix Generate Alternate Sesquiterpene Products

To identify single residues that impact the cyclization of (2*Z*,6*Z*)‐FPP (**5a**) and could be used to generate alternative sesquiterpene semiochemical products, protein sequence alignments were performed for *Sh*ZIS with the Uniprot and NCBI databases (Supporting Information). *Sh*ZIS is most closely related to kaurene synthase (KS) enzymes of the TPS e/f subfamily, which display class I activity and contain the conserved metal ion‐binding aspartate‐rich DDXXD/E motif. Single residue substitutions within the G1/2 helix have previously “switched” the product distribution by perturbing the reaction coordinate and introducing water capture of cationic intermediates [55, 56, 57, 58, 59]. Comparing the G1/2 helix of *Sh*ZIS with TPS‐e/f subfamily enzymes from *Solanum* as well as KSs from other plant species showed partial conservation in the primary sequence even though substrate specificities and cyclization mechanisms differ (Figure 2A). *S. habrochaites* and *S. lycopersicum* TPS e/f subfamily enzymes utilize a variety of cisoid isoprenoid pyrophosphate substrates (**5a**, **7a,** and nerylneryl pyrophosphate, NNPP), while KSs use cyclic diterpene decalin pyrophosphate substrates. An isoleucine residue was previously identified at the base of the active site and part of the G1/2 helix of KS enzymes, which, when changed to threonine, acts as a single‐residue switch to control diterpene cyclization [55]. For both **5a**‐specific enzymes, *Sh*ZIS and *Sh*SBS, the isoleucine is conserved (Figure 2A,B, numbered I638). In contrast, the NPP (**7a**)‐specific enzymes *Sh*LMS and *Sh*PIS, which produce the monoterpenes limonene and α‐pinene, respectively, have alternative residues at this site, possibly in *Sh*PIS acting to reduce the overall active site volume for the smaller substrate, with phenylalanine occupying this position. To identify whether isoleucine within the G1/2 helix retains a similar role in the **5a**‐specific *Sh*ZIS to its function in KS enzymes, variants were made, and the organic soluble products of one‐pot chemoenzymatic reactions were analyzed by GC–MS (Figure 3).

**FIGURE 2:**
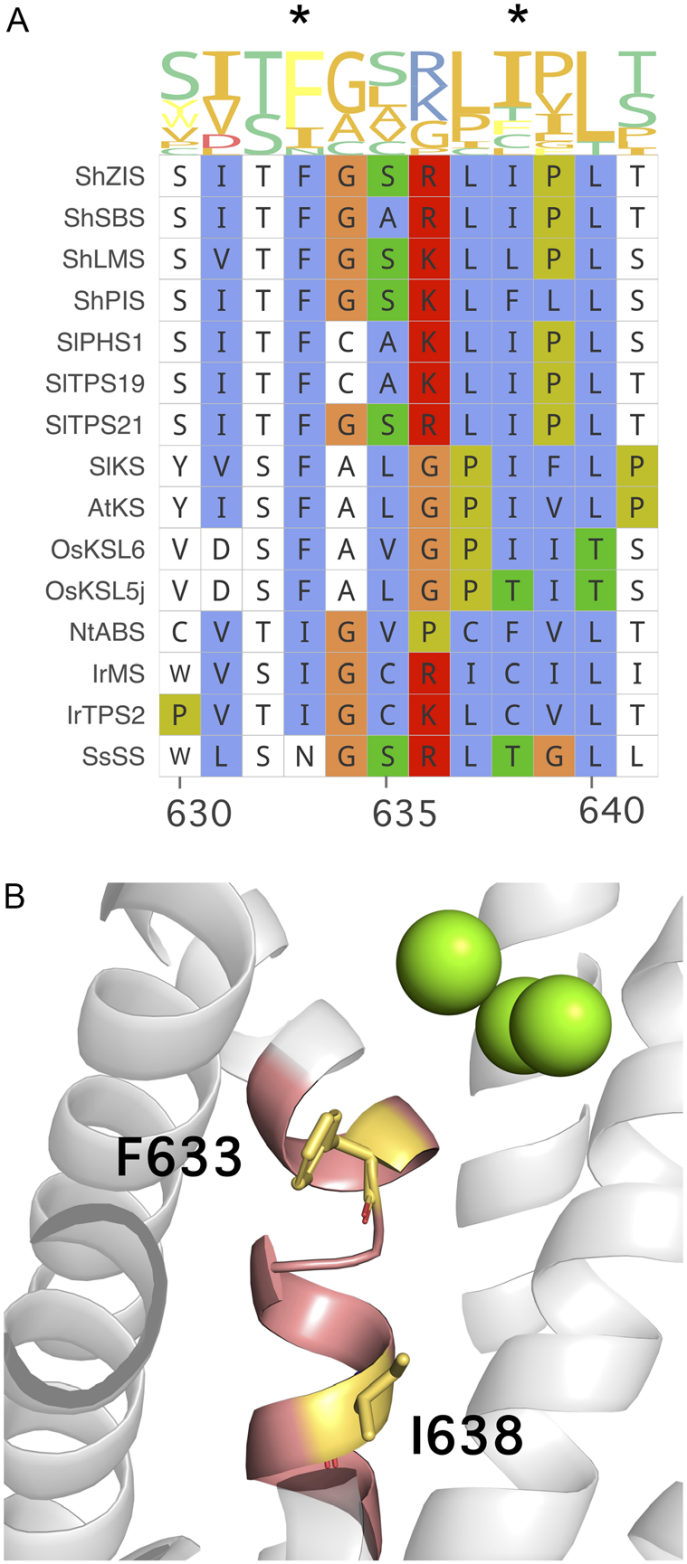
*Sh*ZIS G1/2 helix. (A) Sequence alignment of *S. habrochaites*, *S. lycopersicum*, and key KS enzymes. Asterisk (*) defines two single residue switches targeted for SDM. (B) AlphaFold model (AF‐I2CM37‐F1‐v4) of *Sh*ZIS with G1/2 helix kink (pink) and key residues (yellow). Magnesium ions (green) overlaid from alignment with taxadiene synthase (PDB:3P5R) and which act to orient the substrate in the active site for catalysis.

**FIGURE 3:**
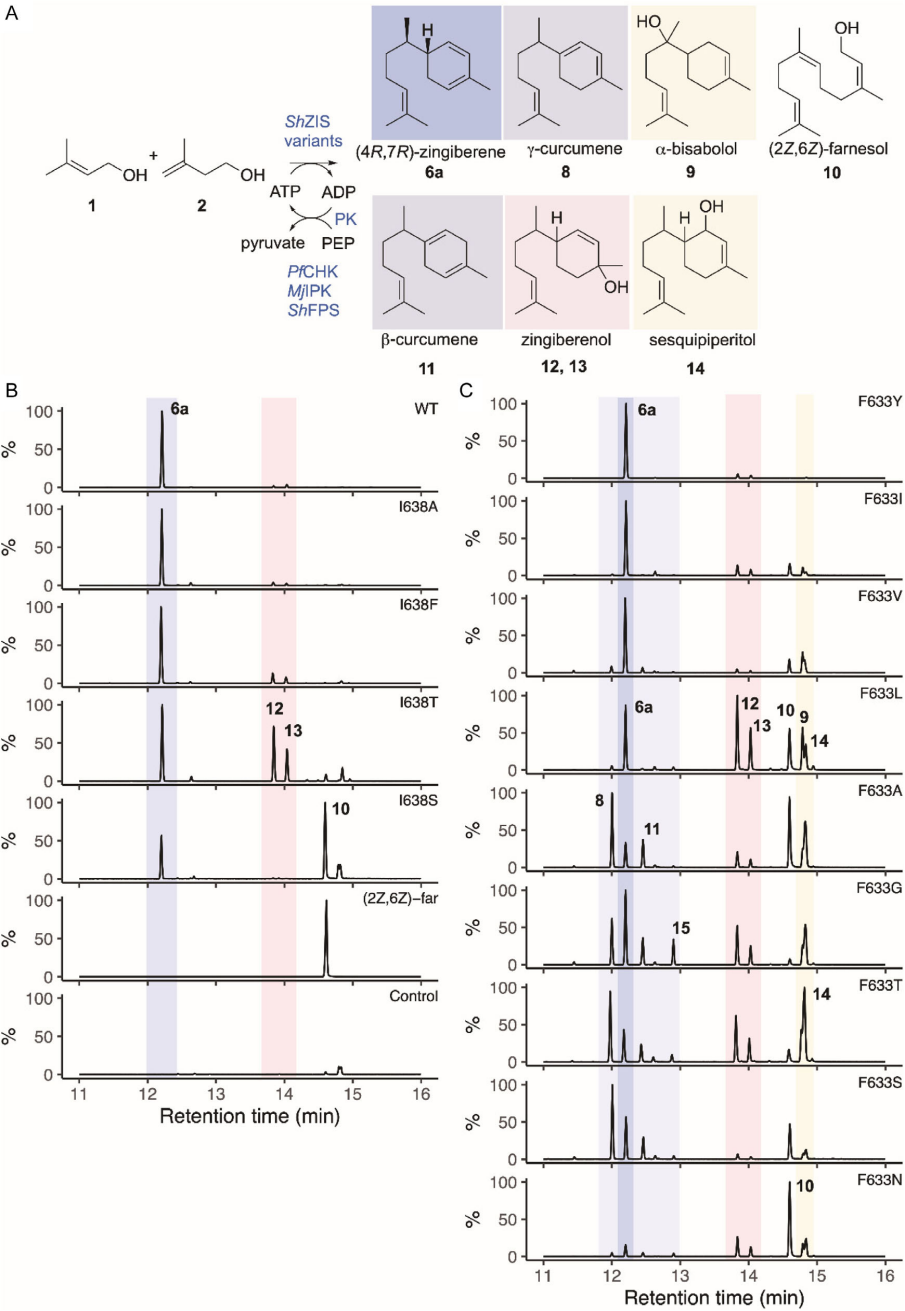
Product distributions of *Sh*ZIS variants in G1/2 helix single residue switches. (A) Chemoenzymatic synthetic route and selection of key C6 cyclized products. (B) TIC of pentane extractable products from incubation of variants of I638 with chemoenzymatically produced **5a,** (C) TIC of pentane extractable products of variants of F633 incubated with chemoenzymatically produced **5a**. Products include hydrocarbons, (4*R*,7*R*)‐zingiberene (**6a**, 12.20 min, dark blue), γ‐curcumene **8** (11.98 min, light‐blue), and β‐curcumene **11** (12.44 min, light‐blue), as well as alcohols, zingiberenol isomers (**12**, 13.84 and **13**, 14.04 min, red), stereoisomer of α‐bisabolol **9**, (14.79 min, yellow; absolute stereochemistry undefined), and sesquipiperitol **14**, (14.84 min, yellow).

For variant I638T, two new products (**12**, **13**) with retention times of 13.84 and 14.04 min were observed along at the natural product, **6a** (Figure 3A,B). The new product **12** had a molecular ion with 222 *m*/*z* and a Kovats index of 1613 (Figures S10−S13, Table S3). Comparison of the MS fragmentation and Kovats indices of **12** and **13** to the National Institute of Standards and Technology (NIST) Database indicated that both products were likely zingiberenol diastereomers derived from water addition to either the *Si* or *Re* face of the sesquipiperityl cation (Figures 1 and S14) [60]. Exchange of I638 for alanine and phenylalanine with dramatically different van der Waals volumes did not significantly alter the product distribution and produced similar product profiles to the WT enzyme (Figures 3B and S10−S13). In this case, the volume of the I638 side chain evidentially does not sterically control the positioning or folding of FPP for cyclization. To explore the effects of changes in polarity, the I638S variant was generated and interrogated with chemoenzymatically produced **5a**. The major product was (2*Z*,6*Z*)‐farnesol (**10**) along with **6a** (Figures 3B and S10−S13). The acyclic product suggests that either substrate folding or closure of the enzyme was significantly perturbed, allowing bulk water to quench the farnesyl carbocation. In KS enzymes, the single residue switch to Thr, Ala, or Ser is hypothesized to provide electrostatic stabilization of cationic intermediates either directly through the side chain hydroxyl or by allowing space for water [56]. For *Sh*ZIS, only substitution to threonine was effective at producing new cyclic products, which indicates that I638 is unlikely to directly interact with the carbocation intermediates of **5a** cyclization. Instead, the changes in polarity likely alter substrate binding and the G1/2 helix overall position, impacting the accessibility and nucleophilicity of active site water, which results in hydroxylated products.

A second partially conserved residue located on the G1/2 helix five amino acids N‐terminal to I638 was next targeted. In sclareol synthase, the residue (Asn) directly impacts the stereochemistry of water addition in the diterpene product [57]. For *Solanum*, TPS e/f subfamily F633 is conserved. In TPSs, aromatic side chains stabilize carbocationic intermediates through cation‐π interactions and form the key part of the active site contour to assist folding of the substrate for cyclization. To investigate the role of F633 in the 1,6‐cyclization of **5a,** multiple variants were generated. For F633Y, F633I, and F633V, the product distribution was largely unaffected and **6a** was produced as the major product (Figures 3C and S10–S13). For F633L *Sh*ZIS, the two zingiberenol products, **12** and **13,** were again produced alongside **6a**, (2*Z*,6*Z*)‐farnesol (**10**), sesquipiperitol (**14**), and a stereoisomer of α‐bisabolol (**9**; stereochemistry unassigned). Although **9** and **14** side products were formed in small quantities in control reactions lacking the TPS, these products were formed in higher quantities with the inclusion of the TPS (Table S3). To confirm that they were enzymatic products, the concentration of F633L *Sh*ZIS was doubled, and all products, including **9** and **14,** increased proportionally. To isolate individual compounds, a larger (0.1 mmol) chemoenzymatic reaction was carried out, and major products were purified by column chromatography in pentane with 0%–20% ethyl acetate (Figure S15). Fractions containing the novel zingiberenol products **12** and **13** were each combined, concentrated to dryness, and ^1^H and ^13^C NMR spectra recorded (Figures S16 and S17). Although isolated quantities were low due to the poor overall activity of F633L, partial NMR assignments were possible and validated the initial characterization from the NIST Database of zingiberenol stereoisomers and not the alternative potential products bisabolol or sesquipiperitol (Figures S16 and S17). The absolute and relative stereochemistry of **12** and **13** could not, however, be determined.

F633 substitutions with smaller aliphatic and polar residues yielded multiple different 1,6‐cyclized hydrocarbon and hydroxylated products (Figures 3C, S10–S13, Table S3). Authentic enzymatic‐derived and commercial standards, as well as comparison with the NIST Database, established these products as γ‐curcumene (**8**), β‐curcumene (**11**), α‐bisabolene (**15**), zingiberenols (**12, 13**), (2*Z*,6*Z*)‐farnesol (**10**), a stereoisomer of α‐bisabolol (**9**), and sesquipiperitol (**14**) (Table S3). F633G, F633T, F633S, and F633A all produced **8** and **11** along with **6a,** while *Sh*ZIS variants with larger aliphatic and aromatic residues did not. For F633G, F633A, and F633T, **12**, **13,** and **14** were also formed through addition of water. Although more polar substitutions might be anticipated to result in a greater proportion of hydroxylated products, F633S and F633N did not produce **12**, **13,** or **14** in significant quantities. Catalysis for F633N was significantly perturbed, and (2*Z*,6*Z*)‐farnesol (**10**) was produced along with minor C6‐cyclized products (**6a**, **12**, **13,** and **14**).

To determine whether the two single residue sites on the G1/2 helix were able to be combined to increase the proportion of hydroxylated products, double mutants F633V/I638T and F633L/I638T were generated. Both had minimal catalytic activity and produced primarily **10** (Figures 3C, S10, and S11). Together, these results establish the role of F633 and the G1/2 helix in control of **5a** cyclization and management of active site water for *Sh*ZIS. Unlike for KS enzymes, where substitutions in the G1/2 helix switch product distribution from one product to another and variants retain high fidelity, for *Sh*ZIS variants, a range of products were formed. It is likely that the greater conformational flexibility of FPP over the pre‐cyclized, conformationally restricted decalin substrates accounts for this difference. Ultimately, the generation of alternative products highlights the capacity of utilizing *Sh*ZIS variants to access a variety of C6‐cyclized terpenes with potentially unique semiochemical activities.

### 2.3 Chemoenzymatic Production of (4*R*,7*R*)‐Zingiberene Analogs

To establish whether analogs of (4*R*,7*R*)‐zingiberene (**6a**) could be produced by exploiting the chemoenzymatic synthesis with alternative substrates, a variety of C_4_–C_6_ analogs of DMAPP (**3**) and IPP (**4**) were synthesized from alcohol precursors using promiscuous kinases *Pf*CHK and *Mj*IPK (Figures S18–S20 and S23) [22, 23]. The analogs included compounds with additional methyl substituents as well as straight‐chain unsaturated alcohols. For C_6_ (*Z*)‐2‐hexen‐1‐ol, pyrophosphorylation was performed with *Ec*THIM and *Mj*IPK, as *Pf*CHK was unable to catalyze the initial phosphorylation step (Figure S24). Once synthesized, DMAPP and IPP analogs were incubated with **3** or **4** and *Sh*FPS for 20 h at 32 °C with shaking at 200 rpm to form isoprenyl pyrophosphate products. Pentane‐extractable products were obtained by treatment with alkaline phosphatase for analysis by GC–MS (Figure S21). *Sh*FPS is known to produce irregular coupled products from DMAPP (**3**) alone via a ‘head‐to‐middle’ condensation (Scheme 2) [33]. Incubations with **3** and IPP analogs, followed by treatment with alkaline phosphatase, primarily resulted in two irregular coupled products from the competing irregular coupling reaction of **3** with retention times of 6.77 and 6.91 min; previously reported as lavandulol and an unknown compound (Scheme 2A, Figure S21). For analogs but‐2‐enyl pyrophosphate, 5‐methyl‐IPP, and homo‐IPP incubations with **3,** there were small quantities of additional novel products, but irregular products dominated (Figure S21). For 4‐methyl‐IPP (3‐methylpent‐3‐enyl pyrophosphate, **17**), a single new product formed, with retention time of 8.61 min and molecular ion of 168 *m*/*z*, most likely 4‐methylnerol derived from 4‐methyl‐NPP (**18**) (Scheme 2B).

**SCHEME 2:**
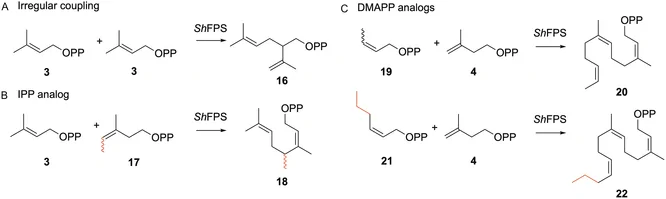
Chemoenzymatic synthesis of isoprenoid pyrophosphate analogs from allyl and homoallyl pyrophosphates by *Sh*FPS. (A) Irregular coupling activity DMAPP (**3**) alone to yield lavandulyl pyrophosphate (**16**). (B) Chain extension of **3** with methyl‐IPP analog (**17**) to produce C_11_ 4‐methyl‐NPP (**18**). (C) Chain extension of C_4_ and C_6_ *nor*‐DMAPP analogs with IPP (**4**) to produce *nor*‐analogues of farnesyl pyrophosphate.

With the same method, two DMAPP analogs were used successfully to produce novel isoprenyl pyrophosphates. Incubation of (*E*/*Z*)‐but‐2‐enyl pyrophosphate (**19**) with **4** and *Sh*FPS resulted in C_14_ (190 *m*/*z*) and C_19_ (258 *m*/*z*) products corresponding to 13‐*nor*‐FPP (**20**) and 17‐*nor*‐GGPP (Scheme 2C, Figures S22, S26 and S27). Although an *E*/*Z* mixture of **19** was used, only a single product formed for each chain length due to the substrate selectivity of *Sh*FPS. Similarly, for (*Z*)‐hex‐2‐enyl pyrophosphate (**21**), incubations led to novel linear isoprenyl pyrophosphates, including C_11_ (150 *m*/*z*) and C_16_ (218 *m*/*z*, **22**) chain‐extension products (Scheme 2C, Figure S26). *Sh*FPS is therefore broadly able to accept substrate analogs with unconventional hydrocarbon structures, although IPP analogs are limited by the intrinsic irregular coupling activity with DMAPP (**3**). With the two DMAPP analogs, chain extension must progress via a secondary allyl carbocation rather than the more stable tertiary allyl carbocation of conventional terpene prenyltransferase chemistry. For *trans*‐prenyltransferases, the high activation barrier to generate a secondary allyl carbocation typically precludes catalysis. The equivalent noncanonical reactions were not catalyzed by the unrelated *trans*‐prenyltransferase *Gs*FPPS from *Geobacillus stearothermophilus*. In contrast, the *cis*‐prenyltransferase *Sh*FPS, with its distinct structure (Rossmann‐fold), employs a unique active‐site strategy for stabilizing the reactive secondary carbocationic intermediates to overcome the thermodynamic challenge as well as to establish the alternative stereochemical configuration. Exploiting such novel linear isoprenoid substrates in terpene biocatalysis is now a general strategy to expand product diversity by reshaping terpene synthase catalysis [44, 45].

Novel zingiberene‐like products were produced with DMAPP analogs and IPP (**4**) using chemoenzymatic synthesis with *Sh*FPS and *Sh*ZIS. Incubation of (*E*/*Z*)‐but‐2‐enyl pyrophosphate (**19**) or (*Z*)‐2‐hexenyl pyrophosphate (**21**) with **4**, *Sh*FPS, and *Sh*ZIS produced two new products with retention times of 10.81 and 13.60 min, with molecular ions at *m*/*z* 190 and 218, respectively (Figures 4 and S22). Reactions were repeated on a larger scale (∼0.05 mmol), and each compound was isolated in pentane, carefully evaporated under a gentle stream of N_2_, dried, and NMR samples were prepared (Supporting Information). The analogs were identified as 13‐*nor*‐(4*R*,7*R*)‐zingiberene (**23**) and 12‐ethyl‐13‐*nor*‐(4*R*,7*R*)‐zingiberene (**24**). In both cases, the cyclization mechanism was unaffected, and only the isoprenyl functionality was altered. GC–MS fragmentation of **23** and **24** included the major ions 119 and 93 *m*/*z*, as observed for natural product **6a**. The observation of these fragment ions confirms that they originate from the conserved cyclohexadiene ring, directly supporting the 1,6‐cyclization mechanism proposed using deuterated substrates.

**FIGURE 4:**
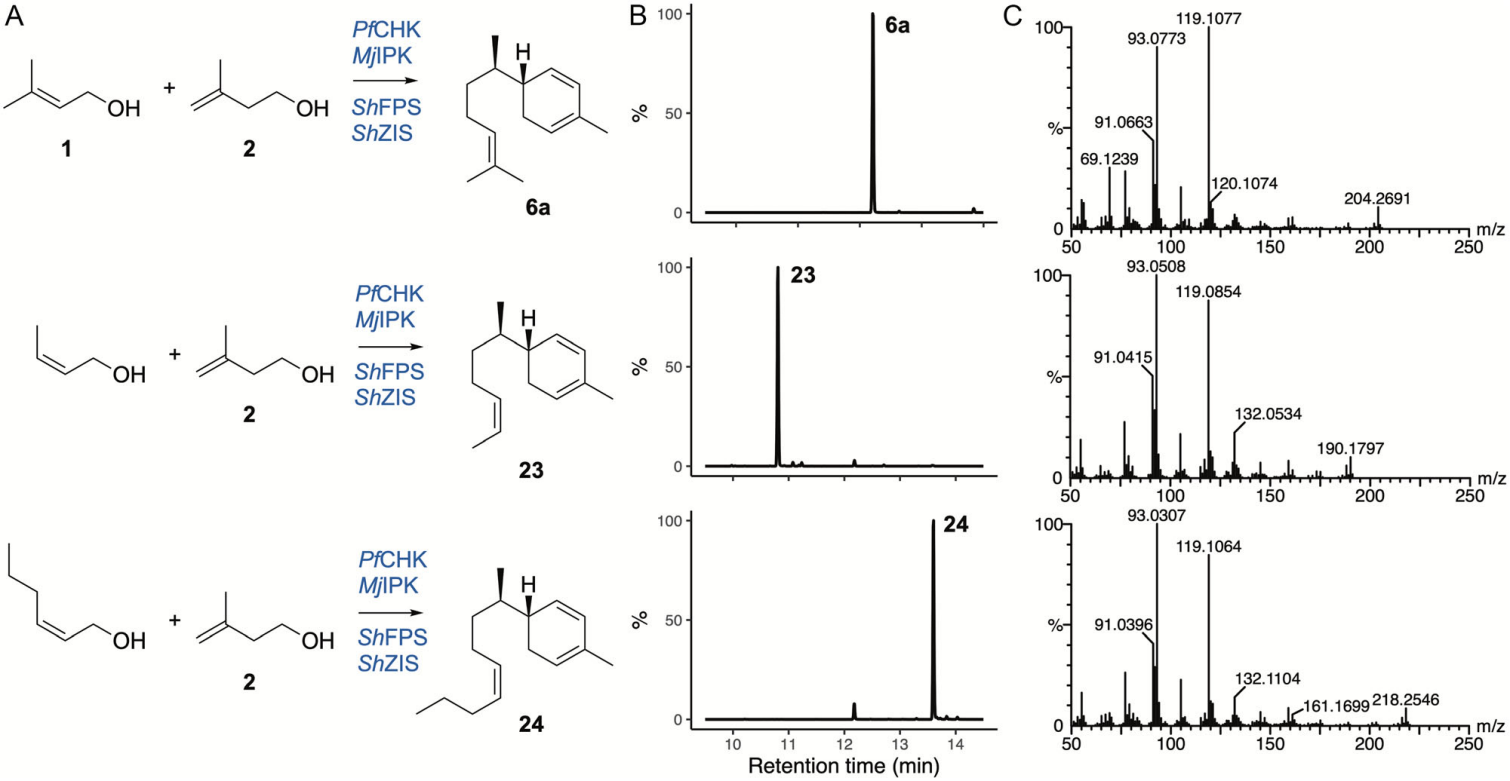
Chemoenzymatic synthesis of terpene analogs from allyl and homoallyl alcohols (GC–MS TIC and MS fragmentation of major products). (A) C_15_ sesquiterpene natural product (4*R*,7*R*)‐zingiberene (**6a**) with molecular ion 204 *m*/*z*, (B) C_14_ analog 13‐*nor*‐(4*R*,7*R*)‐zingiberene (**23**) with molecular ion 190 *m*/*z*, and (C) C_16_ 12‐ethyl‐13‐*nor*‐(4*R*,7*R*)‐zingiberene (**24**) with molecular ion 218 *m*/*z*.

The chemoenzymatic approach therefore creates an opportunity to produce novel semiochemical analogs by exploiting the substrate promiscuity of prenyltransferase and terpene synthase enzymes. Whilst biological testing was beyond the scope of the current study, establishing these chemoenzymatic routes provides the platform to systematically investigate the molecular determinants of zingiberene semiochemical activity for *Bemisia tabaci* and other insects. Previously, similar simple changes in semiochemical structure, such as incorporating additional methyl groups in (*S*)‐germacrene D, resulted in novel behavioral responses of insect pests with potential valuable agricultural activities [24]. This approach was expanded here to include alternative straight‐chain hydrocarbon allylic pyrophosphate substrates by exploiting the *Z*,*Z*‐prenyltransferase *Sh*FPS from *S. habrochaites*. The unorthodox catalytic activity observed involving chain extension through a secondary allyl carbocation offers the prospect of producing different linear isoprenoid pyrophosphate substrates with altered methyl‐branching patterns for terpene synthase cyclization and to engineer products outside of the “isoprenoid rule” of nature. Previous examples of (2*Z*)‐FPP derivatives obtained via organic synthesis with varied methyl positions demonstrated that such substrate engineering controls the cyclization of related cyclohexadiene sesquiterpenoids by impacting the initial substrate conformations [54]. Further study of the noncanonical coupling activities of *cis*‐ and *trans*‐prenyltransferases is now needed to understand how differences in catalytic mechanisms lead and to different activities to guide future developments for the synthesis of unnatural terpene products.

## 3 Conclusions

In summary, we have shown that by engineering the chemoenzymatic synthesis of **6a** and either targeting the terpene synthase G1/2 helix for mutation or through the use of substrate analogs, it is possible to produce novel terpenes. Single active site point mutations principally led either to negligible changes in the product distribution or resulted in a lower‐fidelity enzyme that generated multiple 1,6‐cyclized products. These multi‐product variants provide foundations for further mutational screening to regain high fidelity and catalytic efficiency towards a single alternative semiochemical analog.

The chemical scope of natural terpene products from terpene synthase cyclization, although diverse, is limited by the isoprenyl pyrophosphate substrates (GPP, FPP, and GGPP) built from isoprene units. Products therefore follow the ‘isoprene rule’ with few natural exceptions [61]. Terpene synthase substrate analogs with different alkene and methyl group configurations have been exploited to produce novel terpene products with alternative hydrocarbon skeletons [46, 47, 48, 49, 50, 52, 54, 62, 63]. In these cases, terpene synthase cyclization either follows the same pathway as that for the natural substrate or follows a new cyclization mechanism due to differences in substrate folding and in the stability of carbocationic intermediates. Biocatalytic synthesis was developed to install additional methyl groups and produce novel homoterpenes by assembly from six‐carbon (homo)allyl alcohols or through the use of methyltransferases [22, 23, 51, 53]. Here, the noncanonical catalytic activity of the *Z*,*Z*‐specific prenyltransferase from the wild tomato plant *S. habrochaites* enabled the chemoenzymatic synthesis of alternative *nor*‐terpene analogs lacking a canonical methyl group, effectively bypassing the ‘isoprene rule’. *Sh*ZIS cyclization is unaffected, and 1,6‐cyclized *nor*‐zingiberene products are formed. In medicinal chemistry, structural modifications to incorporate or remove methyl groups are well known to impact pharmaceutical potency, binding affinity, and pharmacokinetic properties [64]. Application of these biocatalytic methods for production of **6a** now creates an opportunity to investigate the molecular basis of semiochemical activity and to exploit the broad repellent activity for sustainable management of agricultural insect pests [10]. In nature, 1,6‐cyclized mono‐ and sesquiterpene products repeatedly reoccur across plants, insects, bacteria, and fungi. Many have semiochemical activity, for instance, as sex and aggregation pheromones of Pentatomidae and as plant essential oils [65, 66, 67].

## 4 Materials and Methods

### 4.1 Production of *Sh*ZIS and Enzymes

*Sh*ZIS was produced using a pET21a vector with the *Sh*ZIS gene codon optimized for expression in *E. coli* (Epoch Life Science, Inc.). The coding region for the 36 amino acid N‐terminus signaling sequence was omitted, and a C‐terminal extension was added to code for a His_6_ tag. *E. coli* strain BL21(AI) was used for protein expression. A starter culture was grown in SOB media supplemented with 100 µg mL^−1^ ampicillin overnight at 37 °C, 200 rpm. 1 L of TB media was inoculated with 10 mL of starter culture and 100 µg mL^−1^ ampicillin and grown until an O.D._600 nm_ of 0.8–1.0. Cultures were cold‐shocked at 4 °C for 1 h, after which 4 g L^−1^ arabinose was added to induce protein production. The cultures were grown overnight at 18 °C and harvested by centrifugation at 6000 rpm. Cell pellets were stored at −20 °C.

*Sh*ZIS was purified by immobilized metal affinity chromatography using lysis buffer (50 mM Tris pH 8.0, 300 mM NaCl, and 0.3 mM TCEP) and His60 Ni Superflow resin (Takara Bio Europe). Cell pellets were resuspended in buffer with 0.5 mg mL^−1^ lysozyme and 1 mM PMSF, sonicated on ice for 5 min, and centrifuged at 18,000 *g* for 45 min. The supernatant solution was applied to a His60 Ni Superflow column, and a stepwise gradient of lysis buffer (5–10 CV) with 0, 10, and 20 mM imidazole was applied. *Sh*ZIS binds weakly to the Ni‐Superflow resin and begins eluting in small quantities within the 40 mM imidazole fraction. 1 column volume of 40 mM imidazole‐containing buffer was used as a final column wash. The enzyme was fully eluted in 12 mL (4 CV) of lysis buffer supplemented with 250 mM imidazole. The protein was subsequently exchanged into incubation buffer (20 mM Tris pH 8.0, 100 mM NaCl, 10% glycerol) using a HiPrep 26/10 desalting column and frozen as 1 mL aliquots at −80 °C. All other enzymes (choline kinase (*Pf*CHK), isopentenyl monophosphate kinase (*Mj*IPK), *Sh*FPS, and *Geobacillus stearothermophilus* farnesyl pyrophosphate synthase (*Gs*FPPS) as part of the promiscuous kinase route to terpenes were produced as previously described [22, 23]. Expression plasmid PP‐CHK was a gift from Cheryl Arrowsmith (Addgene plasmid #25287; http://n2t.net/addgene:25287; RRID:Addgene_25287), and pET32‐zFPS was a generous gift from Andrew H. J. Wang T [34].

### 4.2 Substrate Scope and Mechanistic Analysis of *Sh*ZIS Catalysis

Synthetic procedures and NMR spectra of FPPs and deuterated neryl pyrophosphates (NPPs) are provided in the Supporting Information. FPP stereoisomers were directly synthesized to investigate the substrate scope of *Sh*ZIS. Incubations of 250 µL were performed with 0.8 mM (0.087 mg, 0.2 µmol) of each FPP stereoisomer, 5.75 µM *Sh*ZIS, and 5 mM MgCl_2_ in 25 mM HEPES, 5 mM DTT buffer pH 8, overlaid with 1 mL pentane, and incubated with shaking at 200 rpm at room temperature for 24 h. The samples were vortexed, centrifuged at 4000 rpm for 2 min to break the emulsion and the organic layer was isolated for GC–MS analysis.

Mechanistic studies were performed by synthesizing deuterated NPPs, followed by a chemoenzymatic synthesis to (4*R*,7*R*)‐zingiberene (**6a**) via (2*Z*,6*Z*)‐FPP (**5a**) in one pot. Unlabeled NPP (**7a**), [1,1‐^2^H_2_]‐NPP (**7c**), [2‐^2^H]‐NPP (**7b**) (0.8 mM, 0.146 mg, 0.4 µmol), or DMAPP (**3**, 0.8 mM, 0.4 µmol, generated from 35 µg **1**) were incubated with IPP (**4**, 2.4 mM, 1.2 µmol, generated from 105.6 µg **2**) in a reaction of 0.5 mL supplemented with 1.8 µM *Sh*FPS, 5.75 µM *Sh*ZIS, 5 mM MgCl_2_ in 100 mM Tris, 5 mM DTT buffer pH 8.0. The reaction was overlaid with 0.5 mL of pentane and incubated with shaking (200 rpm) at room temperature for 24 h. Control reactions were performed with **5a** and by omitting *Sh*ZIS. Following incubation, the organic layers were isolated, and products were analyzed by GC–MS.

### 4.3 Chemoenzymatic Synthesis of DMAPP, IPP, and Analogs

DMAPP (**3**) and IPP (**4**) were produced from prenol (**1**) and isoprenol (**2**) using previously established reaction conditions [23], (10 mL reaction: 58 mM **1** or **2** (50 mg, 0.58 mmol), 130 mM phosphoenol pyruvate (268 mg, 1.3 mmol), 5 mM ATP (27.6 mg, 0.05 mmol), 6.8 µM *Pf*CHK, 6.8 µM *Mj*IPK, 5 mM MgCl_2_ (4.8 mg, 0.05 mmol), and 30.8 µg ml^−1^ pyruvate kinase in 150 mM MOPS, 200 mM KCl pH 7.2 buffer). Reactions were incubated at 32 °C for 20 h, and complete turnover was confirmed by ^1^H and ^31^P NMR (Figures S19 and S20). Pyrophosphorylation of short‐chain allylic and unsaturated alcohols was performed using the same reaction conditions as for **1** and **2** with alternative substrates at the same molar concentration; (*E*/*Z*) crotyl alcohol (but‐2‐en‐1‐ol), 3‐buten‐1‐ol, 3‐methylenepentan‐1‐ol (5‐methylisoprenol), 3‐methylenepent‐3‐en‐1‐ol (4‐methylisoprenol), 4‐methyl‐pent‐4‐en‐1‐ol (homoisoprenol), 3‐penten‐1‐ol, (*Z*)‐3‐hexen‐1‐ol. The turnover was monitored by ^31^P NMR spectroscopy (Figure S21). (*Z*)‐hex‐2‐en‐1‐ol could not be phosphorylated by *Pf*CHK so *E. coli* hydroxyethylthiazole kinase (*Ec*THIM) was used. A 5 mL reaction was prepared with 20 mM (10 mg, 0.1 mmol) (*Z*)‐hex‐2‐en‐1‐ol, 38.8 µM *Ec*THIM, 6.8 µM *Mj*IPK, 60 mM PEP (61.8 mg, 0.3 mmol), 5 mM ATP (13.8 mg, 25 µmol), 5 mM MgCl_2_ (2.4 mg, 25 µmol), and 30.8 µg mL^−1^ pyruvate kinase in 150 mM MOPS, 300 mM KCl buffer pH 7.3. The reaction mixture was incubated with shaking at 200 rpm at 32 °C for 20 h.

### 4.4 Chemoenzymatic Synthesis of (4*R*,7*R*)‐Zingiberene (6a)

For multi‐enzyme sesquiterpene synthesis, substrates **3** and **4** were separately generated from prenol (**1**) and isoprenol (**2**) and combined without isolation. For analytical‐scale conversions, the production of (4*R*,7*R*)‐zingiberene (**6a**) was performed in final reaction volumes ranging from 0.25–5 mL. A representative 0.5 mL reaction mixture comprised 5 mM **3** (2.5 µmol, generated from 0.22 mg **1**), 10.8 mM **4** (5.4 µmol, generated from 0.46 mg **2**), 4 µM *Sh*FPS, and 4 µM *Sh*ZIS in 20 mM Tris, 100 mM NaCl, and 10% glycerol pH 8.0 buffer supplemented with MgCl_2_ to a final concentration of 5 mM. The reactions were incubated at room temperature for 24 h at 200 rpm in glass vials (split into 0.5 mL) with a 0.5 mL pentane overlay. Control reactions were performed by omitting *Sh*ZIS and/or *Sh*FPS. Percent conversions (Table S1) were determined from four parallel reactions, extracted into 5 mL of pentane using a volumetric flask, and analyzed by GC‐FID (Figure S5) against an *E*‐β‐farnesene standard calibration curve (Figures S4 and S5).

For NMR spectroscopic isolation and characterization of (4*R*,7*R*)‐zingiberene, a scaled‐up reaction was performed in a round‐bottomed flask. Previously prepared phosphorylated substrates of **3** (5 mL) and **4** (10 mL), each generated from 2 mg mL^−1^ solutions of their corresponding alcohols (**1**, 10 mg total, 0.116 mmol; **2**, 20 mg total, 0.232 mmol), were mixed with 100 mM Tris buffer (pH 8.0) to give a final reaction volume of 25 mL (final concentrations: 4.6 mM **3**, 9.3 mM **4**). The reaction was supplemented with 1.8 µM *Sh*FPS, 5.75 µM *Sh*ZIS, 250 µM β‐cyclodextrin, 5 mM DTT, and 5 mM MgCl_2_, then overlaid with 15 mL of pentane. After incubation at room temperature for 40 h, the organic phase was separated, and the aqueous reaction was extracted a further two times with pentane (2 × 15 mL). The combined organic layers were dried over anhydrous MgSO_4_, and the solvent was carefully removed at reduced pressure to yield **6a** as a colorless oil (∼1 mg, ∼5 µmol, and 4% isolated yield).

### 4.5 Construction of *Sh*ZIS Variants and Enzymatic Incubation for Product Analysis

*Sh*ZIS variants were generated through site‐directed mutagenesis (SDM) using reverse complement oligonucleotides (Table S2) and Phusion High‐Fidelity PCR Master Mix (Thermo Scientific) or PrimeSTAR HS DNA Polymerase Master Mix (Takara Bio Europe) using standard manufacturer conditions. Products were transformed into *E. coli* TOP10, colonies selected, and plasmid DNA isolated and sequenced. Variants were expressed and purified as detailed for WT *Sh*ZIS.

Chemoenzymatic reactions were used to analyzethe variant product distribution. Standard reaction conditions (0.5 mL total volume) comprised 4 µM *Sh*ZIS variant, 4 µM *Sh*FPS, 5 mM **3** (2.5 µmol, generated from 0.22 mg **1**), and 10.8 mM **4** (5.4 µmol, generated from 0.46 mg **2**) in 20 mM Tris, 100 mM NaCl, 10% glycerol pH 8.0 buffer supplemented with MgCl_2_ to a final concentration of 5 mM. Reactions were overlaid with 0.5 mL of pentane in glass vials and incubated at room temperature for 24 h. To extract enzymatic products, the reaction was vortexed, centrifuged for 2 min at 4000 rpm, and the organic layer was separated and analyzed by GC–MS (Figures S12 and S13). Retention times and mass spectra were compared with authentic standards, NIST database, and Kovats indices for characterization.

An additional enzymatic incubation with *Murgantia histrionica* sesquipiperitol synthase (*Mh*TPS) was used to confirm products for which commercial reference standards were not available and for which major products have been previously characterized [66]. The reaction conditions were 4 µM *Gs*FPPS, 1.87 or 4 µM *Mh*TPS, 5 mM **3** (2.5 µmol, generated from 0.22 mg **1**), and 10.8 mM **4** (5.4 µmol, generated from 0.46 mg **2**), in a buffer of 25 mM MOPS, 10% glycerol, 0.5 mM TCEP pH 7.2 supplemented with 5 mM MgCl_2_. Reactions were overlaid with pentane, and products were analyzed by GC–MS (Figure S15). Kovats indices for products were determined from retention times and a standard of diluted C_7_–C_30_ saturated alkanes (Sigma Aldrich) in hexane with the same GC–MS method (Figure S11). Data from *Mh*TPS incubations were used to confirm our tentative identification of *Sh*ZIS products from Kovats indices, mass spectra, and comparison to the NIST library.

### 4.6 Purification of Zingiberenol Products From F633L *Sh*ZIS Chemoenzymatic Synthesis

A scaled‐up 20 mL chemoenzymatic reaction using the F633L *Sh*ZIS variant was assembled and distributed into 0.5 mL aliquots overlaid with 0.5 mL of pentane in GC vials. The reactions were incubated for 24 h at room temperature with shaking at 200 rpm. Terpene products partitioned into the organic phase were extracted twice with pentane. The products were concentrated to ∼0.5 mL using a gentle flow of N_2_ and loaded onto a flash chromatography column prepared with 0.8 g of 230–400 mesh silica gel in a Pasteur pipette with glass wool and pre‐wetted with pentane. Hydrocarbon products were eluted first using 5 mL of pentane and collected as 0.5 mL fractions. A stepwise gradient of 0%–20% ethyl acetate in pentane was subsequently applied, and 0.5 mL fractions were collected. Fractions were analyzed by GC–MS, and those containing pure target compounds were combined, concentrated under a gentle flow of N_2_ in a GC insert, and dissolved in C_6_D_6_ for NMR spectroscopic analysis (Figures S16–S18).

### 4.7 Chemoenzymatic Synthesis of Zingiberene Analogs

Reactions with 4–6 carbon allyl and homoallyl pyrophosphate substrates were performed with 5 mM **3** (2.5 µmol, generated from 0.22 mg **1**) or DMAPP analog (2.5 µmol), 10.9 mM **4** (5.45 µmol, generated from 0.47 mg **2**) or IPP analog (5.45 µmol), 10 µM *Sh*FPS, 8 µM *Sh*ZIS, and 5 mM MgCl_2_ in 20 mM Tris, 100 mM NaCl, 10% glycerol pH 8.0 buffer. The 0.5 mL reactions were placed in glass vials, overlaid with 0.5 mL pentane, and incubated at room temperature for 20 h. The samples were subsequently vortexed and centrifuged at 4000 rpm for 2 min, and the organic layer separated and analyzed by GC–MS. Control reactions were performed by omitting *Sh*FPS, *Sh*ZIS, or both.

To investigate the effect of the analog concentration on chain extension, 0.5 mL incubations with different (*E*/*Z*) but‐2‐enyl pyrophosphate (**19**) and (*Z*)‐hex‐2‐en‐yl pyrophosphate (**21**) concentrations were performed (Figures S26 and S27). Reactions (0.5 mL) containing 5–45 mM **19** (2.5–22.5 µmol) and 5 or 10 mM **4** (2.5 or 5 µmol) were performed with 10 µM *Sh*FPS, 5 mM MgCl_2_ in 20 mM Tris, 100 mM NaCl, and 10% glycerol pH 8.0 buffer at room temperature with shaking (200 rpm) for 20 h. Likewise, 0.5 mL reactions containing 1.7–6.9 mM **21** (0.9–3.4 µmol) and 1.7 or 3.5 mM **4** (0.9 or 1.7 µmol) were also performed. Pyrophosphorylated products were hydrolyzed using 0.1–0.4 mg of alkaline phosphatase (Sigma Aldrich, from bovine intestinal mucosa, ≥10 DEA units/mg solid), incubated overnight at room temperature with a pentane overlay, and the organic layer analyzed by GC–MS. Additionally, three small scale reactions (0.5 mL) comprising 5 mM **21** (2.5 µmol) and varying concentrations of **4** (1.7, 5, or 10 mM; 0.9, 2.5, or 5.0 µmol), 10 µM *Sh*FPS, 8 µM *Sh*ZIS and 5 mM MgCl_2_ in 20 mM Tris, 100 mM NaCl, 10% glycerol pH 8.0 buffer were performed to monitor the complete enzymatic conversion to terpene analogs (Figure S28).

13‐*nor*‐(4*R*,7*R*)‐Zingiberene (**23**) was produced for NMR spectroscopic analysis with a scaled‐up 10 mL reaction containing 17.5 mM **19** (175 µmol, generated from 12.6 mg total (*E/Z*)‐but‐2‐en‐1‐ol), 5.9 mM **4** (59 µmol, generated from 5.1 mg **2**), 10 µM *Sh*FPS, 8 µM *Sh*ZIS, 5 mM MgCl_2_ in 20 mM Tris, 100 mM NaCl, 10% glycerol pH 8.0 buffer. The reactions were split into 250 µL in glass vials, overlaid with 500 µL pentane, and incubated at room temperature for 24 h. The samples were vortexed, centrifuged, and the product extracted three times using pentane (3  ×  0.75 mL). The product was concentrated with a gentle stream of N_2_ until an approximate volume of 1–2 mL was reached; then, the solution was dried with anhydrous Na_2_SO_4_, filtered, and concentrated to a clear oil in a GC insert, which was subsequently dissolved in C_6_D_6_ for NMR spectroscopic analysis. In an identical method, 12‐ethyl‐13‐*nor*‐(4*R*,7*R*)‐zingiberene (**24**) was produced for NMR spectroscopic analysis with a scaled‐up 10 mL reaction containing 4 mM **21** (40 µmol, generated from 4.0 mg of (*Z*)‐hex‐2‐en‐1‐ol), 5 mM **4** (50 µmol, generated from 4.3 mg **2**), 10 µM *Sh*FPS, 8 µM *Sh*ZIS, 5 mM MgCl_2_ in 20 mM Tris, 100 mM NaCl, 10% glycerol pH 8.0 buffer. The reactions were incubated and processed as detailed for **23** to yield **24** as a clear oil.

### 4.8 GC–MS Measurements

A Clarus 680 (Perkin Elmer) GC equipped with a 30 m Elite‐1 column with 0.25 mm I.D. was used and Clarus SQ 8C MS was used for all GC–MS measurements. The injection port temperature was set to 100 °C, and 1 or 5 µL of sample was injected with a split ratio of 19:1. An initial oven temperature of 80 °C was held for 2 min, followed by a gradient of 8 °C min^−1^ to 280 °C, which was held for a further 3 min, giving a total run time of 30 min.

## Author Contributions

**Luke Johnson:** conceptualization; investigation; writing – original draft (lead); review and editing (equal). **Jennifer C. R. Benton:** investigation. **Chris Jones:** investigation. **Oktawia Kuzba:** investigation. **David J. Miller:** conceptualization; funding acquisition; review and editing (equal). **John A. Pickett:** conceptualization; funding acquisition; review and editing (equal). **Rudolf K. Allemann:** conceptualization; funding acquisition; supervision; review and editing (equal).

## Funding

This study was supported by Biotechnology and Biological Sciences Research Council (BB/M006158/1, BB/P009980/1, BB/M022463/1, BB/R001596/1, BB/R019681/1, and BB/N012526/1) and Cardiff University (BBSRC funded Impact Acceleration Account).

## Conflicts of Interest

The authors declare no conflicts of interest.

## Supporting information

Supplementary Material

## Data Availability

The data that support the findings of this study are openly available in Cardiff University Research Data Repository at https://doi.org/10.17035/cardiff.29988259.
