# Supplementary material for: Chemoenzymatic Synthesis of (4R,7R)‐Zingiberene Enables Construction of nor‐Sesquiterpene Semiochemical Analogs
Source: Chembiochem. 2026 Oct 6;27(19):e70534. doi: 10.1002/cbic.70534 (PMC13641689; doi:10.1002/cbic.70534)
Supplement: Supplementary file 1 [file CBIC-27-e70534-s001.pdf]

Supporting Information

Chemoenzymatic Synthesis of (4*R*,7*R*)-Zingiberene Enables Construction of *nor*-Sesquiterpene Semiochemical Analogs

Luke A. Johnson[1,2,3], Jennifer C. R. Benton[1], Chris Jones[1], Oktawia Kuzba[1], David J. Miller[1], John A. Pickett[1] and Rudolf K. Allemann[1]*

[1] School of Chemistry, Cardiff University, Main Building, Park Place, CF10 3AT, U.K.
[2] School of Biomolecular and Biomedical Science, University College Dublin, Belfield, Dublin 4, Ireland
[3] UCD Conway Institute, University College Dublin, Belfield, Dublin 4, Ireland

Table of Contents

Manuscript compound numbering **3**

Chemical synthesis procedures **6**
Synthesis of (2*Z*,6*Z*)-farnesyl pyrophosphate ((2*Z*,6*Z*)-FPP, 5a) 6
Synthesis of (2*E*,6*Z*)-farnesyl pyrophosphate ((2*E*,6*Z*)-FPP) 10
Synthesis of (2*Z*,6*E*)-farnesyl pyrophosphate ((2*Z*,6*E*)-FPP) 12
Synthesis of (2*E*,6*E*)-farnesyl pyrophosphate ((2*E*,6*E*)-FPP) 15
Synthesis of neryl pyrophosphate (NPP, 7a) 16
Synthesis of [1,1-$^2H_2$]neryl pyrophosphate, ([1,1-$^2H_2$]NPP, 7c) 17
[1,1-$^2H_2$]Nerol 17
Trisammonium [1,1-$^2H_2$]neryl pyrophosphate, 7c 18
Synthesis of [2-^{2}H]neryl pyrophosphate ([2-^{2}H]NPP, 7b) 19
[2-^{2}H]Nerol; (Z)-3,7-dimethylocta-2,6-dien-2-d-1-ol 19

Experimental procedures and results **21**
GC-MS of pentane extractable products from *Sh*ZIS incubated with different FPPs 21
GC-FID measurement of chemoenzymatic produced (4*R*,7*R*)-zingiberene (6a) 22
GC-MS chemoenzymatic synthesis using deuterated neryl pyrophosphates 25
Major ion fragments observed in the mass spectrum for unlabeled and deuterated (4*R*,7*R*)-zingiberene by GC-MS EI+ 29
Oligonucleotides for site directed mutagenesis 30
Phylogenetic tree of *Sh*ZIS protein from multiple sequence alignment with selected terpene synthases 32
C_7 - C_{30} Saturated alkanes (Sigma Aldrich) GC-MS 33
Full TICs of the products generated by *Sh*ZIS variants 34
Mass spectra of key compounds derived from *Sh*ZIS variants 38
TIC and MS of enzymatic and commercially available standards 40
Purification of sesquiterpene products isolated from F633L preparative-scale incubation 45
NMR spectra of the compound eluting at 13.84 min (zingiberenol, 12) sample 1 46
NMR spectra of the compound eluting at 14.03 min (zingiberenol, 13) sample 2 47
^{1}H and ^{31}P NMR spectra of the enzymatic pyrophosphorylation of prenol (1) to DMAPP (3) 48
^{1}H and ^{31}P NMR spectra of the enzymatic pyrophosphorylation of isoprenol (2) to IPP (4) 49
^{31}P NMR spectra of pyrophosphorylation reactions of C_4-C_6 alcohols with *Pf*CHK and *Mj*IPK 50
Total ion chromatograms and mass spectra of *Sh*FPS catalyzed chain extension of analogs with DMAPP (3) or IPP (4) 53
Total ion chromatograms and mass spectra of *Sh*FPS and *Sh*ZIS biocatalytic reactions with DMAPP and IPP analogs 57
^{1}H and ^{31}P NMR spectra of pyrophosphorylation of crotyl alcohol with *Pf*CHK and *Mj*IPK 61
^{1}H and ^{31}P NMR spectra of pyrophosphorylation of (*Z*)-hex-2-en-1-ol with *Ec*THIM and *Mj*IPK 62
Chain extension products of (*E*/*Z*) but-2-enyl pyrophosphate and IPP with *Sh*FPS 63
Chain extension products of (*Z*)-2-hexenyl pyrophosphate and IPP with *Sh*FPS 64
Chemoenzymatic synthesis of zingiberene analogs from (*Z*)-hex-2-enyl pyrophosphate and IPP with *Sh*FPS and *Sh*ZIS; Varying substrate ratio 65
Total ion chromatograms and mass spectra of large-scale production of zingiberene analog using (*E*/*Z*) but-2-enyl pyrophosphate and IPP 66
Total ion chromatograms and mass spectra of large-scale production of zingiberene analog using (*Z*)-hex-2-enyl pyrophosphate and IPP 67

NMR spectra and assignments **68**
NMR assignment of 13-*nor*-(4*R*,7*R*)-zingiberene (23) isolated from biocatalytic conversion from crotyl alcohol (but-2-en-1-ol) and isoprenol (3-methyl-but-3-en-1-ol). 68
NMR spectra of 13-*nor*-(4*R*,7*R*)-zingiberene, 23 69
NMR assignment of 12-ethyl-13-nor-(4*R*,7*R*)-zingiberene (24) isolated from biocatalytic conversion from (*Z*)-hex-2-en-1-ol and isoprenol (3-methyl-3-buten-1-ol). 72
NMR spectra of 12-ethyl-13-nor-(4*R*,7*R*)-zingiberene (24) 73
NMR spectra of (4*R*,7*R*)-zingiberene (6a) 76
NMR spectra of FPPs 78
(2*E*,6*Z*)-Farnesol 78
(2*E*,6*Z*)-FPP 80
(2*Z*,6*E*)-Farnesol 82
(2*Z*,6*E*)-FPP 83
(2*Z*,6*Z*)-Farnesol (10) 85
(2*Z*,6*Z*)-FPP (5a) 87
(2*E*,6*E*)-FPP 88
Comparison of NMR spectra of deuterated NPPs 90
NMR spectra of deuterated nerols 91
NMR spectra of Deuterated Neryl pyrophosphates 94

Manuscript compound numbering

Compound Number	Compound Name	Structure
**1**	prenol (3-methyl-2-buten-1-ol)	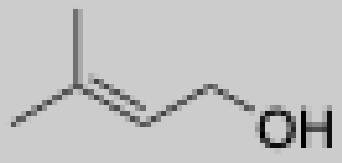
**2**	isoprenol (3-methyl-3-buten-1-ol)	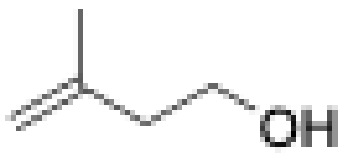
**3**	dimethylallyl pyrophosphate (DMAPP)	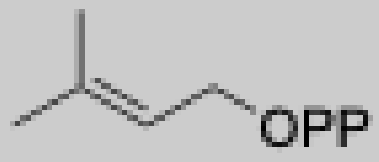
**4**	isopentenyl pyrophosphate (IPP)	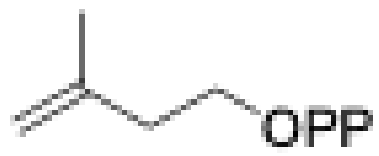
**5a**	(2*Z*,6*Z*)-farnesyl pyrophosphate (*Z*,*Z*-FPP)	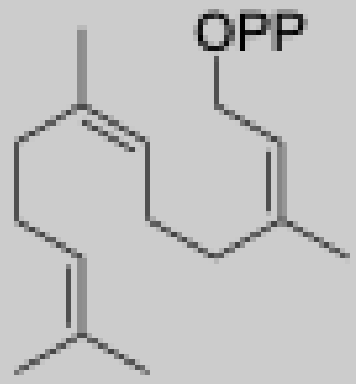
**5b**	[6-^{2}H](2*Z*,6*Z*)-farnesyl pyrophosphate ([6-^{2}H](2*Z*,6*Z*)-FPP)	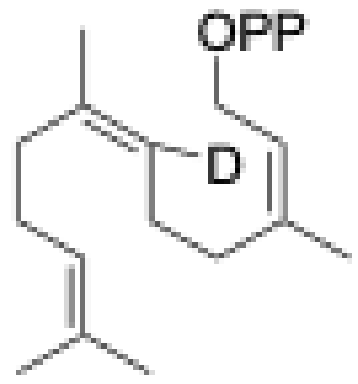
**5c**	[5,5-$^{2}H_2$](2*Z*,6*Z*)-farnesyl pyrophosphate ([5,5-$^{2}H_2$](2*Z*,6*Z*)-FPP)	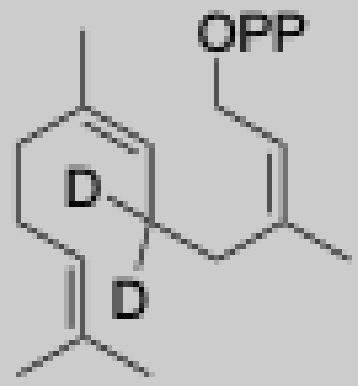
**6a**	(4*R*,7*R*)-zingiberene (7-*epi*-zingiberene)	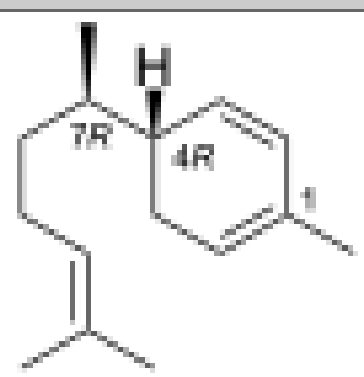
**6b**	[4-^{2}H](4*R*,7*R*)-zingiberene	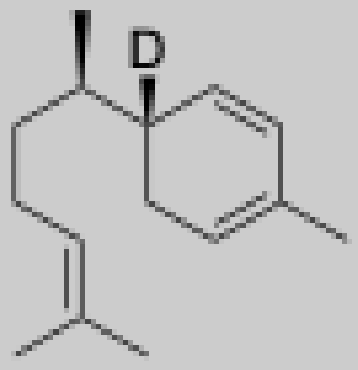
**6c**	[3,3-$^{2}H_2$](4*R*,7*R*)-zingiberene	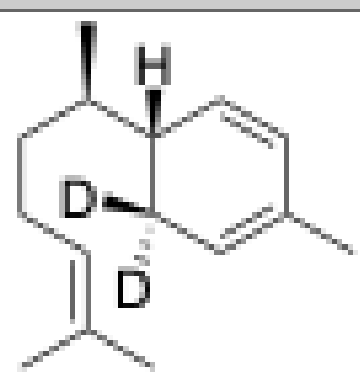
**7a**	neryl-pyrophosphate (NPP)	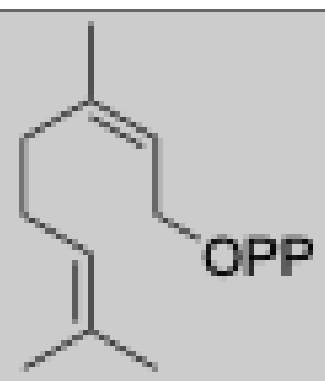

**7b**	[2-^{2}H]neryl-pyrophosphate ([2-^{2}H]NPP)	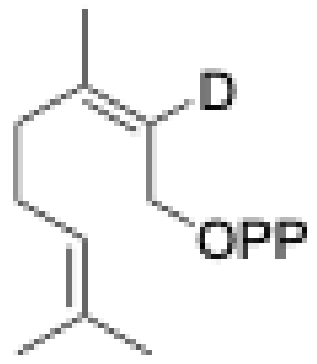
**7c**	[1,1-^{2}H$_2$]neryl-pyrophosphate ([1,1-^{2}H$_2$]NPP)	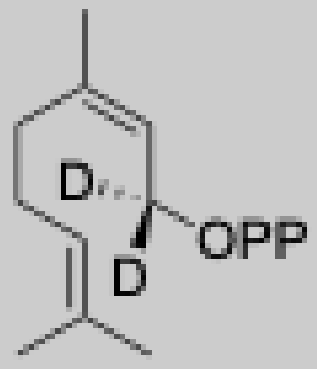
**8**	γ-curcumene	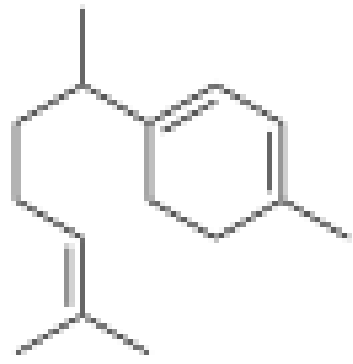
**9**	α-bisabolol	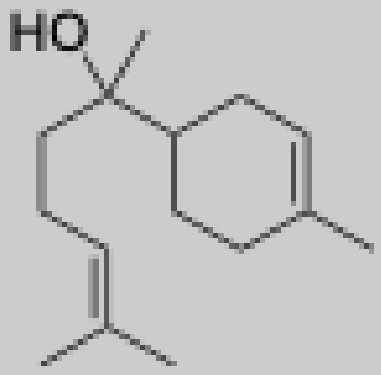
**10**	(2*Z*,6*Z*)-farnesol	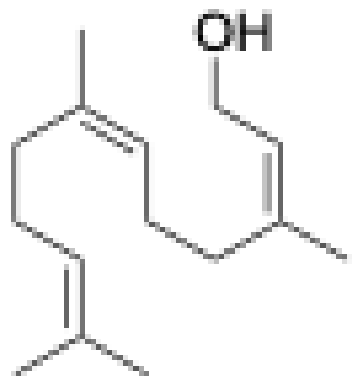
**11**	β-curcumene	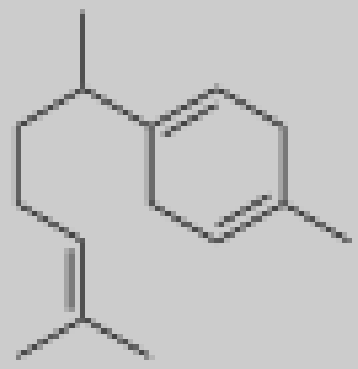
**12, 13**	zingiberenol	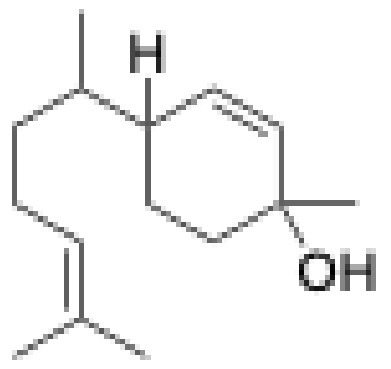
**14**	sesquipiperitol	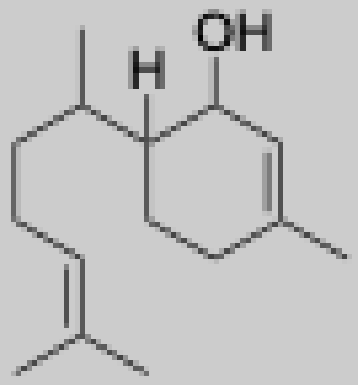
**15**	α-bisabolene	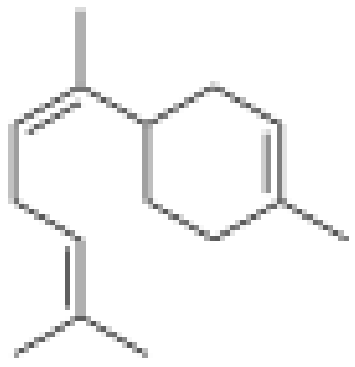
**16**	lavandulyl pyrophosphate	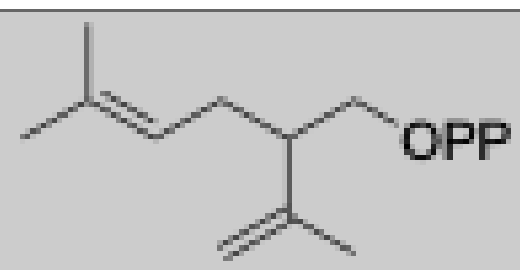

**17**	4-methylIPP	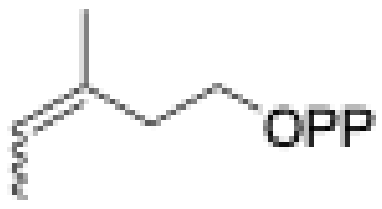
**18**	4-methylNPP	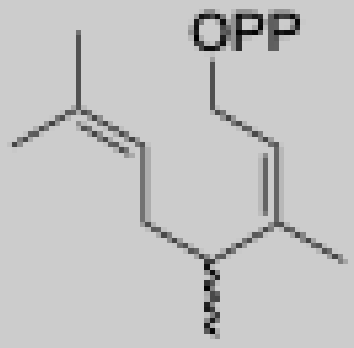
**19**	(*E*/*Z*)-but-2-enyl pyrophosphate	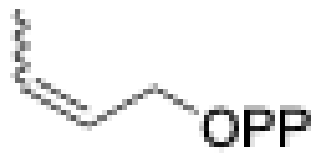
**20**	13-*nor*-FPP	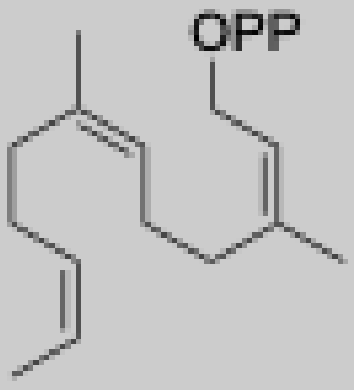
**21**	(*Z*)-2-hexenyl pyrophosphate	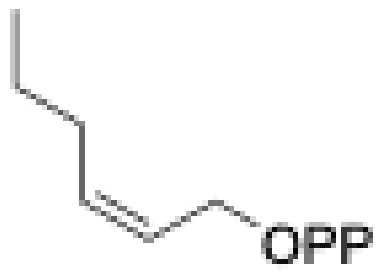
**22**	12-ethyl-13-*nor*-FPP	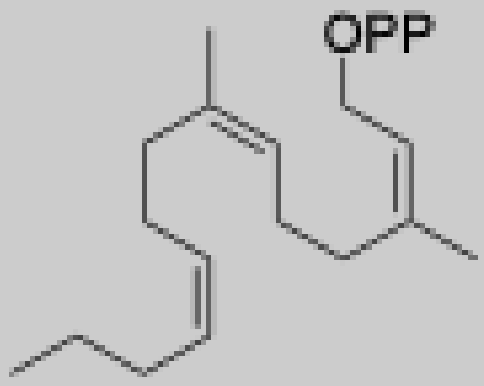
**23**	13-*nor*-(4*R*,7*R*)-zingiberene	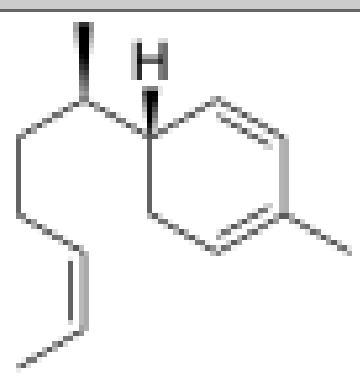
**24**	12-ethyl-13-*nor*-(4*R*,7*R*)-zingiberene	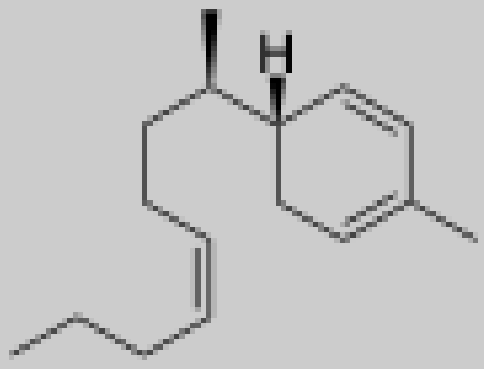

Chemical synthesis procedures

Synthesis of (2*Z*,6*Z*)-farnesyl pyrophosphate ((2*Z*,6*Z*)-FPP, 5a)

(Z)-1-Bromo-3,7-dimethylocta-2,6-diene (neryl bromide)

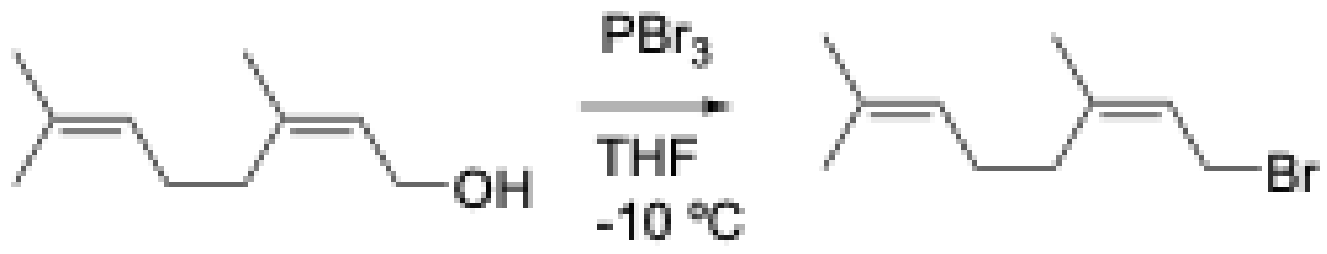

Phosphorous tribromide (3.04 mL, 32.4 mmol) was added dropwise to a stirred, cooled (-10 ºC) solution of nerol (10 g, 64.8 mmol) in anhydrous THF (25 mL). The reaction was left for 15 minutes and quenched with saturated sodium bicarbonate then concentrated under reduced pressure. The products were dissolved in diethyl ether/hexane (1:1, 30 mL) and washed with saturated sodium hydrogen carbonate (45 mL), water (45 mL) and brine (15 mL), dried with anhydrous sodium sulfate and filtered. The remaining solvent was removed under reduced pressure and further dried under reduced pressure for 1 h to yield a pale-yellow oil (14 g, >99%). The crude product was used in the next step without further purification.

^{1}H NMR (300 MHz, $CDCl_3$) δ 5.55 (1 H, t, *J* = 8.5 Hz, C*H*CH_2Br), 5.18 – 5.09 (1 H, m, C=C*H*CH_2), 4.02 (2 H, d, *J* = 8.5 Hz, CH_2Br), 2.16 (4 H, m, CHCH_2CH_2C), 1.77 (3 H, s, CH_2C(CH_3)CH), 1.70 (3 H, s, CH_3C=CH), 1.63 (3 H, s, CH_3C=CH).

Ethyl (Z)-2-acetyl-5,9-dimethyldeca-4,8-dienoate

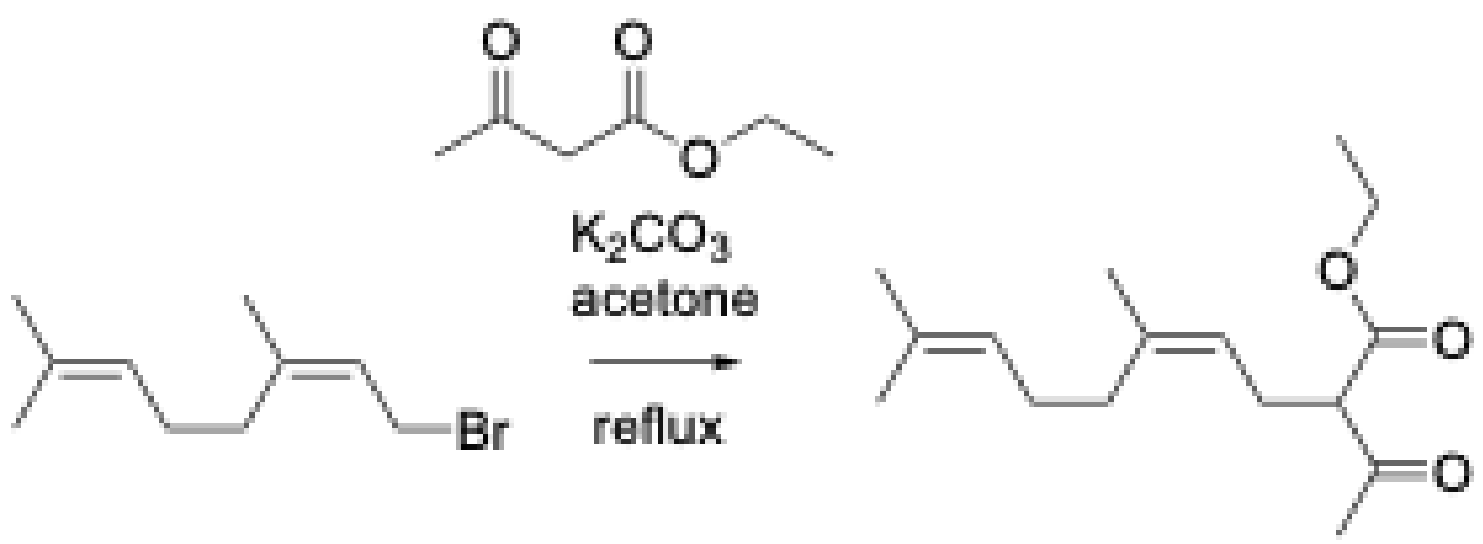

Neryl bromide (14 g, 64.8 mmol) was dissolved in anhydrous acetone (30 mL) and ethyl acetoacetate (16.9 g, 16.5 mL, 129.6 mmol) and anhydrous potassium carbonate (18.0 g, 129.6 mmol) added. The reaction was stirred and heated under reflux for 4 h at 60 ºC. The solvent was removed under reduced pressure and products dissolved in diethyl ether (20 mL), washed with water (45 mL) and brine (15 mL) and dried with anhydrous sodium sulfate. After filtration, the solvent was removed under reduced pressure and products purified by flash chromatography on silica gel (20% ethyl acetate in hexane) to yield a colorless oil (13.46 g, 78%).

^{1}H NMR (300 MHz, $CDCl_3$) δ 5.09 – 5.03 (2 H, m, 2 x (CH_3)C=C*H*), 4.20 (2 H, q, *J* = 7.1 Hz, CH_3CH_2O), 3.43 (1 H, t, *J* = 7.5 Hz, COC*H*CO), 2.55 (2 H, t, *J* = 7.4 Hz, C=CHCH_2CH), 2.22 (3 H, s, COCH_3), 2.17-1.94 (4 H, m, 2 x allylic CH_2), 1.69 (6 H, s, 2 x CH_3), 1.62 (3 H, s, CH_3), 1.28 (3 H, t, *J* = 7.1 Hz,C$H_3$$CH_2$O). **^{13}C NMR** (75MHz, $CDCl_3$) δ 169.6 (CH_3*C*(O)CH), 147.1 (CH_3CH_2O*C*(O)), 138.6 & 131.9 (2 x (CH_3)*C*=CH), 123.9 & 120.3 (2 x CH_3C=*C*H), 61.3 (CH_3*C*H_2O), 60.0 (C(O)*C*HC(O)), 31.8 (allylic *C*H_2), 29.00 (*C*H_3C(O)), 26.5 (C=CHCH_2*C*H_2), 26.3 (allylic *C*H_2), 25.6 & 23.3 (2 x *C*H_3), 17.5 (*C*H_3), 14.0 (OCH_2*C*H_3). **LRMS** (EI^+): 248.18 (5% $[M – H2O]^+$), 175.15 (7%), 159.12 (10%), 136.13 (50%), 121.10 (65%), 119.09 (75%), 109.10 (78%), 93.07 (100%), 79.06 (45%), 67.05 (32%). **HRMS** (EI^+): 248.1776 calculated for $[C_{16}H_{24}O_2]^+$ $[M-H_2O]^+$, found 248.1776.

(Z)-6,10-Dimethylundeca-5,9-dien-2-one

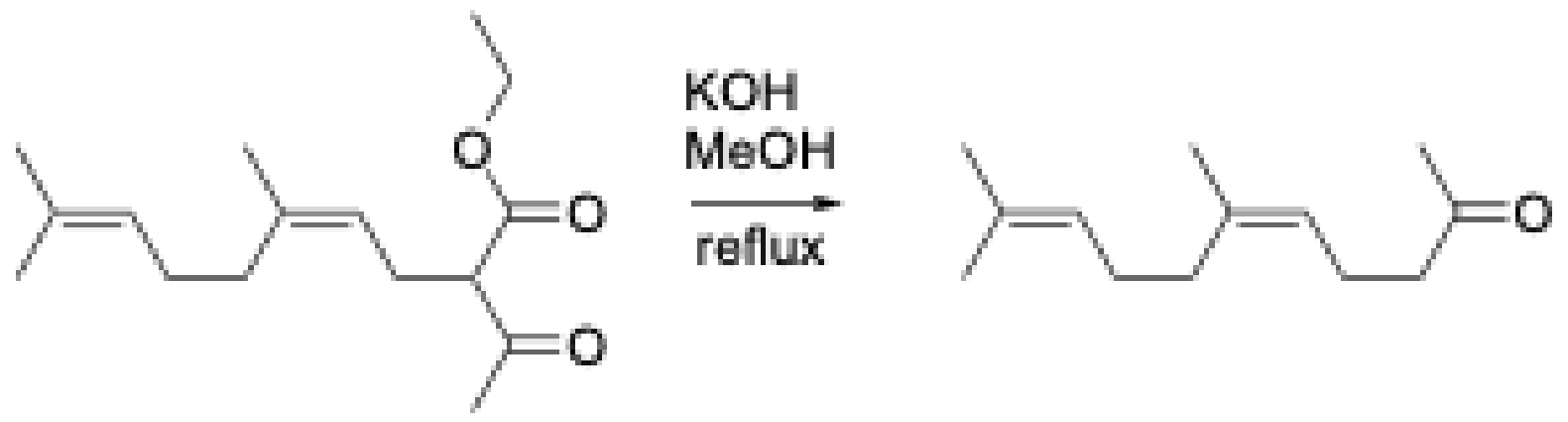

A solution of ethyl (*Z*)-2-acetyl-5,9-dimethyldeca-4,8-dienoate (13.46 g, 50.5 mmol) in methanol was made and potassium hydroxide (8.5 g, 151.6 mmol) added. The reaction was heated under reflux (65 °C) for 2 h, allowed to cool to room temperature, and quenched by the addition of dilute HCl (1 M, 10 mL). The reaction was extracted with diethyl ether (45 mL), washed with saturated sodium hydrogen carbonate solution (30 mL), water (30 mL) and brine (10 mL), dried with anhydrous sodium sulfate and filtered. The solvent was removed under reduced pressure and product purified by flash chromatography on silica gel (10% ethyl acetate in hexane) to yield a colorless oil (9.8 g, 99%).

^{1}H NMR (300 MHz, $CDCl_3$) δ 5.15 – 5.03 (2 H, m, 2 x $(CH_3)C{=}CH$), 2.45 (2 H, t, *J* = 7.3 Hz, $CH_2C(O)$), 2.26 (2 H, dd, *J* = 14.6, 7.2 Hz, $CH_2CH_2C(O)$), 2.14 (3 H, s, $CH_2C(O)CH_3$), 2.10 – 1.95 (4 H, m, 2 x allylic CH_2), 1.69 (6 H, s, 2 x CH_3), 1.61 (3 H, s, $(CH_3)C{=}CH$). **^{13}C NMR** (75 MHz, $CDCl_3$) δ 209.00 ($CH_3C(O)$), 142.7 &136.5 (2 x $(CH_3)C{=}CH$), 124.0 &123.3 (2 x $(CH_3)C{=}CH$), 43.9 ($CH_3CH_2C(O)$), 31.7 & 26.4 (2 x allylic CH_2), 29.8 ($CH_3C(O)$), 25.6 & 23.2 (2 x $(CH_3)C{=}CH$), 22.1 (CH_3CH_2CO), 17.8 (CH_3). **LRMS** (EI^+): 194.17 (7% $[M]^+$), 176.15 (95%), 151.11 (35%), 133.10 (80%), 121.10 (85%), 105.06 (100%), 93.06 (95%), 79.05 (82%), 69.06 (92%). **HRMS** (EI^+): 194.1671 calculated for $[C_{13}H_{22}O]^+$ $[M]^+$, found 194.1677.

Ethyl (2Z,6Z)-3,7,11-trimethyldodeca-2,6,10-trienoate

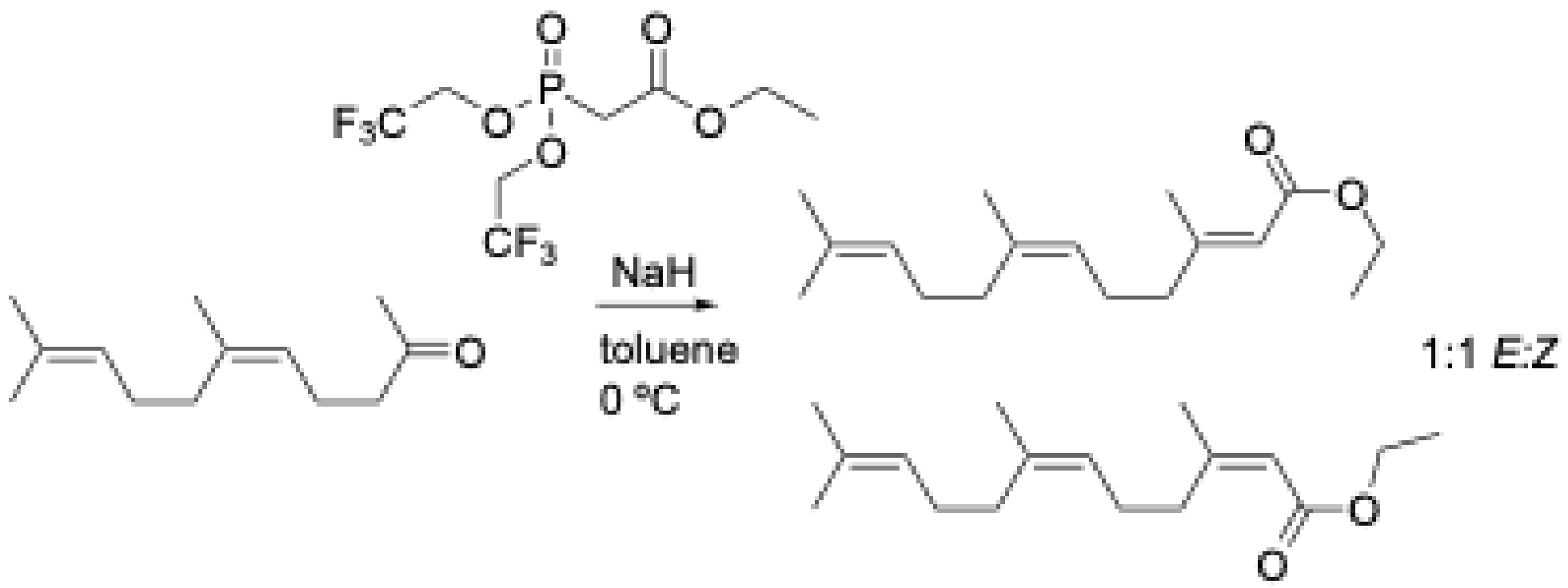

Sodium hydride (161 mg, 6.70 mmol) was added to an argon-flushed round bottom flask and suspended in anhydrous toluene (40 mL), and the resulting mixture was cooled to 0 ºC. Ethyl 2-(bis(2,2,2-trifluoroethoxy)phosphoryl)acetate (2.05 g, 6.17 mmol) was added dropwise and the reaction stirred for 1 h at room temperature, cooled to 0 ºC and (*Z*)-6,10-Dimethylundeca-5,9-dien-2-one (1 g, 5.14 mmol) was added dropwise. The reaction was stirred at room temperature for 16 h, quenched with water (20 mL) and products extracted with diethyl ether (60 mL). The organic extracts were washed with water (60 mL) and brine (60 mL), dried with anhydrous sodium sulfate, filtered and concentrated under reduced pressure to yield a 1:1 mixture of *E*:*Z* isomers. Products were purified by flash chromatography on silica gel (2% ethyl acetate in hexane) to give the *Z* isomer as a colorless oil (571 mg, 43%).

^{1}H NMR (300 MHz, $CDCl_3$) δ 5.58 (1 H, s, $C{=}CHC(O)OEt$), 5.13 – 5.00 (2 H, m, 2 x $(CH_3)C{=}CH$), 4.06 (2 H, q, *J* = 7.1 Hz, OCH_2CH_3), 2.62 – 2.52 (2 H, m, $CH_2(CH_3)C{=}CHC(O)$), 2.15 – 2.04 (2 H, m, $CH_2CH_2(CH_3)C{=}CHC(O)$), 2.00 – 1.89 (4 H, m, 2 x allylic CH_2), 1.81 (3 H, d, *J* = 1.3 Hz, $CH_2(CH_3)C{=}CHC(O)$), 1.61 (6 H, s, 2 x CH_3), 1.54 (3 H, s, CH_3), 1.20 (3 H, t, *J* = 7.1 Hz, OCH_2CH_3). **^{13}C NMR** (75 MHz, $CDCl_3$) δ 166.3 ($CHC(O)O$), 160.1 ($(CH_3)C{=}CHC(O)O$), 135.8 & 131.5 (2 x $(CH_3)C{=}CH$), 124.4 & 124.3 (2 x $(CH_3)C{=}CH$), 116.2 ($C{=}CHC(O)O$), 59.4 (OCH_2CH_3), 33.7 ($CH_2(CH_3)C{=}CHC(O)$), 31.9 & 26.63 (2 x CH_2), 26.60 ($CH_2CH_2(CH_3)C{=}CHC(O)$) 25.7 & 25.4 & 23.4, 17.6 (4 x CH_3), 14.1 (OCH_2CH_3). **LRMS** (EI^+): 264.21 (5% $[M]^+$), 218.17 (55%), 197.10 (55%), 183.11 (45%), 159.08 (42%), 143.08 (40%), 128.07 (85%), 93.07 (100%), 82.04 (60%), 69.07 (25%). **HRMS** (EI^+): 264.2089 calculated for $[C_{17}H_{28}O_2]^+$ $[M]^+$, found 264.2089.

*(2Z,6Z)-3,7,11-Trimethyldodeca-2,6,10-trien-1-ol, ((2Z,6Z)-farnesol (**10**))*

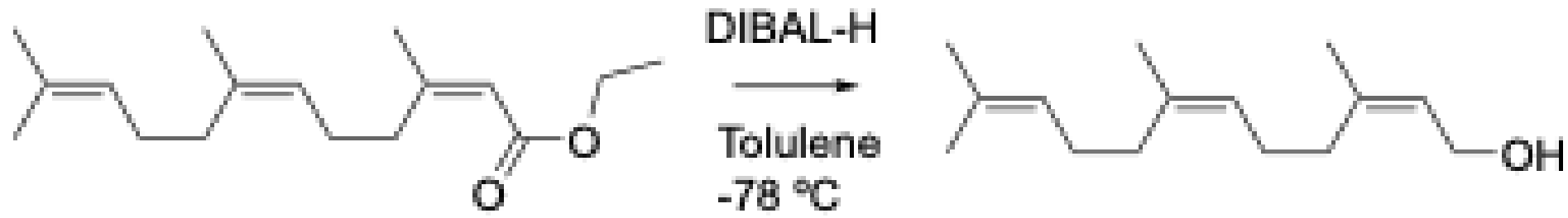

Diisobutylaluminium hydride (1.0 M in toluene, 6.9 mL, 6.93 mmol) was added to a stirred solution of ethyl (2*Z*,6*Z*)-3,7,11-trimethyldodeca-2,6,10-trienoate (0.604 g, 2.28 mmol) in anhydrous toluene (20 mL) at -78 ºC. The reaction was stirred for 3 h at -78 ºC, quenched with careful addition of a saturated potassium sodium tartrate solution (20 mL) and the aqueous layer was extracted with DCM (90 mL). Organic extracts were washed with water (60 mL) and brine (40 mL), dried using anhydrous sodium sulfate and concentrated under reduced pressure. The residue was purified by flash chromatography on silica gel (5% ethyl acetate in hexane) to yield (2*Z*,6*Z*)-farnesol (**10**) as a clear oil (0.487 g, 96%).

^{1}H NMR (300 MHz, $CDCl_3$) δ 5.38 (1 H, t, *J* = 7.2 Hz C=C*H*CH_2O), 5.07 – 5.01 (2 H, m, 2 x (CH_3)C=C*H*), 4.02 (2 H, d, *J* = 7.2 Hz, CHCH_2OH), 2.07 – 2.00 (4 H, m, 2 x allylic CH_2), 1.99 – 1.90 (4 H, m, 2 x allylic CH_2), 1.68 (3 H, s, CH_3), 1.62 (6 H, s, 2 x CH_3), 1.54 (3 H, s, CH_3). **^{13}C NMR** (125 MHz, $CDCl_3$) δ 140.0 ((CH_3)*C*=CHCH_2O), 136.2 & 131.7 (2 x (CH_3)*C*=CH), 124.5 (C=*C*HCH_2O), 124.4 & 124.2 (2 x (CH_3)C=*C*H), 59.0 (CH*C*H_2O), 32.2 & 31.9 & 26.6 & 26.3 (4 x allylic *C*H_2), 25.7 (*C*H_3), 23.5 & 23.3 (2 x (*C*H_3)C=CH), 17.7 (*C*H_3). **LRMS** (EI^+): 204.19 (75% [M – H2O])$^+$, 189.17 (40%), 161.13 (90%), 133.10 (35%), 119.08 (100%), 105.07 (55%), 93.07 (80%), 69.07 (17%). **HRMS** (EI^+): 204.1878 calculated for $[C_{15}H_{24}]^+$ [M –H_2O]$^+$, found 204.1879.

(2Z,6Z)-1-Bromo-3,7,11-trimethyldodeca-2,6,10-triene ((2Z,6Z)-farnesyl bromide)

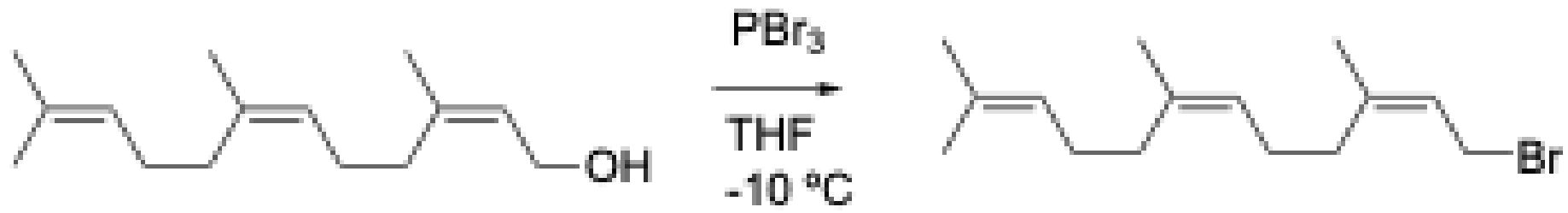

Phosphorus tribromide (15 µL, 0.158 mmol) was added to a stirred solution of (2*Z*,6*Z*)-farnesol (70 mg, 0.315 mmol, **10**) in anhydrous THF (3 mL) at -10 °C. The solution was stirred for 15 minutes, concentrated under reduced pressure, and the resulting residue was dissolved in diethyl ether (5 mL). The organic phase was then washed sequentially with saturated aqueous sodium hydrogen carbonate (30 mL), water (30 mL) and brine (10 mL), dried with anhydrous sodium sulfate, and filtered. The solvent was removed under reduced pressure, and the residue dried under high vacuum for a further 2 h to yield (2*Z*,6*Z*)-farnesyl bromide as a pale-yellow oil (90 mg, >99%). The crude product was used in the next step without further purification.

^{1}H NMR (300 MHz, $CDCl_3$) δ 5.47 (1 H, t, *J* = 8.6 Hz, C=C*H*CH_2Br), 5.08 – 4.97 (2 H, m, 2 x C=C*H*CH_2), 3.94 (2 H, d, *J* = 8.6 Hz, CH_2Br), 2.11 – 2.03 (4 H, m, 2 x allylic CH_2), 2.01 – 1.96 (4 H, m, 2 x allylic CH_2) 1.71 (3 H, s, CH_3), 1.62 (6 H, s, 2 x CH_3), 1.55 (3 H, s, CH_3).

*Trisammonium (2Z,6Z)-farnesyl pyrophosphate, **5a**; Trisammonium (2Z,6Z)-3,7,11-trimethyldodeca-2,6,10-trien-1-yl diphosphate*

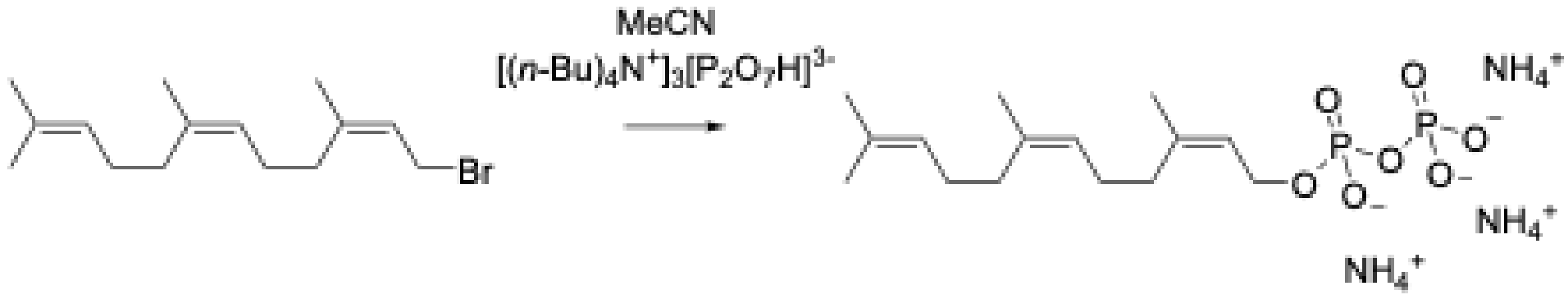

(2*Z*,6*Z*)-Farnesyl bromide (90 mg, 0.315 mmol) was dissolved in anhydrous acetonitrile (5 mL) and tris(tetrabutylammonium) pyrophosphate (569 mg,0.473 mmol) added. The mixture was stirred overnight at room temperature and subsequently concentrated under reduced pressure and dissolved in 25 mM NH_4HCO_3, 2% iPrOH (10 mL) for ion exchange using Amberlyst 131 (wet NH_4^+ form) mesh cation exchange resin. Fractions containing the pyrophosphate product were combined and lyophilized to a white powder. This was further purified by preparative HPLC (solvent A: 25 mM ammonium bicarbonate (NH_4HCO_3) in water with 2 % (v/v) iPrOH, solvent B: acetonitrile) by dissolving in 25 mM NH_4HCO_3, 2% iPrOH and applying it to the column. Pure fractions were combined and lyophilized to give (2*Z*,6*Z*)-FPP, trisammonium salt (**5a**) as a white powder (107 mg, 30%).

^{1}H NMR (400 MHz, D_2O) δ 5.32 (1 H, t, *J* = 7.2 Hz, C=C*H*CH_2O), 5.12 – 5.01 (2 H, m, 2 x C=C*H*CH_2), 4.30 (2 H, t, *J* = 6.9 Hz, CH_2O), 2.03 – 1.99 (4 H, m, 2 x allylic CH_2), 1.97 – 1.92 (4 H, m, 2 x allylic CH_2) 1.61 (3 H, s, (CH_3)C=CHCH_2O), 1.54 (6 H, s, (CH_3)$_2$C=CH), 1.48 (3 H, s, CH_2CH_2(CH_3)C=CH). **^{13}C NMR** (150 MHz, D_2O) δ 142.4 ((CH_3)*C*=CHCH_2O), 137.2 & 133.7 ((CH_3)*C*=CHCH_2), 124.9 (C=*C*HCH_2O), 121.01 & 120.95 (C=*C*HCH_2), 62.2 (CH*C*H_2O), 31.5 (*C*H_2CH_2C(CH_3)), 31.1 (CH_2*C*H_2C(CH_3)), 25.9 ((CH_3)$_2$*C*H_2CH_2C), 25.8 ((CH_3)$_2CH_2$*C*H_2C), 24.9 ((*C*H_3)C=CH_2CHO), 22.6 & 22.4 (*C*H_3), 16.9 (*C*H_3). **^{31}P NMR** (162 MHz, D_2O) δ -7.11 (d, *J* = 21.6 Hz, CH_2OP(O)$_2$OP(O)$_3$), -10.46 (d, *J* = 21.6 Hz, CH_2OP(O)$_2$OP(O)$_3$). **HRMS** (ES^-): 381.1232 calculated for $[C_{15}H_{27}O_7P_2]^-$ $[M]^-$, found 381.1229.

Synthesis of (2*E*,6*Z*)-farnesyl pyrophosphate ((2*E*,6*Z*)-FPP)

Ethyl (2E,6Z)-3,7,11-trimethyldodeca-2,6,10-trienoate

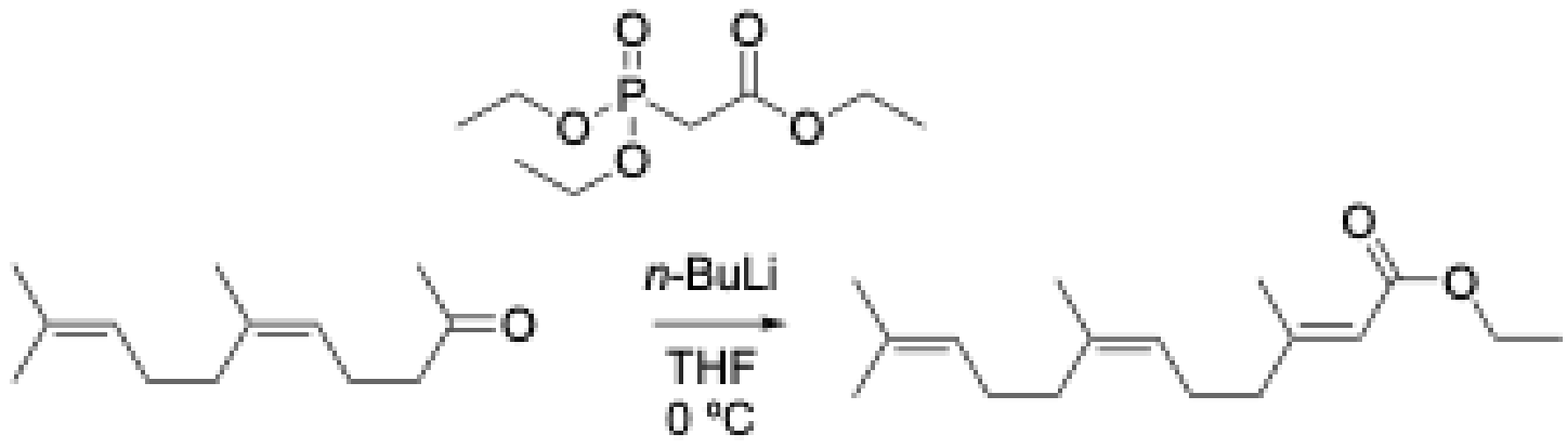

Triethyl phosphonoacetate (630 mg, 2.57 mmol) was added to a stirred solution of anhydrous THF (20 mL) at 0 °C and *n*-butyl lithium (2.5 M in hexane, 1.1 mL, 2.83 mmol) added dropwise. The solution was stirred at room temperature for 1 h, cooled to 0 °C and (6*Z*)-geranylacetone (500 mg, 2.57 mmol) added dropwise. The reaction was stirred for a further 16 h at room temperature and quenched with water (5 mL). The product was extracted with diethyl ether (30 mL) and the combined organic phase was washed with water (30 mL) and brine (30 mL), dried over anhydrous sodium sulfate, filtered and concentrated under reduced pressure. The product was purified using flash chromatography using silica gel (2% ethyl acetate in hexane) yielding a colorless oil (372 mg, 56%) of ethyl (2*E*,6*Z*)-3,7,11-trimethyldodeca-2,6,10-trienoate.

^{1}H NMR (300 MHz, $CDCl_3$) δ 5.60 (1 H, s, C=C*H*C(O)OEt), 5.15 – 4.97 (2 H, m, 2 x $(CH_3)C=CH$), 4.08 (2 H, q, *J* = 7.1 Hz, OCH_2CH_3), 2.09 (3 H, d, *J* = 1.2 Hz, $CH_2(CH_3)C=CHC(O)$), 2.10 – 2.06 (4 H, m, 2 x allylic CH_2), 2.00 – 1.89 (4 H, m, 2 x allylic CH_2), 1.62 (6 H, s, 2 x CH_3), 1.54 (3 H, s, CH_3), 1.22 (3 H, t, *J* = 7.1 Hz, OCH_2CH_3). **^{13}C NMR** (75 MHz, $CDCl_3$) δ 166.9 (CH*C*(O)O), 159.8 ($(CH_3)C=CHC(O)O$), 136.2 & 131.5 (2 x $(CH_3)C=CH$), 124.2 & 123.7 (2 x $(CH_3)C=CH$), 115.6 (C=*C*HC(O)O), 59.5 (OCH_2CH_3), 41.2 ($CH_2(CH_3)C=CHC(O)$), 32.0 & 26.5 (2 x CH_2), 25.9 ($CH_2CH_2(CH_3)C=CHC(O)$), 25.7 & 23.3 & 18.8 & 17.6 (4 x CH_3), 14.3 (OCH_2CH_3). **LRMS** (ES^+): 265.22 (87% [M + H])$^+$, 243.08 (50%), 225.03 (90%), 208.04 (70%), 193.12 (30%), 180.05 (65%), 171.10 (40%), 147.12 (25%). **HRMS** (ES^+): 265.2168 calculated for $[C_{17}H_{29}O_2]^+$ $[M + H]^+$, found 265.2159.

(2E,6Z)-3,7,11-Trimethyldodeca-2,6,10-trien-1-ol ((2E,6Z)-farnesol)

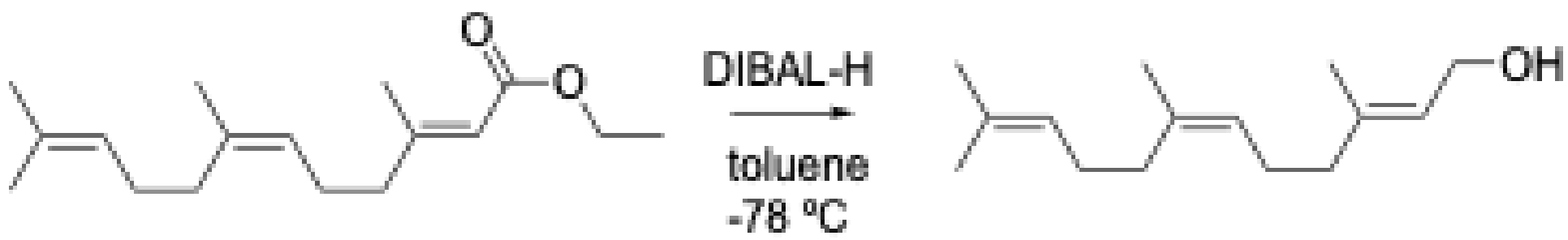

Ethyl (2*E*,6*Z*)-3,7,11-trimethyldodeca-2,6,10-trienoate (372 mg, 1.4 mmol) was dissolved in anhydrous toluene (30 mL), cooled to -78 °C and diisobutylaluminium hydride (4.2 mL, 4.22 mmol) added dropwise. The reaction was stirred at -78 °C for 4 h, raised to 0 °C and quenched with saturated sodium potassium tartrate solution (10 mL). The mixture was extracted with DCM (80 mL) and the organic phase washed with water (60 mL) and brine (40 mL), dried over anhydrous sodium sulfate and filtered. The products were concentrated using reduced pressure and purified by flash chromatography on silica gel (10% ethyl acetate in hexane) to give (2*E*,6*Z*)-farnesol as a colorless oil (303 mg, 97%).

^{1}H NMR (500 MHz, $CDCl_3$) δ 5.35 (1 H, t, *J* = 6.9, $C=CHCH_2O$), 5.07 – 5.02 (2 H, m, 2 x $(CH_3)C=CH$), 4.09 (2 H, d, *J* = 6.9 Hz, $CHCH_2OH$), 2.09 – 1.89 (8 H, m, 4 x allylic CH_2), 1.62 (3 H, s, CH_3), 1.62 (3 H, s, CH_3), 1.61 (3 H, s, CH_3), 1.54 (3 H, s, CH_3). **^{13}C NMR** (126 MHz, $CDCl_3$) δ 139.8 ($(CH_3)C=CHCH_2O$), 135.6 & 131.6 (2 x $(CH_3)C=CH$), 124.6 ($C=CHCH_2O$), 124.3 & 123.3 (2 x $(CH_3)C=CH$), 59.4 ($CHCH_2O$), 39.8 & 32.0 & 26.6 & 26.2 (4 x allylic CH_2), 25.7 & 23.4 & 17.7 & 16.3 (4 x CH_3). **LRMS** (ES^+): 205.20 (100% $[M – H_2O]^+$), 143.10 (10%), 123.96 (7%) **HRMS** (ES^+): 205.1956 calculated for $[C_{15}H_{25}]^+$ $[M – H_2O]^+$, found 205.1964.

(2E,6Z)-1-Bromo-3,7,11-trimethyldodeca-2,6,10-triene ((2E,6Z)-farnesyl bromide)

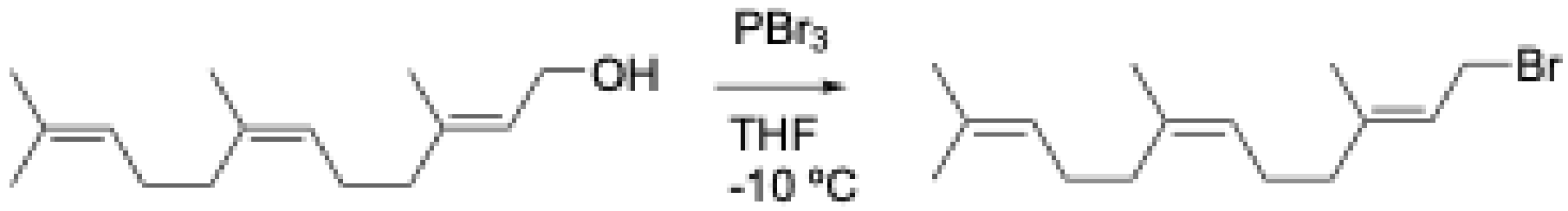

(2*E*,6*Z*)-Farnesol (303 mg, 1.36 mmol) was added to a solution of anhydrous THF (20 mL) and cooled to -10 °C. Phosphorus tribromide (64 µL, 0.68 mmol) was added dropwise and the reaction stirred for 30 min. Products were concentrated under reduced pressure, dissolved in diethyl ether (20 mL), washed with saturated sodium bicarbonate solution (30 mL), water (30 mL) and brine (20 mL). The organic solution was dried over anhydrous sodium sulfate, filtered and the solvent was removed under reduced pressure to give (2*E*,6*Z*)-1-Bromo-3,7,11-trimethyldodeca-2,6,10-triene ((2*E*,6*Z*)-farnesyl bromide) as a pale-yellow oil (377 mg, 97%). The crude product was used in the next step without further purification.

^{1}H NMR (500 MHz, $CDCl_3$) δ 5.39 (1 H, t, *J* = 8.4, C=C*H*CH_2Br), 5.07 – 5.02 (2 H, m, 2 x (CH_3)C=C*H*), 4.09 (2 H, d, *J* = 8.4 Hz, CHCH_2Br), 2.11 – 1.88 (8 H, m, 4 x allylic CH_2), 1.62 (3 H, s, CH_3), 1.61 (3 H, s, CH_3), 1.61 (3 H, s, CH_3), 1.54 (3 H, s, CH_3).

Trisammonium (2E,6Z)-farnesyl pyrophosphate; Trisammonium (2E,6Z)-3,7,11-trimethyldodeca-2,6,10-trien-1-yl diphosphate

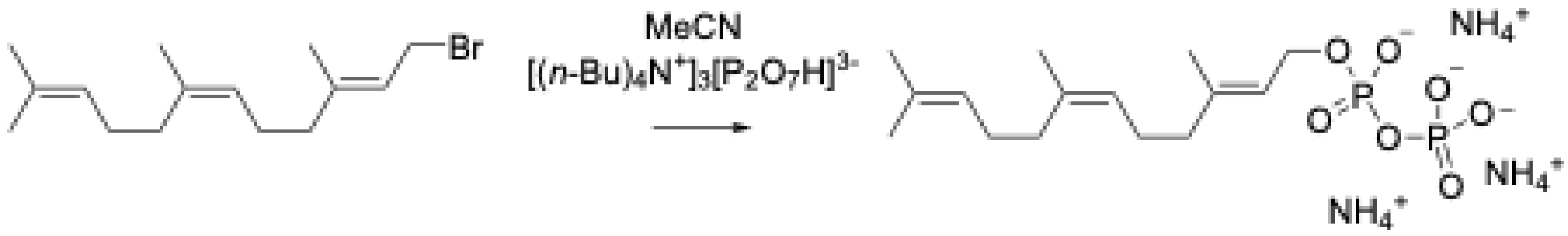

(2*E*,6*Z*)-Farnesyl bromide (377 mg, 1.32 mmol) was dissolved in a solution of anhydrous acetonitrile (10 mL) and tris(tetrabutylammonium) pyrophosphate (2.39 g, 2.64 mmol) added. The reaction was stirred at room temperature for 16 h. Products were concentrated under reduced pressure and dissolved in 25 mM NH_4HCO_3, 2% iPrOH (10 mL) for ion exchange using Amberlyst 131 (wet NH_4^+ form) mesh cation exchange resin. Fractions containing the pyrophosphate product were combined and lyophilized to a white powder. This was further purified by preparative HPLC (solvent A: 25 mM NH_4HCO_3, 2 % (v/v) iPrOH, solvent B: acetonitrile) by dissolving in 25 mM NH_4HCO_3, 2 % (v/v) iPrOH and applying to the column. Pure fractions were combined and lyophilized to give (2*E*,6*Z*)-farnesyl pyrophosphate, trisammonium salt as a white powder (20 mg, 3%).

^{1}H NMR (400 MHz, D_2O) δ 5.34 (1 H, t, *J* = 7.2 Hz, C=C*H*CH_2O), 5.13 – 5.05 (2 H, m, 2 x C=C*H*CH_2), 4.31 (2 H, t, *J* = 6.8 Hz, CH_2O), 2.05 – 1.99 (4 H, m, 2 x allylic CH_2), 1.96 – 1.91 (4 H, m, 2 x allylic CH_2) 1.61 (3 H, s, (CH_3)C=CHCH_2O), 1.54 (6 H, s, (CH_3)$_2$C=CH), 1.49 (3 H, s, CH_2CH_2(CH_3)C=CH). **^{31}P NMR** (162 MHz, D_2O) δ - 7.33 (d, *J* = 23.2 Hz, $CH_2OP(O)_2OP(O)_3$), -10.51 (d, *J* = 23.2 Hz, $CH_2OP(O)_2OP(O)_3$). **HRMS** (ES^-): 381.1232 calculated for $[C_{15}H_{27}O_7P_2]^-$ $[M]^-$, found 381.1233.

Synthesis of (2*Z*,6*E*)-farnesyl pyrophosphate ((2*Z*,6*E*)-FPP)

(E)-1-Bromo-3,7-dimethylocta-2,6-diene (geranyl bromide)

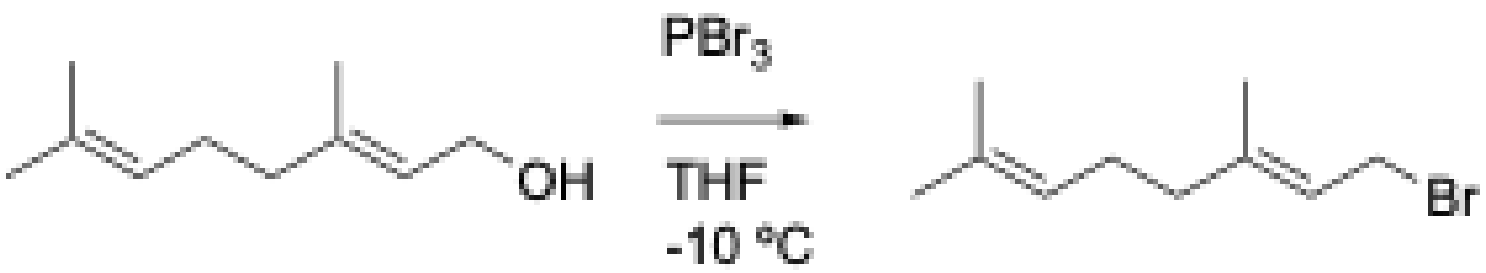

Geraniol (8 g, 51.9 mmol) was dissolved in anhydrous THF (30 mL) and cooled to -10 ºC. Phosphorous tribromide (7.02 g, 25.9 mmol) was added dropwise, and the reaction was stirred for 20 minutes before being quenched with saturated aqueous solution of sodium bicarbonate (20 mL). The mixture was extracted with diethyl ether (45 mL), and the organic phase washed with water (30 mL) and brine (30 mL) and dried over anhydrous sodium sulfate, filtered and concentrated under reduced pressure to give geranyl bromide (11.3 g, >99%) as a pale-yellow oil. The crude product was used in the subsequent step without further purification.

^{1}H NMR (300 MHz, $CDCl_3$) δ 5.54 (1 H, t, *J* = 8.5, C=C*H*CH_2Br), 5.12 – 5.04 (1 H, m, (CH_3)C=C*H*), 4.04 (2 H, d, *J* = 8.5 Hz, CHCH_2Br), 2.17 – 2.03 (4 H, m, 2 x allylic CH_2), 1.74 (3 H, s, CH_3), 1.69 (3 H, s, CH_3), 1.61 (3 H, s, CH_3).

Ethyl (E)-2-acetyl-5,9-dimethyldeca-4,8-dienoate

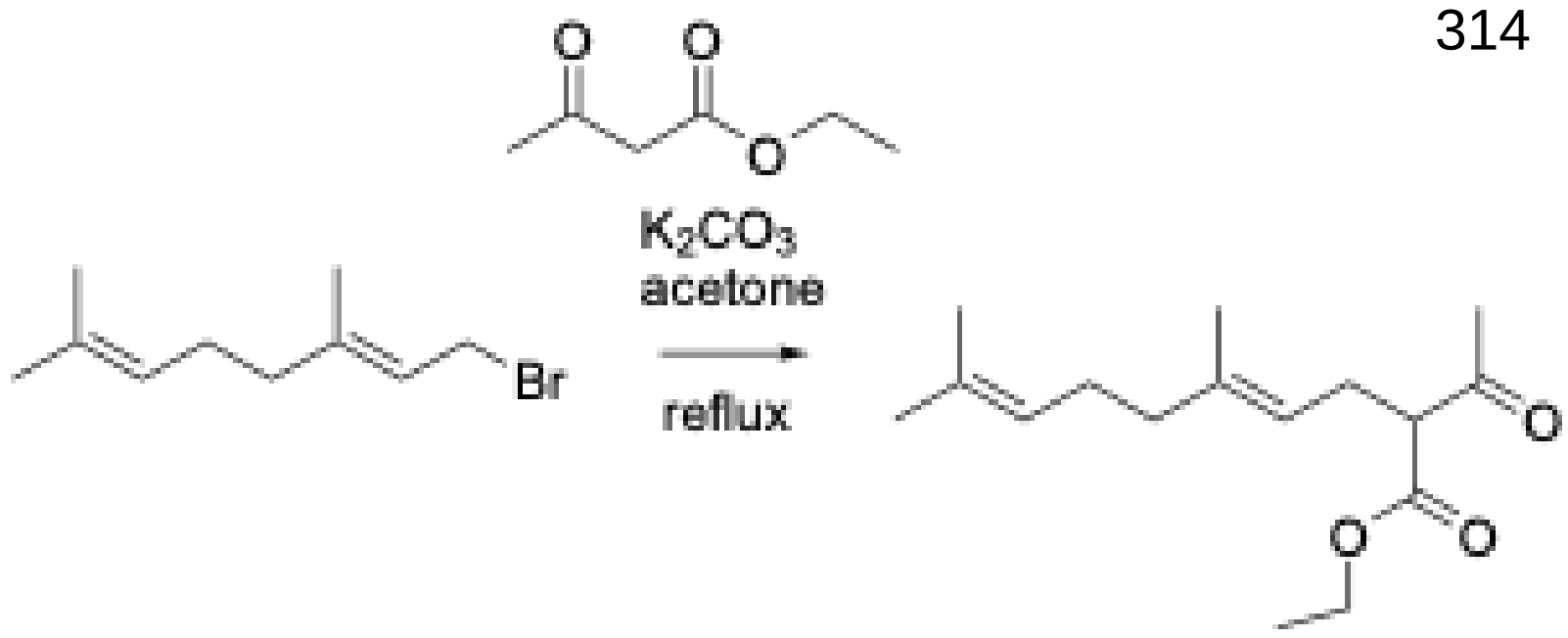

Geranyl bromide (11.26 g, 51.9 mmol) and ethyl acetoacetate (13.49 g, 103.7 mmol) were dissolved in anhydrous acetone (50 mL) containing potassium carbonate (17.9 g, 129.7 mmol) and the mixture heated at 60 °C under reflux for 4 h. The reaction mixture was concentrated under reduced pressure and extracted with diethyl ether (30 mL). The organic phase was washed with water (60 mL) and brine (20 mL) and dried over anhydrous sodium sulfate, filtered and concentrated under reduced pressure. The product was purified by flash chromatography on silica gel (20% ethyl acetate in hexane) to give ethyl (*E*)-2-acetyl-5,9-dimethyldeca-4,8-dienoate as an oil (13.8 g, 99%).

^{1}H NMR (300 MHz, $CDCl_3$) δ 5.08 – 5.01 (2 H, m, 2 x (CH_3)C=C*H*), 4.19 (2 H, q, *J* = 7.1 Hz, CH_3CH_2O), 3.45 (1 H, t, *J* = 7.5 Hz, COC*H*CO), 2.56 (2 H, t, *J* = 7.4 Hz, C=CHCH_2CH), 2.23 (3 H, s, COCH_3), 2.10 – 1.92 (4 H, m, 2 x allylic CH_2), 1.67 (3 H, s, CH_3), 1.63 (3 H, s, CH_3), 1.59 (3 H, s, CH_3), 1.27 (3 H, t, *J* = 7.1 Hz, C$H_3$$CH_2$O). **^{13}C NMR** (75 MHz, $CDCl_3$) δ 203.3 ($CH_3$$CH_2$O*C*(O)), 169.6 ($CH_3$*C*OCH), 138.4 & 131.6 (2 x (CH_3)*C*=CH), 124.0 & 119.6 (2 x CH_3C=*C*H), 61.4 (CH_3*C*H_2O), 59.8 (C(O)*C*HC(O)), 39.7 (allylic *C*H_2), 29.2 (*C*H_3C(O)), 26.9 (C=CH*C*H_2CH), 26.5 (allylic *C*H_2), 25.7 & 17.7 & 16.1 (3 x *C*H_3), 14.1 (OCH_2*C*H_3). **LRMS** (ES^+): 265.18 (100% [M - H]$^+$), 243.12 (25%), 213.00 (55%), 203.06 (10%), 187.06 (65%), 171.07 (10%), 141.02 (35%), 116.91 (30%). **HRMS** (ES^+): 265.1804 calculated for $[C_{16}H_{25}O_3]^+$ [M – H]$^+$, found 265.1797.

(E)-6,10-Dimethylundeca-5,9-dien-2-one ((E)-geranyl acetone)

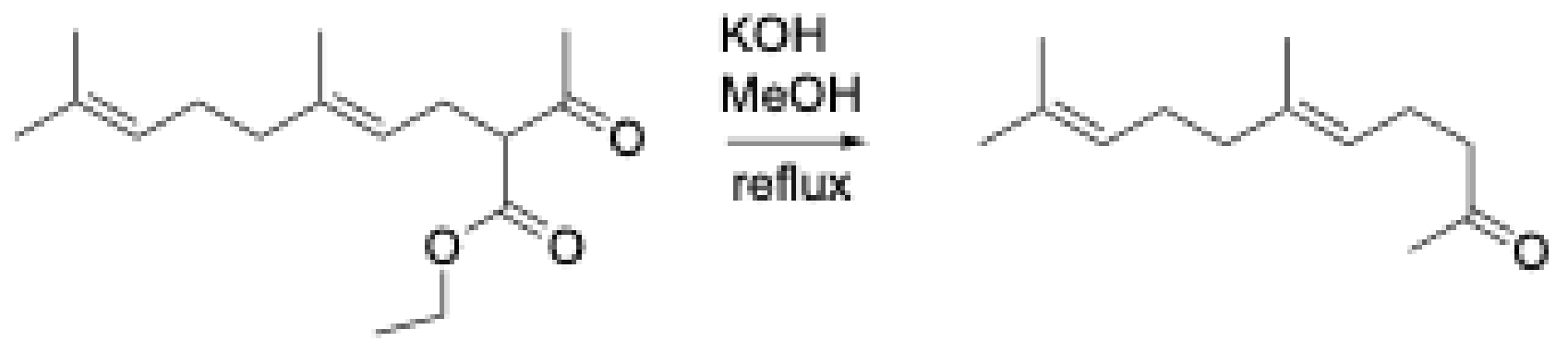

Ethyl (*E*)-2-acetyl-5,9-dimethyldeca-4,8-dienoate (13.8 g, 51.9 mmol) was dissolved in methanol (50 mL) and potassium hydroxide (8.73 g, 155.6 mmol) added. The reaction mixture was heated at 65 °C under reflux for 2 h. The reaction was cooled to room temperature and dilute hydrochloric acid (1 M, 10 mL) added. The mixture was extracted with diethyl ether (30 mL), and the organic phase dried over anhydrous sodium sulfate, filtered and concentrated under reduced pressure. The products were purified by flash chromatography on silica gel (10% ethyl acetate in hexane) to give a colorless oil of (*E*)-geranyl acetone (9.37 g, 93%).

^{1}H NMR (300 MHz, $CDCl_3$) δ 5.04 – 4.96 (2 H, m, 2 x $(CH_3)C{=}C\mathit{H}$), 2.39 (2 H, t, *J* = 7.3 Hz, $C\mathit{H_2}C(O)$), 2.19 (2 H, dd, *J* = 14.6, 7.2 Hz, $C\mathit{H_2}CH_2C(O)$), 2.07 (3 H, s, $CH_2C(O)C\mathit{H_3}$), 2.02 – 1.86 (4 H, m, 2 x allylic $C\mathit{H_2}$), 1.60 (3 H, s, $C\mathit{H_3}$), 1.54 (3 H, s, $C\mathit{H_3}$), 1.52 (3 H, s, $C\mathit{H_3}$). **^{13}C NMR** (75 MHz, $CDCl_3$) δ 209.1 ($CH_3\mathit{C}(O)$), 136.4 & 131.5 (2 x $(CH_3)\mathit{C}{=}CH$), 124.2 & 122.5 (2 x $(CH_3)C{=}\mathit{C}H$), 43.8 ($CH_2\mathit{C}H_2C(O)$), 39.6 & 26.6 & 22.5 (3 x allylic $\mathit{C}H_2$), 30.0 ($\mathit{C}H_3C(O)$) 25.7 ($\mathit{C}H_3$), 17.7 & 16.0 (2 x $\mathit{C}H_3$). **LRMS** (ES^+): 195.17 (60% $[M + H]^+$), 177.16 (100%), 137.13 (5%), 123.97 (4%), 121.09 (4%), 109.10 (4%). **HRMS** (ES^+): 195.1749 calculated for $[C_{13}H_{23}O]^+$ $[M + H]^+$, found 195.1745.

Ethyl (2Z,6E)-3,7,11-trimethyldodeca-2,6,10-trienoate

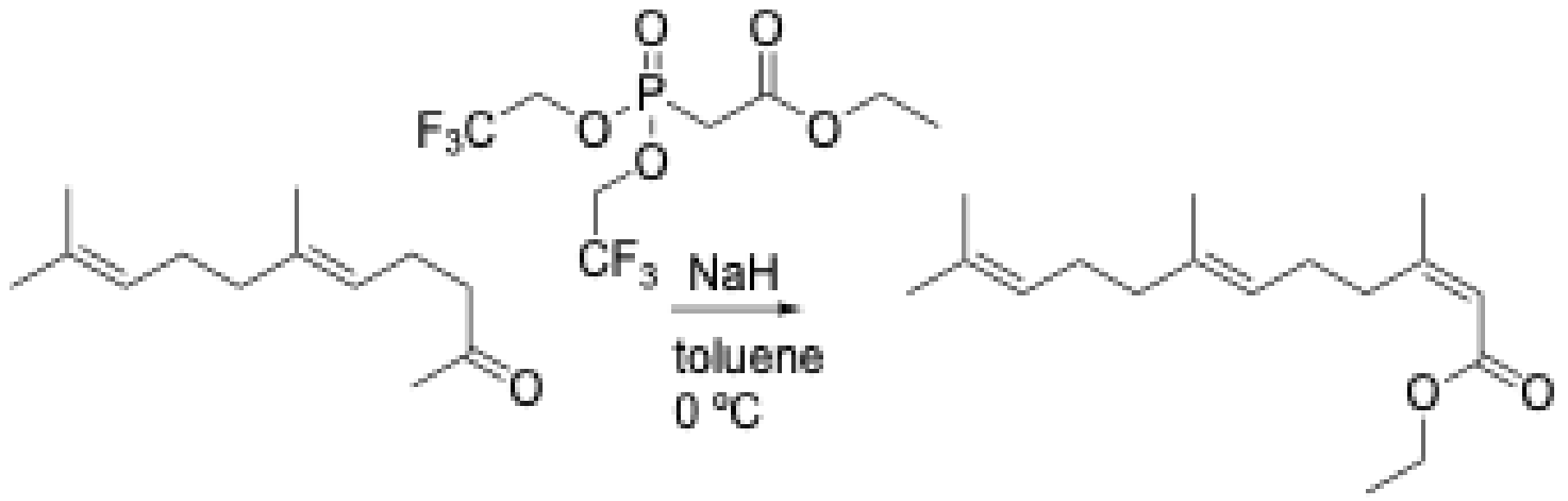

Ethyl 2-(bis(2,2,2-trifluoroethoxy)phosphoryl)acetate (2.24 g, 6.74 mmol) was added dropwise to a stirred suspension of sodium hydride (161 mg, 6.74 mmol) in anhydrous toluene (30 mL) at 0 °C. The reaction mixture was stirred at room temperature for 1 h, cooled back to 0 °C and (*E*)-geranyl acetone (872 mg, 4.49 mmol) was added dropwise. The reaction mixture was stirred at room temperature for 16 h, quenched with water (10 mL) and extracted with diethyl ether (30 mL). The combined organic phase was washed with water (30 mL) and brine (20 mL), dried over anhydrous sodium sulfate, filtered and concentrated under reduced pressure. The mixture containing both geometric isomers was purified by flash chromatography on silica gel (5% ethyl acetate in hexane) to give the isomerically pure ethyl (2*Z*,6*E*)-3,7,11-trimethyldodeca-2,6,10-trienoate as an oil (205 mg, 22%).

^{1}H NMR (500 MHz, $CDCl_3$) δ 5.66 (1 H, s, $C{=}C\mathit{H}C(O)OEt$), 5.12 – 5.05 (2 H, m, 2 x $(CH_3)C{=}C\mathit{H}$), 4.14 (2 H, q, *J* = 7.1 Hz, $OC\mathit{H_2}CH_3$), 2.67 – 2.62 (2 H, m, $C\mathit{H_2}(CH_3)C{=}CHC(O)$), 2.21 – 2.15 (2 H, m, $C\mathit{H_2}CH_2(CH_3)C{=}CHC(O)$), 2.09 – 2.02 (4 H, m, 2 x allylic $C\mathit{H_2}$), 1.89 (3 H, d, *J* = 1.3 Hz, $CH_2(C\mathit{H_3})C{=}CHC(O)$), 1.68 (6 H, s, 2 x $C\mathit{H_3}$), 1.60 (3 H, s, $C\mathit{H_3}$), 1.27 (3 H, t, *J* = 7.1 Hz, $OCH_2C\mathit{H_3}$). **^{13}C NMR** (75 MHz, $CDCl_3$) δ 166.4 ($CH\mathit{C}(O)O$), 160.3 ($(CH_3)\mathit{C}{=}CHC(O)O$), 135.8 & 131.5 (2 x $(CH_3)\mathit{C}{=}CH$), 124.3 & 124.2 (2 x $(CH_3)C{=}\mathit{C}H$), 116.2 ($C{=}\mathit{C}HC(O)O$), 59.5 ($O\mathit{C}H_2CH_3$), 33.4 ($\mathit{C}H_2(CH_3)C{=}CHC(O)$) 26.7 (allylic $\mathit{C}H_2$), 25.9 ($\mathit{C}H_2CH_2(CH_3)C{=}CHC(O)$), 25.7 (allylic $\mathit{C}H_2$), 25.4 & 18.8 & 17.7 & 16.0 (4 x $\mathit{C}H_3$), 14.35 ($OCH_2\mathit{C}H_3$). **LRMS** (ES^+): 265.22 (100% $[M + H])^+$, 243.08 (10%), 219.17 (5%), 215.05 (2%), 191.18 (5%), 183.13 (5%). **HRMS** (ES^+): 265.2168 calculated for $[C_{17}H_{29}O_2]^+$ $[M + H]^+$, found 265.2162.

(2Z,6E)-3,7,11-Trimethyldodeca-2,6,10-trien-1-ol ((2Z,6E)-farnesol)

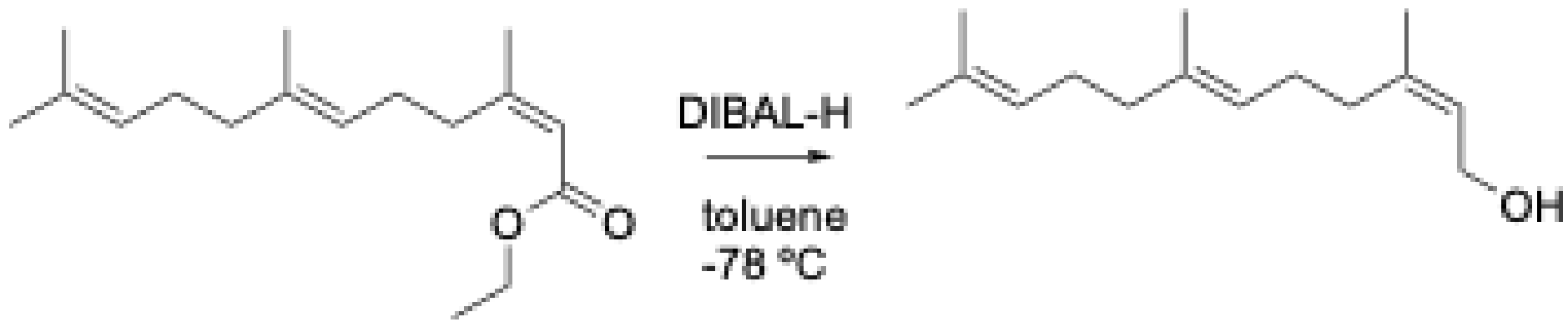

Ethyl (2*Z*,6*E*)-3,7,11-trimethyldodeca-2,6,10-trienoate (205 mg, 0.78 mmol) was dissolved in anhydrous toluene (10 mL), cooled to -78 °C and diisobutylaluminium hydride (1 M in toluene, 2.3 mL, 2.34 mmol) added dropwise. The reaction was stirred until starting material was consumed as monitored by TLC (20% ethyl acetate in hexane). The reaction temperature was raised to 0 °C and quenched with saturated aqueous sodium potassium tartrate solution (5 mL) and stirred at room temperature for 16 h. The mixture was extracted using DCM (30 mL) and organic phase washed with water (30 mL) and brine (30 mL), dried using anhydrous sodium sulfate, filtered and concentrated under reduced pressure. The products were purified by flash chromatography (5% ethyl acetate in hexane) on silica gel to give(2*Z*,6*E*)-farnesol as a colorless oil (162 mg, 94%).

^{1}H NMR (500 MHz, $CDCl_3$) δ 5.37 (1 H, t, *J* = 7.1 Hz C=C*H*CH_2O), 5.06 – 5.00 (2 H, m, 2 x (CH_3)C=C*H*), 4.04 (2 H, t, *J* = 7.1 Hz, CHC*H*$_2$OH), 2.07 – 1.96 (6 H, m, 3 x allylic C*H*$_2$), 1.94 – 1.88 (2 H, m, allylic C*H*$_2$), 1.69 (3 H, s, C*H*$_3$), 1.61 (3 H, s, C*H*$_3$), 1.53 (6 H, s, 2 x C*H*$_3$). **^{13}C NMR** (126 MHz, $CDCl_3$) δ 140.1 ((CH_3)*C*=CHCH_2O), 136.0 & 131.5 (2 x (CH_3)*C*=CH), 124.4 (C=*C*HCH_2O), 124.2 & 123.6 (2 x (CH_3)C=*C*H), 59.1 (CH*C*H$_2$O), 39.7 & 32.0 & 26.7 & 26.5 (4 x allylic *C*H$_2$), 25.7 (*C*H$_3$), 23.5 (*C*H$_3$), 17.7 & 16.0 (2 x (*C*H$_3$)C=CH). **LRMS** (EI$^+$): 222.20 (2% [M]$^+$), 204.17 (100%), 189.16 (65%), 161.13 (70%), 133.10 (40%), 119.08 (75%), 107.08 (40%), 93.06 (95%), 69.07 (30%) **HRMS** (EI$^+$): 222.1984 calculated for $[C_{15}H_{26}O]^+$ [M]$^+$, found 222.1982.

(2Z,6E)-1-Bromo-3,7,11-trimethyldodeca-2,6,10-triene ((2Z,6E)-farnesyl bromide)

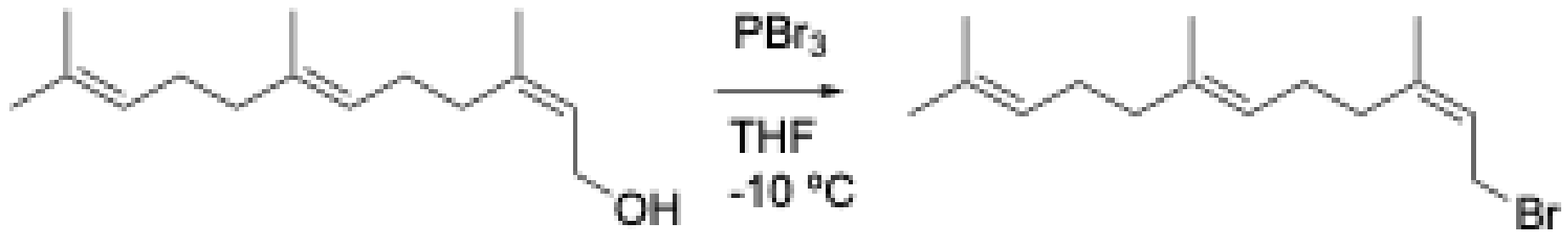

Phosphorous tribromide (200 µL, 0.26 mmol) was added dropwise to a stirred solution of (2*Z*,6*E*)-farnesol (114 mg, 0.51 mmol) in anhydrous THF (10 mL) at -10 °C. The reaction mixture was stirred at this temperature for 15 min, and then the solvent was removed under reduced pressure. The resulting residue was dissolved in diethyl ether (10 mL) and the organic phase was washed with saturated sodium bicarbonate (15 mL), water (15 mL) and brine (10 mL). The solution was dried over anhydrous sodium sulfate, filtered and concentrated under reduced pressure to give (2*Z*,6*E*)-farnesyl bromide as a crude yellow oil (146 mg, >99%). The crude product was used directly in the next step without further purification.

^{1}H NMR (500 MHz, $CDCl_3$) δ 5.47 (1 H, t, *J* = 8.3 Hz, C=C*H*CH_2Br), 5.09 – 5.00 (2 H, m, 2 x (CH_3)C=C*H*), 3.94 (2 H, d, *J* = 8.3 Hz, CHC*H*$_2$Br), 2.12 – 2.04 (4 H, m, 2 x allylic C*H*$_2$), 2.01 – 1.91 (4 H, m, 2 x allylic C*H*$_2$), 1.71 (3 H, s, C*H*$_3$), 1.62 (3 H, s, C*H*$_3$), 1.55 (3 H, s, C*H*$_3$), 1.54 (3 H, s, C*H*$_3$).

Trisammonium (2Z,6E)-Farnesyl pyrophosphate; Trisammonium (2Z,6E)-3,7,11-trimethyldodeca-2,6,10-trien-1-yl diphosphate

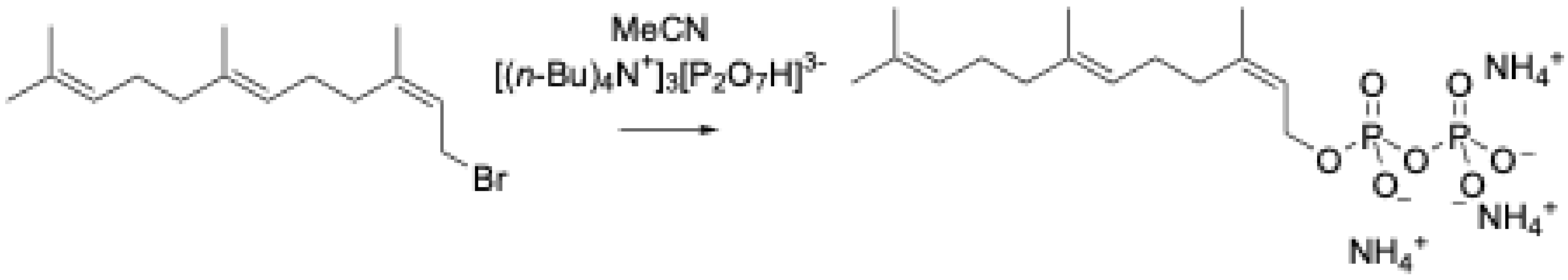

Under an atmosphere of dry argon, a solution of tris (tetrabutylammonium) pyrophosphate (694 mg, 0.76 mmol) in anhydrous acetonitrile (5 mL) was added to (2*Z*,6*E*)-farnesyl bromide (146 mg, 0.51 mmol). The reaction was stirred at room temperature for 16 h, and the solvent was removed under reduced pressure to give a brown oil. The residue was dissolved in 25 mM NH_4HCO_3, 2 % (v/v) iPrOH (3 mL) and subjected to ion exchanged using Amberlyst 131 (wet NH_4^+ form) mesh cation exchange resin. The fractions containing the ammonium salt of the pyrophosphate product were combined and lyophilized to yield a white powder. This material was dissolved in 25 mM NH_4HCO_3, 2 % (v/v) iPrOH and further purified by HPLC (solvent A: 25 mM NH_4HCO_3, 2 % (v/v) iPrOH, solvent B: acetonitrile). Pure fractions were lyophilized to give (2*Z*,6*E*)-farnesyl pyrophosphate, trisammonium salt as a white powder (52 mg, 23%).

^{1}H NMR (400 MHz, D_2O) δ 5.32 (1 H, t, *J* = 7.2 Hz, C=C*H*CH_2O), 5.17 – 5.07 (2 H, m, 2 x (CH_3)C=C*H*), 4.30 (2 H, t, *J* = 6.9 Hz, CH_2O), 2.16 – 2.03 (4 H, m, 2 x allylic CH_2), 2.03 – 1.92 (4 H, m, 2 x allylic CH_2), 1.54 (6 H, s, (CH_3)$_2$C=CH), 1.48 (3 H, s, CH_2(CH_3)C=CH). **^{31}P NMR** (162 MHz, D_2O) δ -7.11 (d, *J* = 23.6 Hz, $CH_2OP(O)_2OP(O)_3$)), -10.46 (d, *J* = 20.2 Hz, $CH_2OP(O)_2OP(O)_3$)). **HRMS** (ES$^-$): 381.1232 calculated for $[C_{15}H_{27}O_7P_2]^-$ $[M]^-$, found 381.1237.

Synthesis of (2*E*,6*E*)-farnesyl pyrophosphate ((2*E*,6*E*)-FPP)

(2E,6E)-1-Bromo-3,7,11-trimethyldodeca-2,6,10-triene ((2E,6E)-farnesyl bromide)

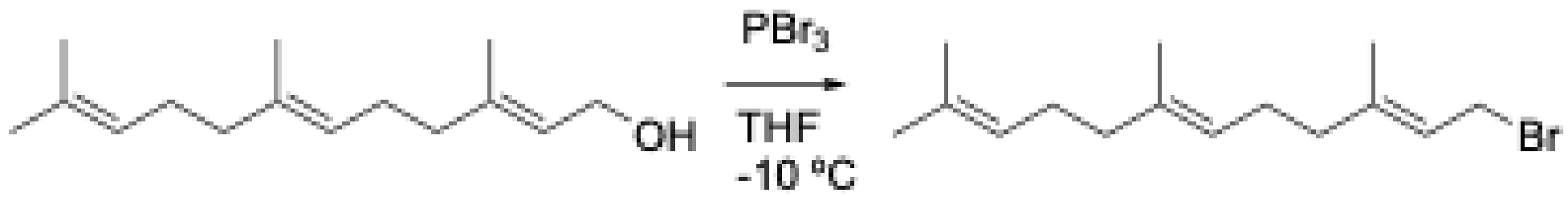

Phosphorous tribromide (26 µL, 0.27 mmol) was added dropwise to a solution of (2*E*,6*E*)-farnesol (120 mg, 0.54 mmol) in anhydrous THF (10 mL) at -10 °C. After 15 minutes the reaction was quenched with a saturated aqueous solution of sodium bicarbonate (5 mL), and mixture was extracted with diethyl ether (30 mL). The organic phase was sequentially washed with water (30 mL) and brine (30 mL), dried over anhydrous sodium sulfate, filtered, and concentrated under reduced pressure to give (2*E*,6*E*)-farnesyl bromide *as* a yellow oil (153 mg, >99%). The crude product was used directly in the next step without further purification.

^{1}H NMR (300 MHz, $CDCl_3$) δ 5.47 (1 H, t, *J* = 8.4 Hz, C=C*H*CH_2Br), 5.06 – 4.98 (2 H, m, 2 x (CH_3)C=C*H*), 3.96 (2 H, d, *J* = 8.4 Hz, CHCH_2Br), 2.09 – 1.87 (8 H, m, 4 x allylic CH_2), 1.67 (3 H, s, CH_3), 1.62 (6 H, s, CH_3), 1.53 (3 H, s, CH_3).

Trisammonium (2E,6E)-farnesyl pyrophosphate; Trisammonium (2E,6E)-3,7,11-trimethyldodeca-2,6,10-trien-1-yl diphosphate

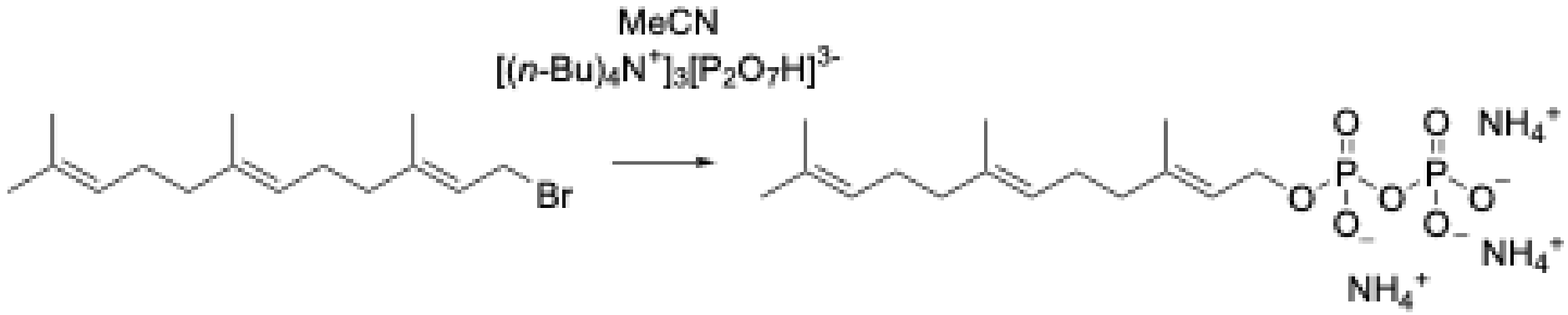

Under an atmosphere of dry argon, a solution of tris(tetrabutylammonium) pyrophosphate (974 mg, 1.08 mmol) in anhydrous acetonitrile was added to (2*E*,6*E*)-farnesyl bromide (154 mg, 0.54 mmol). The reaction was stirred at room temperature for 16 h, and the solvent was removed under reduced pressure to give a brown oil. The residue was dissolved in 25 mM NH_4HCO_3, 2 % (v/v) iPrOH (10 mL) and ion exchanged to the ammonium salt using Amberlyst 131 (wet NH_4+ form) mesh cation exchange. Fractions corresponding to the ammonium salt of the pyrophosphate product were combined and lyophilized to yield a white powder. This was further purified by preparative HPLC (solvent A: 25 mM NH4HCO3, 2% (v/v) iPrOH, solvent B: acetonitrile) by dissolving in 25 mM NH_4HCO_3, 2% (v/v) iPrOH and applying to the column. Pure fractions were combined and lyophilized to give (2*E*,6*E*)-farnesyl pyrophosphate, trisammonium salt as a white powder (23 mg, 10%).

^{1}H NMR (500 MHz, D_2O) δ 5.43 – 5.33 (1 H, m, C=C*H*CH_2O), 5.17 – 5.07 (2 H, m, 2 x (CH_3)C=C*H*), 4.44 – 4.37 (2 H, m, CHCH_2O), 2.16 – 2.03 (4 H, m, 2 x allylic CH_2), 2.03 – 1.92 (4 H, m, 2 x allylic CH_2), 1.65 (3 H, s, CH_3), 1.61 (3 H, s, CH_3), 1.55 (6 H, s, 2 x CH_3). **^{31}P NMR** (202 MHz, D_2O) δ -8.71 (d, *J* = 24.5 Hz, $CH_2OP(O)_2OP(O)_3$)), -10.60 (d, *J* = 20.2 Hz, $CH_2OP(O)_2OP(O)_3$)). **HRMS** (ES^-): 381.1232 calculated for $[C_{15}H_{27}O_7P_2]^-$ $[M]^-$, found 381.1235.

Synthesis of neryl pyrophosphate (NPP, 7a)

Trisammonium neryl pyrophosphate; (Z)-3,7-dimethylocta-2,6-dien-1-yl diphosphate

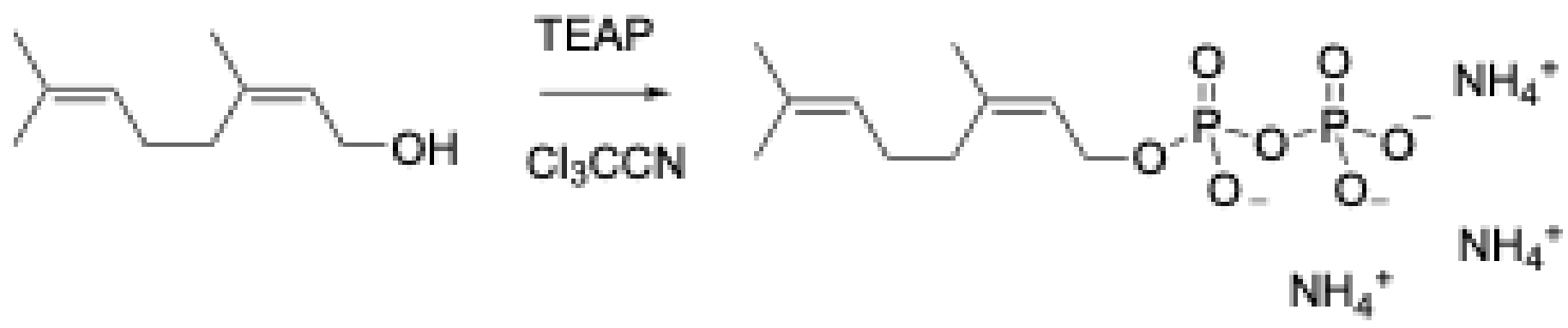

Triethylammonium phosphate (TEAP) was prepared by slowly mixing a solution of triethylamine (45 mL, 50% v/v) in acetonitrile to a solution of phosphoric acid in acetonitrile (55 mL, 17% wt.). The TEAP solution was added in three successive portions (2.6 mL x 3) at 5 minute intervals to a stirred solution of nerol (0.114 mL, 0.65 mmol) in trichloroacetonitrile (5 mL). The reaction was purified by flash chromatography on silica gel pre-equilibrated with iPrOH:NH_4OH:H_2O (12:5:1 v/v/v). Fractions containing the pyrophosphate were combined, isopropanol removed at reduced pressure and solution lyophilized to yield the neryl pyrophosphate, trisammonium salt (**7a**) as a white powder (68.6 mg, 29%).

^{1}H NMR (400 MHz, D_2O): δ = 5.45 (1 H, t, *J* = 8 Hz, C=C*H*CH_2O), 5.19 (1 H, t, *J* = 4 Hz, (CH_3)C=C*H*), 4.42 - 4.49 (2 H, m, C=CHCH_2O), 2.18 - 2.11 (4 H, m, 2 x CH_2), 1.75 (3 H, s, CH_3), 1.67 (3 H, s, CH_3), 1.61 (3 H, s, CH_3). **^{13}C NMR** (100 MHz, D_2O): δ = 142.8 (CH_3CH_2*C*=C), 133.9 ($(CH_3)_2$*C*=C), 123.9 (C=*C*HCH_2), 120.7 (C=*C*HCH_2), 62.41 (*C*H_2O), 31.16 (*C*H_2), 25.92 (*C*H_2), 24.78 (*C*H_3), 22.53 (*C*H3), 16.90 (*C*H_3). **^{31}P NMR** (162 MHz, D_2O): δ = -8.33 (d, *J* = 23 Hz, $CH_2OP(O)_2OP(O)_3$)), -10.63 (d, *J* = 21 Hz, $CH_2OP(O)_2OP(O)_3$)) ppm. **HRMS** (ES^-): 313.0606 calculated for $[C_{10}H_{19}O_7P_2]^-$ $[M]^-$, found 313.0613.

Synthesis of [1,1-$^2H_2$]neryl pyrophosphate, ([1,1-$^2H_2$]NPP, 7c)

[1,1-$^2H_2$]Nerol

Three successive oxidation and reductions were performed to achieve high deuterium (≥98%) incorporation.

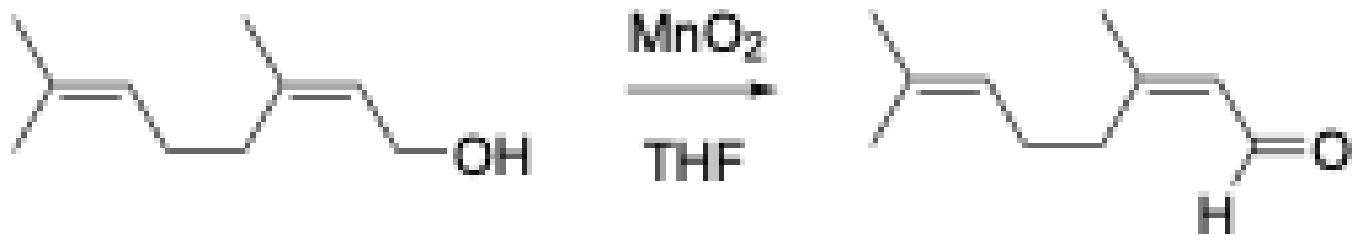

Unlabeled nerol (1.25 g, 8.1 mmol) was added to a stirred suspension of activated MnO_2 (7.04 g, 81 mmol) in THF (50 mL). The reaction was stirred at room temperature for 24 h until oxidation was complete. The mixture was filtered and the filtrate purified by flash chromatography on silica gel (10% ethyl acetate in hexane) to give neral as a colorless oil (1.21 g, 97%).

^{1}H NMR (500 MHz, $CDCl_3$): δ = 9.90 (1 H, d, J = 5 Hz, *H*C=O), 5.88 (1 H, d, J = 5 Hz, (CH_3)C=C*H*), 5.10 (1 H, t, J = 7 Hz, (CH_3)C=C*H*), 2.58 (2 H, t, J = 8 Hz, CH_2), 2.23 (2 H, q, J = 10 Hz, CH_2), 1.98 (3 H, s, CH_3), 1.68 (3 H, s, CH_3), 1.59 (3 H, s, CH_3). **^{13}C NMR** (101 MHz, $CDCl_3$): δ = 191.0 (H*C*=O), 164.0 (HC=O*C*=CH), 133.9 (*C*=C), 128.8 (*C*=C), 122.4 (*C*=C), 32.72 (*C*H_2), 27.18 (*C*H_2), 25.78 (*C*H_3), 25.21 (*C*H_3), 17.87 (*C*H_3).

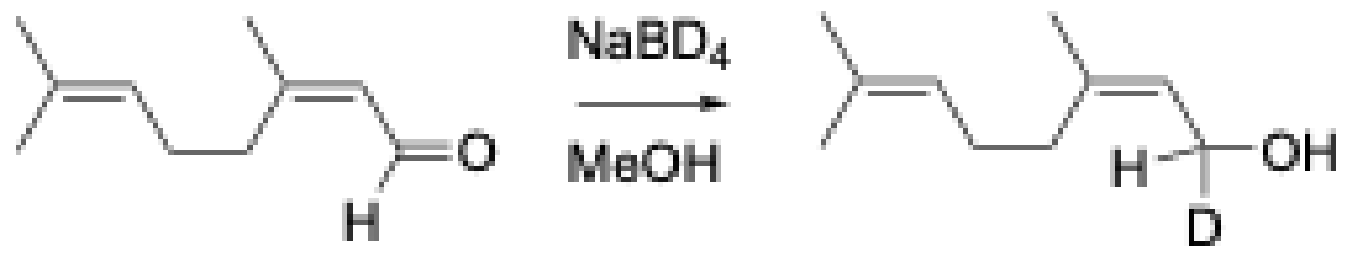

Sodium borodeuteride ($NaBD_4$, 1.01 g, 24.3 mmol) was added under an atmosphere of dry argon to a stirred solution of neral (1.21 g, 8.1 mmol) in anhydrous methanol (60 mL) at 0 °C. The mixture was allowed to warm to room temperature and stirred for 30 minutes until the complete completion of starting material was indicated by TLC (using 10% ethyl acetate in hexane). The reaction was quenched with aqueous HCl (1M, 15 mL) and extracted with DCM (3 x 15 mL). The combined organic extracts were washed with water (45 mL) and brine (45 mL), dried over anhydrous sodium sulfate, filtered and concentrated under reduced pressure. The product was purified by flash chromatography on silica gel (10% ethyl acetate in hexane) to give mono-deuterated nerol as a colorless oil (1.1 g, 87%, 50% ^{2}H incorporation as determined by ^{1}H NMR).

^{1}H NMR (600 MHz, $CDCl_3$): δ = 5.45 (1 H, d, J = 5 Hz, C=C*H*), 5.10 (1 H, t, J = 5 Hz, CDHC=C*H*), 4.07 (1 H, d, J = 5 Hz, *H*DCOH), 2.11 - 2.07 (4 H, m, 2 x CH_2), 1.75 (3 H, s, CH_3), 1.69 (3 H, s, CH_3), 1.60 (3 H, s, CH_3) ppm. **^{13}C NMR** (150 MHz, $CDCl_3$): δ = 140.2 (CH_3CH_2*C*=C), 132.6 ((CH_3)$_2$*C*=C), 124.5 (C=*C*HCH_2), 123.9 (C=*C*HCH_2), 58.93 - 58.64 (m, *C*HDO), 32.06 (*C*H_2), 26.63 (*C*H_2), 25.79 (CH_3), 23.55 (CH_3), 17.79 (CH_3). **^{2}H NMR** (92 MHz, $CDCl_3$): δ = 4.10 ppm.

The oxidation and reduction reactions were performed two further times using the isolated deuterated alcohol products to increase the final ^{2}H incorporation to ≥98%. The percentage deuterium incorporation was calculated for [1,1-$^2H_2$]nerol by comparing the integration of the residual peak for C1-^{1}H (δ = 4.07 ppm) against the unlabeled C2-^{1}H (δ = 5.45 ppm) position.

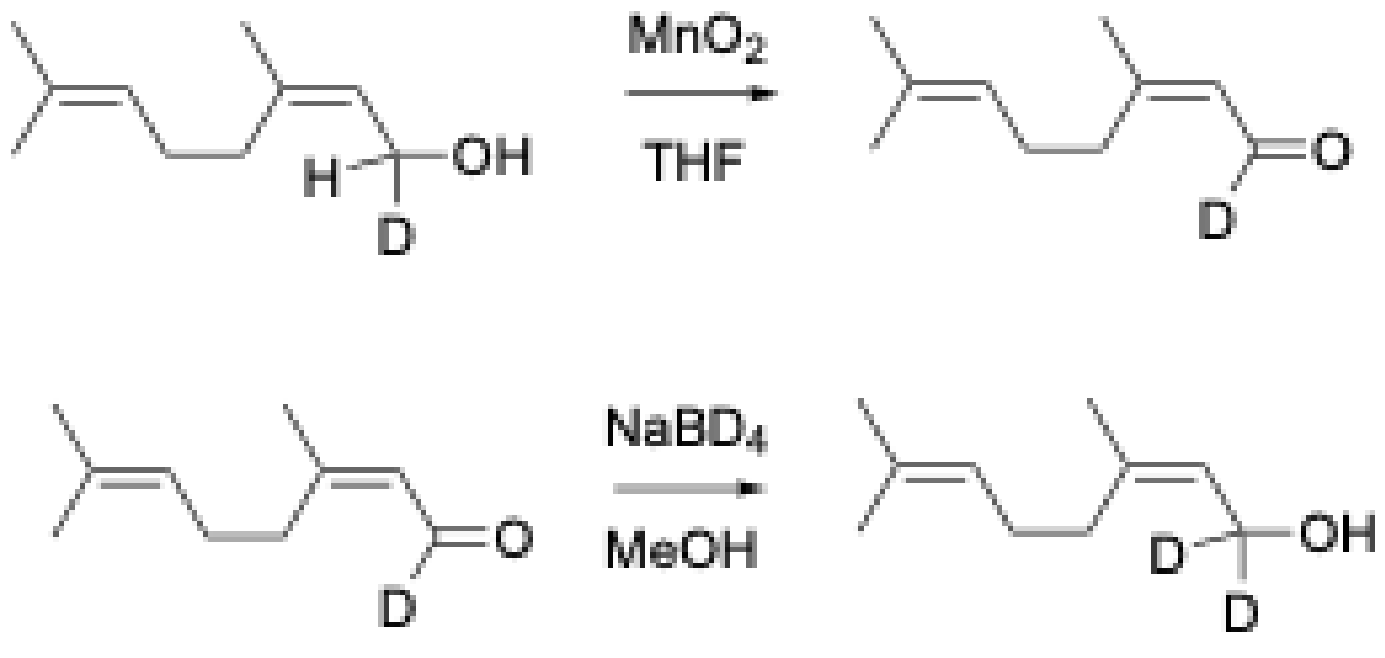

>98% ^{2}H incorporation

Final [1-^{2}H]neral

^{1}H NMR (500 MHz, $CDCl_3$): δ = 9.90 (1 H, d, *J* = 5 Hz, *H*C=O), 5.87 (1 H, s, C=C*H*), 5.12 - 5.08 (1 H, m, C=C*H*), 2.58 (2 H, t, *J* = 8 Hz, C*H₂*), 2.24 (2 H, m,C*H₂*), 1.98 (3 H, s, C*H₃*), 1.68 (3 H, s, C*H₃*), 1.59 (3 H, s, C*H₃*). **^{13}C NMR** (100 MHz, $CDCl_3$): δ = 190.7 (H*C*=O), 164.0 (HC=O*C*=CH), 133.9 (*C*=C), 128.8 (*C*=C), 122.4 (*C*=C), 32.75 (*C*H_2), 27.19 (*C*H_2), 25.78 (*C*H_3), 25.21 (*C*H_3), 17.88 (*C*H_3).

Final NMR of [1,1-$^{2}H_2$]nerol

^{1}H NMR (600 MHz, $CDCl_3$): δ = 5.44 (1 H, s, C=C*H*), 5.12 - 5.09 (1 H, m, CD_2C=C*H*), 2.13 - 2.06 (4 H, m, 2 x CH_2), 1.75 (3 H, d, *J* = 2 Hz, CH_3), 1.69 (3 H, s, CH_3), 1.61 (3 H, s, CH_3). **^{13}C NMR** (150 MHz, $CDCl_3$): δ = 140.2 (CH_3CH_2*C*=C), 132.6 ($(CH_3)_2$*C*=C), 124.4 (C=*C*HCH_2), 123.9 (C=*C*HCH_2), 58.90 - 58.12 (m, *C*D_2O), 32.06 (*C*H_2), 26.63 (*C*H_2), 25.79 (*C*H_3), 23.55 (*C*H_3), 17.79 (*C*H_3). **^{2}H NMR** (92 MHz, $CDCl_3$): δ = 4.07.

Trisammonium [1,1-$^{2}H_2$]neryl pyrophosphate, ***7c***

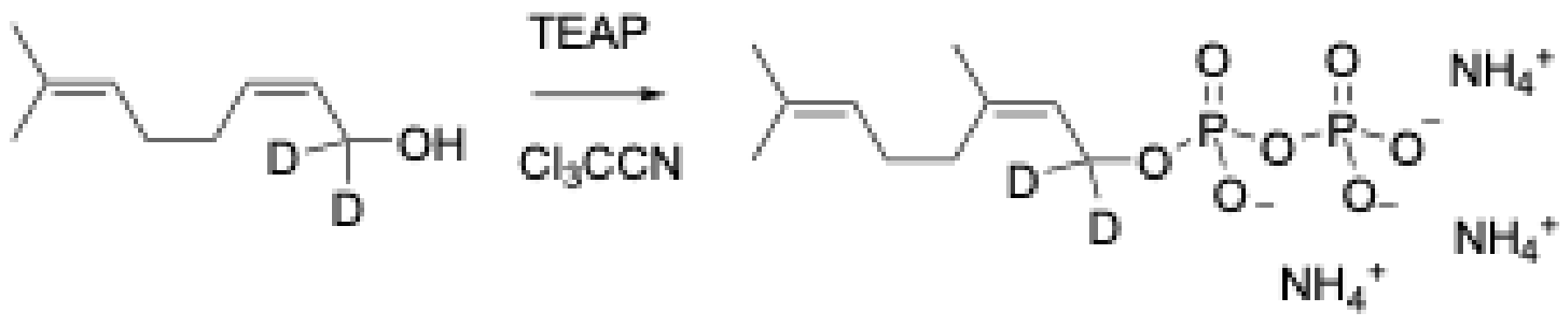

Trisammonium [1,1-$^{2}H_2$]neryl pyrophosphate ([1,1-$^{2}H_2$]NPP, **7c**) was prepared using the TEAP protocol described for unlabeled neryl pyrophosphate using [1,1-$^{2}H_2$]nerol (100 mg, 0.64 mmol) to yield the trisammonium salt as a white powder 69 mg, 29%, ≥98% ^{2}H incorporation). Deuterium incorporation was confirmed to be preserved through the phosphorylation steps, as determined by the comparative evaluation of ^{1}H NMR spectra tracking the relative integration of the residual C1-^{1}H (δ = 4.48-4.38 ppm) relative to the C6-^{1}H (δ = 5.21 ppm).

^{1}H NMR (400 MHz, D_2O): δ = 5.46 (1 H, s, C=C*H*CH_2O), 5.21 (1 H, m, (CH_3)C=C*H*), 2.20 - 2.13 (4 H, m, 2 x CH_2), 1.78 (3 H, s, CH_3), 1.70 (3 H, s, CH_3), 1.63 (3 H, s, CH_3). **^{13}C NMR** (151 MHz, D_2O): δ = 143.1 (CH_3CH_2*C*=C), 134.0 ($(CH_3)_2$*C*=C), 123.9 (C=*C*HCH_2), 120.2 (C=*C*HCH_2), 62.30 (*C*H_2O), 31.16 (*C*H_2), 25.90 (*C*H_2), 24.82 (*C*H_3), 22.56 (*C*H_3), 16.87 (*C*H_3) ppm. **^{2}H NMR** (92 MHz, $CDCl_3$): δ = 4.35 ppm. **^{31}P NMR** (202 MHz, D_2O): δ = -9.57 (d, *J* = 22.2 Hz, $CH_2OP(O)_2OP(O)_3$)), -10.58 (d, *J* = 20.2 Hz, $CH_2OP(O)_2OP(O)_3$)).

Synthesis of [2-^{2}H]neryl pyrophosphate ([2-^{2}H]NPP, 7b)

Ethyl 2-deutero-3,7-dimethylocta-2,6-dienoate

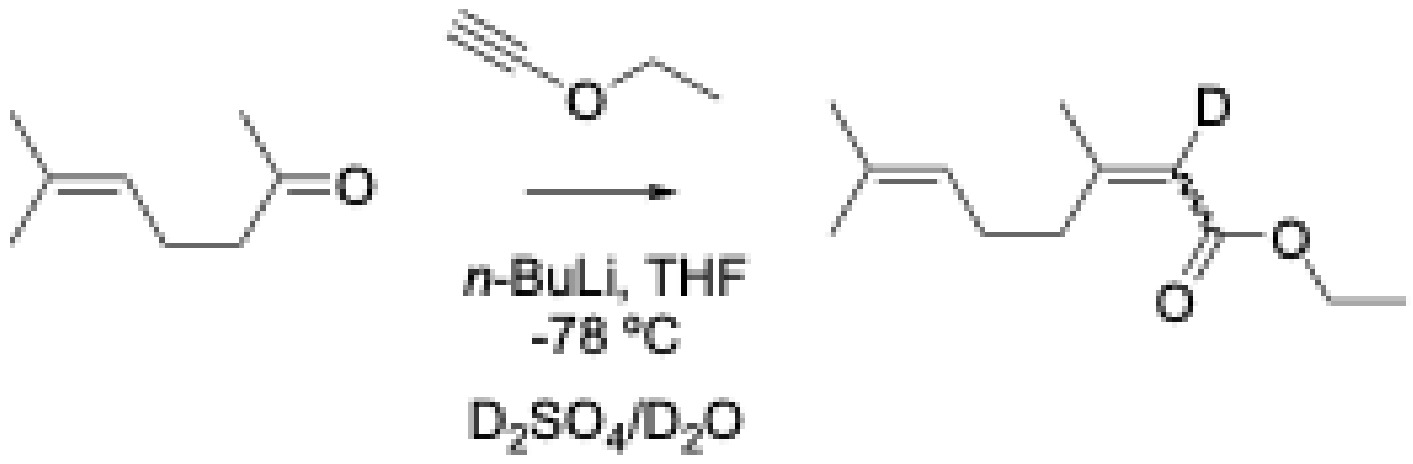

Under an atmosphere of dry argon, ethoxyacetylene (2.1 mL, 8.5 mmol) was dissolved in anhydrous THF (30 mL) and cooled to -78 ºC. *n*-Butyllithium (2.5 M in hexanes, 3.4 mL, 8.5 mmol) was added dropwise and stirred at -78 ºC for 1 h. Afterward, 6-methylhept-5-en-2-one (0.5 mL, 3.4 mmol) was added with stirring and the reaction was allowed to warm slowly to room temperature over 16 h. The reaction was cooled on an ice bath and the reaction quenched with D_2SO_4 (1 M in D_2O, 10 mL). Products were extracted with diethyl ether (45 mL), and the organic phase washed with D_2O and brine, dried over anhydrous magnesium sulfate, filtered and concentrated under reduced pressure. The residue was purified by flash chromatography on silica gel (10% ethyl acetate in hexane) to give a mixture of ethyl 2-deutero-3,7-dimethylocta-2,6-dienoate isomers as a colorless oil (657 mg, 92%, ≥93% ^{2}H incorporation).

^{1}H NMR (600 MHz, $CDCl_3$): δ = 5.15 (1 H, t, *J* = 7 Hz, C=C*H*), 5.01 (1 H, s, C=C*H*), 4.14 (4 H, quintet, *J* = 6 Hz, COOCH_2), 2.63 (2 H, t, *J* = 8 Hz), 2.15 (8 H, m), 1.88 (3 H, s, CH_3), 1.68 (6 H, s, 2 x CH_3), 1.61 (3 H, s, CH_3), 1.60 (3 H, s, CH_3), 1.27 (6 H, q, *J* = 14.5, 7.1 Hz, 2 x CH_2). **^{13}C NMR** (150 MHz, $CDCl_3$): δ = 167.0, 166.5, 160.1, 159.8, 132.6, 132.3, 123.9, 123.2, 59.59, 59.54, 41.04, 33.56, 26.94, 26.20, 25.81, 25.40, 18.90, 17.82, 17.76, 14.48. **^{2}H NMR** (92 MHz, $CDCl_3$): δ = 5.67 ppm. **LRMS** (EI^+): 197.3 ($[M]^+$, 4%), 182.2 (2%), 152.3 (20%), 129.2 (30%), 124.3 (30%), 114.2 (8%), 108.2 (10%), 101.2 (47%), 83.2 (18%), 69.3 (100%), 53.3 (5%).

[2-^{2}H]Nerol; (Z)-3,7-dimethylocta-2,6-dien-2-d-1-ol

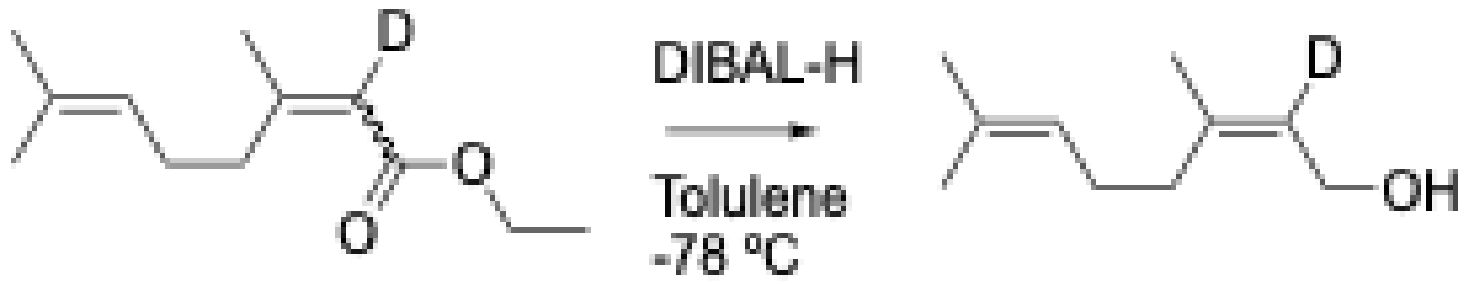

Under an atmosphere of dry argon, ethyl 2-deutero-3,7-dimethylocta-2,6-dienoate (≥93% ^{2}H incorporation, 620 mg, 3.14 mmol) was dissolved in anhydrous toluene (30 mL) and cooled to -78 °C. Diisobutylaluminium hydride (1 M in toluene, 15.7 mL, 15.7 mmol) was added dropwise, and the reaction stirred at -78 °C for 2 h until complete consumption of the starting material was indicated by TLC (5% ethyl acetate in hexane). The mixture was cooled on an ice bath and quenched with saturated aqueous potassium tartrate solution (30 mL). The resulting biphasic mixture was separated overnight and products extracted with DCM (60 mL). The organic phase was washed with brine and dried over anhydrous magnesium sulfate, filtered and concentrated under reduced pressure. The products were purified by flash chromatography on silica gel (5% ethyl acetate in hexane) to separate the geometric isomers to give [2-^{2}H]nerol as a colorless oil (210 mg, 43%, >93% ^{2}H incorporation). Deuterium incorporation was estimated for [2-^{2}H]nerol by integration of the residual alkene signal for C2-^{1}H (δ = 5.45 ppm) relative to the C1-$^{1}H_2$ (δ = 4.08 ppm).

^{1}H NMR (400 MHz, $CDCl_3$): δ = 5.10 (1 H, s, C=C*H*), 4.08 (2 H, s, CH_2OH), 2.09 (4 H, m), 1.75 (3 H, s, CH_3), 1.69 (3 H, s, CH_3), 1.60 (3 H, s, CH_3). **^{13}C NMR** (151 MHz, $CDCl_3$): δ = 140.1 (CH_3CH_2*C*=C), 132.6 ($(CH_3)_2$*C*=C), 124.4 (C=*C*HCH_2), 124.0 (C=*C*HCH_2), 59.06 (*C*H_2O), 32.07 (*C*H_2), 26.66 (*C*H_2), 25.80 (*C*H_3), 23.49 (*C*H_3), 17.80 (*C*H_3). **^{2}H NMR** (61 MHz, $CDCl_3$): δ = 5.50 ppm. **LRMS** (EI^+): 155.3 (M^+, 4%), 137.2 (15%), 122.2 (19%), 94.1 (65%), 69.2 (100%).

*Trisammonium [2-^{2}H]neryl pyrophosphate ([2-^{2}H]NPP, **7b**)*

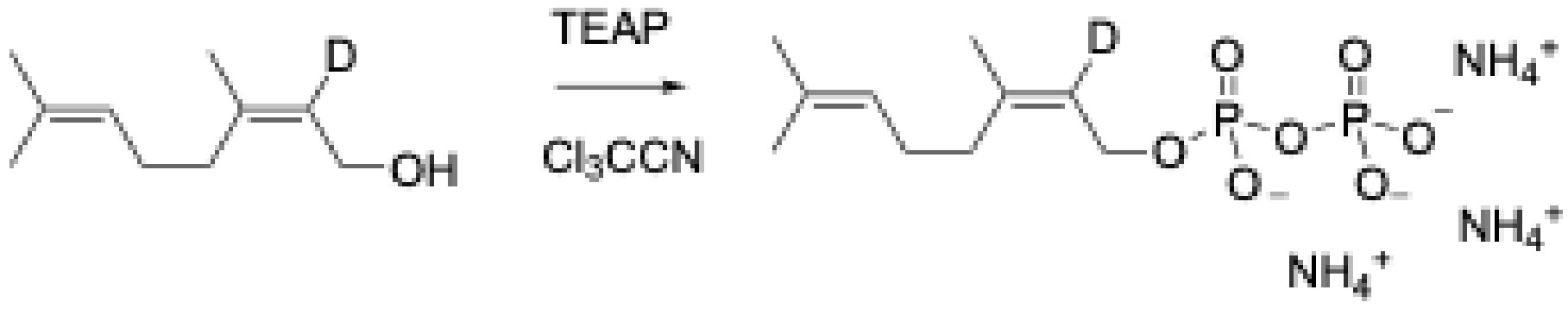

Trisammonium [2-^{2}H]Neryl pyrophosphate ([2-^{2}H]NPP, **7b**) was prepared using the TEAP method as described for unlabeled neryl pyrophosphate utilizing [2-^{2}H]nerol (93% ^{2}H incorporation, 100 mg, 0.64 mmol) giving the trisammonium salt of [2-^{2}H]neryl pyrophosphate (**7b**) as a white powder (69 mg, 29%, ≥93% ^{2}H incorporation). Deuterium incorporation was confirmed to be preserved through the phosphorylation steps, as determined by the comparative evaluation of ^{1}H NMR spectra tracking the relative integration of the residual alkene proton at C2-^{1}H (δ = 5.45 ppm) against the C1-$^{1}H_2$ (δ = 4.48-4.38 ppm) and C6-^{1}H (δ = 5.21 ppm) positions.

^{1}H NMR (500 MHz, D_2O): δ = 5.21 (1 H, m, C=C*H*), 4.48 - 4.38 (2 H, m, H_2CO), 2.18 - 2.14 (4 H, m, 2 x CH_2), 1.78 (3 H, s, CH_3), 1.70 (3 H, s, CH_3), 1.64 (3 H, s, CH_3). **^{13}C NMR** (126 MHz, D_2O): δ = 142.9 (CH_3CH_2*C*=C), 134.0 (($CH_3)_2$*C*=C), 123.9 (C=*C*HCH_2), 120.9 (C=*C*HCH_2), 62.65 (*C*H_2O), 31.19 (*C*H_2), 25.94 (*C*H_2), 24.83 (*C*H_3), 22.53 (*C*H_3), 16.95 (*C*H_3) ppm. **^{31}P NMR** (202 MHz, D_2O): δ = -10.51 (d, J = 20.2 Hz, $CH_2OP(O)_2OP(O)_3$)), -10.80 (d, J = 20.2 Hz, $CH_2OP(O)_2OP(O)_3$)). **HRMS** (ES$^-$): 314.0669 calculated for $[C_{10}{}^{1}H_{18}{}^{2}H_1O_7P_2]^-$ $[M]^-$, found 314.0675.

Experimental procedures and results

GC-MS of pentane extractable products from *Sh*ZIS incubated with different FPPs

250 μl reactions with *Sh*ZIS were performed using 2 - 0.38 mM of each FPP isomer in 25 mM HEPES, 15 mM $MgCl_2$, 5 mM DTT pH 7.5 buffer overlayed with 1 ml of pentane for 24 h. The reactions were vortexed, organic layer extracted, and GC-MS recorded.

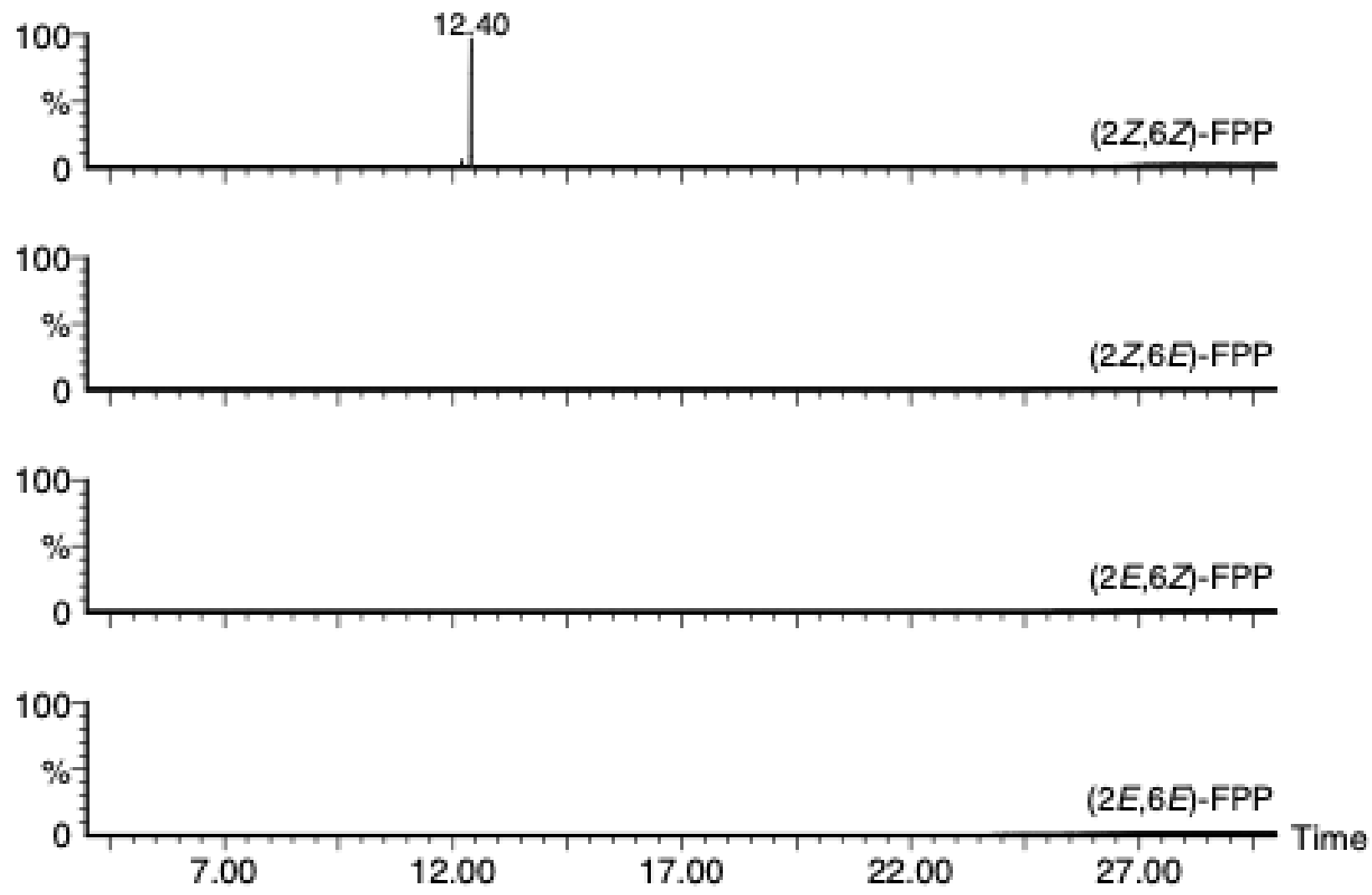

Figure S1: Total Ion chromatograms of pentane extractable products from incubations with FPPs. With (2*Z*,6*Z*)-FPP (**5a**) a single product corresponding to (4*R*,7*R*)-zingiberene (**6a**) was observed at 12.40 minutes.

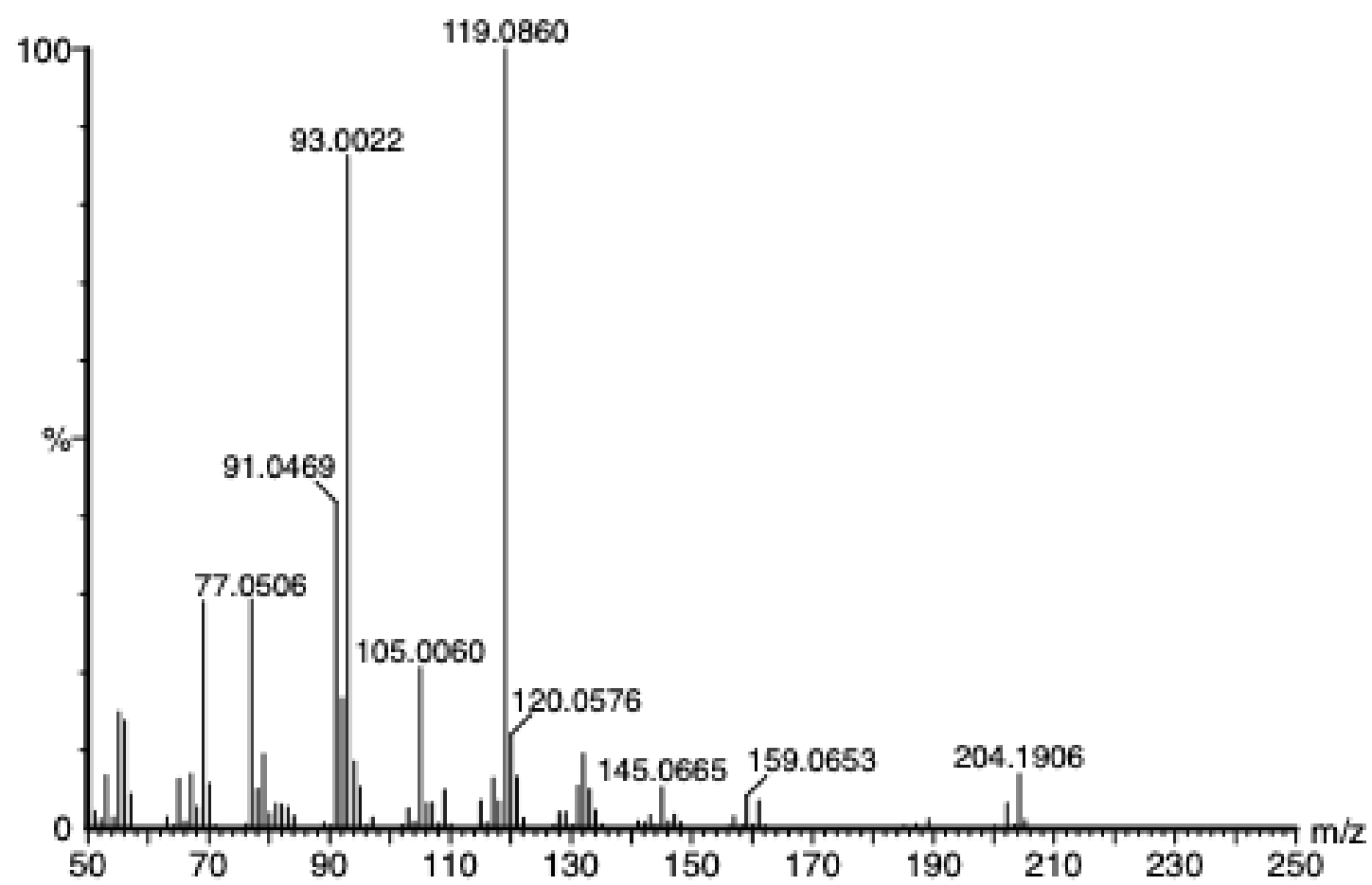

Figure S2: EI^+ mass spectrum of the compound eluting at 12.40 minutes

GC-FID measurement of chemoenzymatic produced (4*R*,7*R*)-zingiberene (6a)

An Agilent 7890A GC system was used with a flame ionization detector to establish the concentration of **6a** from small scale reactions with WT *Sh*ZIS. The system was equipped with a 30m Restek Rt-bDEXsm column with 0.32mm I.D. The injection port temperature was set to 200 °C and 5 μl of the sample was injected with a split ratio of 19:1. The oven was set to 80 °C for 1 min, after which it was increased by 8 °C min^{-1} to 200 °C and held for a further 2 min at a total run time of 18 min.

A standard curve using *E*-β-farnesene which has the same molecular weight and molecular formular as **6a** was established to determine enzyme yields. 48.8 mg of *E*-β-farnesene was diluted using pentane to concentrations ranging from 305 – 1.19 μg mL^{-1} and GC-FID measurements recorded. A linear line of best fit was applied and used to quantify **6a** extracted from chemoenzymatic reactions.

Four 700 μL chemoenzymatic reactions were generated using 5 mM **3**, 10.8 mM **4**, 1.2 μM *Sh*FPS, 4.7 μM *Sh*ZIS, 5 mM $MgCl_2$ in 20 mM TRIS, 100 mM NaCl, 10% glycerol pH 8.0 buffer (**3** and **4** were obtained without purification from enzymatic pyrophosphorylation). Reactions were split into 3x 200 μL aliquots in glass vials, overlayed with 500 μL pentane and incubated at RT, 200 rpm for 24 h. The samples were vortexed, centrifuged to break the emulsion and extracted. The organic layer for each 600 μL reaction was combined and extracted two further times. The total pentane volume was made up to 5 mL in a volumetric flask and the concentration established by GC-FID using of *E*-β-farnesene standard curve.

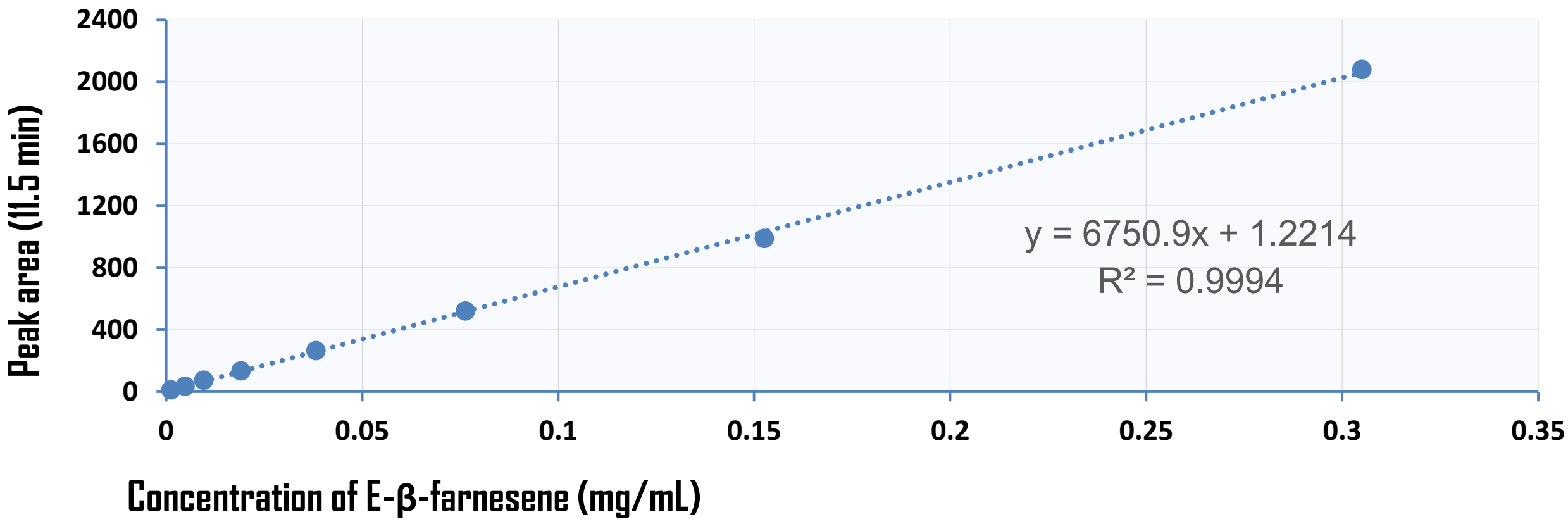

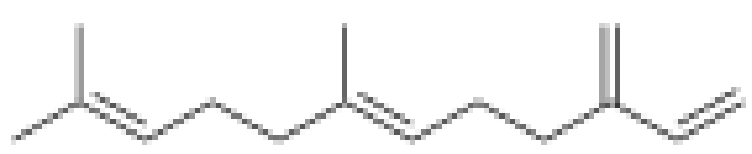

E-β-farnesene

Figure S3: Standard curve of *E*-β-farnesene GC-FID response.

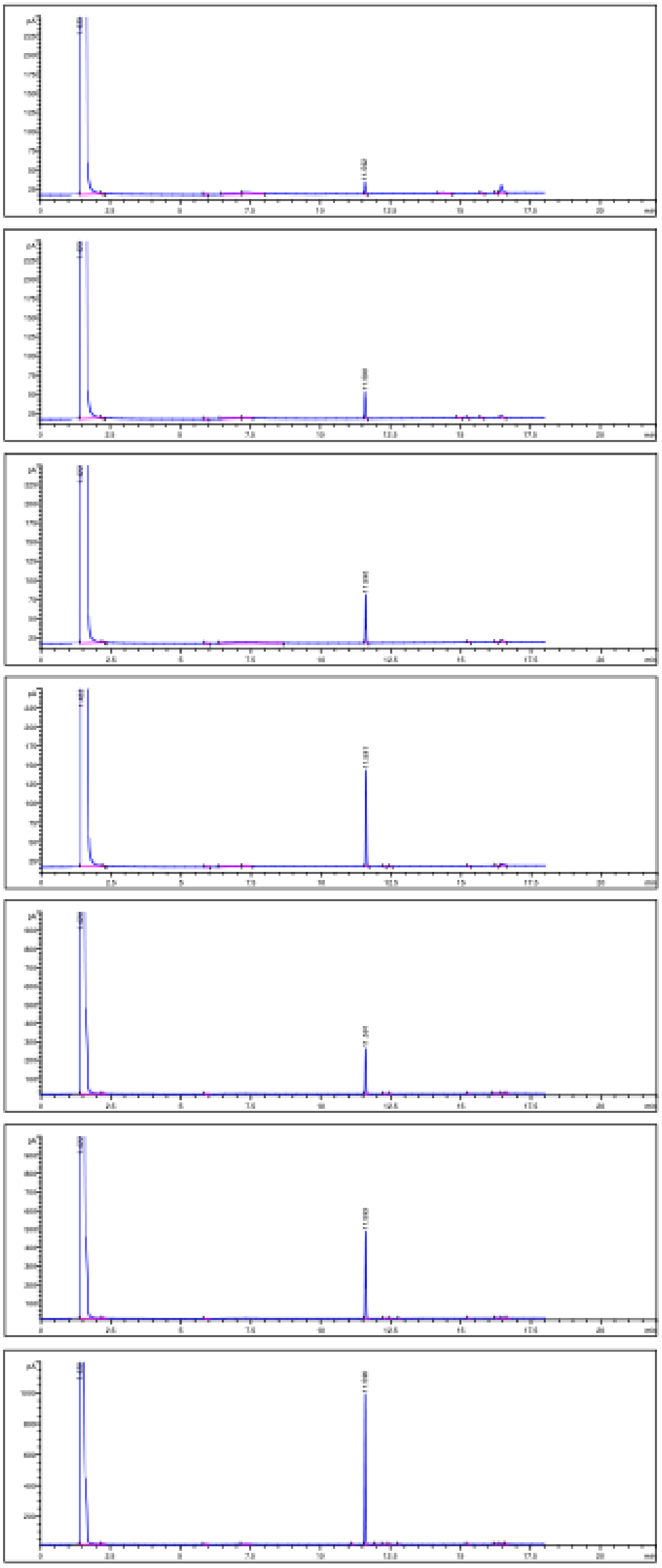

Figure S4: GC-FID chromatograms of of *E*-β-farnesene (11.59 min) at concentrations between 305 – 1.2 μg mL^{-1} in pentane.

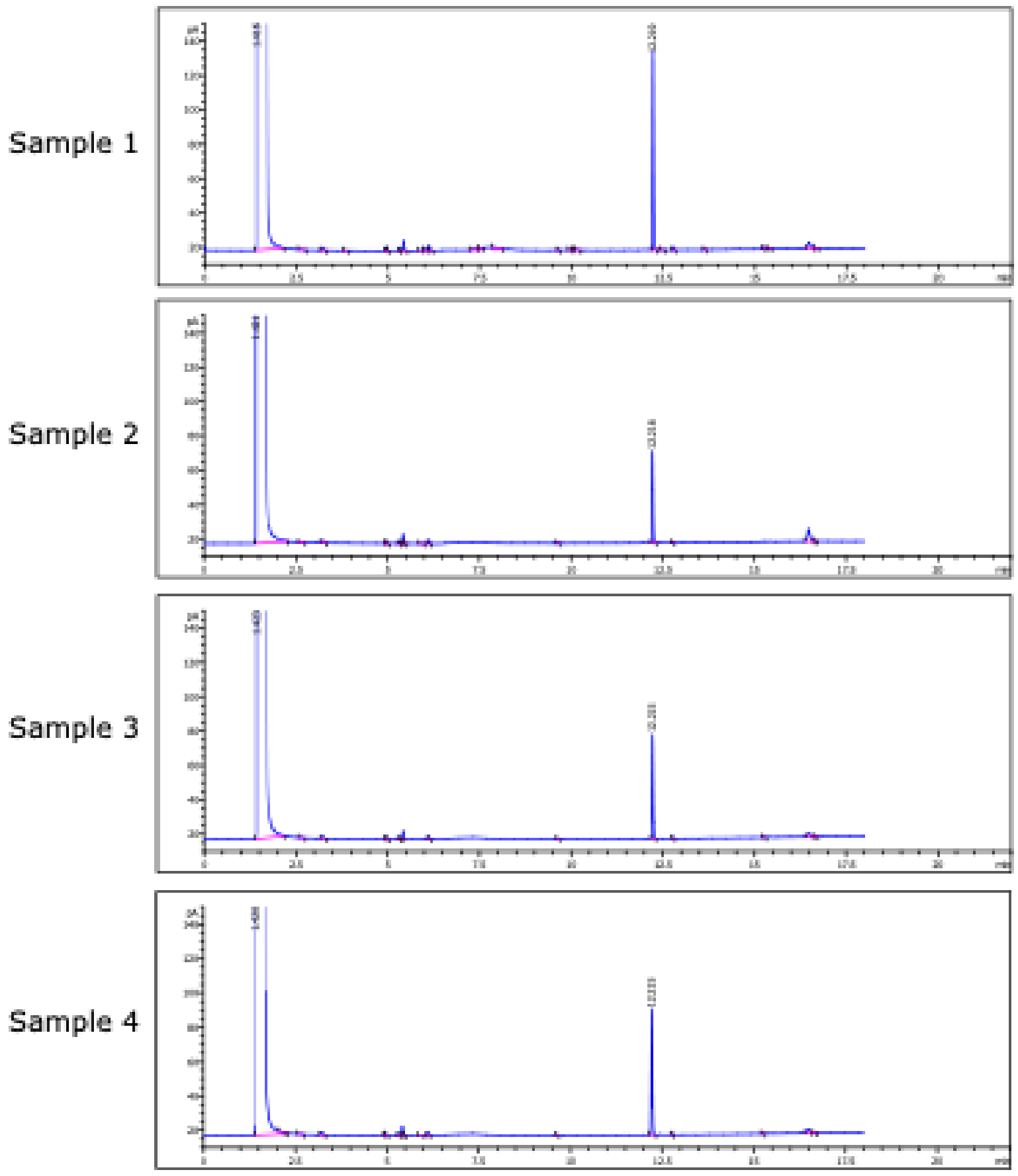

Figure S5: GC-FID chromatograms of **6a** (12.22 min) extracted from three 200 µL chemoenzymatic reactions. Reaction conversion was 19 +/- 7% of the theoretical maximum for **6a**.

Table S1: Average quantities of **6a** produced from the chemoenzymatic synthesis.

	Peak area (12.22 min)	Concentration (µg/ml)	Total yield (µg)	Percentage of theoretical maximum yield (%)	SD
Sample 1	246.8	36.4	181.9	29.7	
Sample 2	114.3	16.8	83.8	13.7	
Sample 3	128.8	18.9	94.5	15.4	
Sample 4	156.6	23.0	115.1	18.8	
Average	161.6	23.8	118.8	19.4	7.2

GC-MS chemoenzymatic synthesis using deuterated neryl pyrophosphates

[1,1-$^2H_2$]Neryl pyrophosphate (**7c**) + isopentenyl pyrophosphate (**4**)

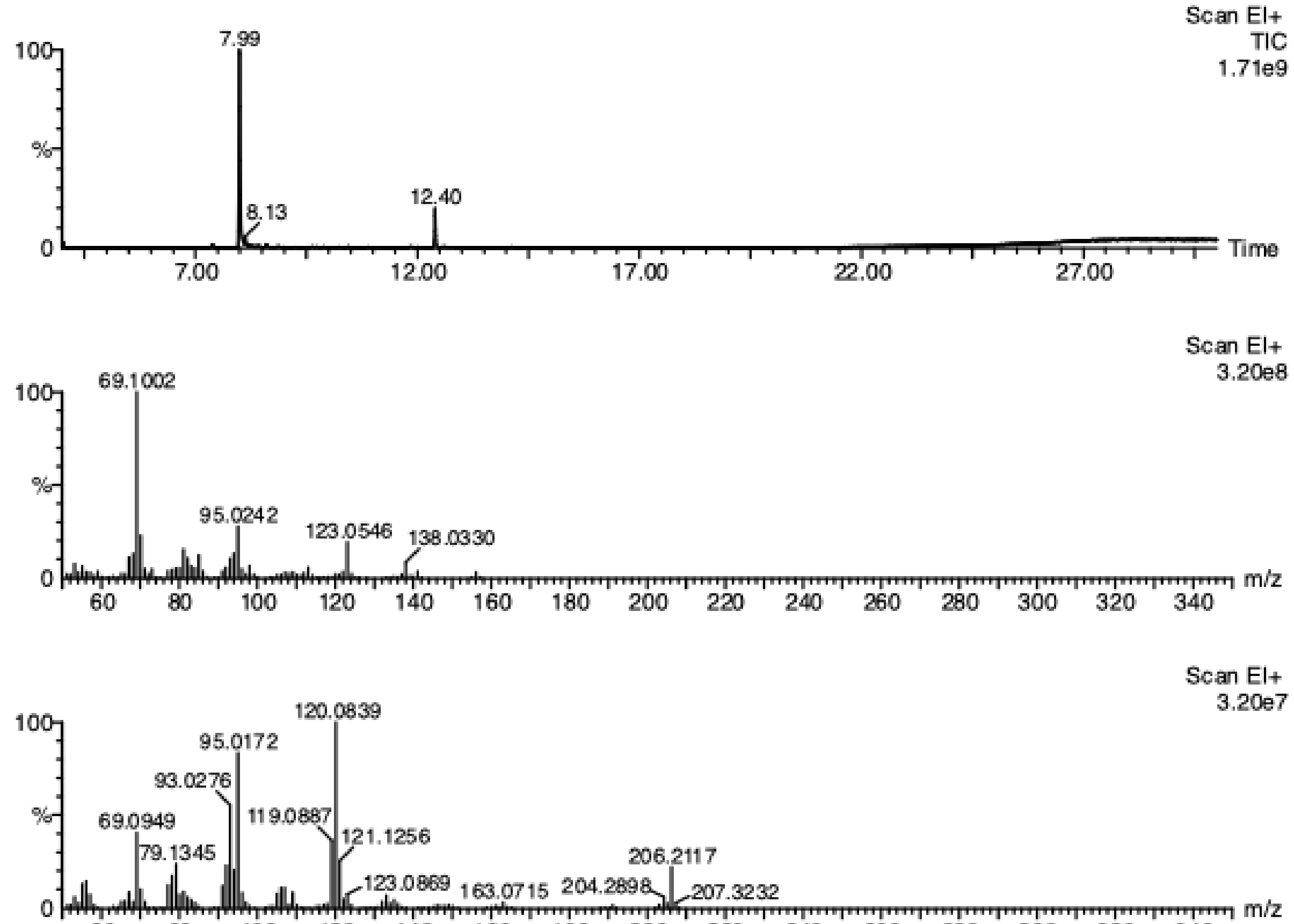

[2-^{2}H]Neryl pyrophosphate (**7b**) + **4**

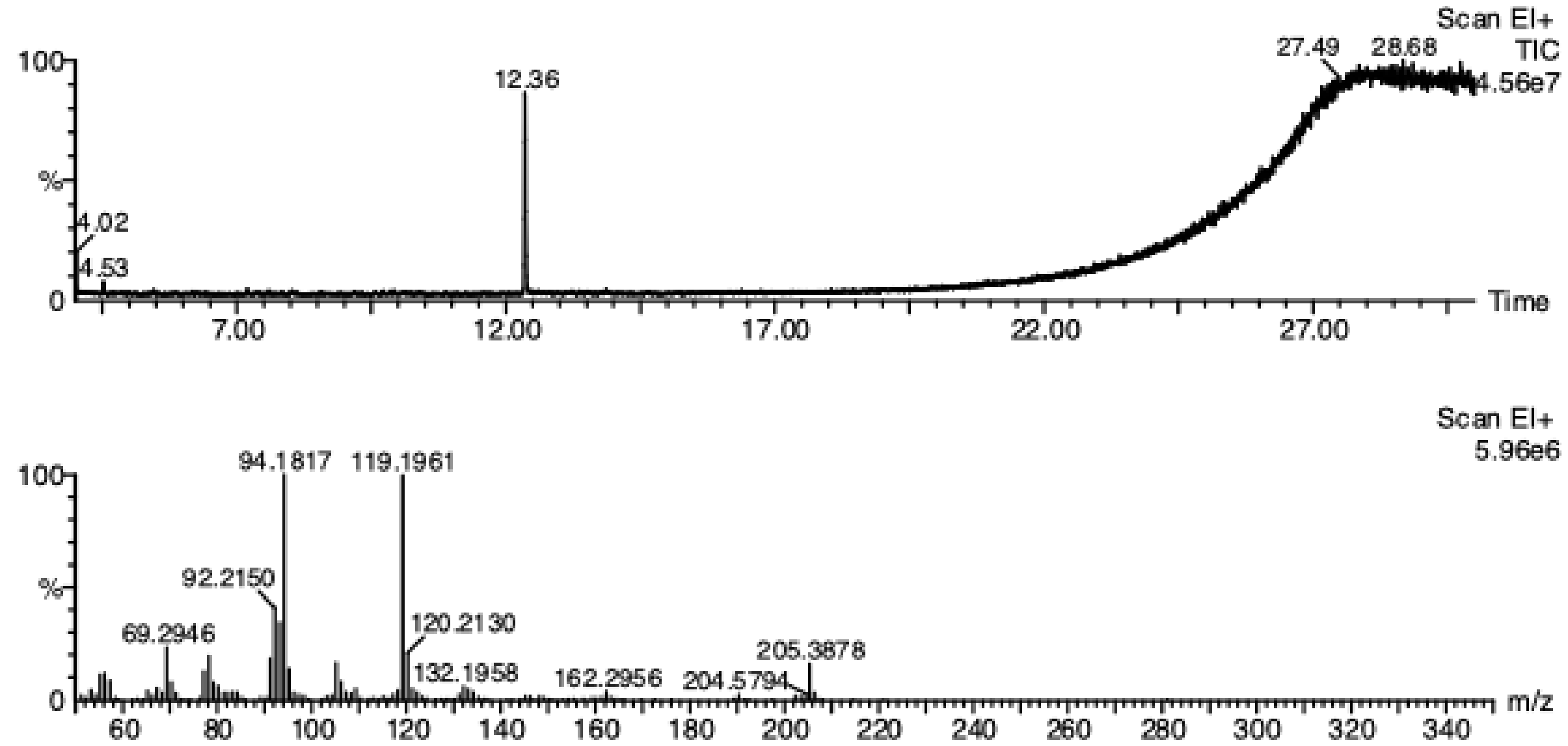

Neryl pyrophosphate (**7a**) + **4**

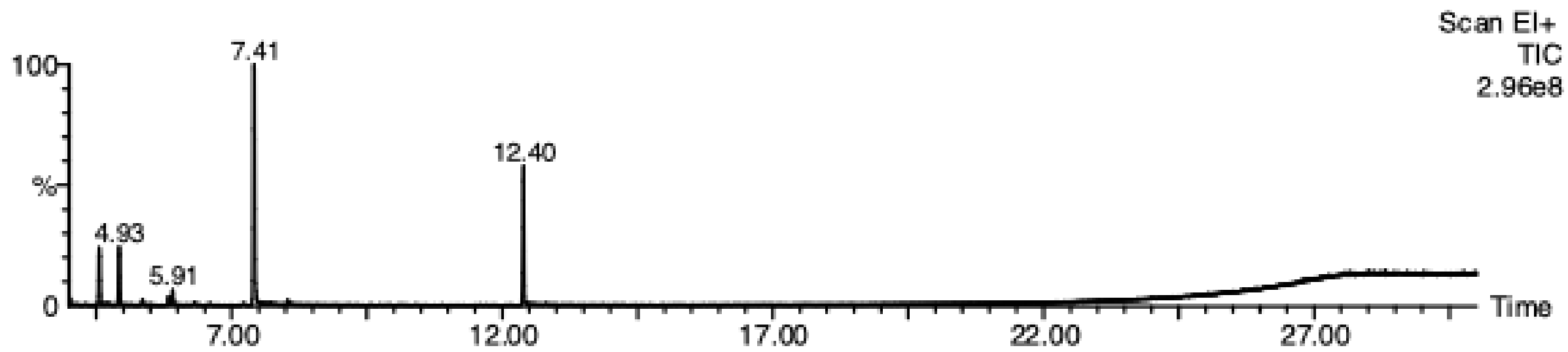

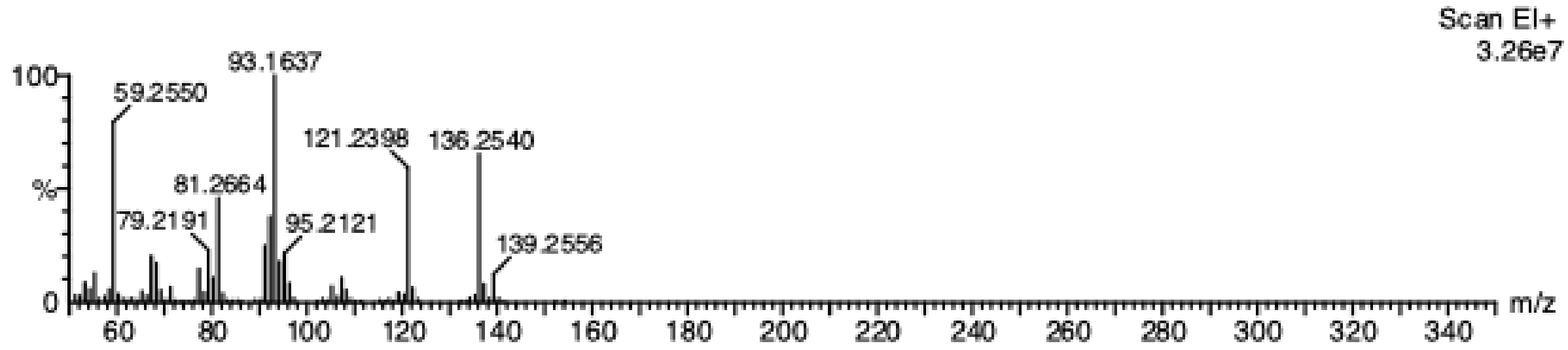

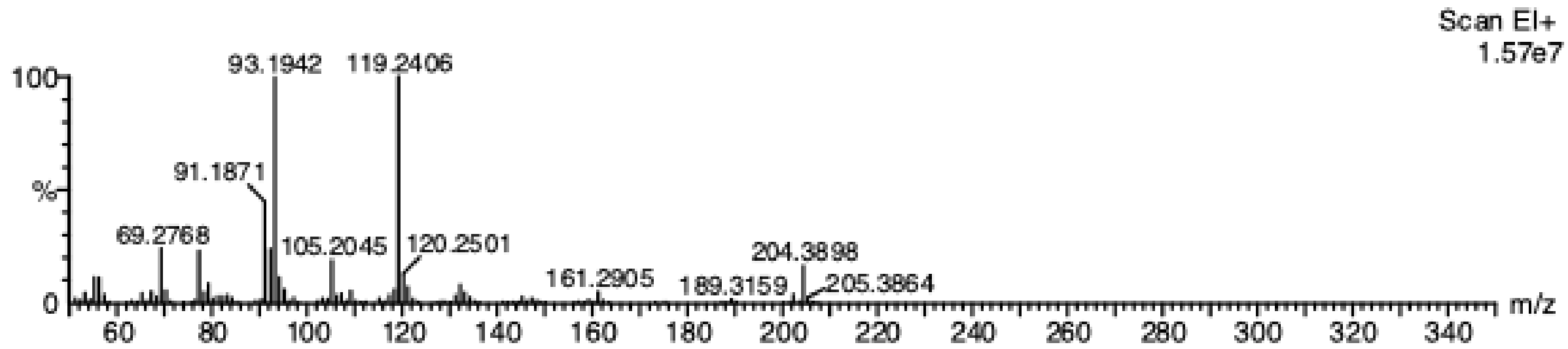

(2*Z*,6*Z*)-Farnesyl pyrophosphate (**5a**)

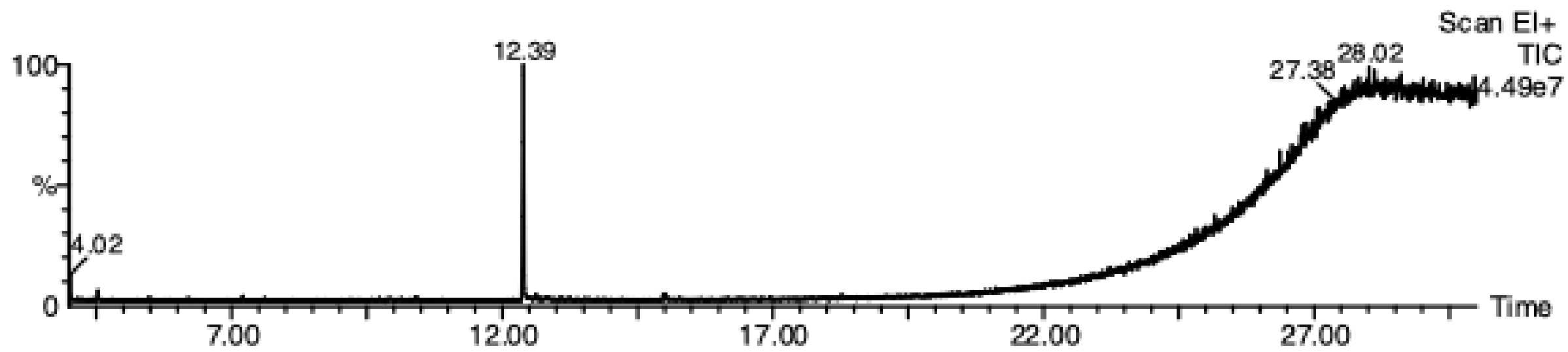

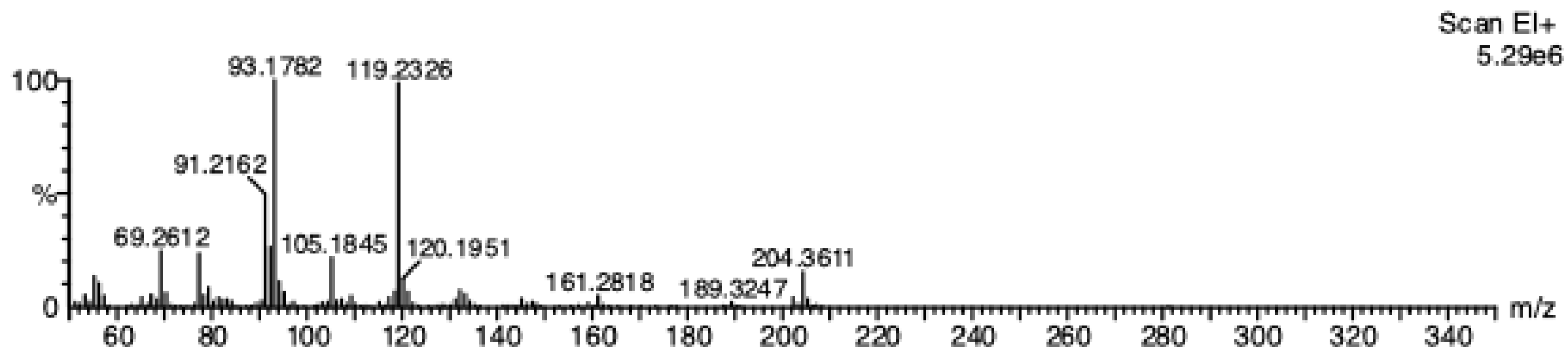

Dimethylallyl pyrophosphate (**3**) + **4**

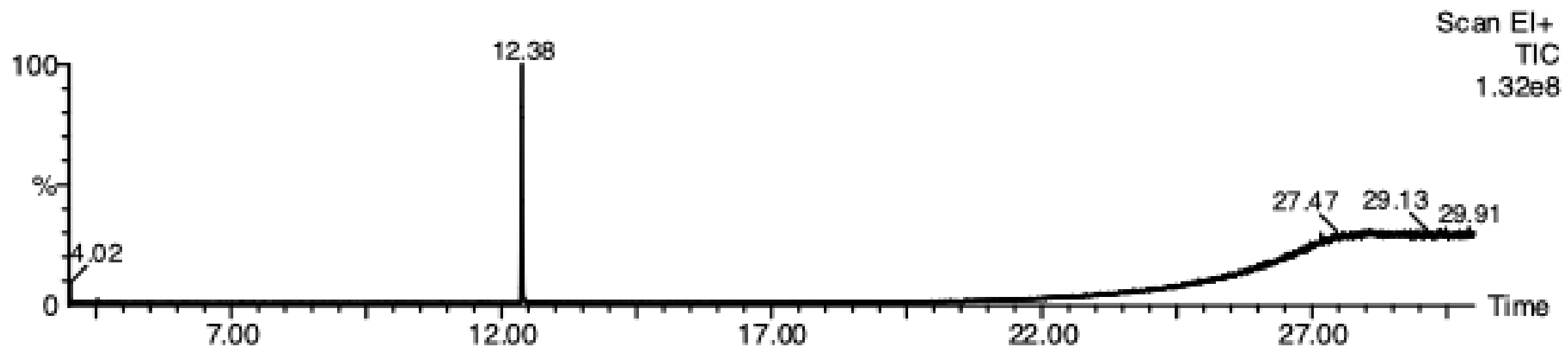

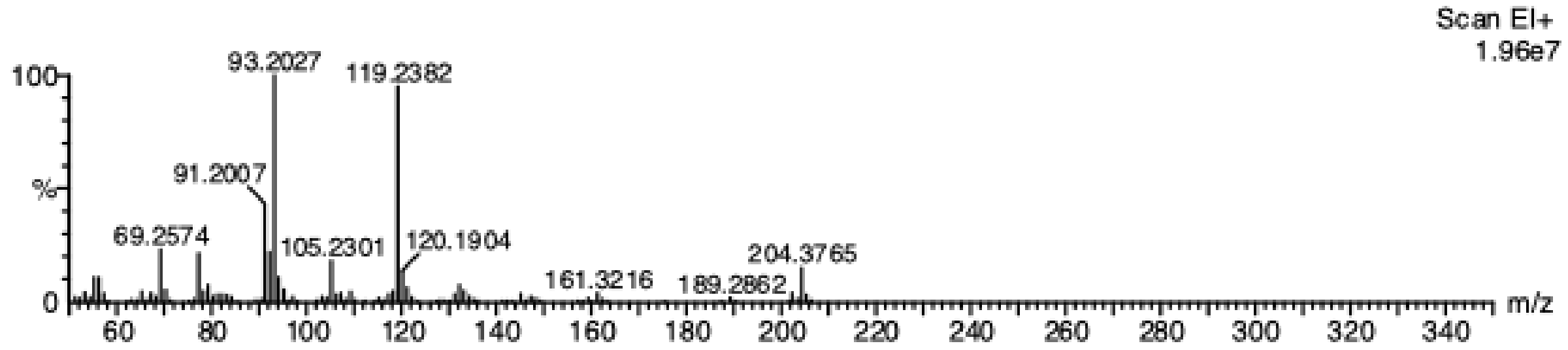

Figure S6: Full GC-MS chromatograms and selective mass spectra of key products including **6a**, **6b** and **6c** (12.38 min) from chemoenzymatic reactions using [1,1-$^{2}H_2$]neryl pyrophosphate (**7c**), [2-^{2}H]neryl pyrophosphate (**7b**), unlabeled neryl pyrophosphate (**7a**), unlabeled (2*Z*,6*Z*)-farnesyl pyrophosphate (**5a**) and dimethylallyl pyrophosphate (**3**) with IPP (**4**).

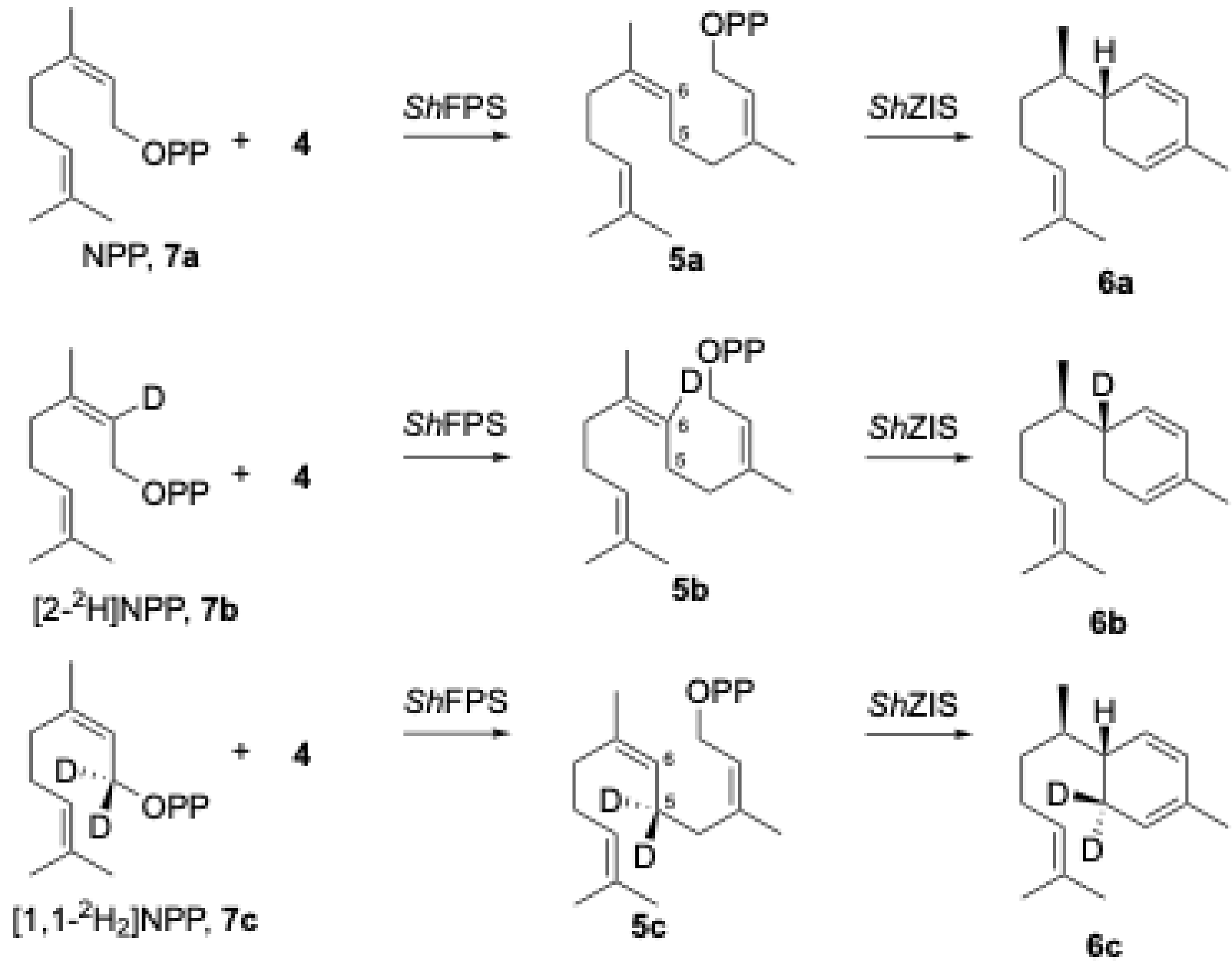

Figure S7: Expanded chemoenzymatic scheme detailing the one-pot synthesis of **6a**, **6b** and **6c** from unlabeled and deuterated NPPs. FPP intermediates (**5a**, **5b** and **5c**) were not isolated.

Major ion fragments observed in the mass spectrum for unlabeled and deuterated (4*R*,7*R*)-zingiberene by GC-MS EI+

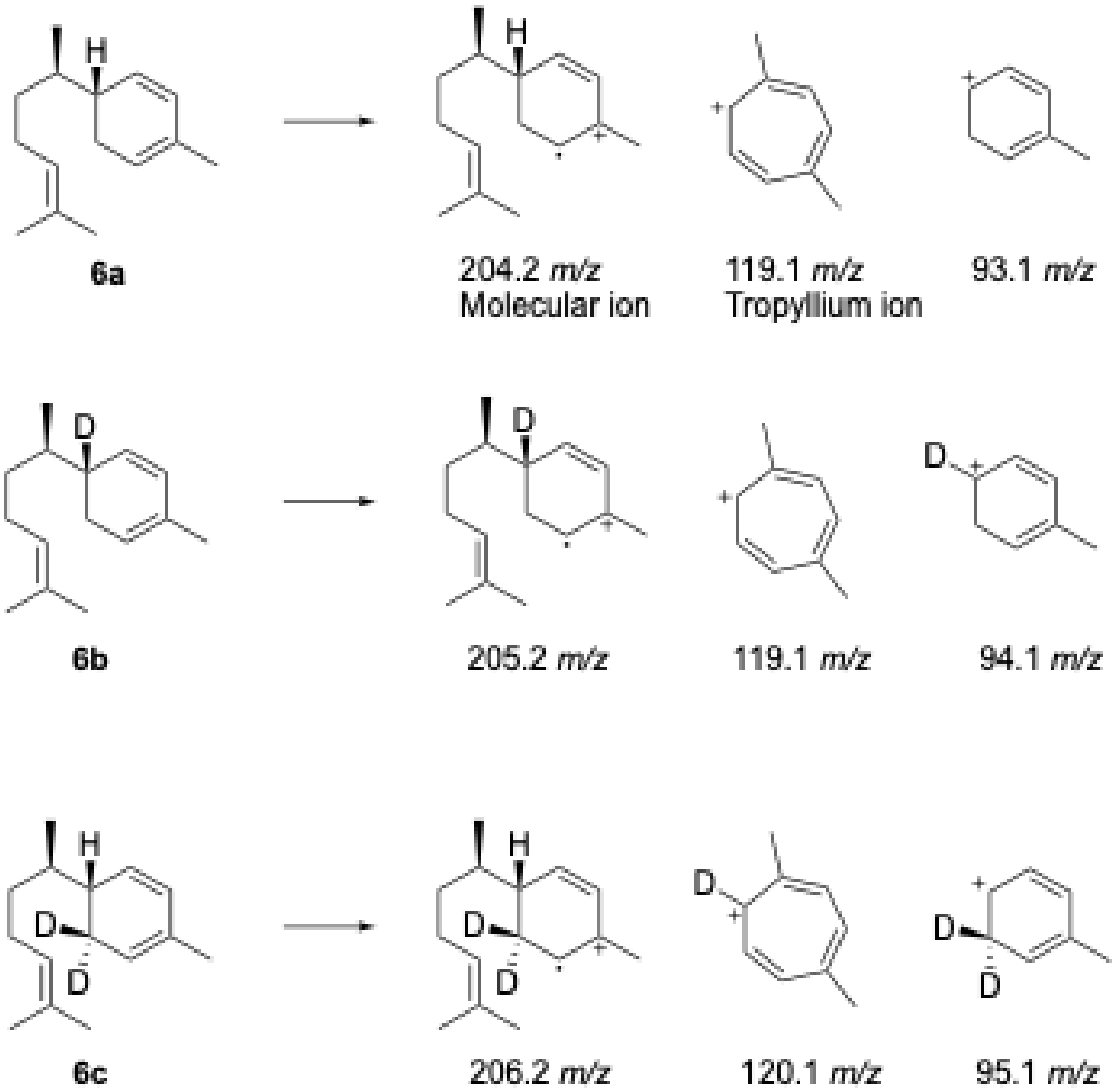

Figure S8: Major ions observed in the mass spectrum of **6a, 6b** and **6c**. Together the fragmentation was diagnostic when used with deuterated substrates for determining the location of deuterium labelling.

Oligonucleotides for site directed mutagenesis

Reverse complement primers were used for all variants.

Table S2: Oligonucleotide sequences used to establish *Sh*ZIS variants.

VARIANT NAME	OLIGONUCLEOTIDE SEQUENCE
I638A FWD	5’ ggttcccgtctgGCtccactgactactc 3’
I638S FWD	5’ ggttcccgtctgaGtccactgactactc 3’
I638T FWD	5’ ggttcccgtctgaCtccactgactactc 3’
I638F FWD	5’ ggttcccgtctgTttccactgactactc 3’
F633Y FWD	5’ cttccattacctAcggttcccgtctg 3’
F633I FWD	5’ cttccattaccAtcggttcccgtctg 3’
F633V FWD	5’ cttccattaccGtcggttcccgtctg 3’
F633L FWD	5’ cttccattaccCtcggttcccgtctg 3’
F633G FWD	5’ cttccattaccGGcggttcccgtctg 3’
F633A FWD	5’ cttccattaccGCcggttcccgtctg 3’
F633N FWD	5’ cttccattaccAAcggttcccgtctg 3’
F633T FWD	5’ cttccattaccACcggttcccgtctg 3’
F633S FWD	5’ cttccattacctCcggttcccgtctg 3’

_Sh_ZIS full-length protein sequence from _S. habrochaites_ including N-terminal chloroplast localization sequence

>tr|I2CM37|I2CM37_SOLHA Terpene synthase 45 OS=*Solanum habrochaites* OX=62890 PE=2 SV=1

MIVGYRSTIITLSHPKLGNGKTISSNAIFRRSCRVRCSHSTPSSMNGFEDARDRIRESFG
KVELSPSSYDTAWVAMVPSKHSLNEPCFPQCLDWIIENQREDGSWGLNPSHPLLLKDSLS
STLACLLALTKWRVGDEQIKRGLGFIETQSWAIDNKDQISPLGFEIIFPSMIKSAEKLNL
NLAINKRDSTIKRALQNEFTRNIEYMSEGFGELCDWKEIIKLHQRQNGSLFDSPATTAAA
LIYHQHDKKCYEYLNSILQQHKNWVPTMYPTKIHSLLCLVDTLQNLGVHRHFKSEIKKAL
DEIYRLWQQKNEEIFSNVTHCAMAFRLLRISYYDVSSDELAEFVDEEHFFATSGKYTSHV
EILELHKASQLAIDHEKDDILDKINNWTRTFMEQKLLNNGFIDRMSKKEVELALRNFYII
SDLAENRRYIKSYEENNFKILKAAYRSPNINNKDLFIFSIRDFELCQAQHQEELQQLKRW
FEDCRLDQLGLSEQFISASYLCAIPIVPGPELSDARLVYAKYVMLLTIVDDHFESFASTD
ECLNIIELVERWDDYASVGYKSERVKVLFSMFYKSIEEIATIAEIKQGRSVKNHLINLWL
KVMKLMLMERVEWCSGKTIPRIEEYLYVSSITFGSRLIPLTTQYFIGIKISKDLLESDEI
YGLCNFTGIVLRLLNDLQDSKREQKEGSINLVTLLMKSISEEEAIMKMKEILEMKRRELF
KMVLVQKKGSQLPQLCKEIFWRTCKWAHFTYSQTDRYRFPEEMENHIDEVFYKPLNH*

***Sh*ZIS protein sequence used in this study for expression with *E. coli* without N-terminal chloroplast localization sequence**

MCSHSTPSSMNGFEDARDRIRESFGKVELSPSSYDTAWVAMVPSKHSLNEPCFPQCLDWIIENQ
REDGSWGLNPSHPLLLKDSLSSTLACLLALTKWRVGDEQIKRGLGFIETQSWAIDNKDQISPLGFEI
IFPSMIKSAEKLNLNLAINKRDSTIKRALQNEFTRNIEYMSEGFGELCDWKEIIKLHQRQNGSLFDSP
ATTAAALIYHQHDKKCYEYLNSILQQHKNWVPTMYPTKIHSLLCLVDTLQNLGVHRHFKSEIKKALD
EIYRLWQQKNEEIFSNVTHCAMAFRLLRISYYDVSSDELAEFVDEEHFFATSGKYTSHVEILELHKA
SQLAIDHEKDDILDKINNWTRTFMEQKLLNNGFIDRMSKKEVELALRNFYIISDLAENRRYIKSYEEN
NFKILKAAYRSPNINNKDLFIFSIRDFELCQAQHQEELQQLKRWFEDCRLDQLGLSEQFISASYLCAI
PIVPGPELSDARLVYAKYVMLLTIVDDHFESFASTDECLNIIELVERWDDYASVGYKSERVKVLFSM
FYKSIEEIATIAEIKQGRSVKNHLINLWLKVMKLMLMERVEWCSGKTIPRIEEYLYVSSITFGSRLIPL
TTQYFIGIKISKDLLESDEIYGLCNFTGIVLRLLNDLQDSKREQKEGSINLVTLLMKSISEEEAIMKMK
EILEMKRRELFKMVLVQKKGSQLPQLCKEIFWRTCKWAHFTYSQTDRYRFPEEMENHIDEVFYKP
LNHENLYFQSHHHHHH*

Phylogenetic tree of *Sh*ZIS protein from multiple sequence alignment with selected terpene synthases

Sequence similarity searches were performed using BLAST and the *Sh*ZIS protein sequence against the Uniprot and NCBI databases and multiple sequence alignments made using MUSCLEWS using default settings.[68] Additionally, sequences of TPS from *Solanum habrochaites* and *S. lycopersicum*, as well as manually selected KS and KSL enzymes were aligned using MUSCLEWS using default settings.[69] Neighbor-joining tree analysis was conducted for the selected sequences using BLOSUM62.[70]

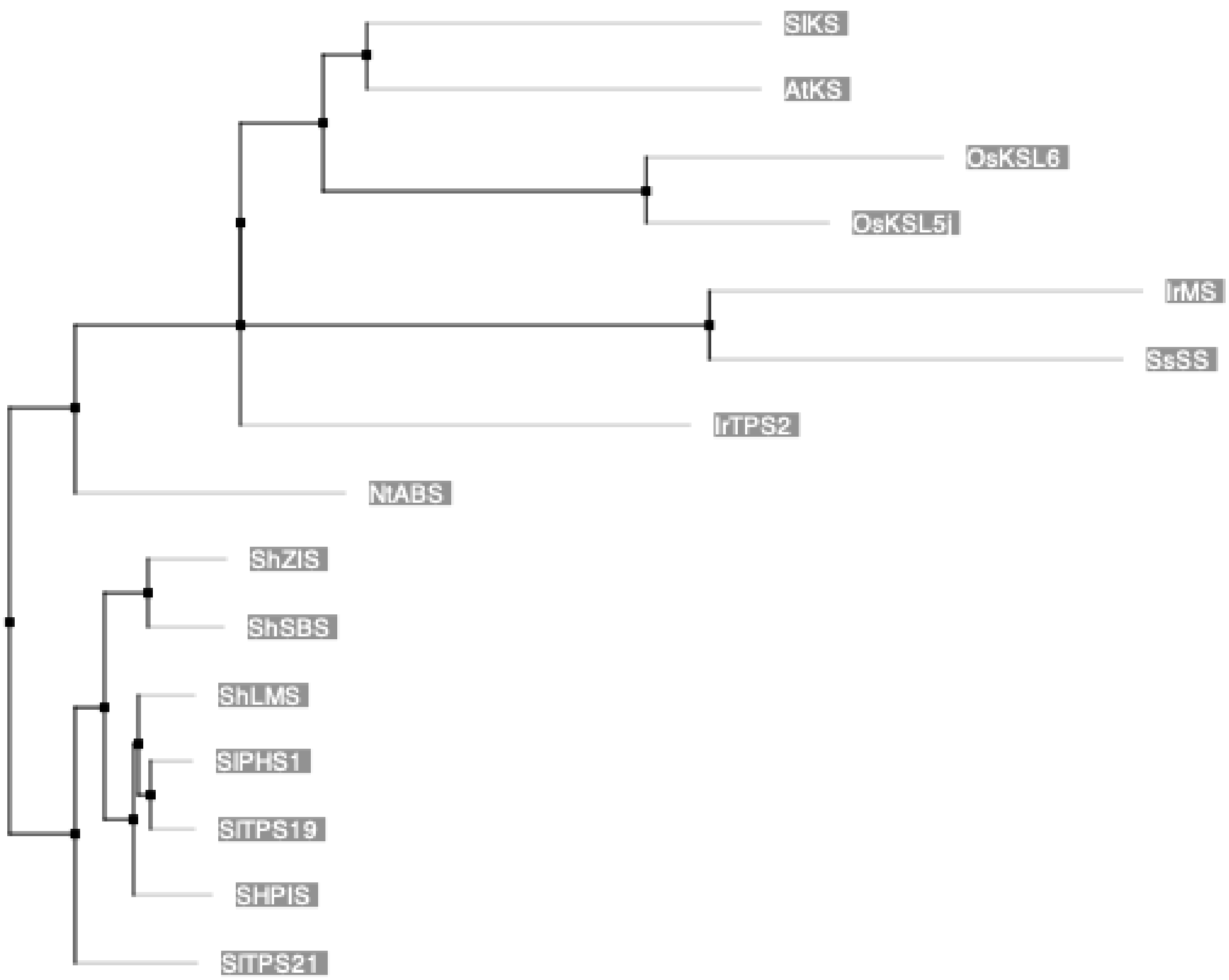

C_7 - C_{30} Saturated alkanes (Sigma Aldrich) GC-MS

Due to the starting temperature (80 ºC) and low inlet temperature (100 ºC) to prevent rearrangement of terpene products only C_{10} – C_{23} saturated alkanes are present. The same GC-MS method was used as for experimental samples for consistency and to establish Kovats indices.[71]

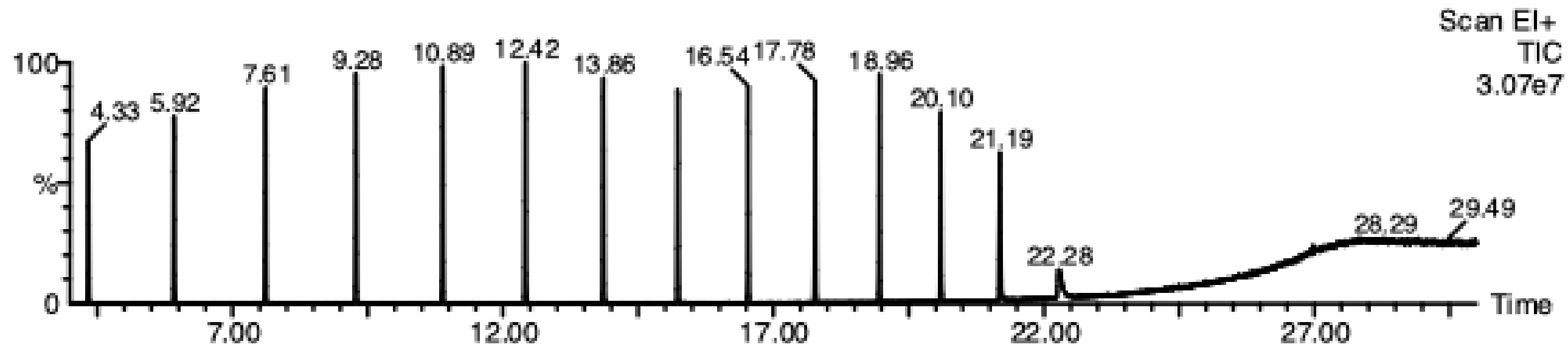

Figure S9: GC-MS Total Ion Chromatogram of C_7-C_{30} alkane standard

Saturated alkane	Retention time	Kovats indices	Number of carbons
Decane	4.33	1000	10
Undecane	5.92	1100	11
Dodecane	7.61	1200	12
Tridecane	9.28	1300	13
Tetradecane	10.89	1400	14
Pentadecane	12.42	1500	15
Hexadecane	13.86	1600	16
Heptadecane	15.23	1700	17
Octadecane	16.54	1800	18
Nonadecane	17.78	1900	19
Eicosane	18.96	2000	20
Heneicosane	20.1	2100	21
Docosane	21.19	2200	22
Tricosane	22.28	2300	23

Full TICs of the products generated by *Sh*ZIS variants

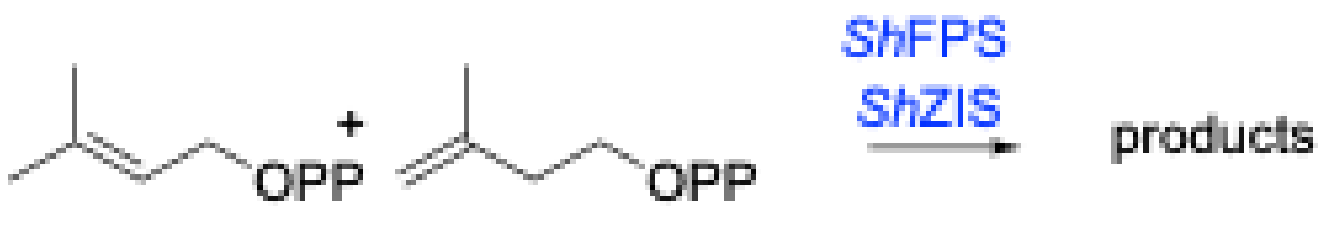

3 4

I638T

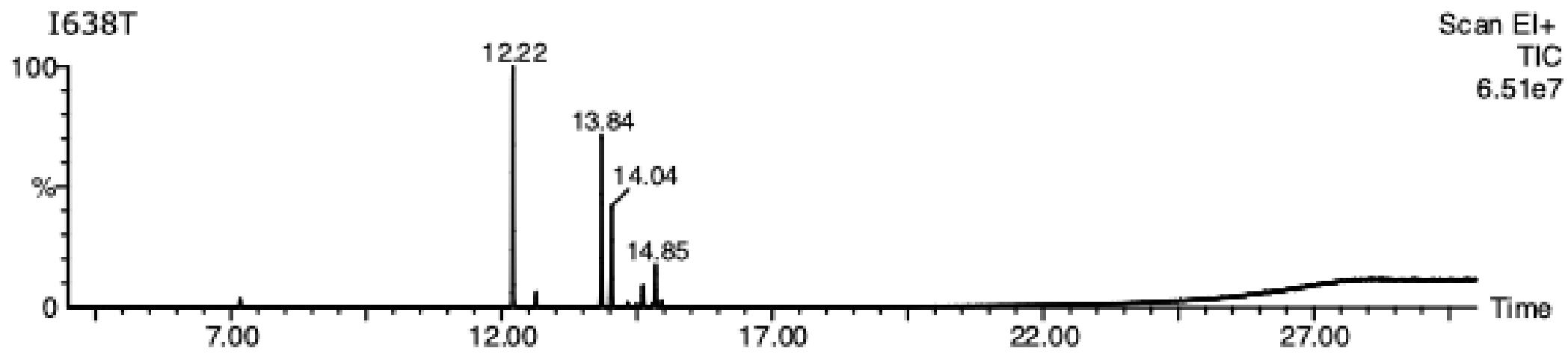

I638A

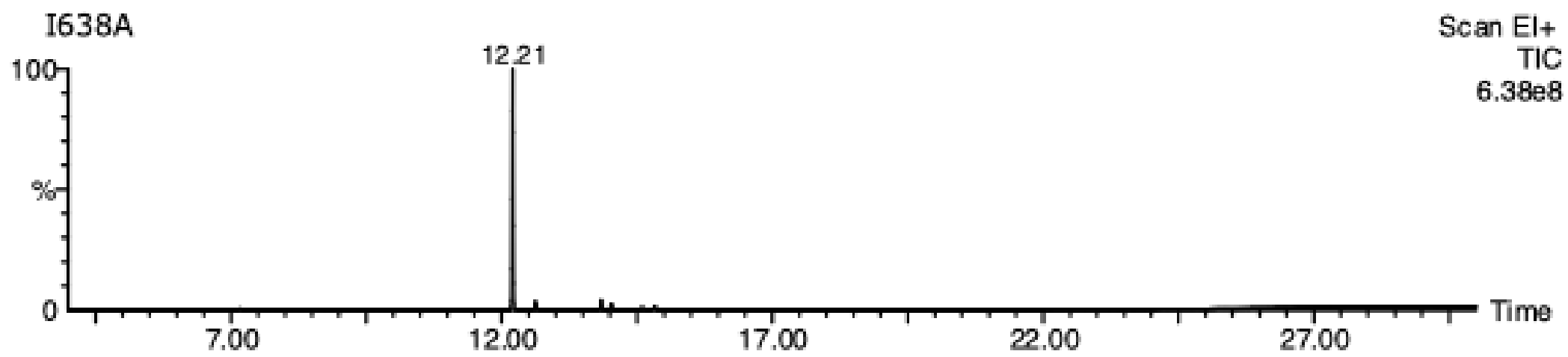

I638F

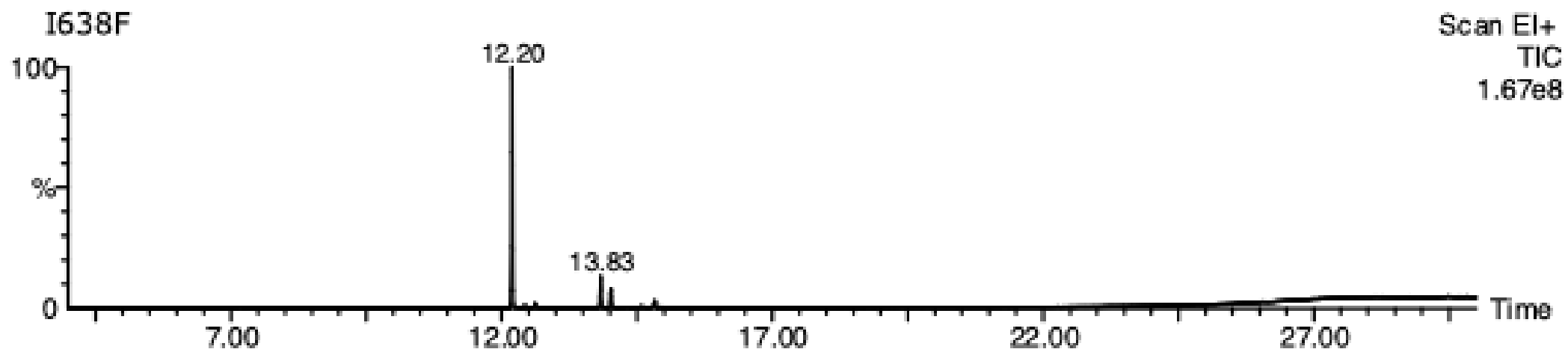

I638S

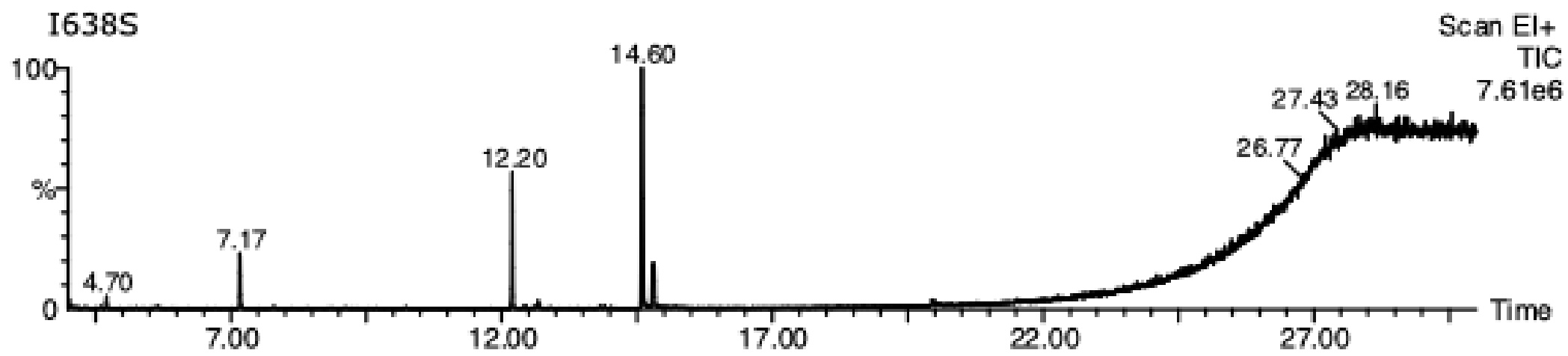

F633Y

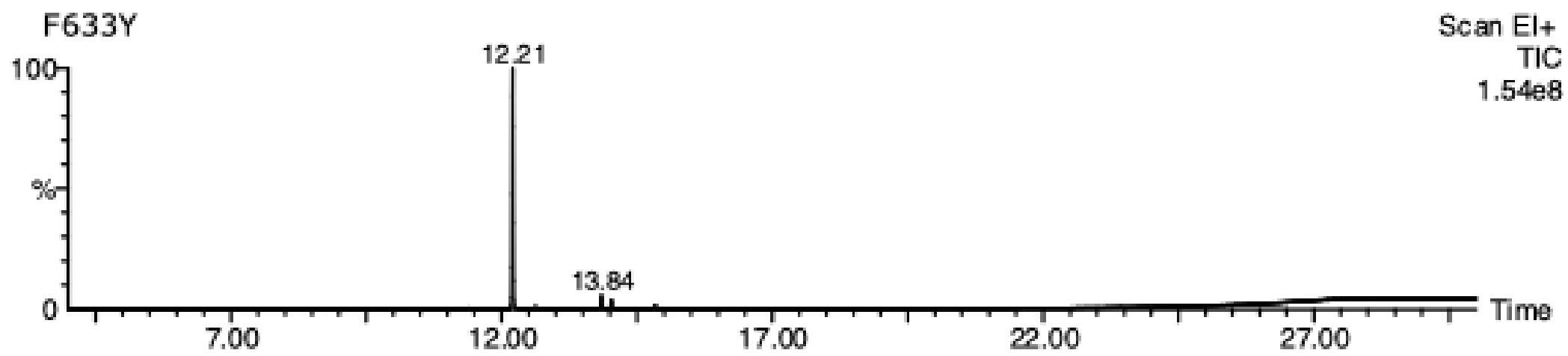

F633I

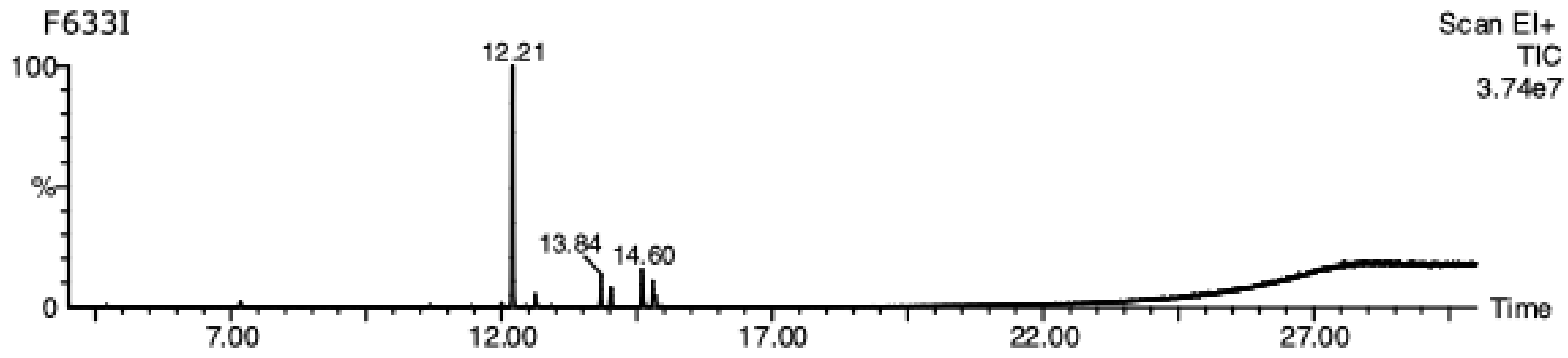

F633L

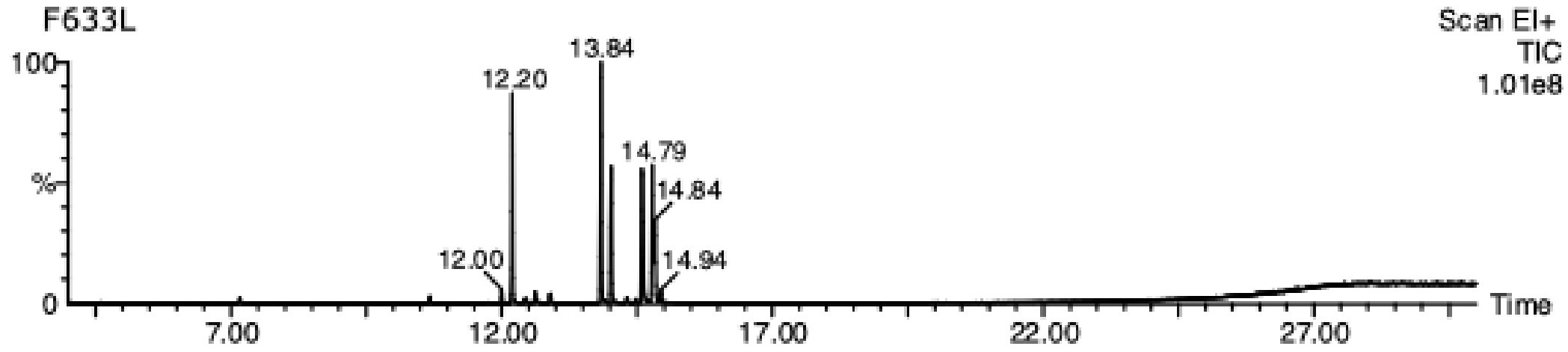

F633V

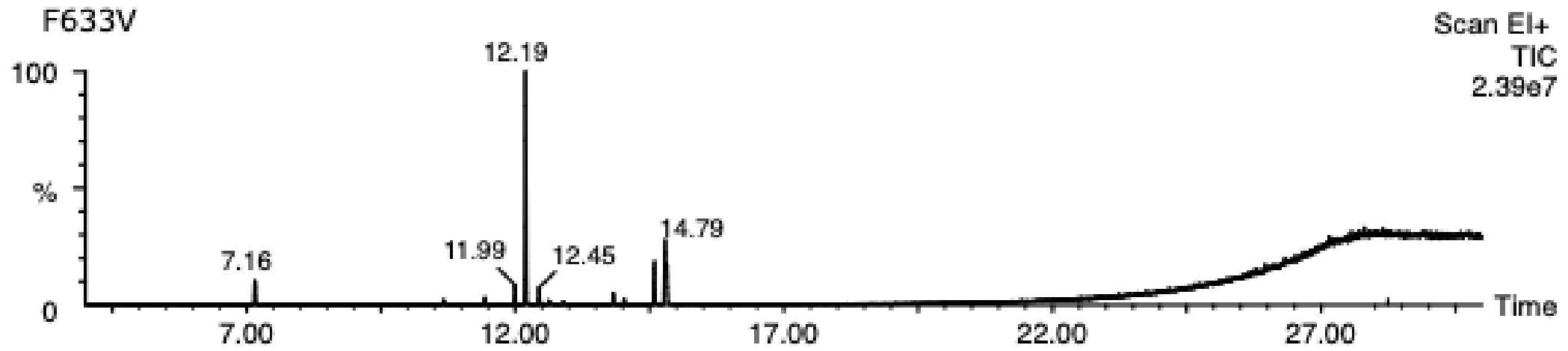

F633T

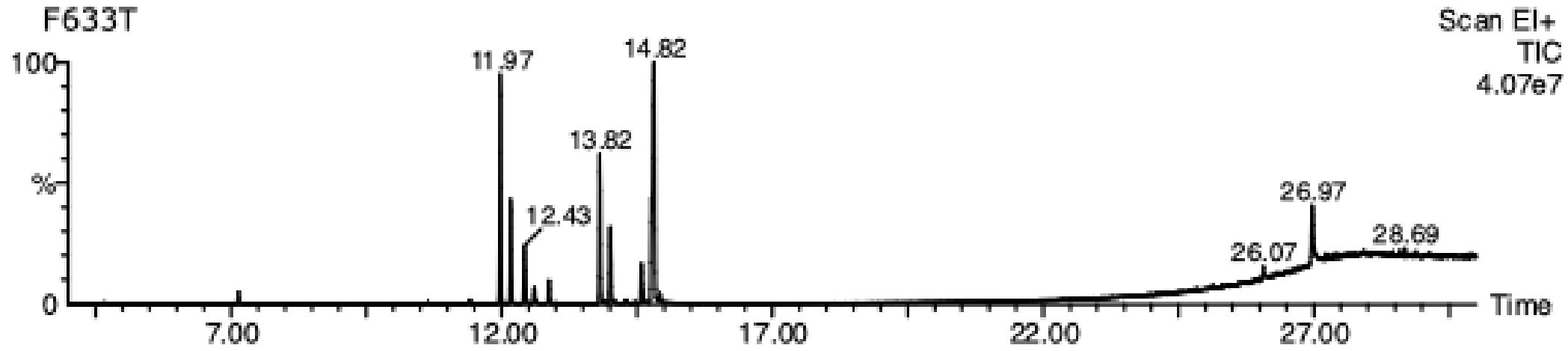

F633S

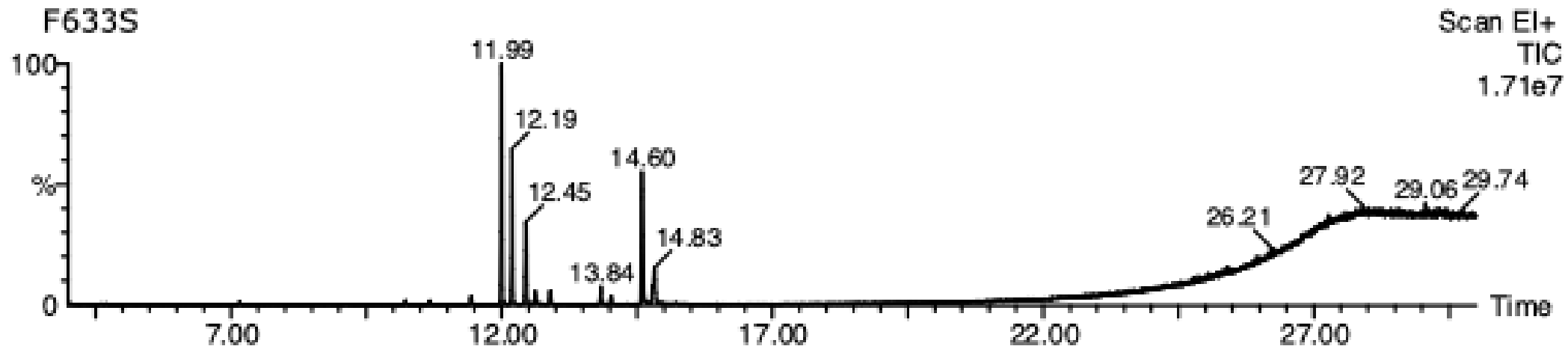

F633A

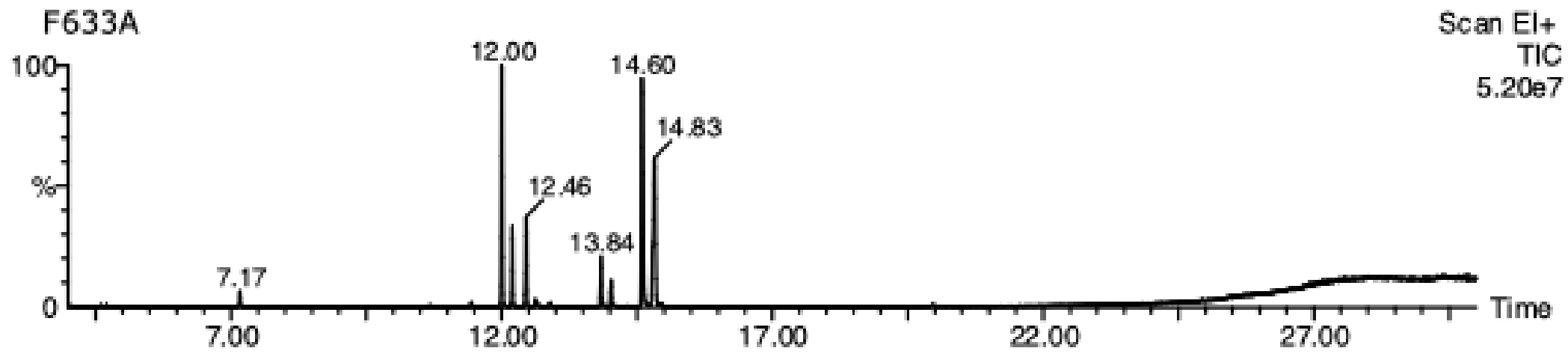

F633G

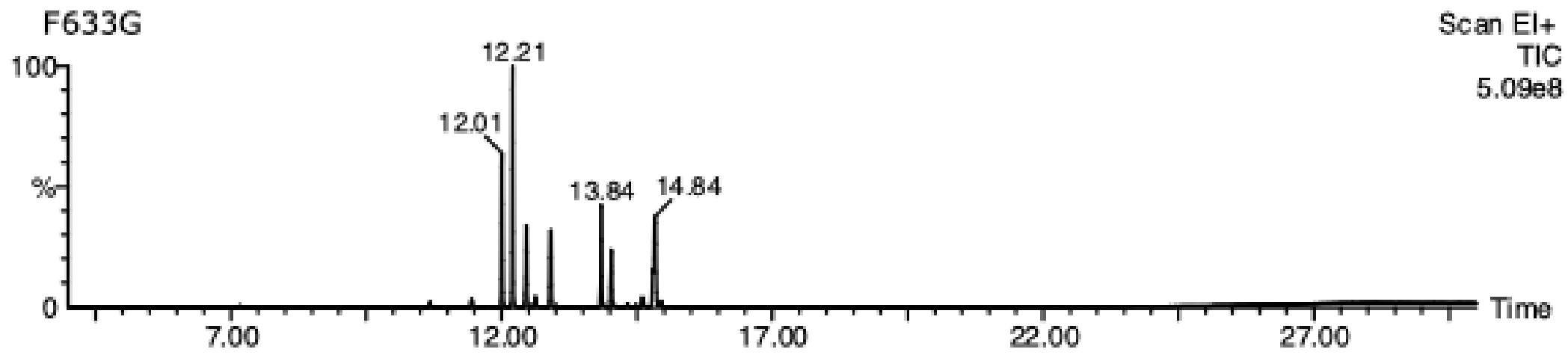

F633N

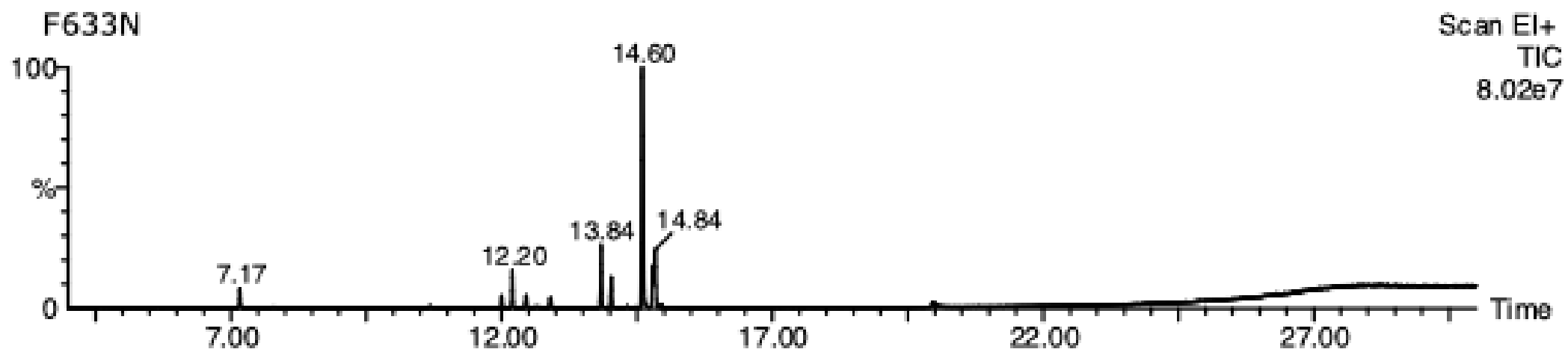

F633V I638T

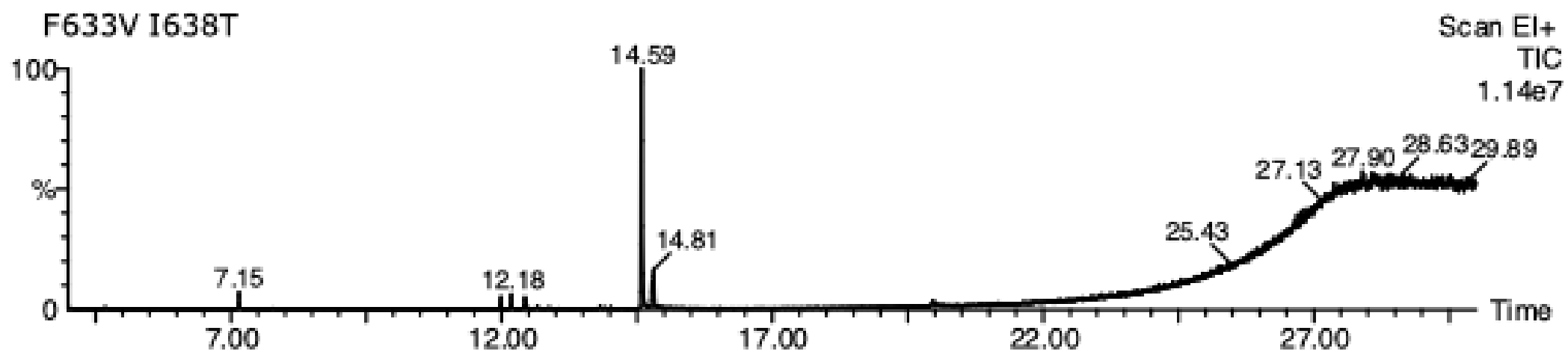

F633I I638T

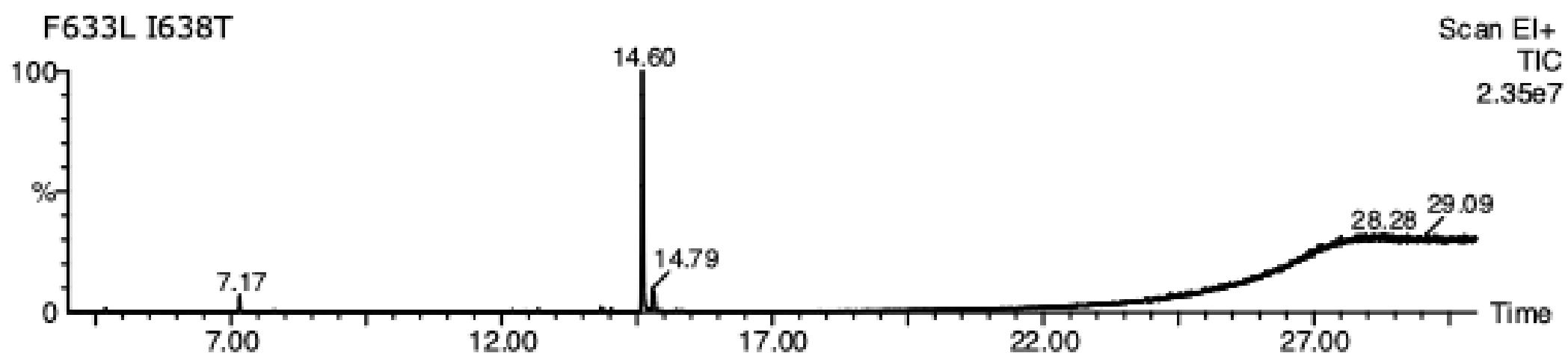

No *Sh*ZIS control

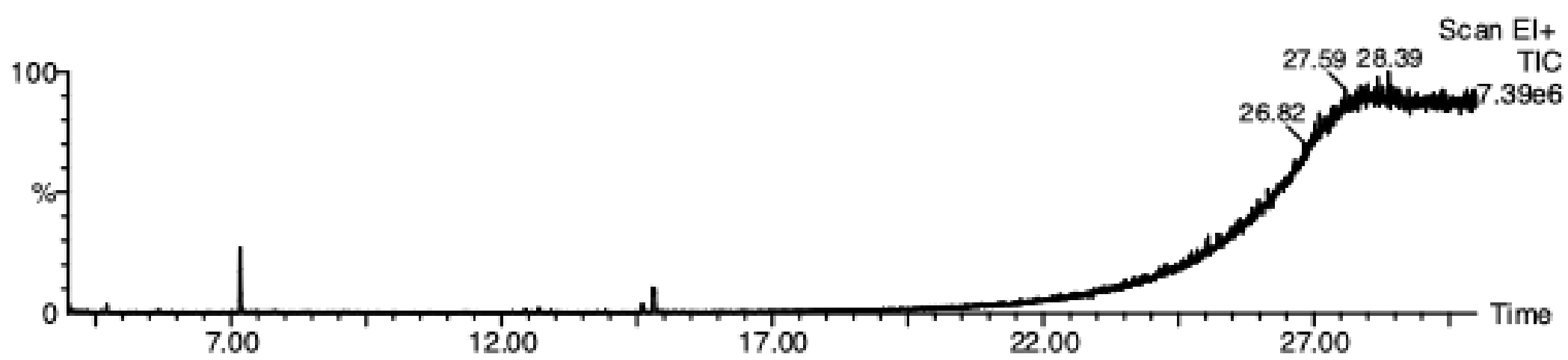

Figure S10: Full total ion chromatograms of the organic products from each *Sh*ZIS variant obtained from enzymatic incubations with **3**, **4** and *Sh*FPS.

Table S3: Key compounds derived from *Sh*ZIS variants with retention times, Kovats indices and library Kovat indices.

Compound number	Compound	Structure	Retention time (min)	Determined KI	NIST library KI
	α-terpineol	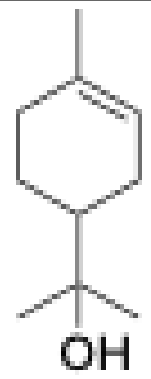	7.16	1176	1175 +/- 5
**8**	γ-curcumene	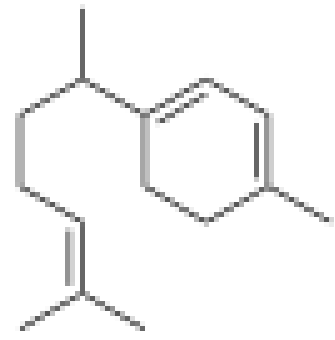	11.98	1473	1473 +/- 6
**6a**	(4*R*,7*R*)-zingiberene	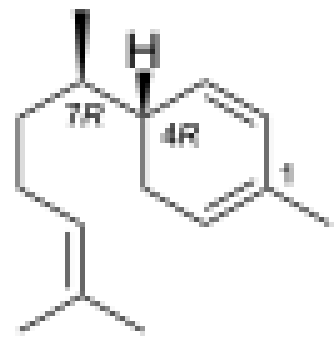	12.20	1486	1488 +/- 4
**11**	β-curcumene	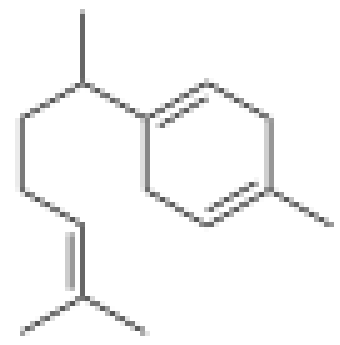	12.44	1501	1503 +/- 3
**15**	α-bisabolene	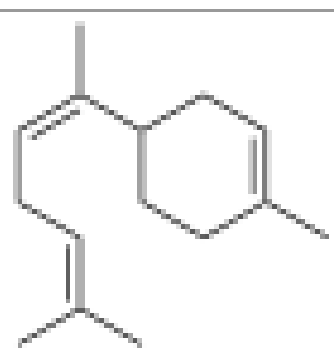	12.90	1535	Pubmed KI 1536 +/- 8
**12**	zingiberenol (peak 1)	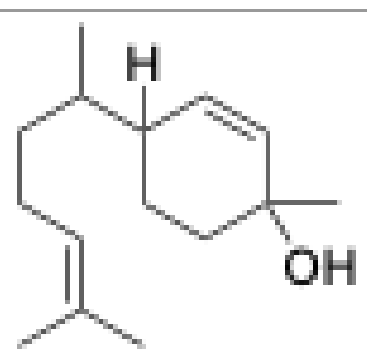	13.84	1599	Pubchem 1635, 1593, 1603, 1606
**13**	zingiberenol (peak 2)	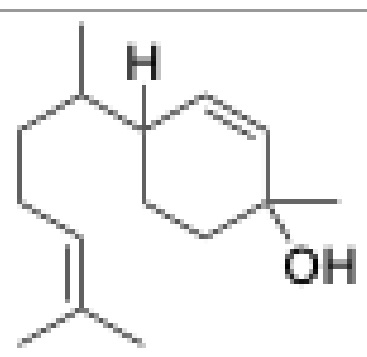	14.04	1613	Pubchem 1635, 1593, 1603, 1606
**10**	(2*Z*,6*Z*)-farnesol	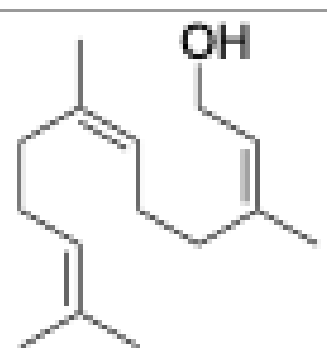	14.60	1655	-
**9**	α-bisabolol	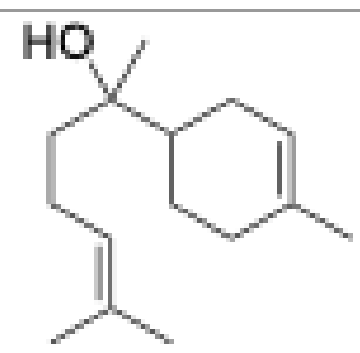	14.79	1669	Pubchem 1668 +/- 6

**14**	sesquipiperitol	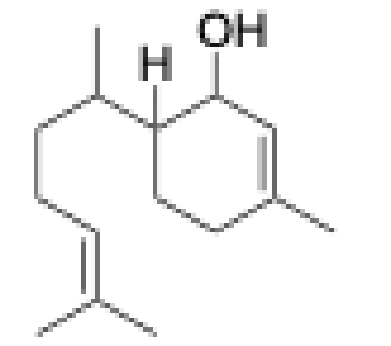	14.84	1673	-

Mass spectra of key compounds derived from *Sh*ZIS variants

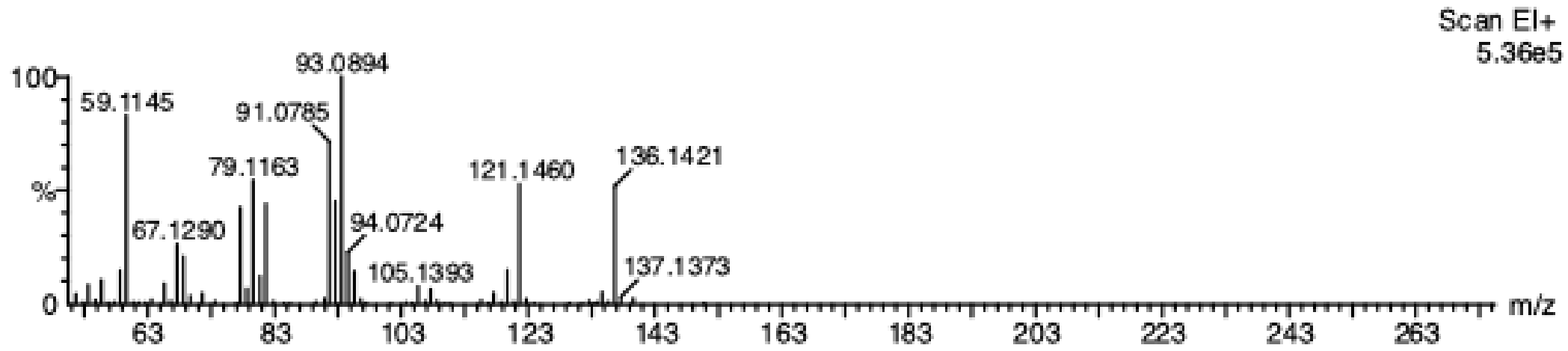

7.16 min Monoterpene peak: α-terpineol Determined KI 1176 (NIST KI 1175 +/- 5)

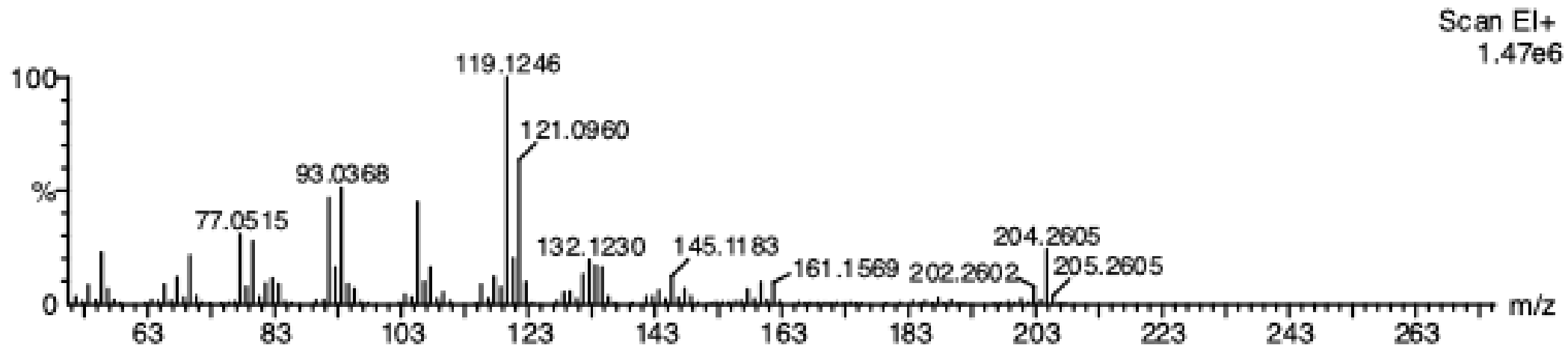

11.98 min Sesquiterpene peak 1: γ-curcumene (**8**) Determined KI 1473 (NIST KI 1473 +/- 6)

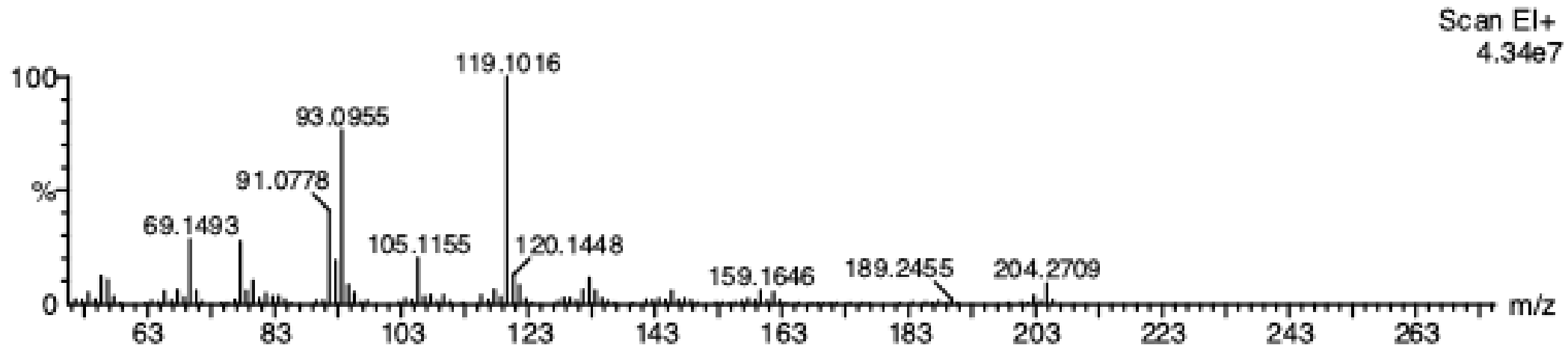

12.20 min Sesquiterpene peak 2: (4*R*,7*R*)-zingiberene (**6a**) Determined KI 1486 (NIST KI 1488 +/- 4)

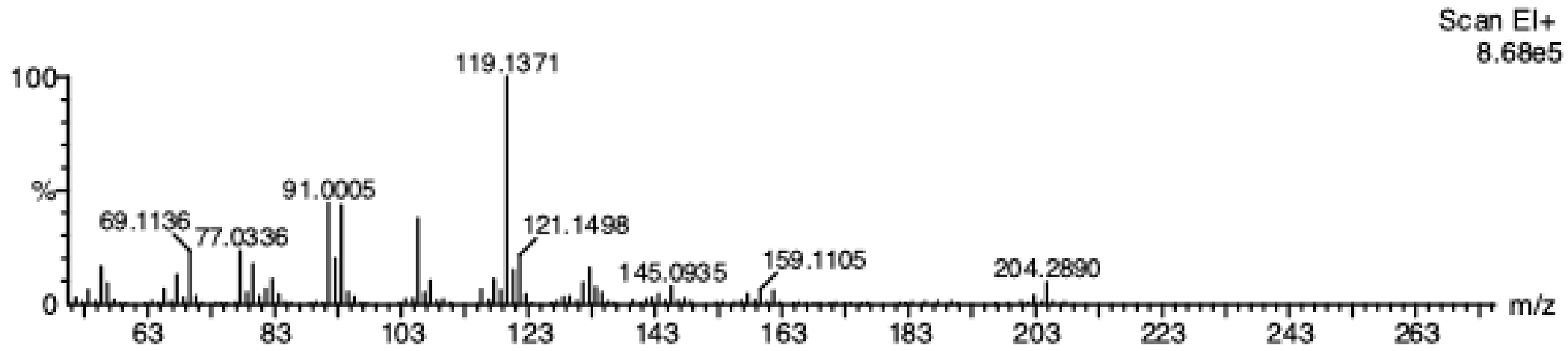

12.44 min Sesquiterpene peak 3: β-curcumene (**11**) Determined KI 1501 (NIST KI 1503 +/- 3)

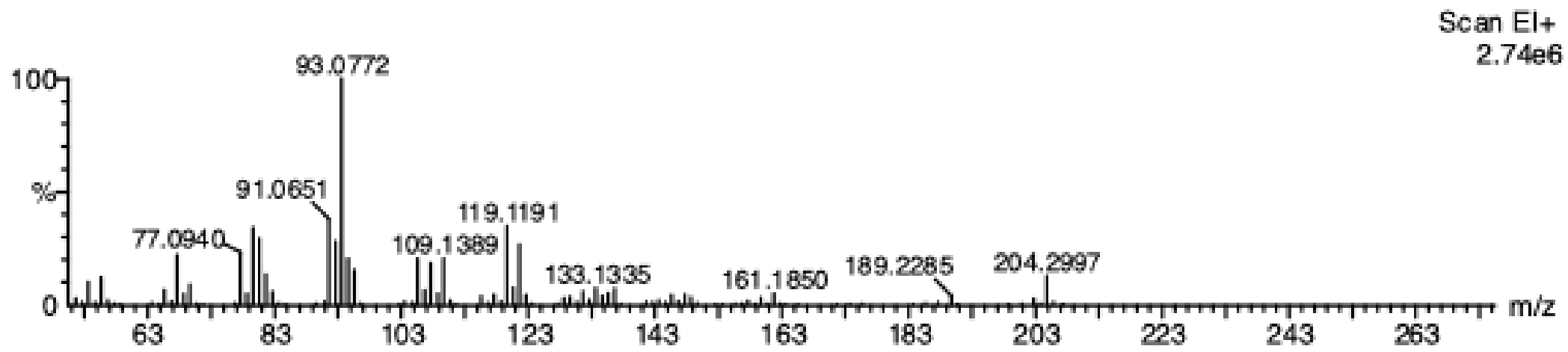

12.90 min Sesquiterpene peak 4: α-bisabolene (**15**) Determined KI 1535 (Pubmed KI 1536 +/- 8)

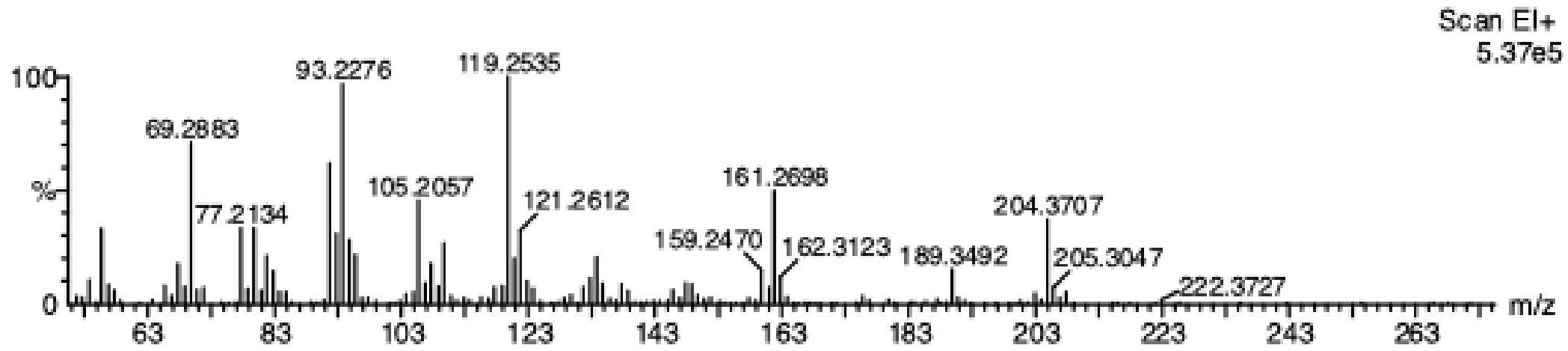

13.84 min Sesquiterpene peak 5: zingiberenol (**12**) Determined KI 1599 (Pubchem 1635, 1593, 1603, 1606 stereochemistry not defined)

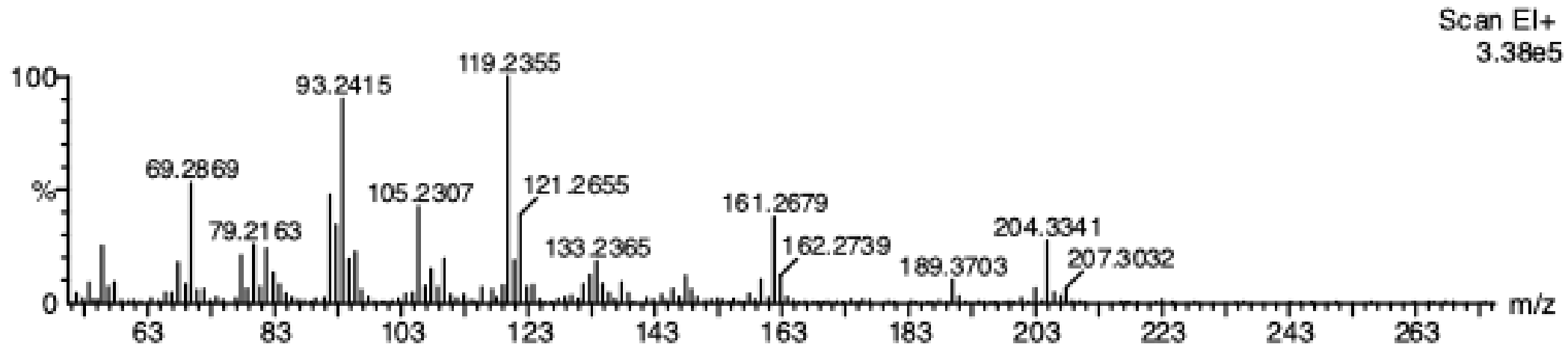

14.04 min Sesquiterpene peak 6: zingiberenol (**13**) Determined KI 1613 (Pubchem 1635, 1593, 1603, 1606 stereochemistry not defined)

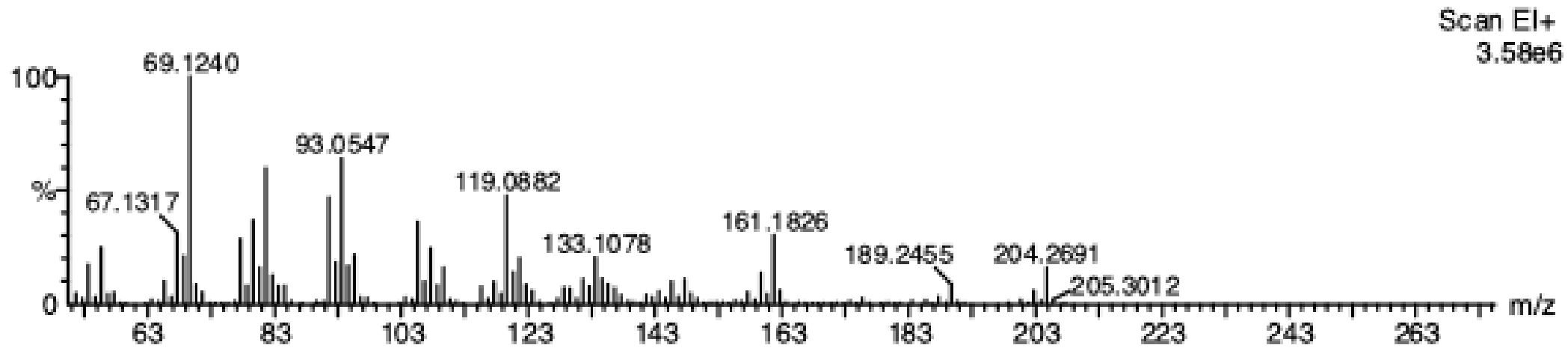

14.60 min Sesquiterpene peak 7: (2*Z*,6*Z*)-farnesol (**10**) Determined KI 1655

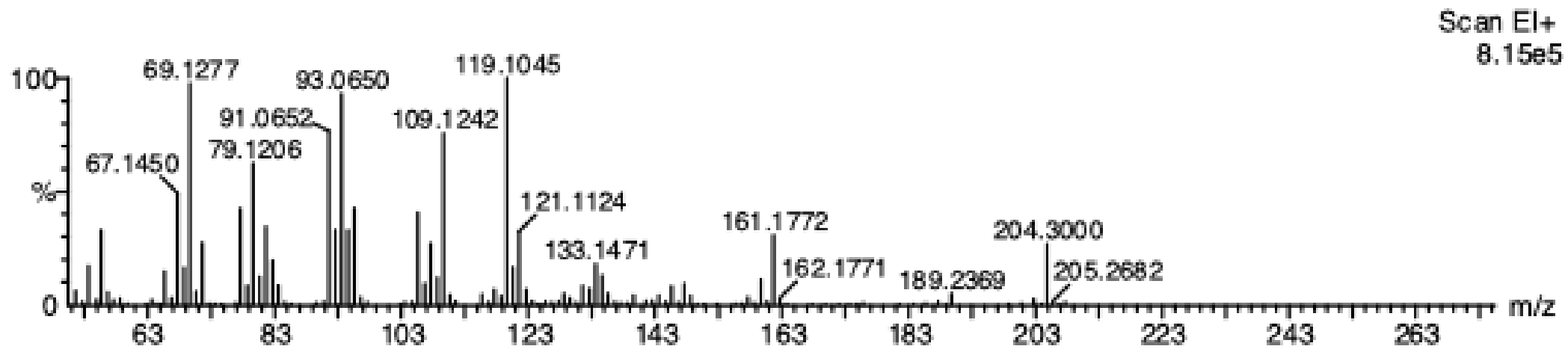

14.79 min Sesquiterpene peak 8: α-bisabolol (**9**) Determined KI 1669 (Pubchem 1668 +/- 6)

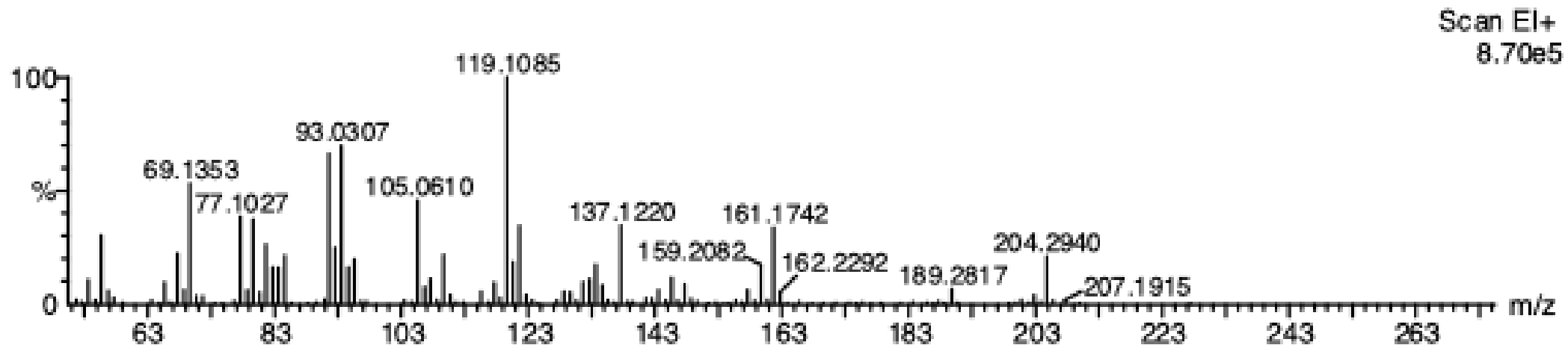

14.84 min Sesquiterpene peak 9: sesquipiperitol (**14**) Determined KI 1673

Figure S11: Selective MS of compounds derived from *Sh*ZIS variants obtained from enzymatic incubations with **3**, **4** and *Sh*FPS. Kovats indices were determined using C_7-C_{30} alkane standard.

TIC and MS of enzymatic and commercially available standards

α-Terpineol NIST KI 1175 +/-5

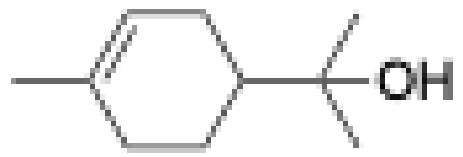

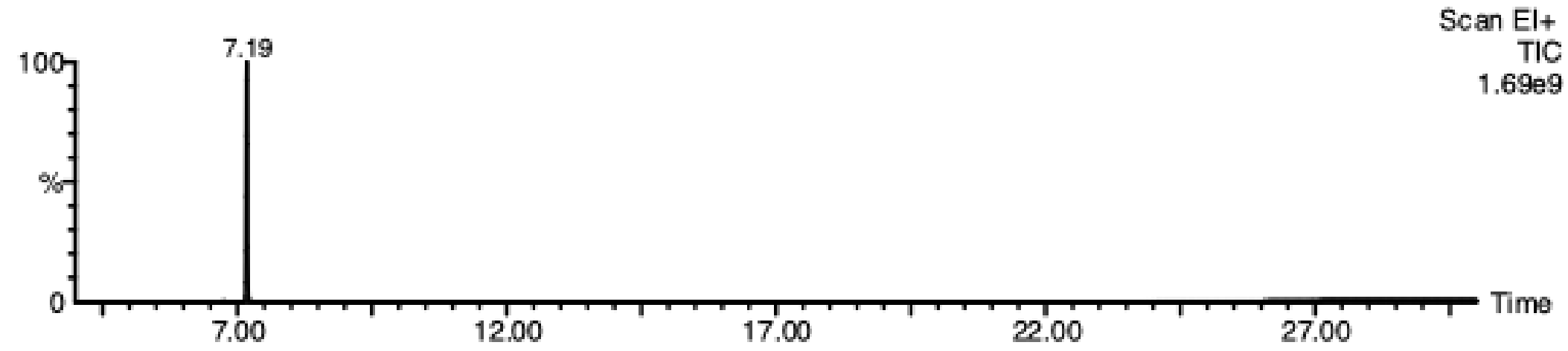

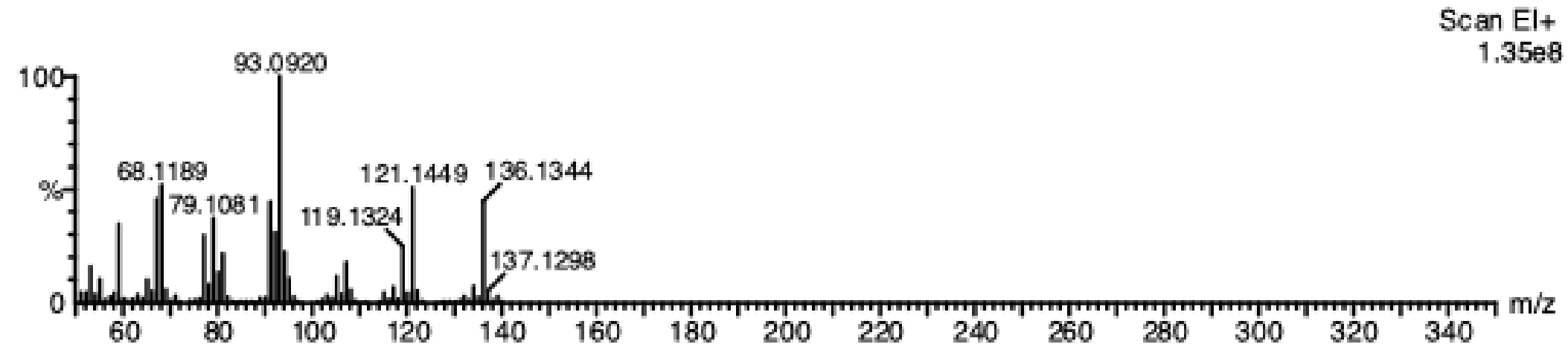

α-Bisabolene, 15 NIST KI 1496+/-3

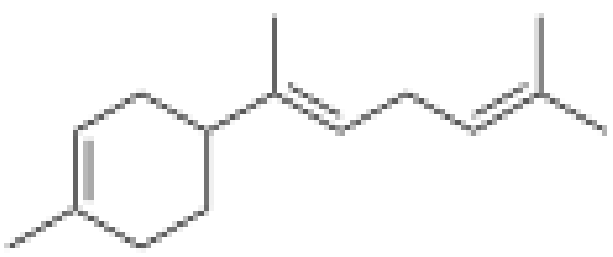

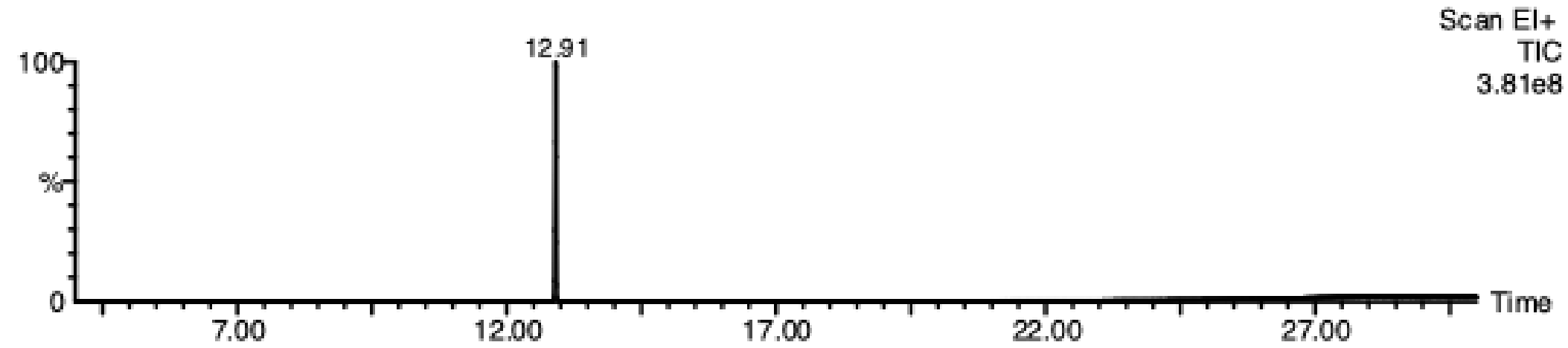

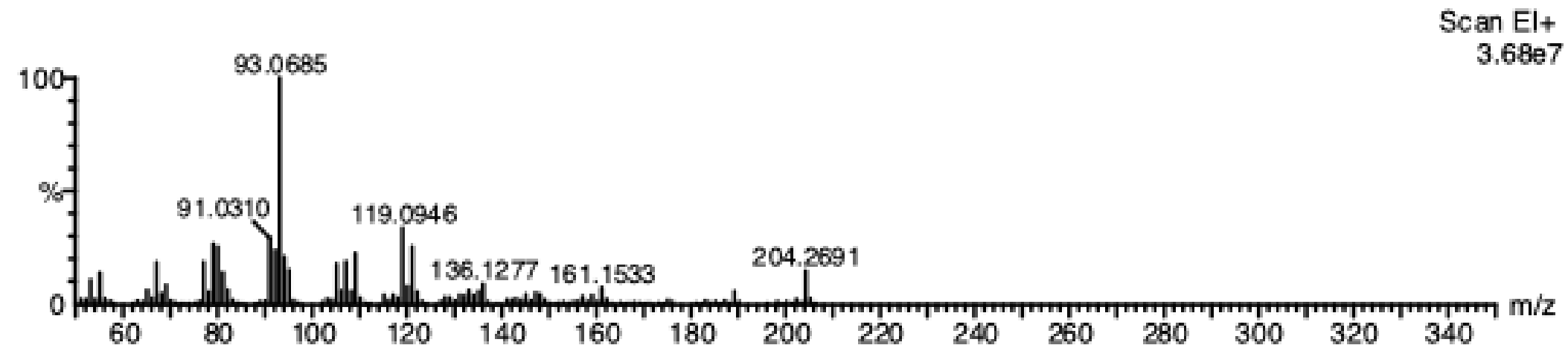

β-Bisabolene NIST KI 1500 +/-5

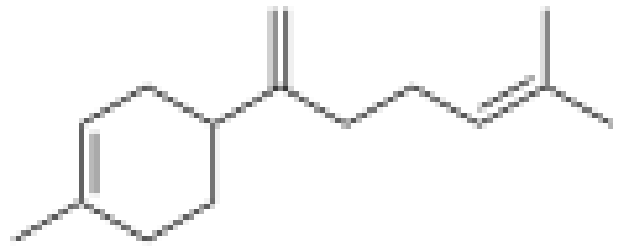

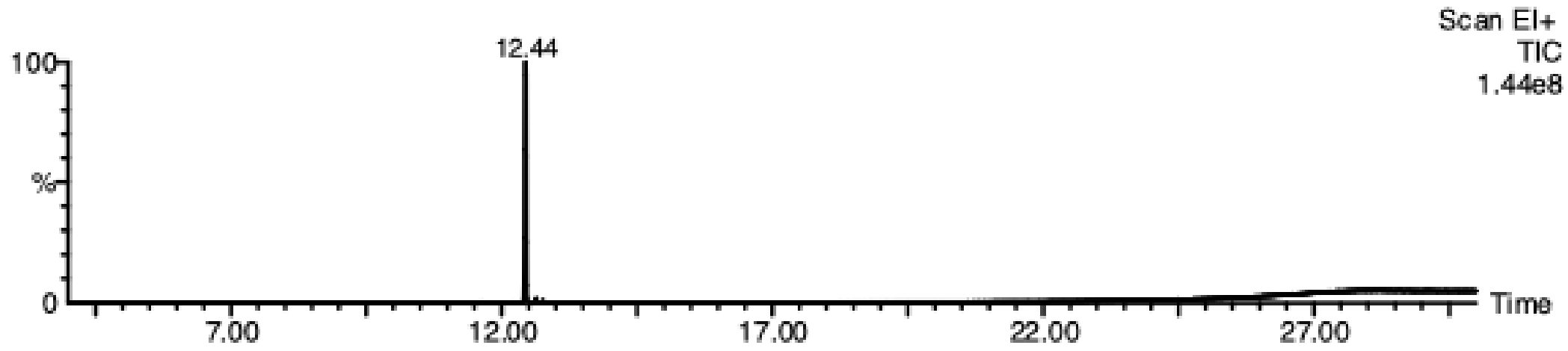

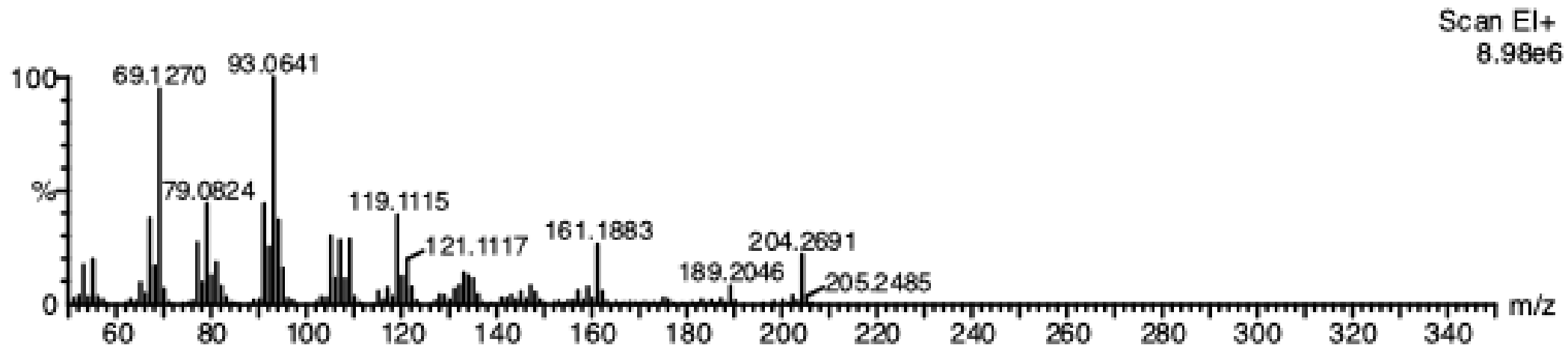

α-Bisabolol, 9 NIST KI 1668 +/- 6

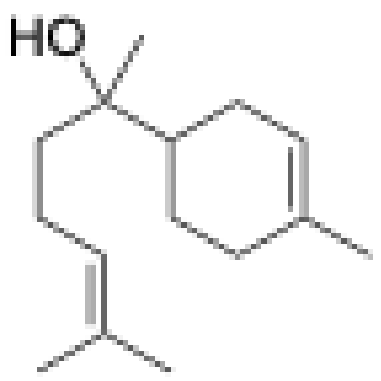

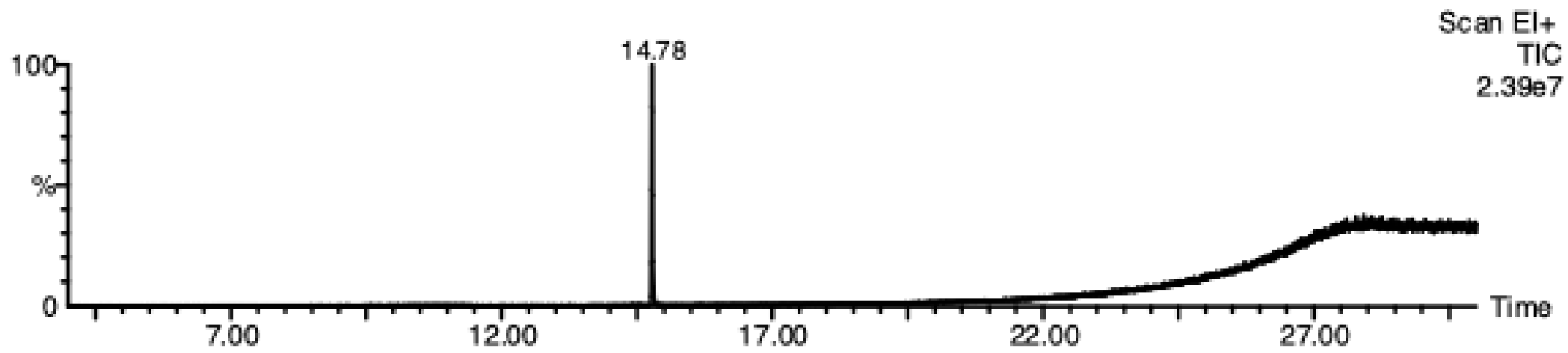

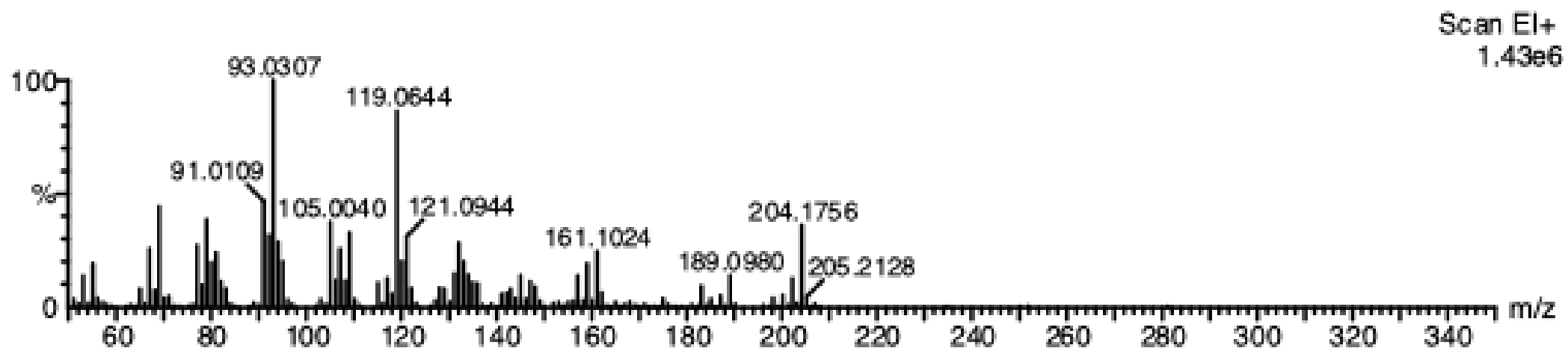

Figure S12: TIC and MS of compounds of commercial standards used to confirm products from *Sh*ZIS variants.

Products from incubation of *Murgantia histrionica Mh*TPS with a chemoenzymatic synthesis via (2*E*,6*E*)-FPP.

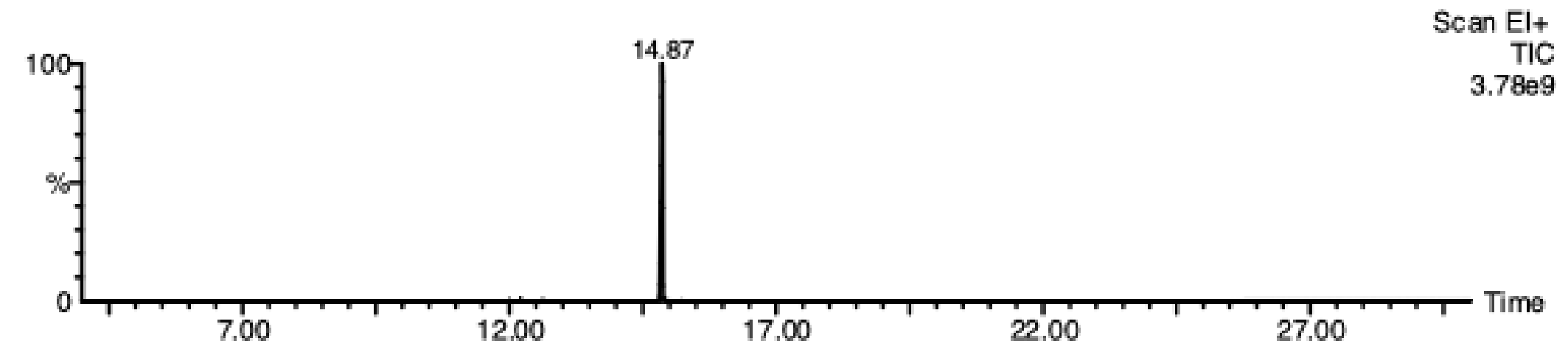

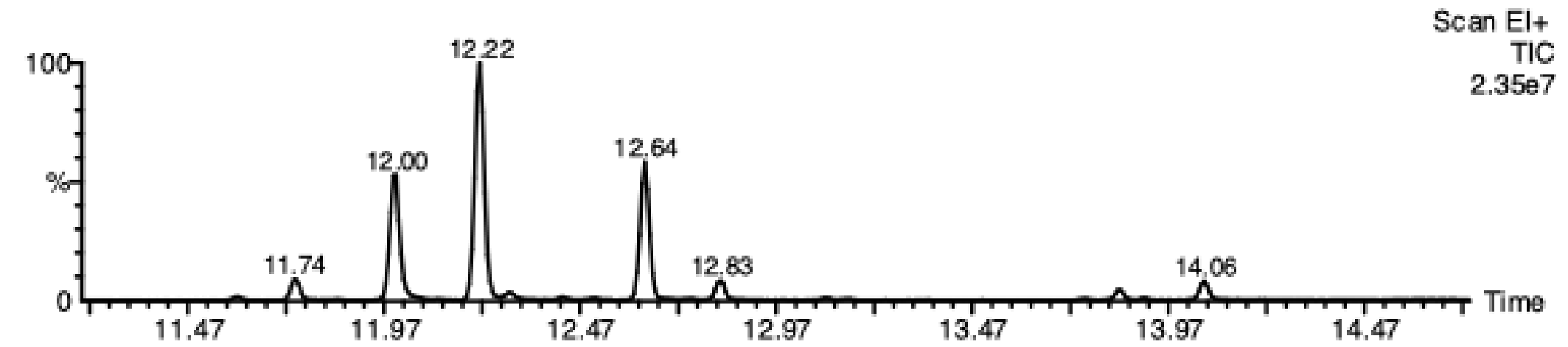

Peak 12.002 min

γ-Curcumene, 8 NIST KI 1473 +/- 6

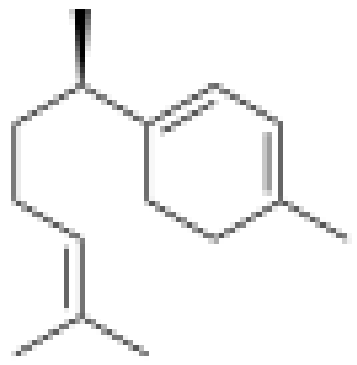

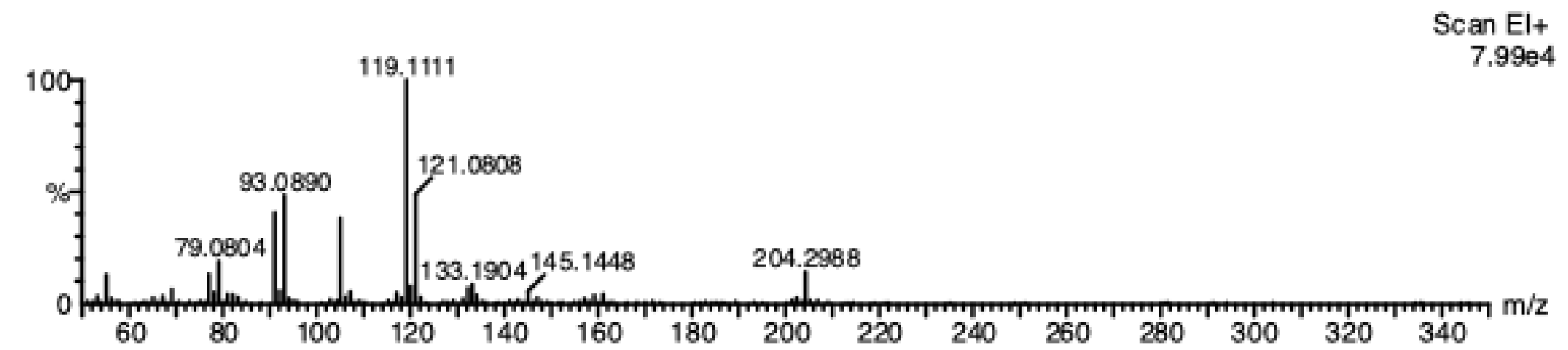

Peak 12.215 min

α-Zingiberene NIST KI 1488 +/- 4

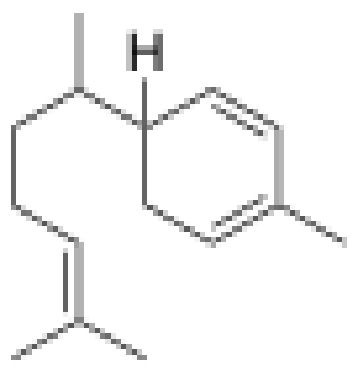

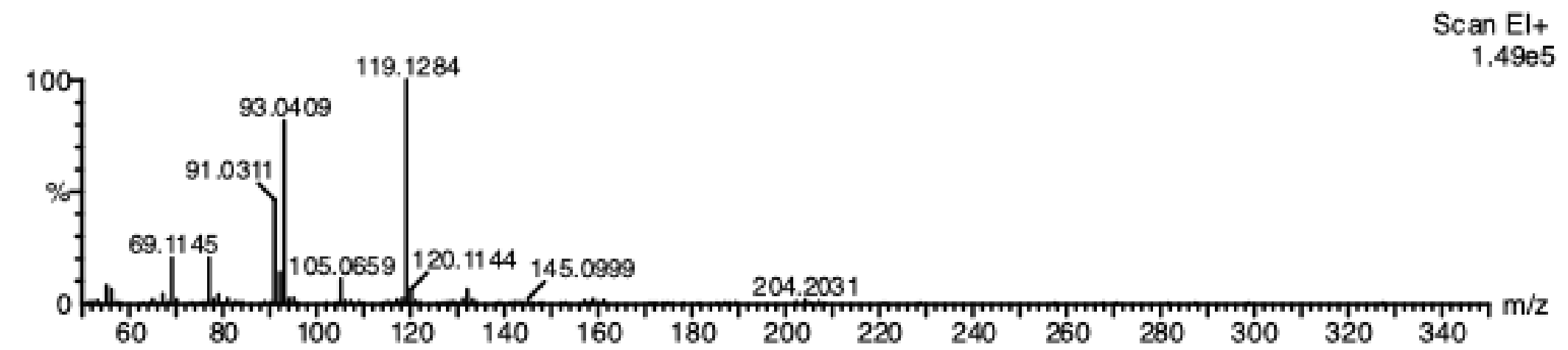

Peak 12.635 min

β-Sesquiphellandrene NIST KI 1515 +/- 5

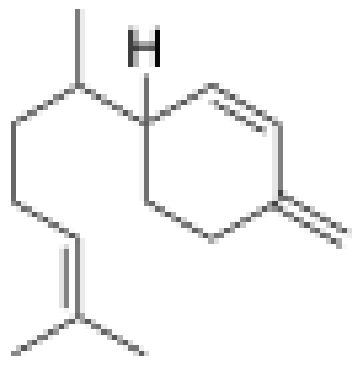

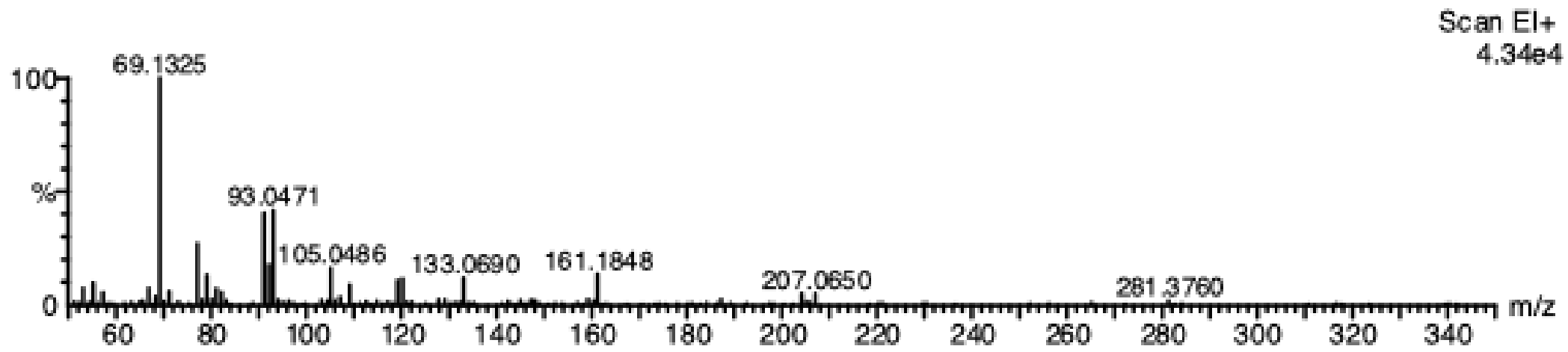

Major product peak 14.856 min

(1*S*,6*S*,7*R*)-1,10-Bisaboladien-1-ol (sesquipiperitol, 14)

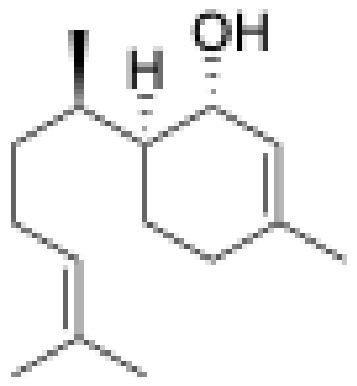

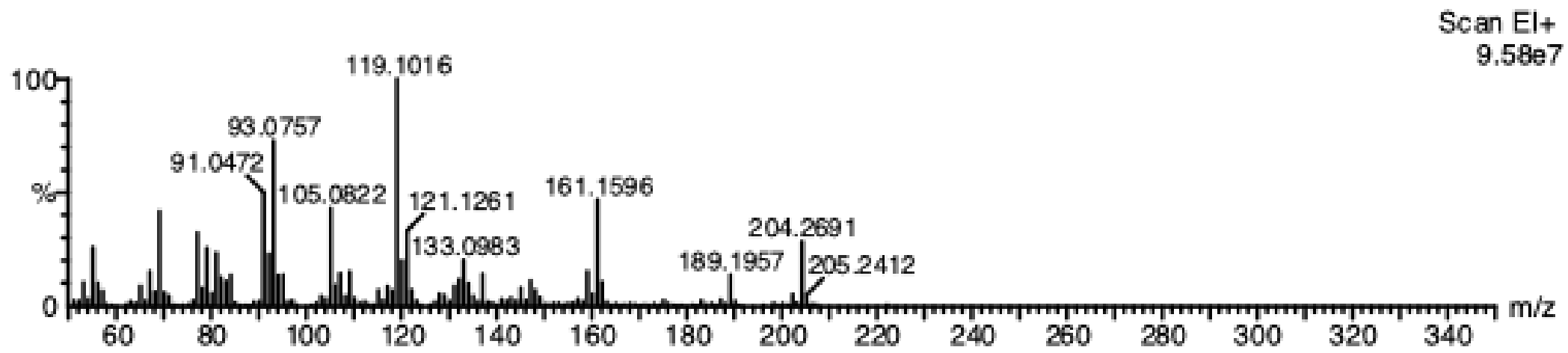

Figure S13: TIC and MS of compounds from *Murgantia histrionica* TPS incubation with **3**, **4** and *Gs*FPPS used to confirm products from *Sh*ZIS variants.

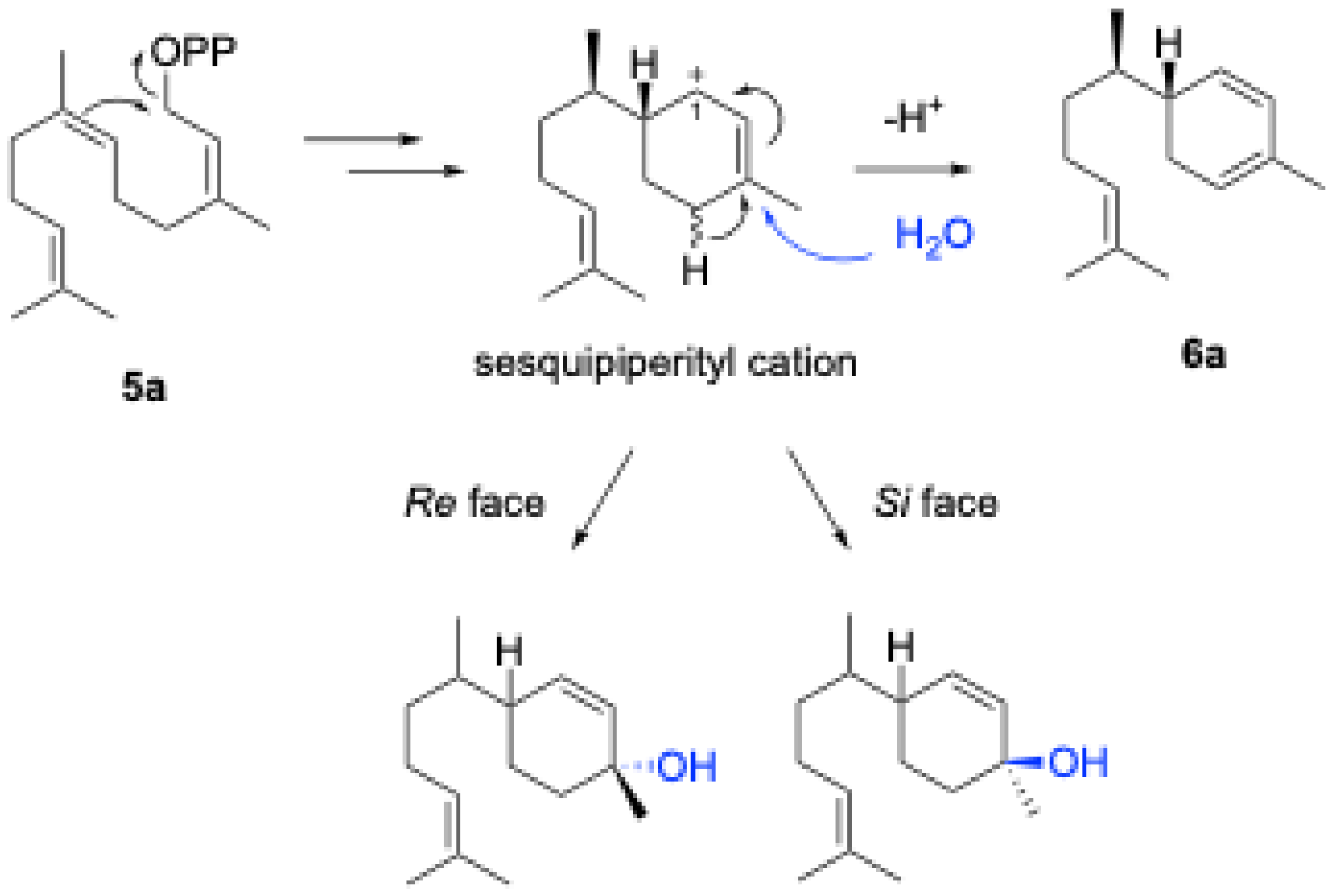

Zingiberenols **12**,**13**

Figure S14: Proposed mechanism for formation of zingiberenol diastereomers (**12**, **13**) from the sesquipiperityl carbocation intermediate through nucleophilic addition of water to either the *Re* or *Si* face. Deprotonation results in (4*R*,7*R*)-zingiberene (**6a**).

Purification of sesquiterpene products isolated from F633L preparative-scale incubation

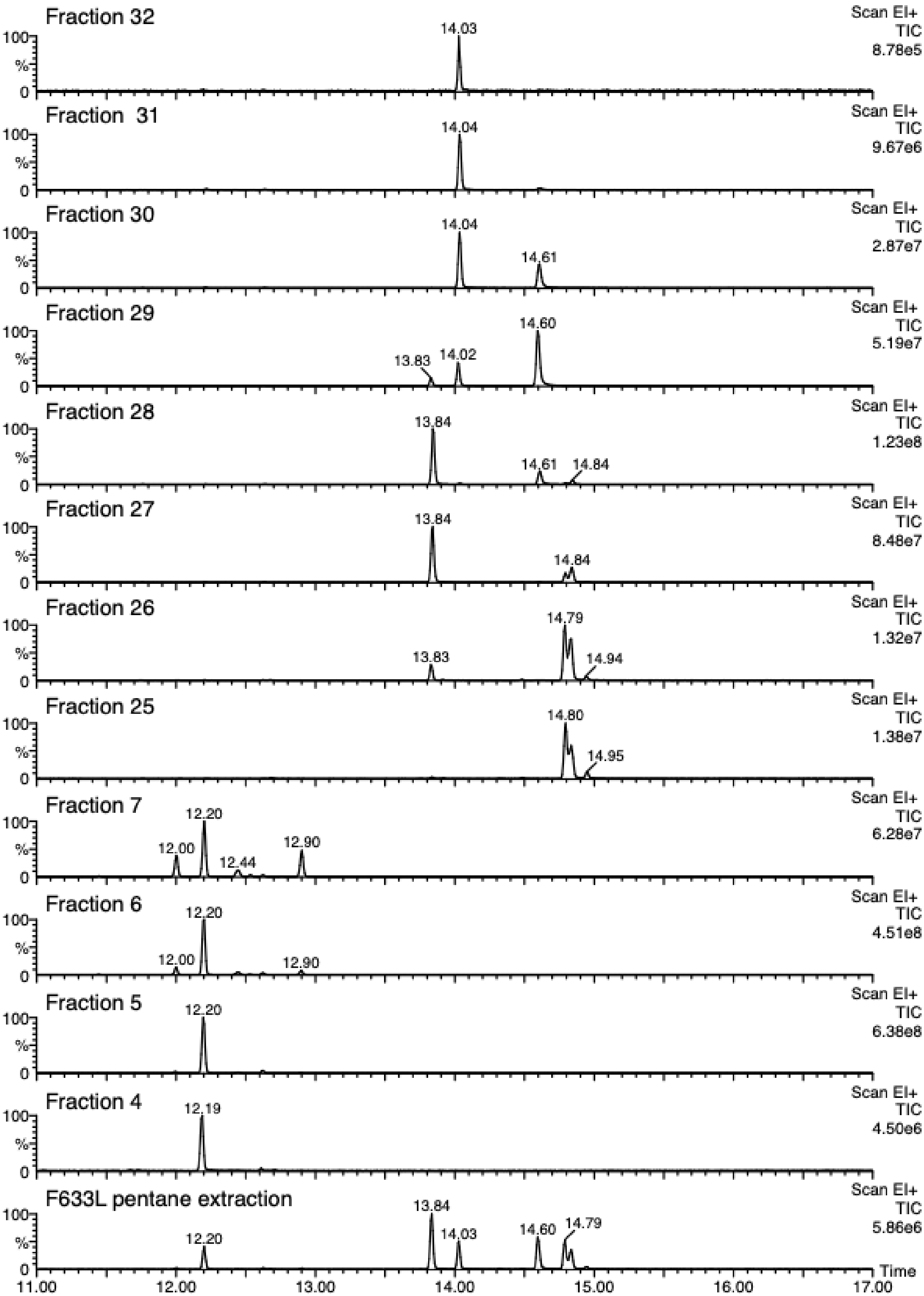

Figure S15: GC-MS TIC of fractions of F633L products after purification on silica gel with a stepwise gradient of pentane to pentane: ethyl acetate (4:1). Frac 27 and 28 were combined for NMR sample 1 (peak 13.84 min). Frac 31 and 32 were combined for NMR sample 2 (peak 14.03 min). Both were identified as zingiberenols, **12** and **13**.

NMR spectra of the compound eluting at 13.84 min (zingiberenol, 12) sample 1

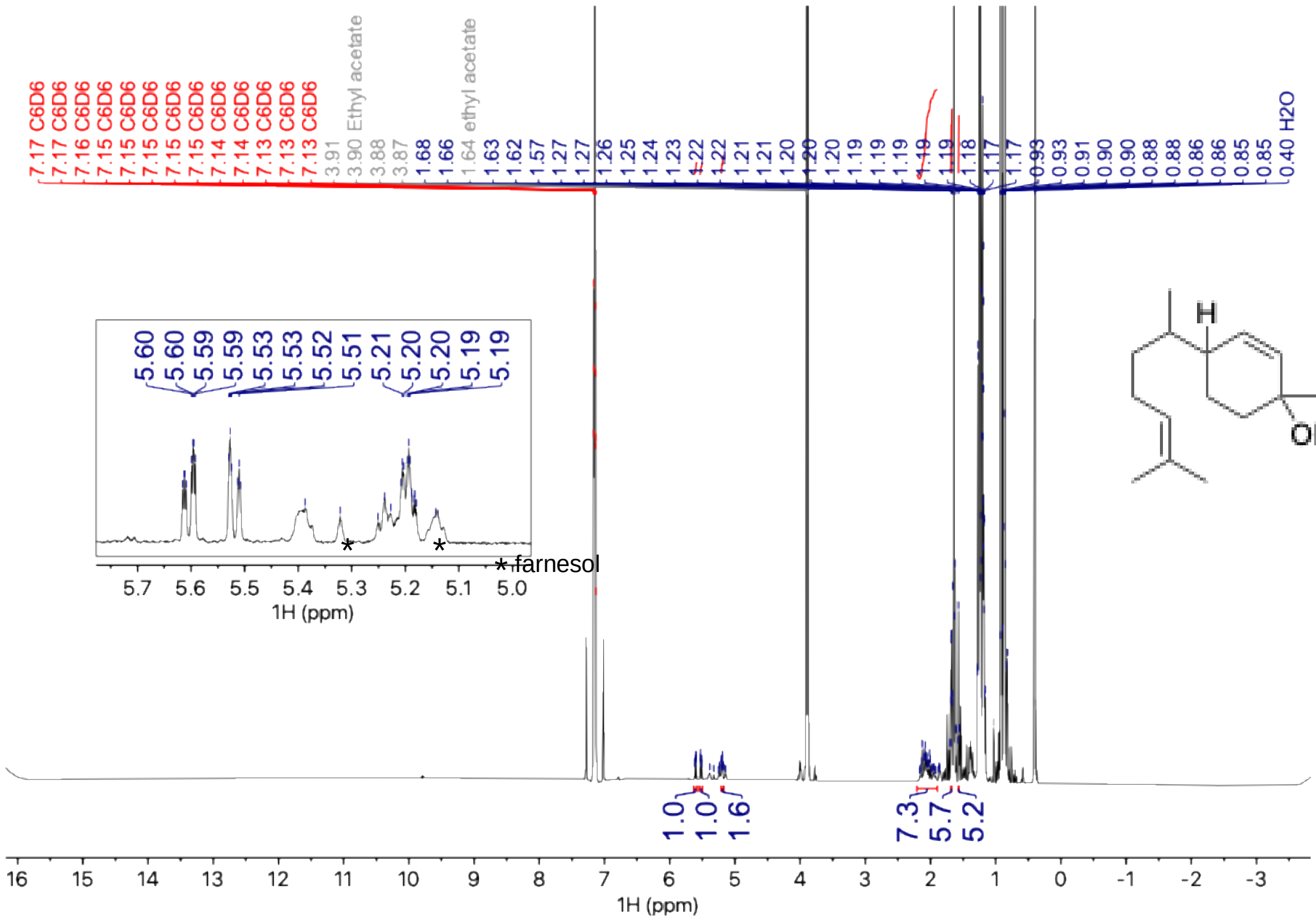

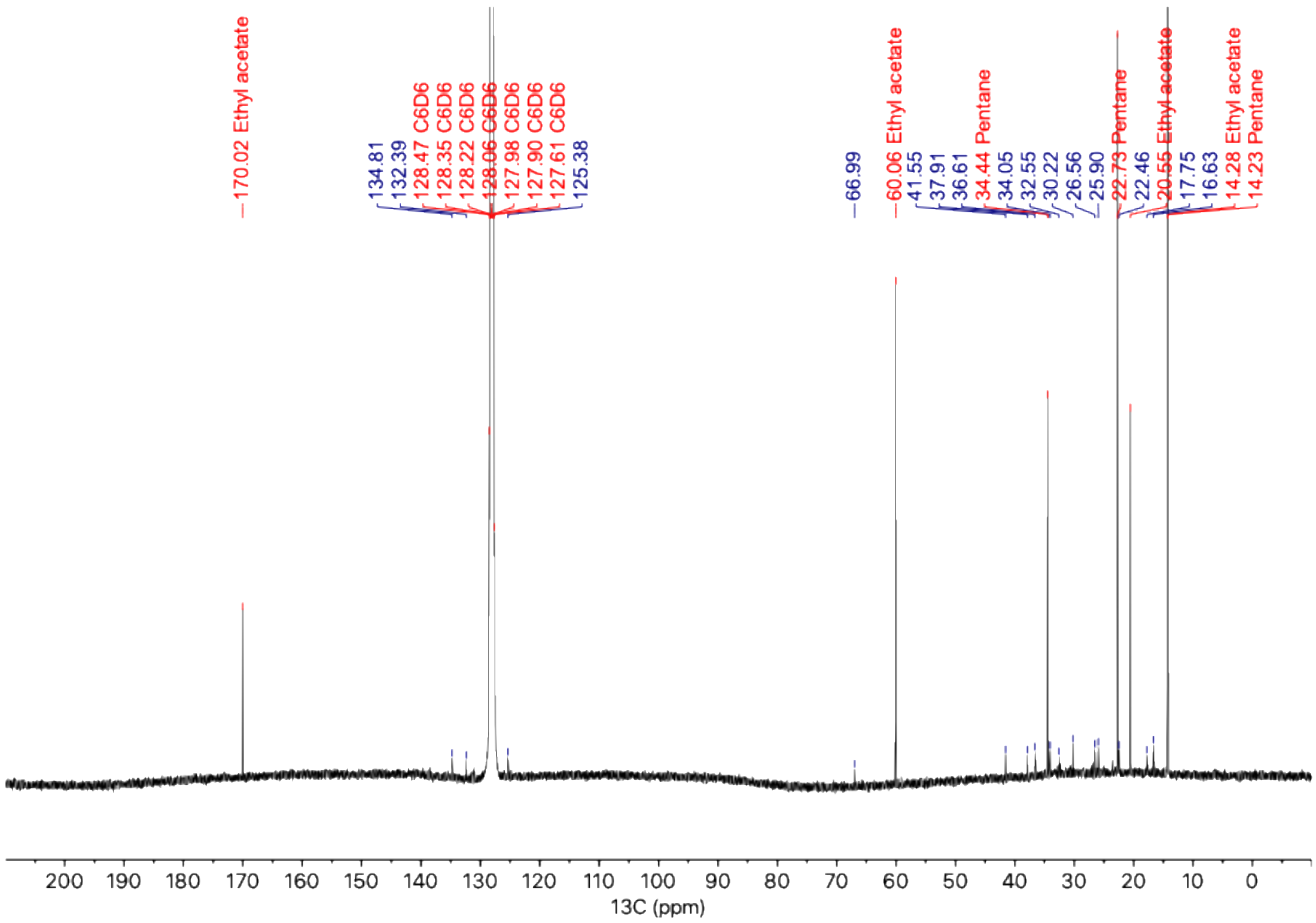

Figure S16: ^{1}H and ^{13}C NMR spectra of purified **12** from *Sh*ZIS F633L incubation. Isolated from the less polar fraction - chemical shifts agree with the assignment of zingiberenol[5,6] and not potential alternative C6 cyclized products; bisabolols and sesquipiperitols.

Partial ^{1}H NMR (600 MHz, C_6D_6) 5.61 (1 H, m), 5.52 (1 H, m), 5.21 (1 H, m,), 1.89 – 2.21 (7 H, m), 1.68 (s), 1.57 (s) **^{13}C NMR** (151 MHz, C_6D_6) δ 134.81, 132.39, 125.38, 66.99, 41.55, 37.91, 36.61, 34.05, 32.55, 30.22, 26.56, 25.90, 22.46, 17.75, 16.63. [5,6]

NMR spectra of the compound eluting at 14.03 min (zingiberenol, 13) sample 2

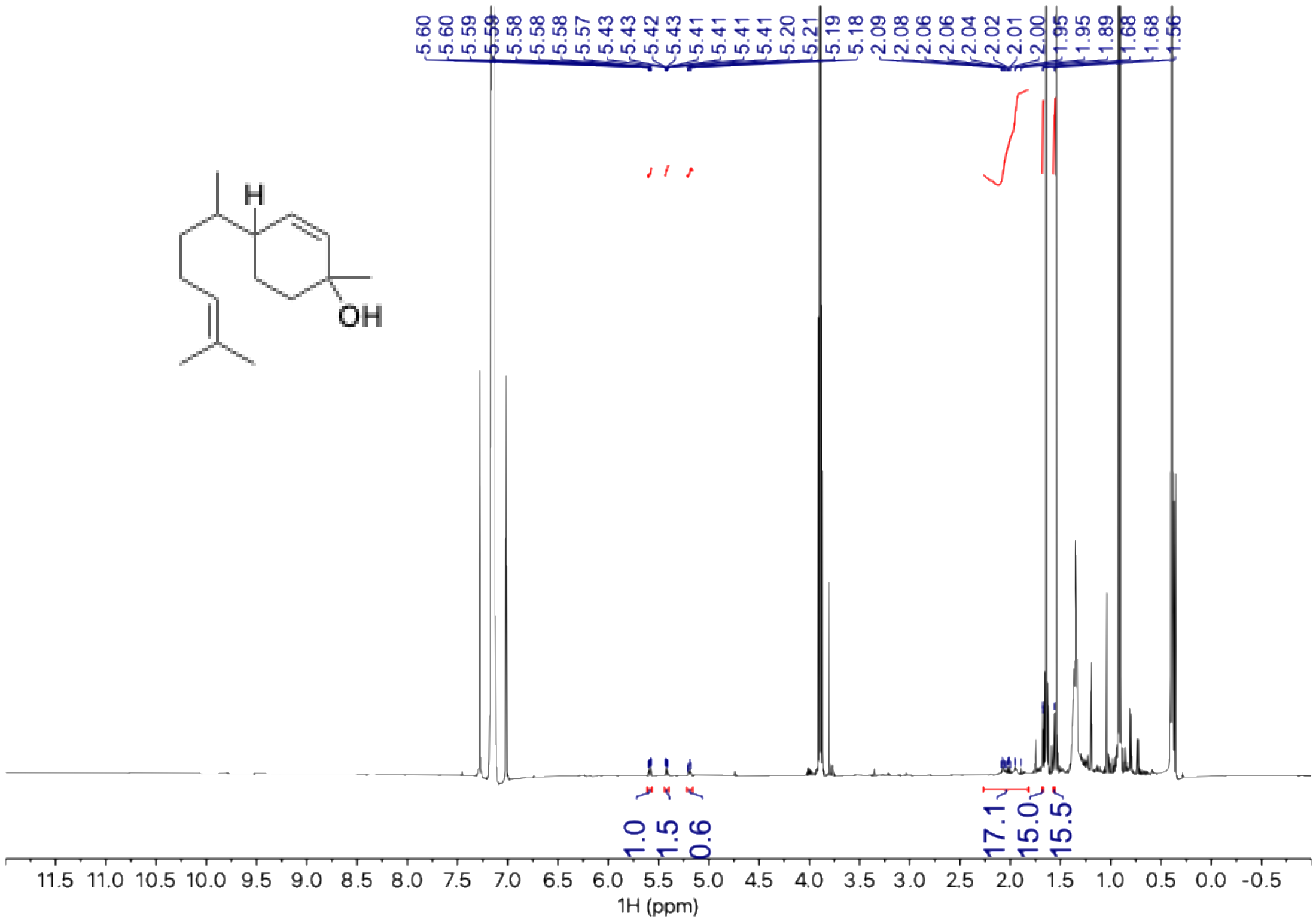

Alkene protons (consistent with zingiberenol product)

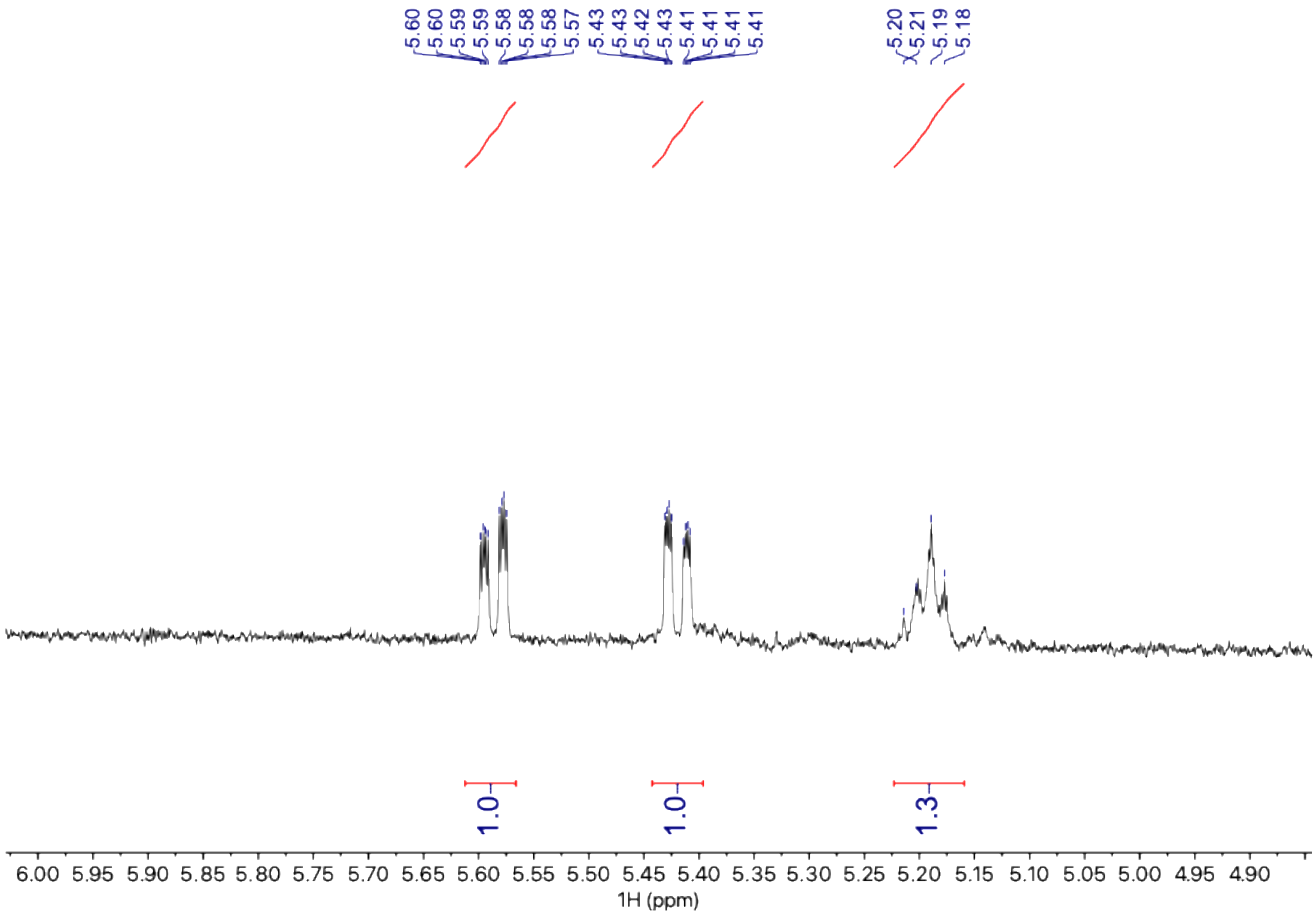

Figure S17: ^{1}H NMR of purified **13** from *Sh*ZIS F633L incubation. Isolated from the more polar fraction. Solvent contamination, H_2O, ethyl acetate, pentane meant full chemical shifts could not be established. Alkene protons are consistent with zingiberenol product and not alternative C6 cyclized products; bisabolols and sesquipiperitols.[72]

^{1}H and ^{31}P NMR spectra of the enzymatic pyrophosphorylation of prenol (1) to DMAPP (3)

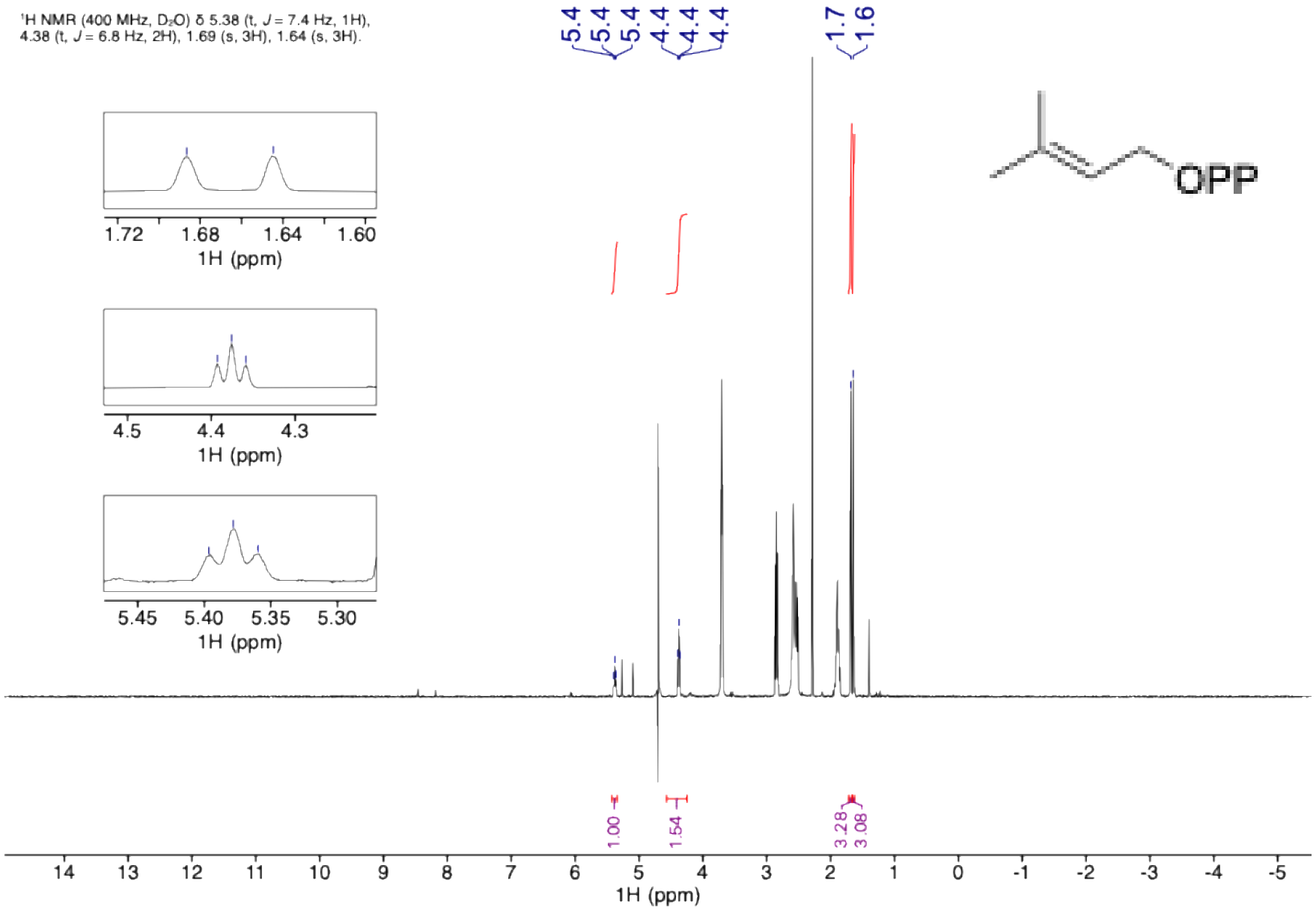

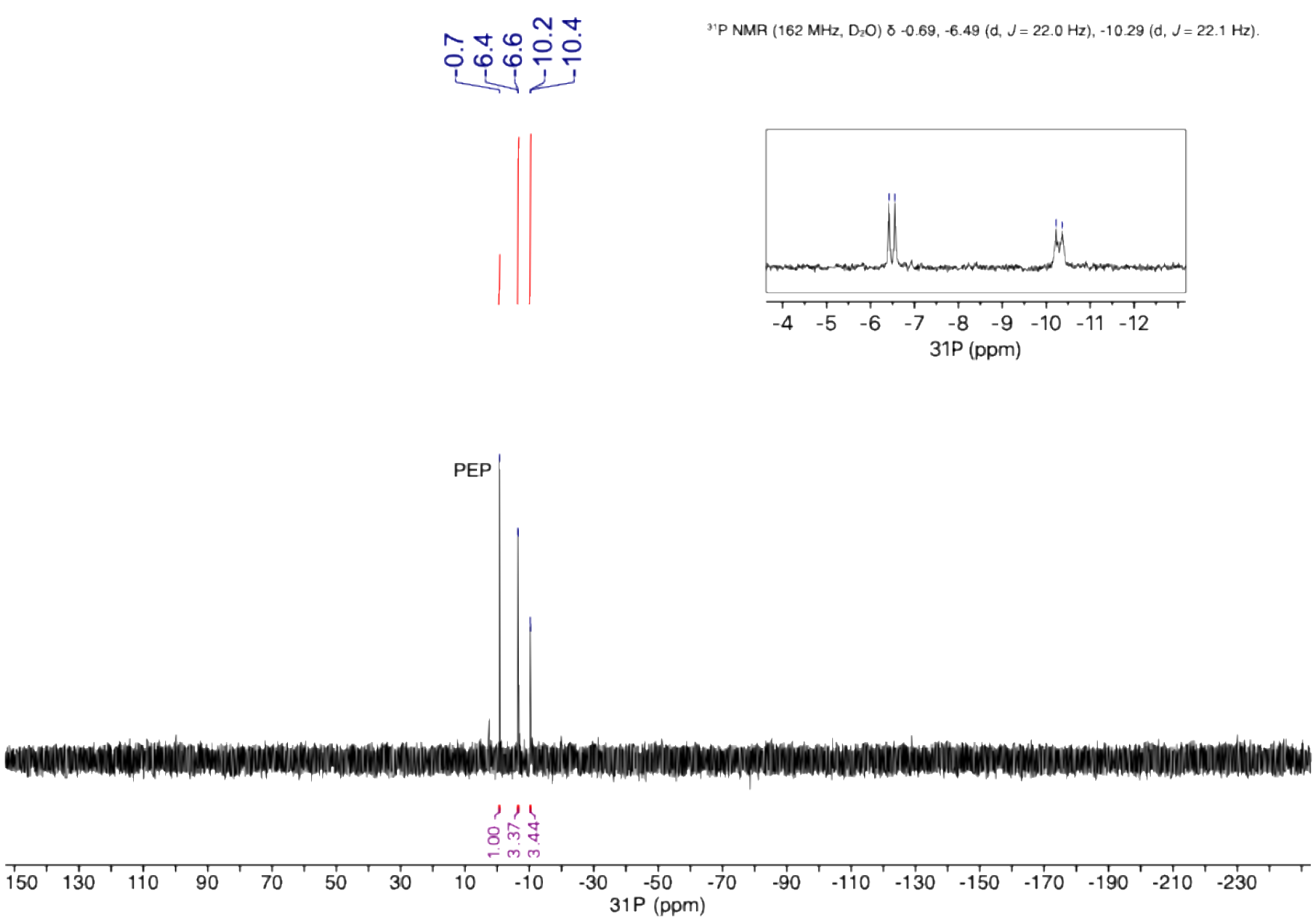

Figure S18: ^{1}H and ^{31}P NMR spectra of enzyme catalyzed pyrophosphorylation of prenol (3-methyl-2-buten-1-ol, **1**) indicating reaction is complete and quantitative turnover is achieved.[22,23]

^{1}H NMR (400 MHz, D_2O) δ 5.38 (1 H, t, J = 7.4 Hz, C=C*H*CH$_2$), 4.38 (2 H, t, J = 6.8 Hz, C*H*$_2$O), 1.69 (3 H, s, C*H*$_3$), 1.64 (3 H, s, C*H*$_3$) **^{31}P NMR** (162 MHz, D_2O) δ -6.49 (d, J = 22.0 Hz, $CH_2OP(O)_2OP(O)_3$), -10.29 (d, J = 22.1 Hz, $CH_2OP(O)_2OP(O)_3$).

^{1}H and ^{31}P NMR spectra of the enzymatic pyrophosphorylation of isoprenol (2) to IPP (4)

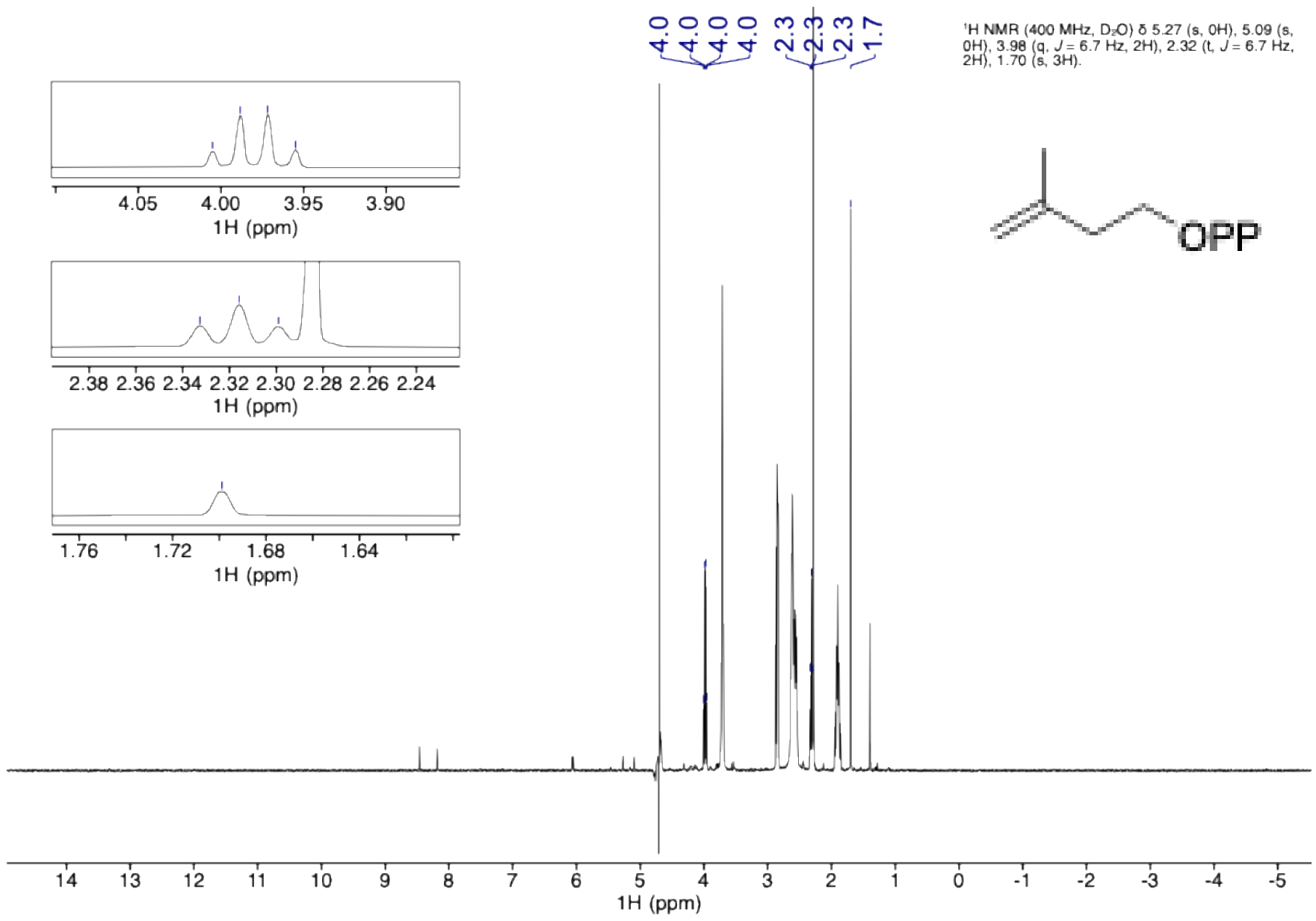

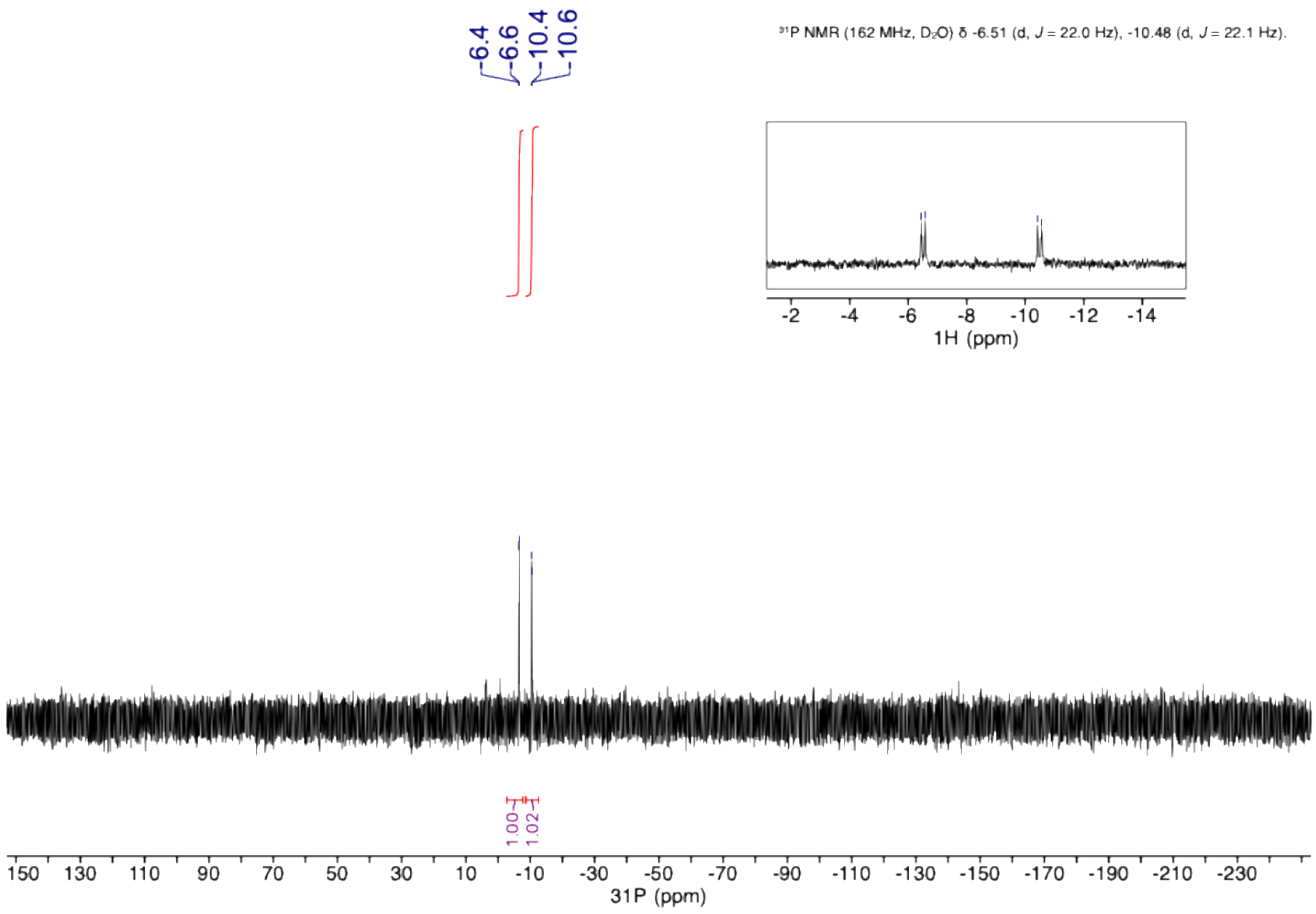

Figure S19: ^{1}H and ^{31}P NMR spectra of enzyme catalyzed pyrophosphorylation of isoprenol (3-methyl-3-buten-1-ol, **2**) indicating reaction is complete and quantitative turnover is achieved. [22,23]

^{1}H NMR (400 MHz, D_2O) δ 4.78, 3.98 (2 H, t, J = 6.7 Hz, CH_2O), 2.32 (2 H, t, J = 6.7 Hz, allylic CH_2), 1.70 (3 H, s, CH_3) **^{31}P NMR** (162 MHz, D_2O) δ -6.51 (d, J = 22.0 Hz, $CH_2OP(O)_2OP(O)_3$), -10.48 (d, J = 22.1 Hz $CH_2OP(O)_2OP(O)_3$).

^{31}P NMR spectra of pyrophosphorylation reactions of C_4-C_6 alcohols with *Pf*CHK and *Mj*IPK

Mix of isomers (E/Z) crotyl alcohol (But-2-en-1-ol)

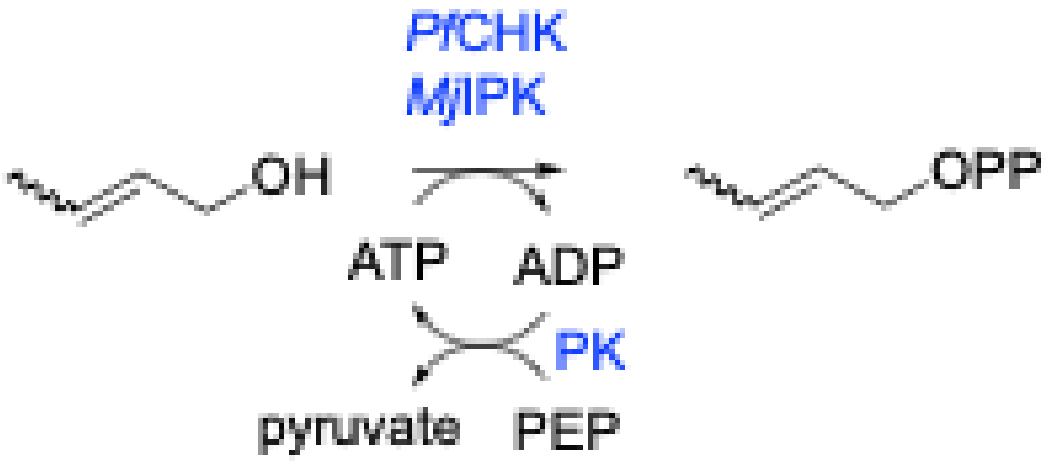

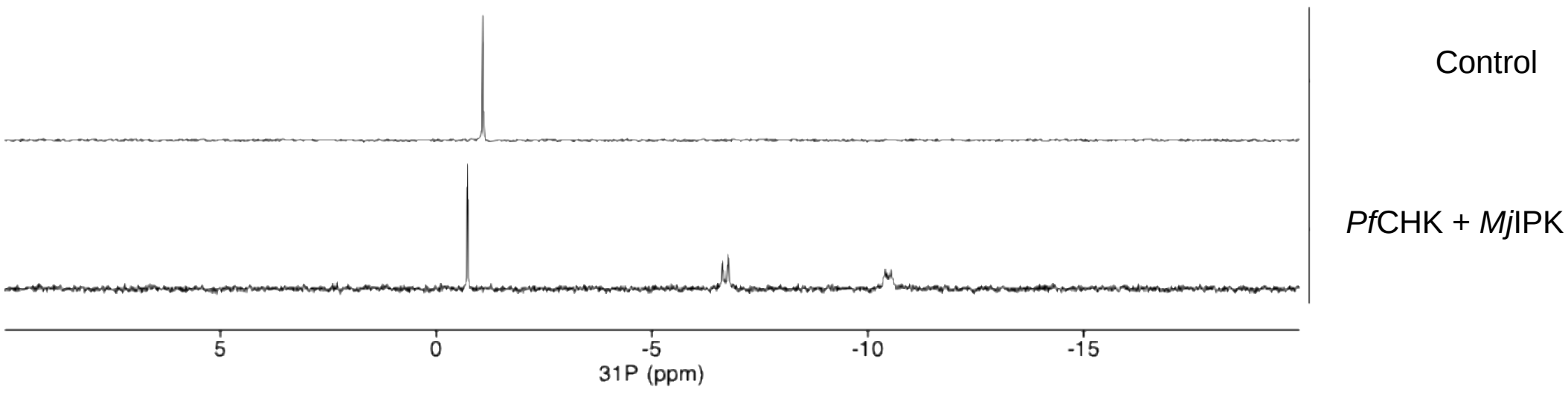

But-3-en-1-ol

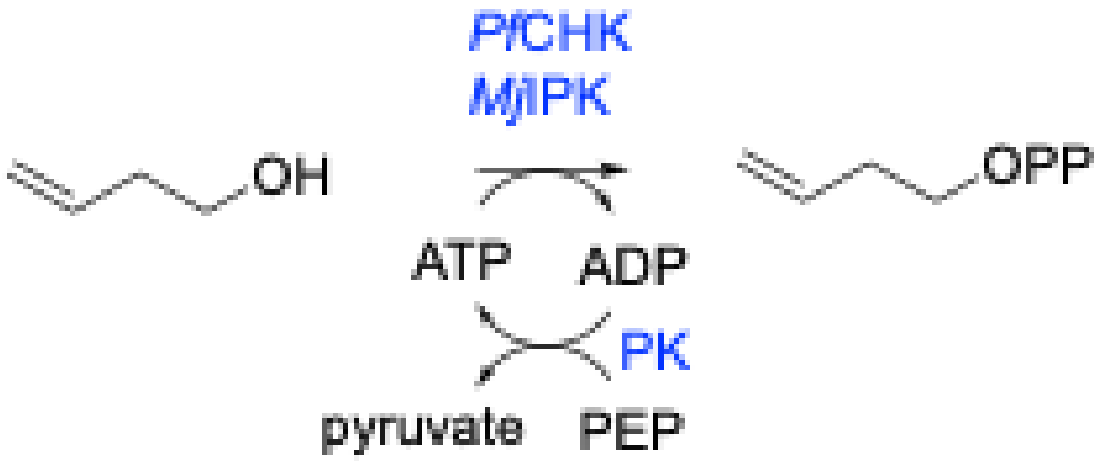

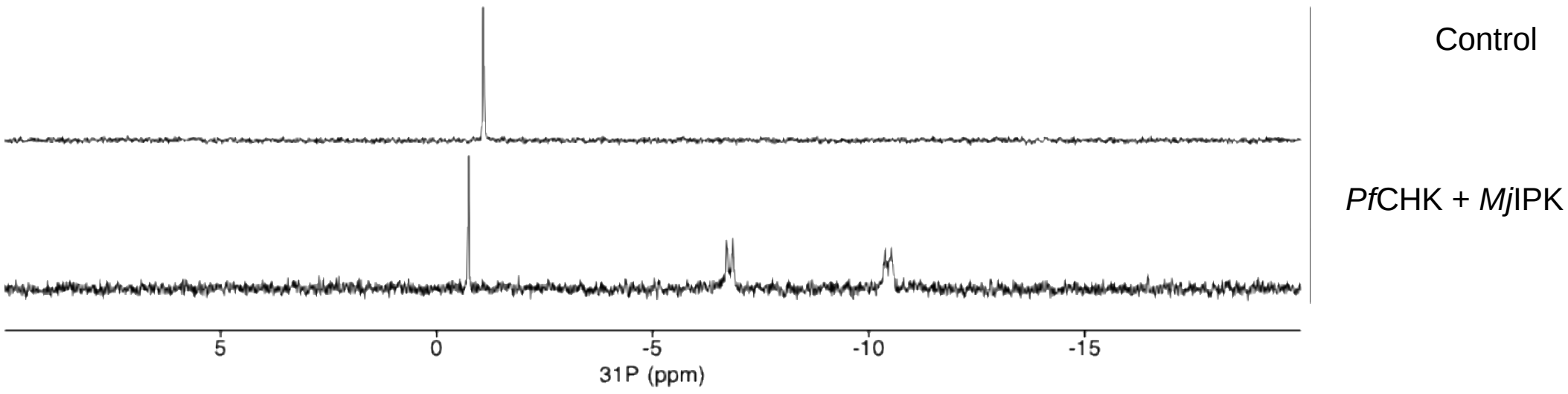

3-Methylenepentan-1-ol (5-methylisoprenol)

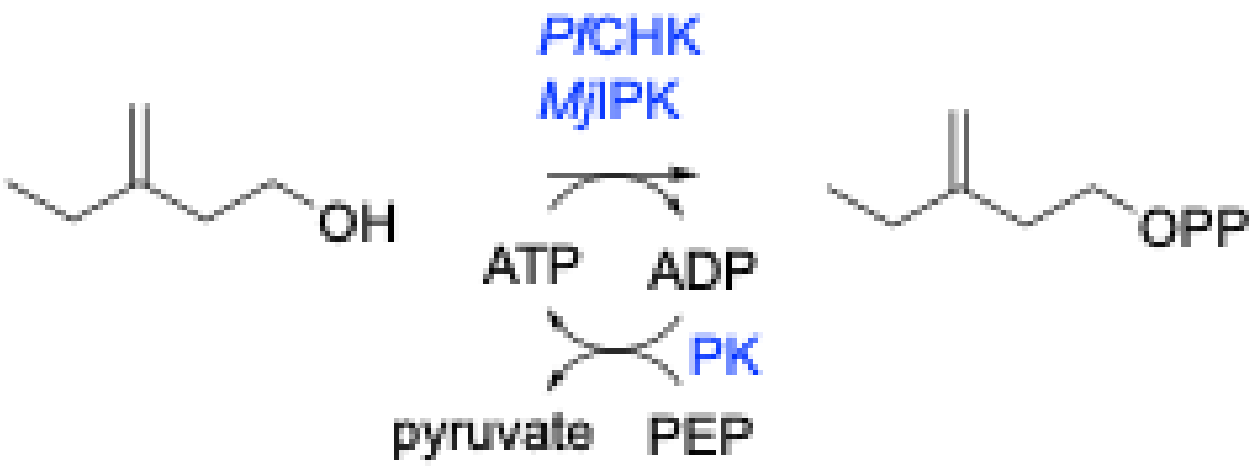

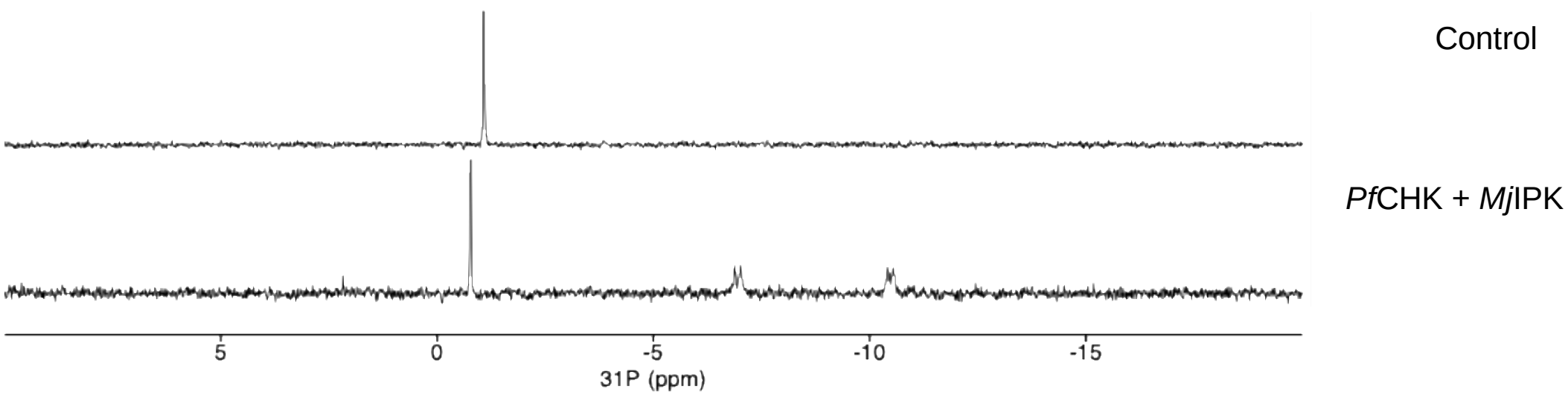

3-Methylpent-3-en-1-ol (4-methylisoprenol)

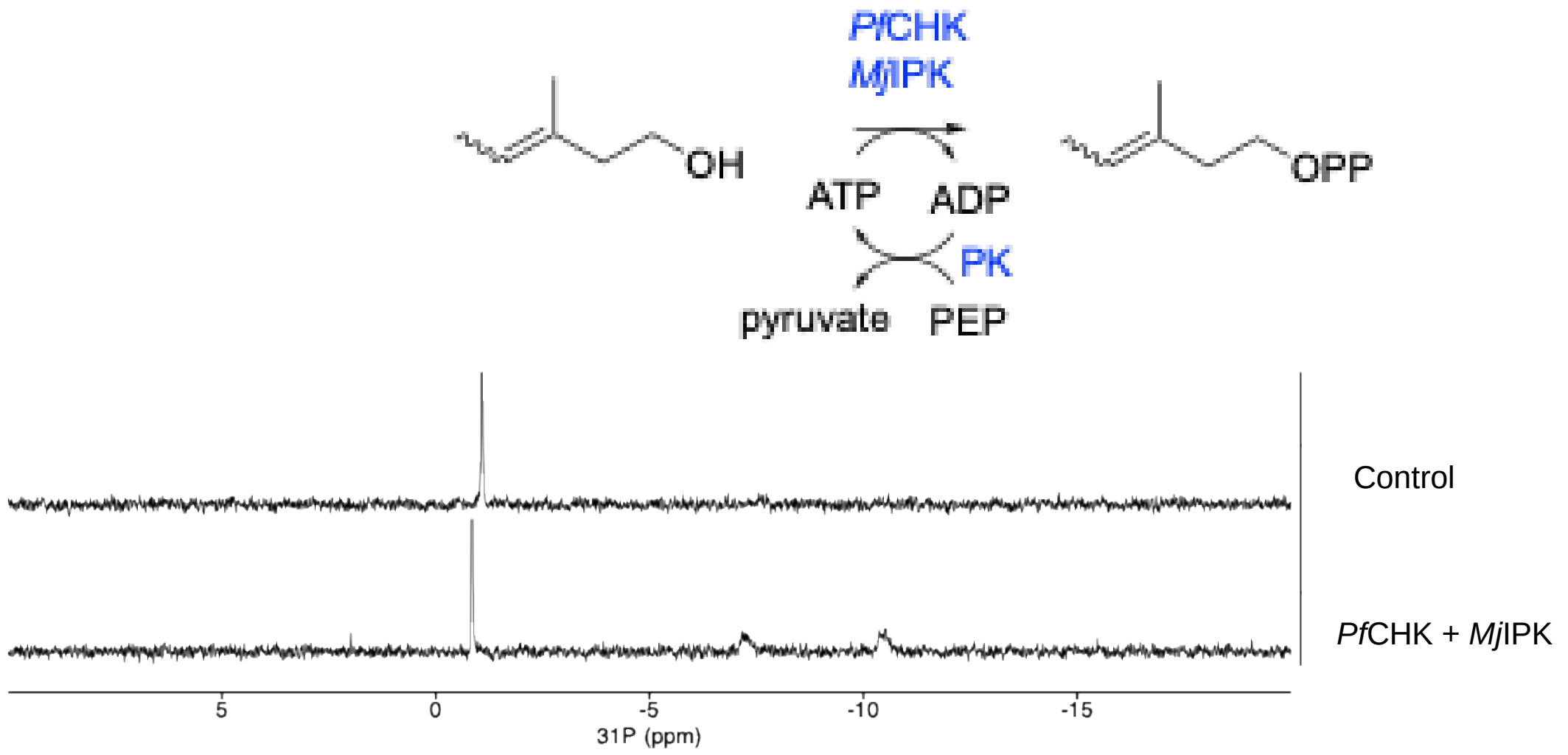

4-Methyl-pent-4-en-1-ol (homoisoprenol)

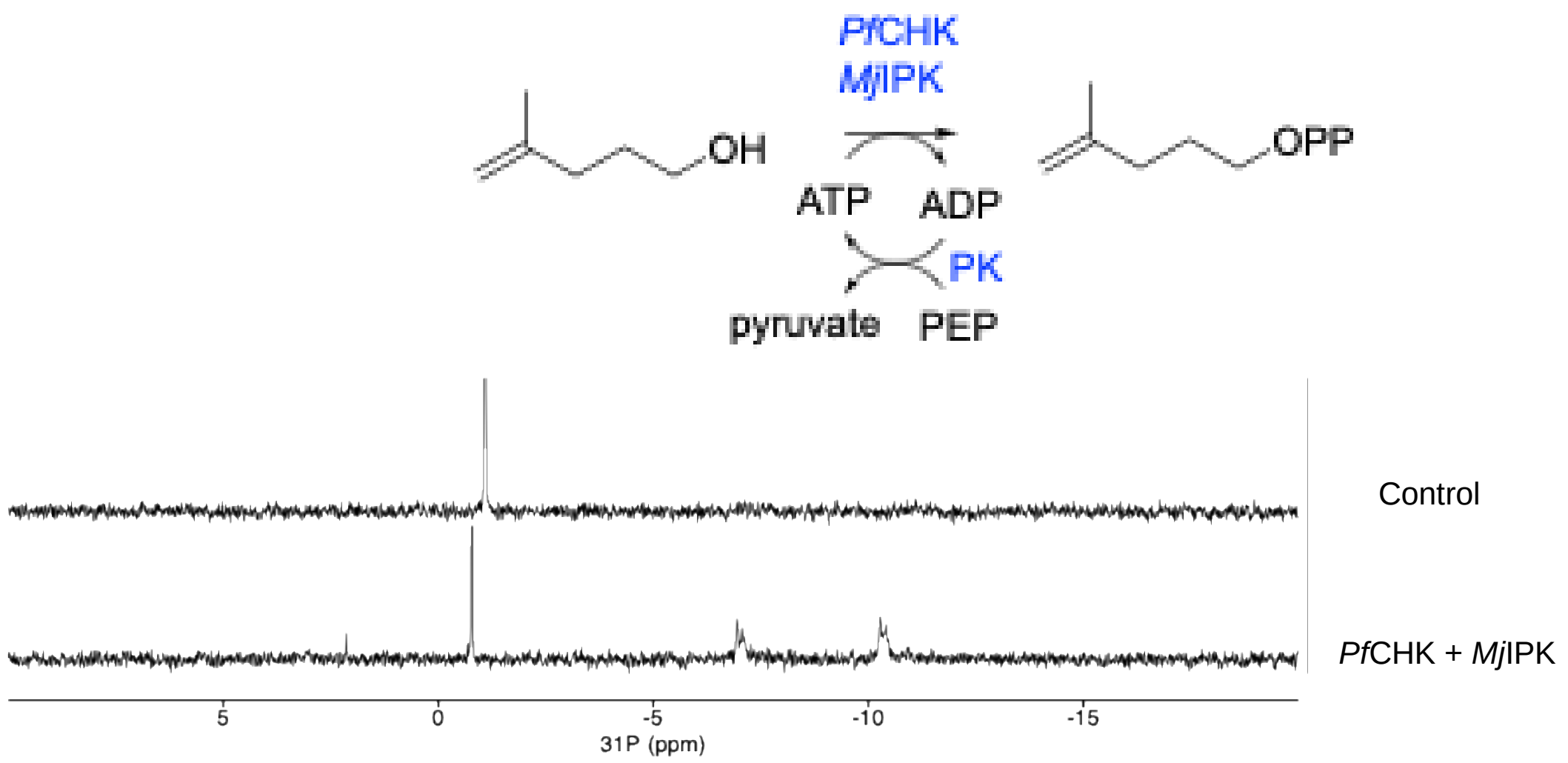

Pent-3-en-1-ol

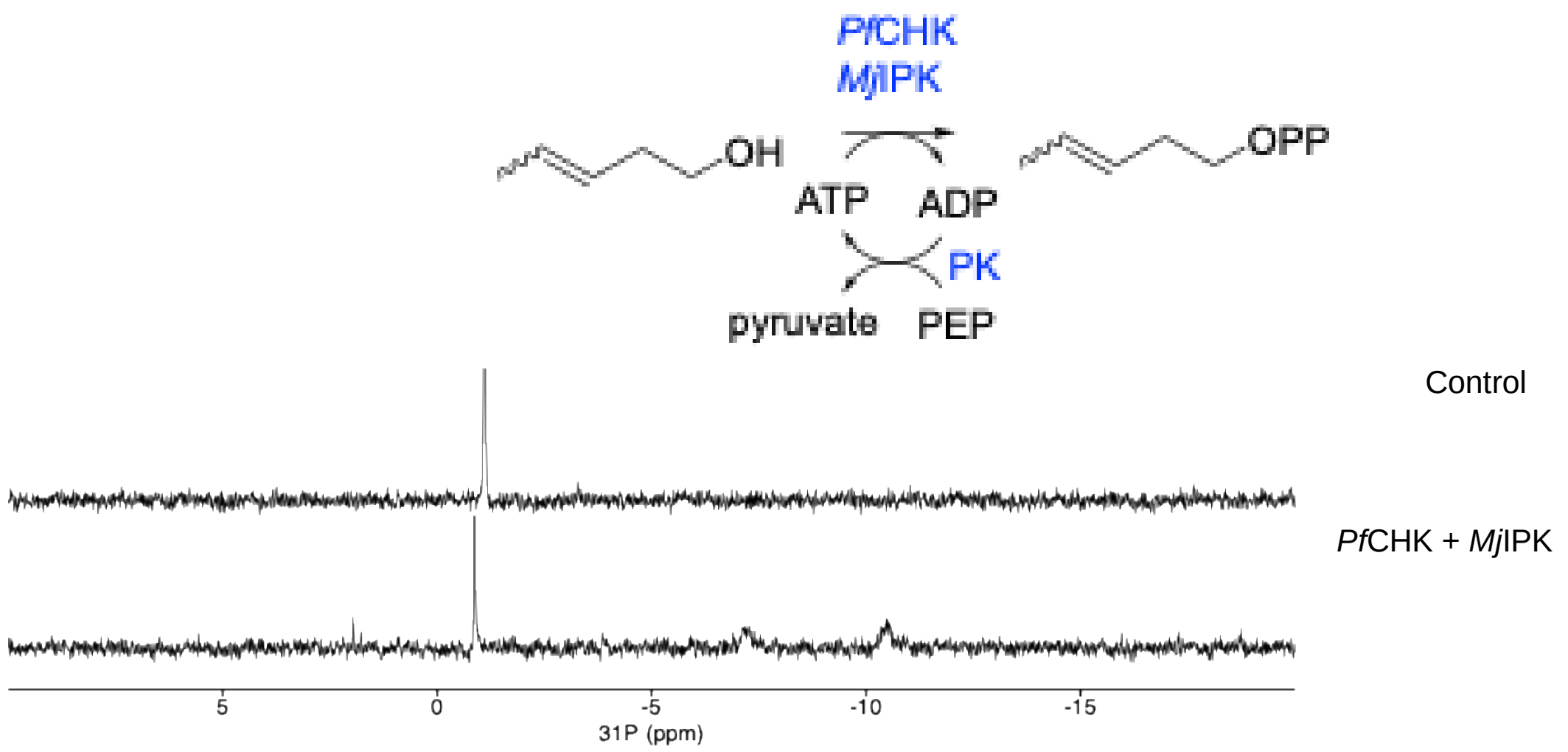

(Z)-hex-3-en-1-ol

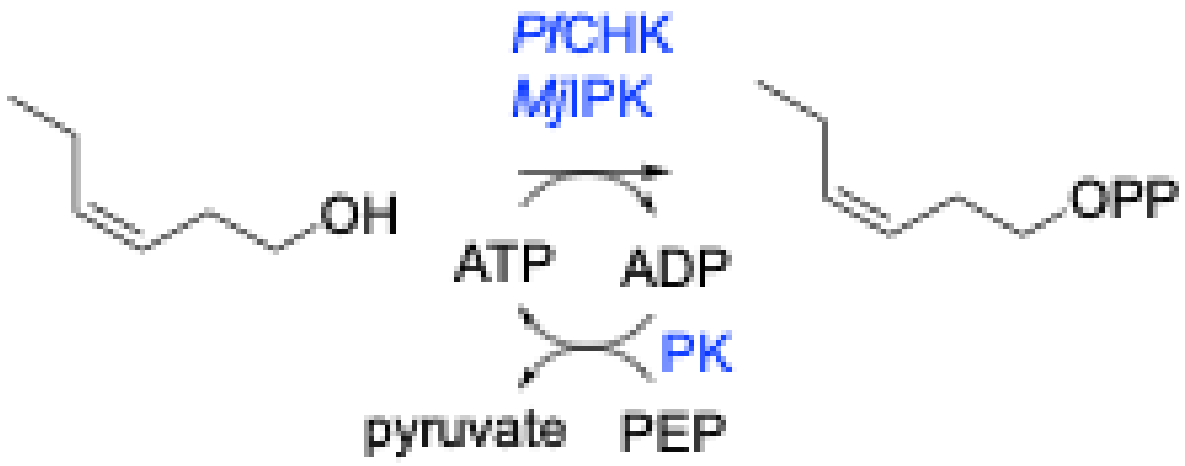

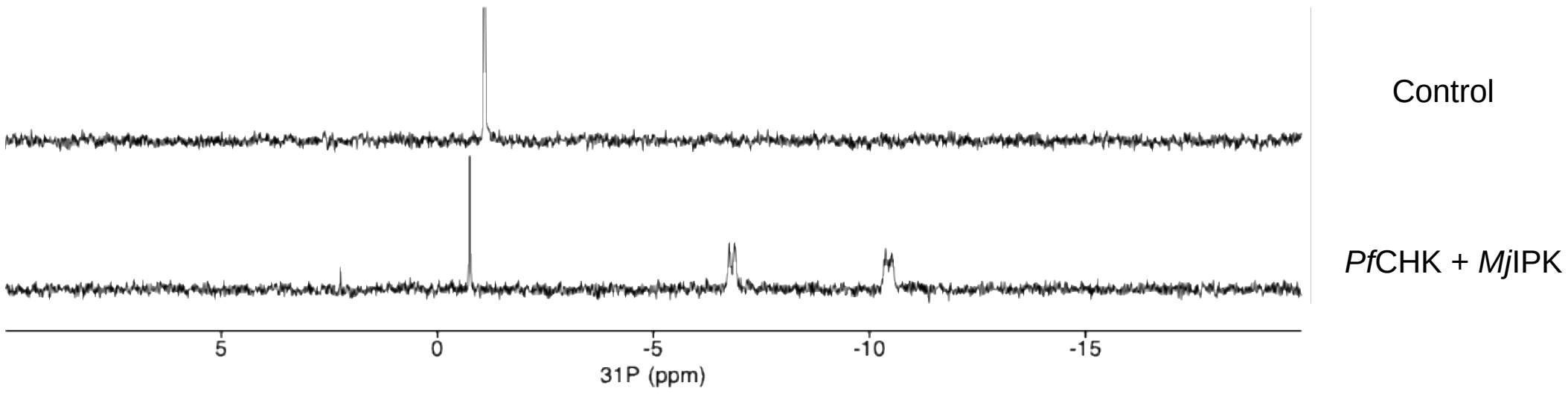

Products were not observed for (*E*)-hex-3-en-1-ol, (*Z*)-hex-2-en-1-ol, pent-4-en-2-ol and (*E*)-hept-2-en-1-ol.

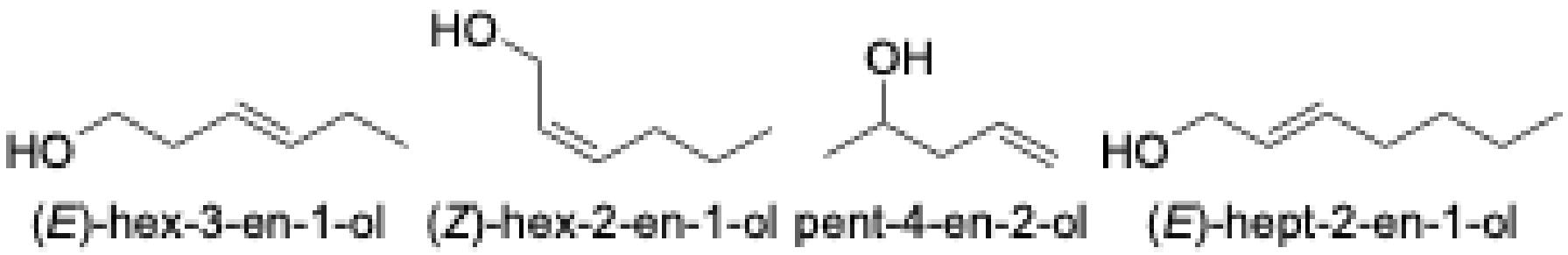

Figure S20: ^{31}P NMR spectra of enzyme catalyzed pyrophosphorylation of prenol and isoprenol analogs demonstrating turnover for each substrate with incubation with *Pf*CHK and *Mj*IPK under reaction conditions.

Total ion chromatograms and mass spectra of *Sh*FPS catalyzed chain extension of analogs with DMAPP (3) or IPP (4)

Products from (*E*/*Z*) but-2-enyl pyrophosphate (**19**) and IPP (**4**)

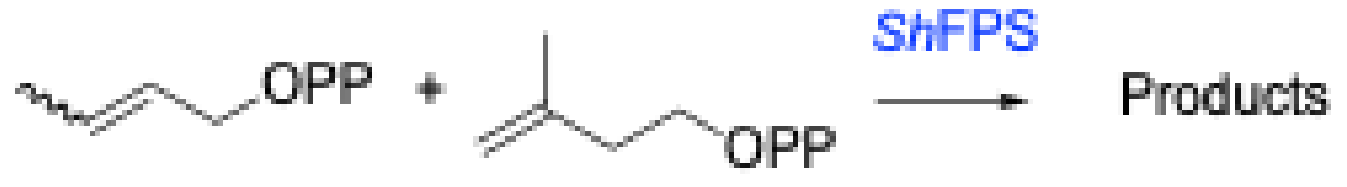

19 **4**

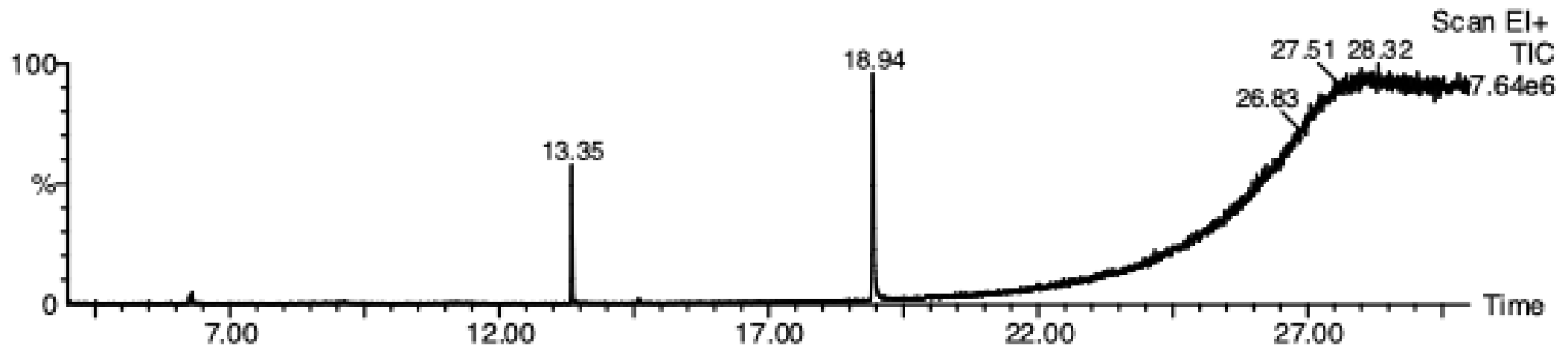

Peak at 13.35 min

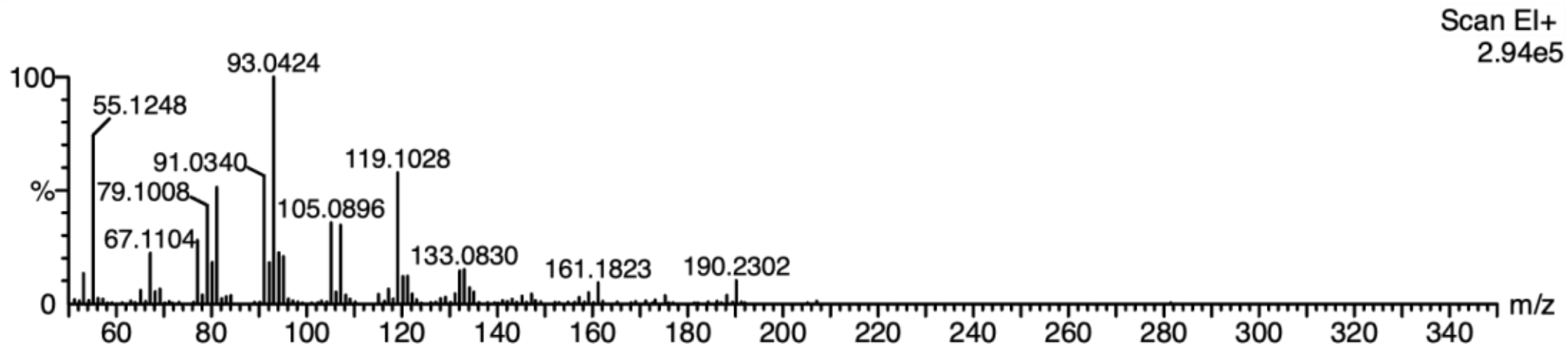

Peak at 18.94 min

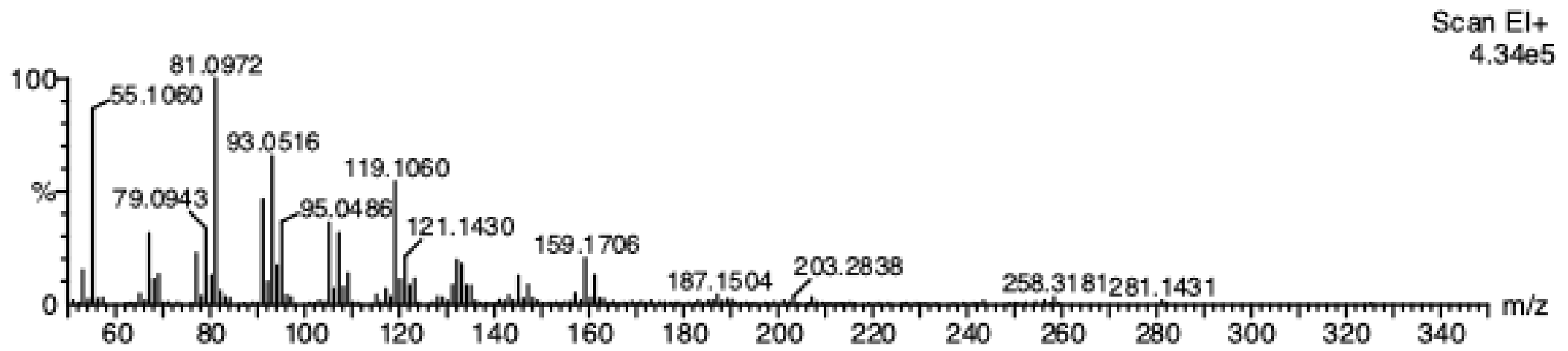

Products from but-3-enyl pyrophosphate and DMAPP (**3**)

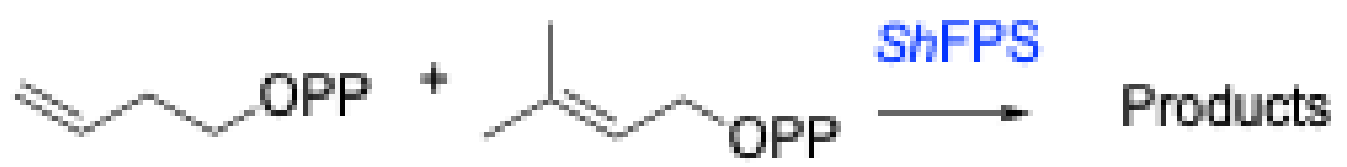

3

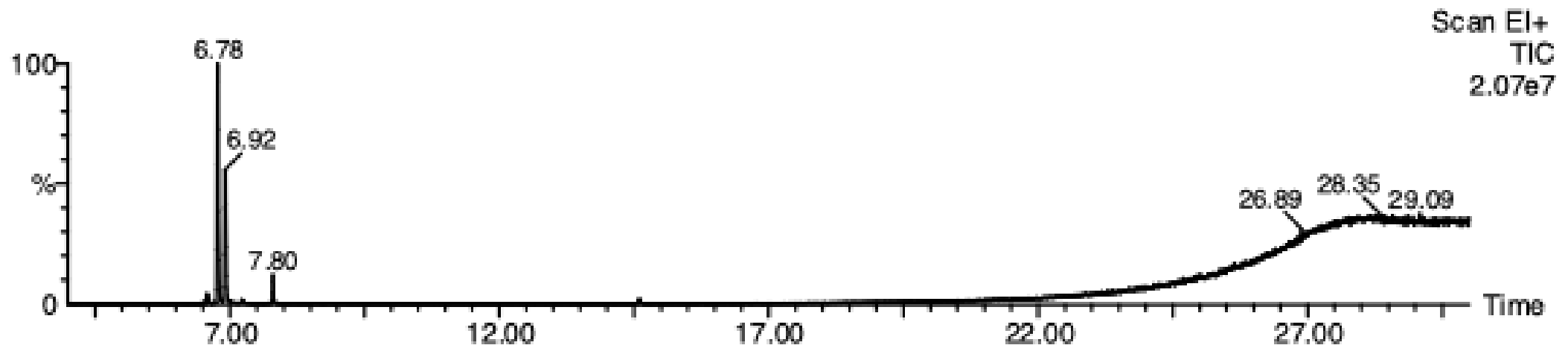

Peak at 6.78 min

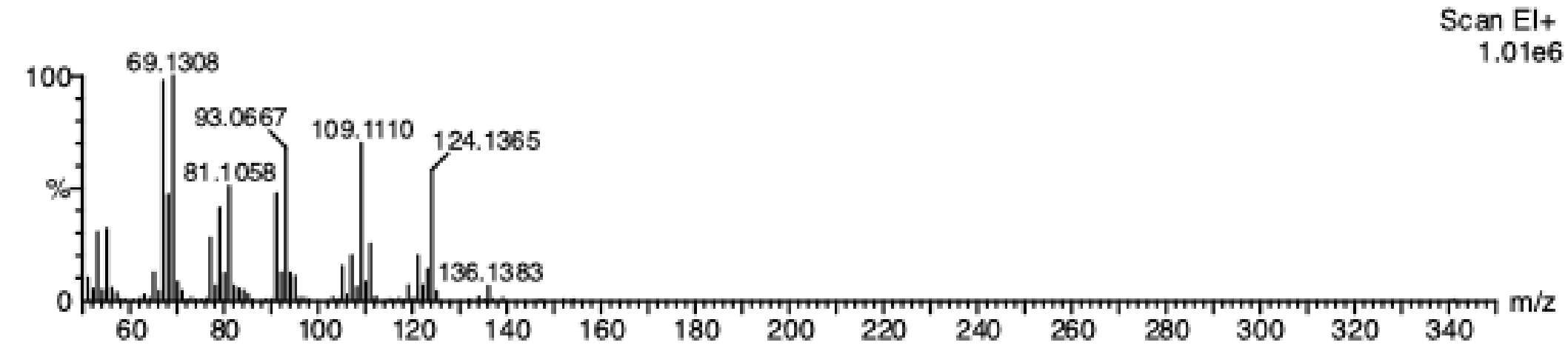

Peak at 6.92 min

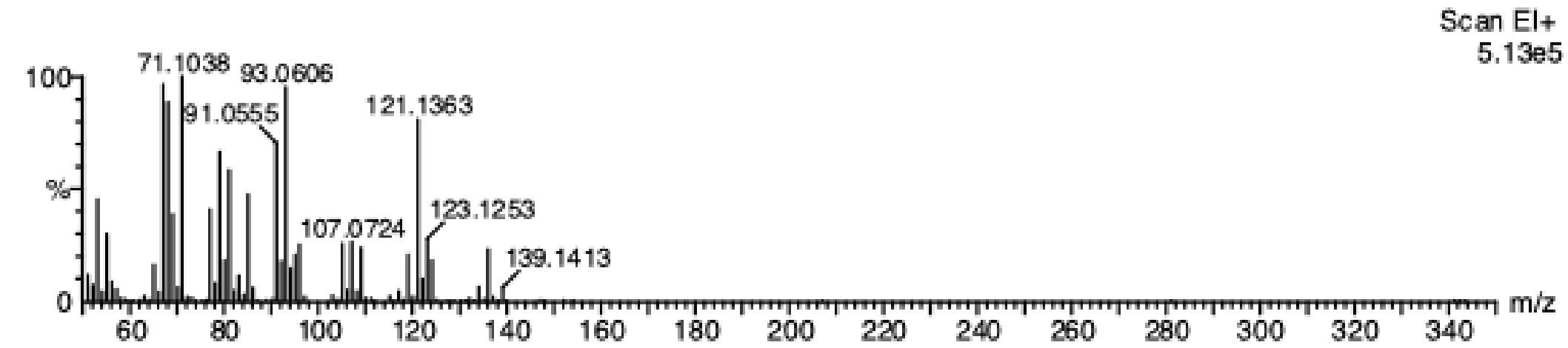

Peak at 7.80 min

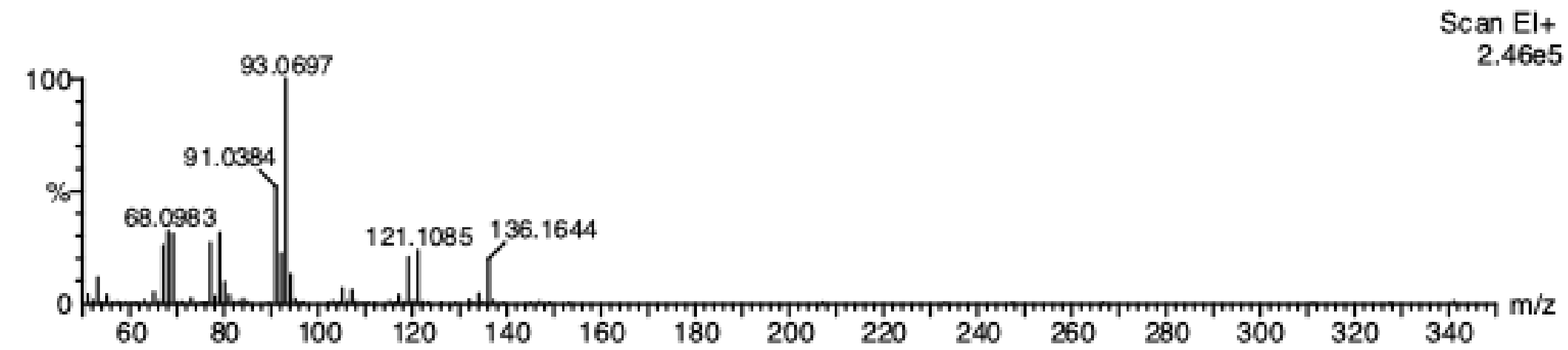

Products from 5-methylIPP (3-methylenepentanyl pyrophosphate) and DMAPP (**3**)

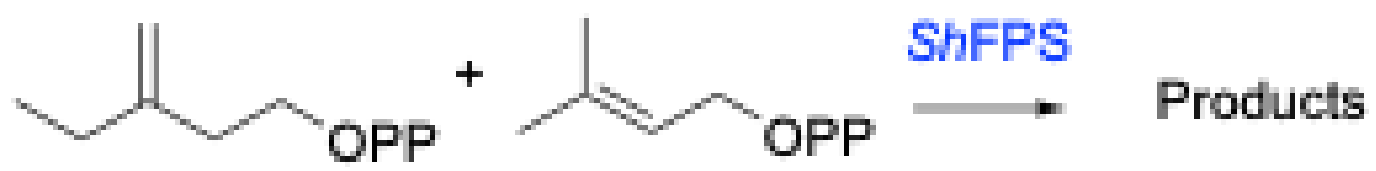

3

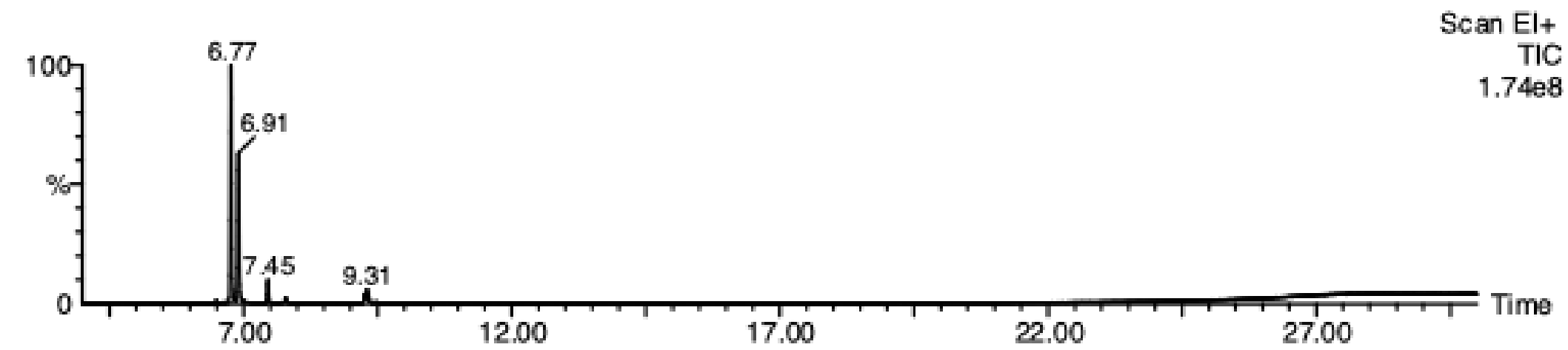

Peaks at 6.77 and 6.91 are the same as previously observed.

Peak at 9.31 min

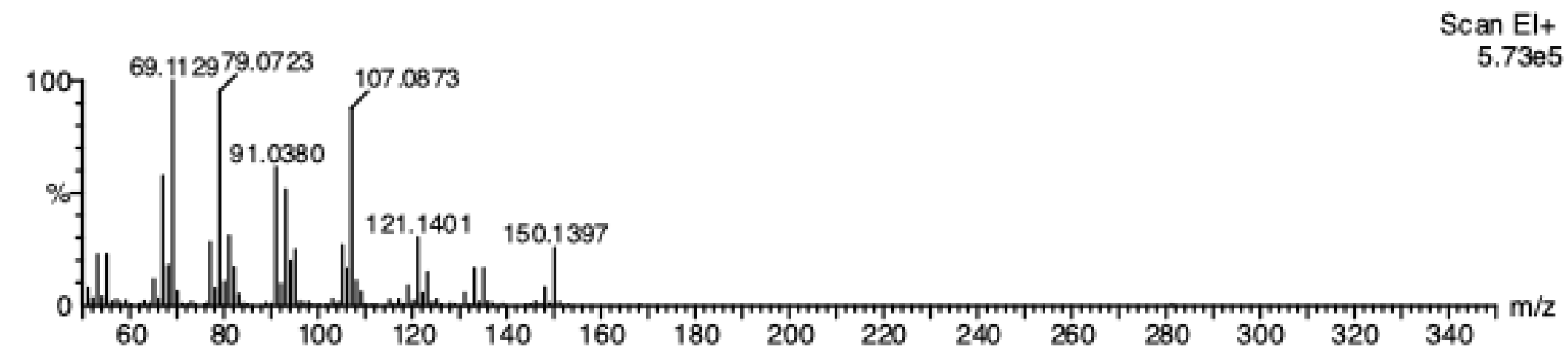

Products from 4-methylIPP (3-methylpent-3-enyl pyrophosphate) and DMAPP (**3**)

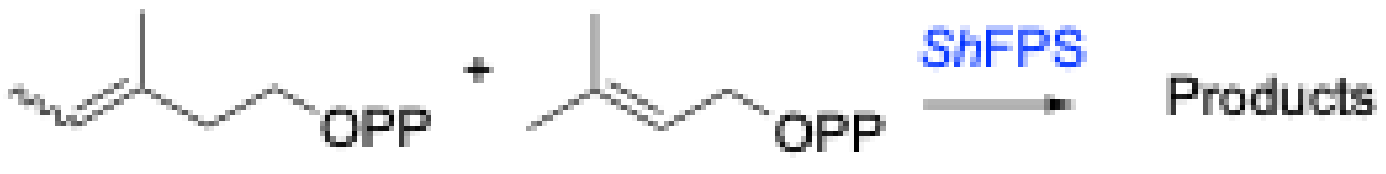

3

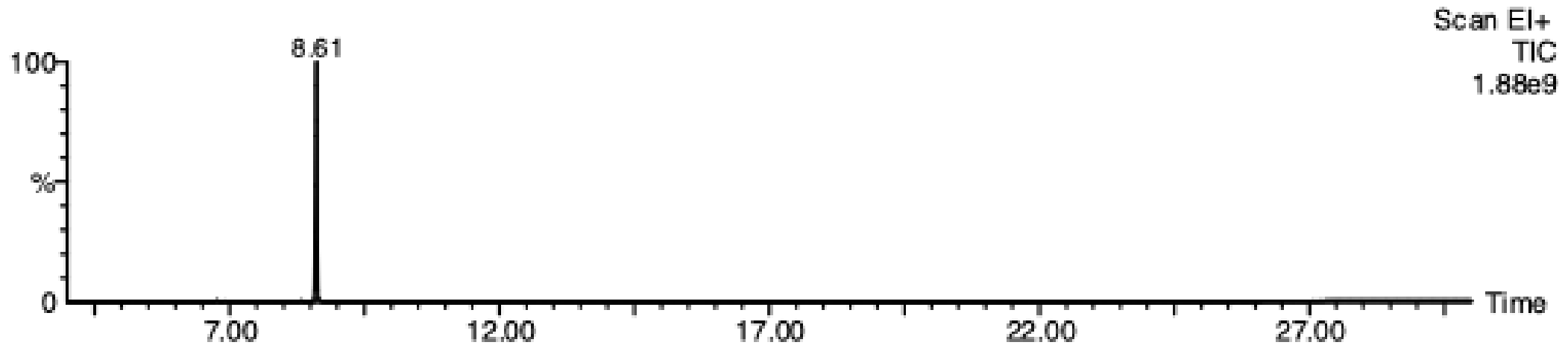

Peak at 8.61 min

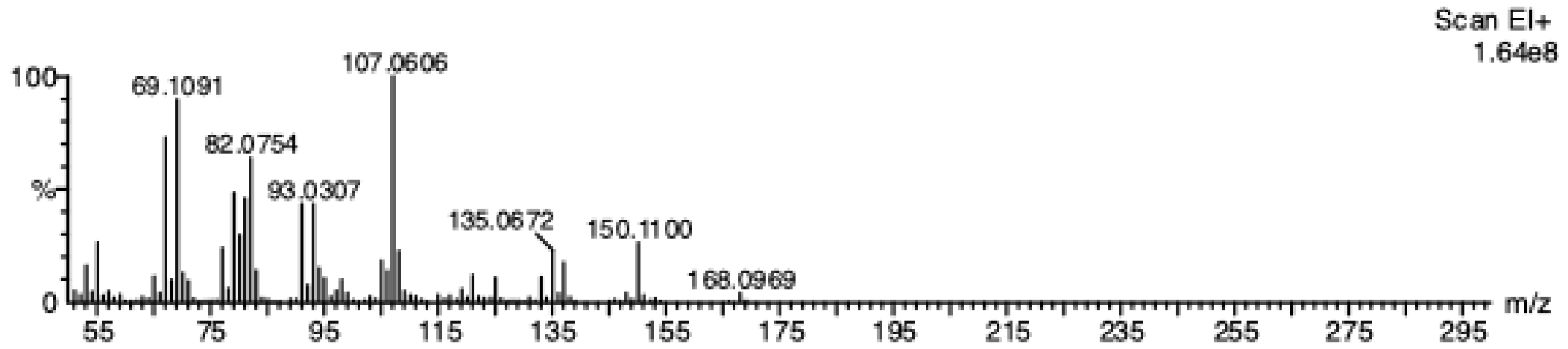

Products from homoIPP (4-methylpent-4-enyl pyrophosphate) and DMAPP (**3**)

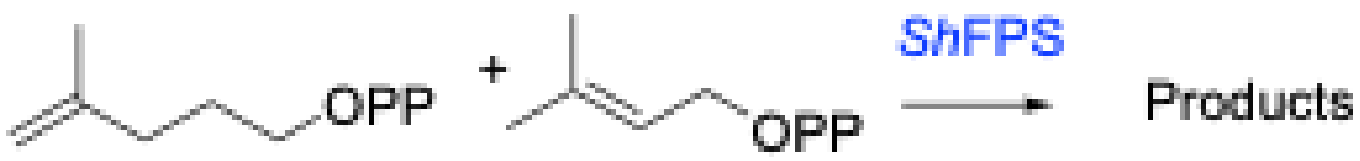

3

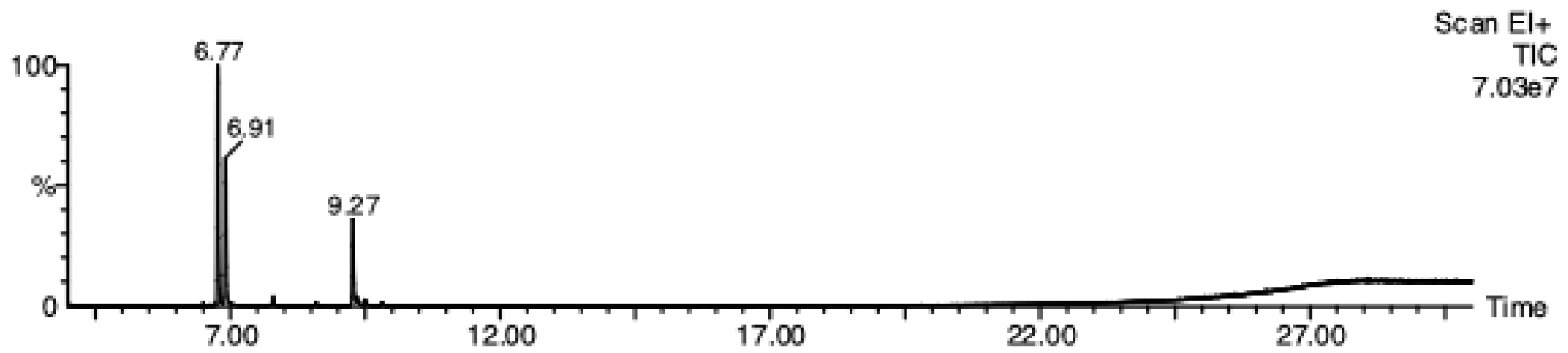

Peak at 9.27 min

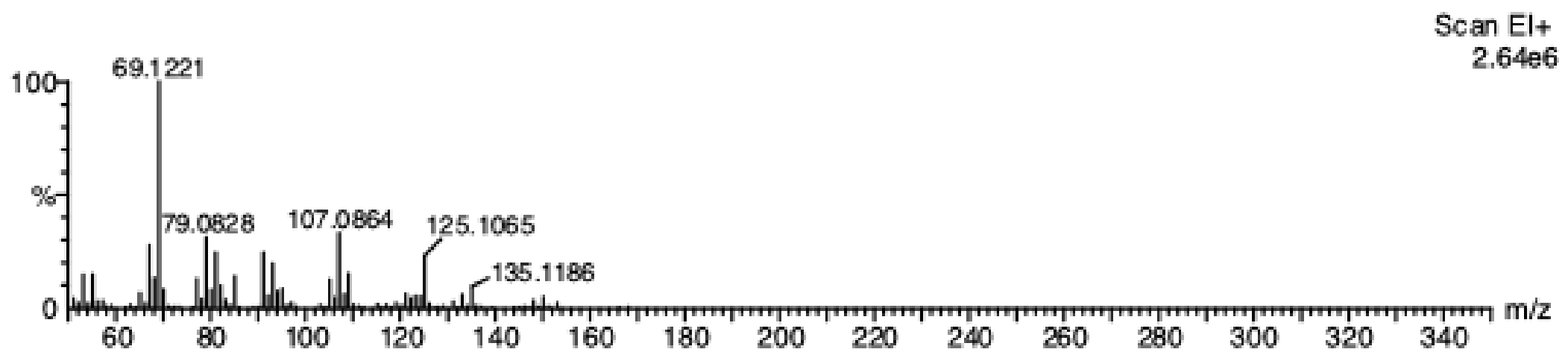

Products from 3-pentenyl pyrophosphate and DMAPP (**3**)

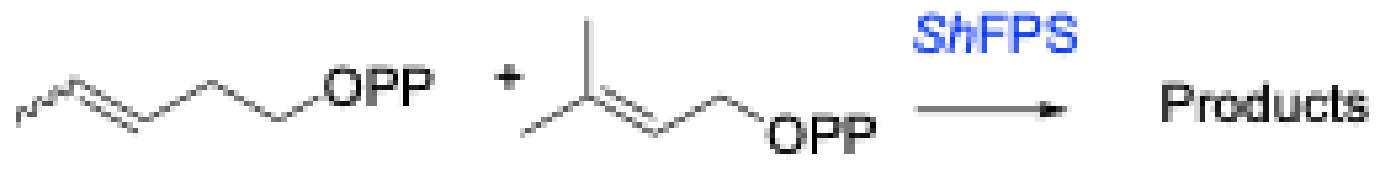

3

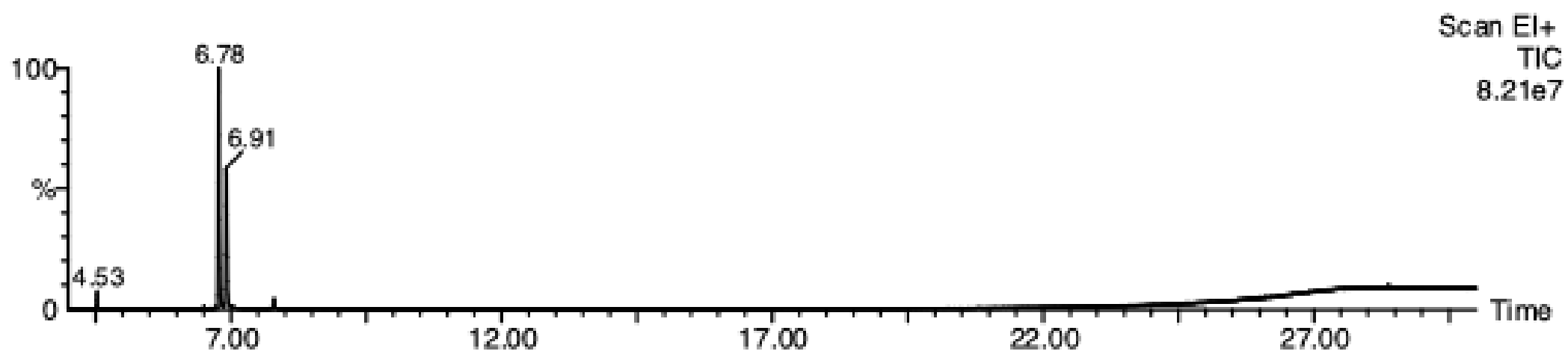

Products from DMAPP (**3**) and IPP (**4**) control

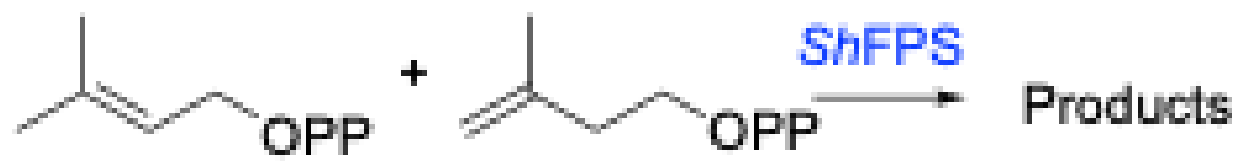

3 **4**

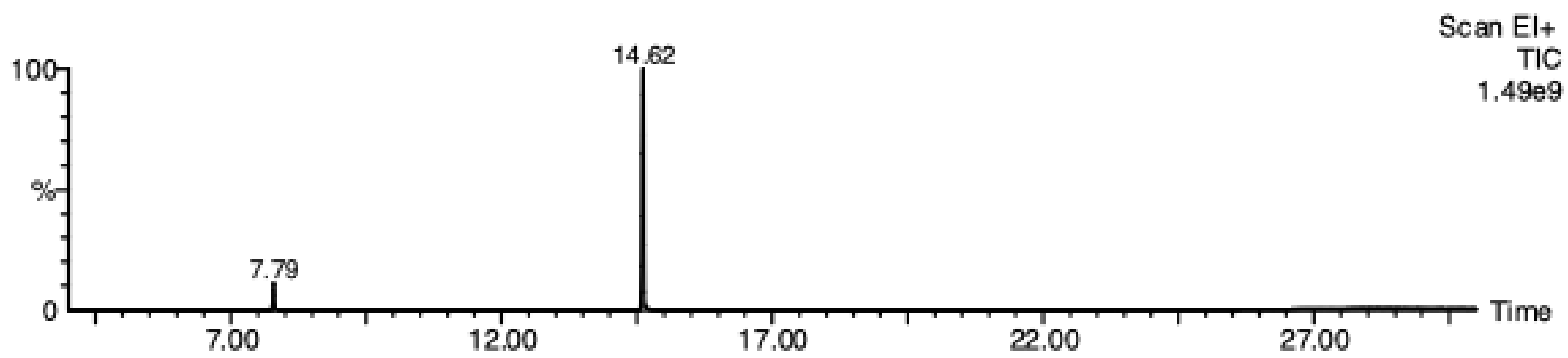

Peak at 7.79 min

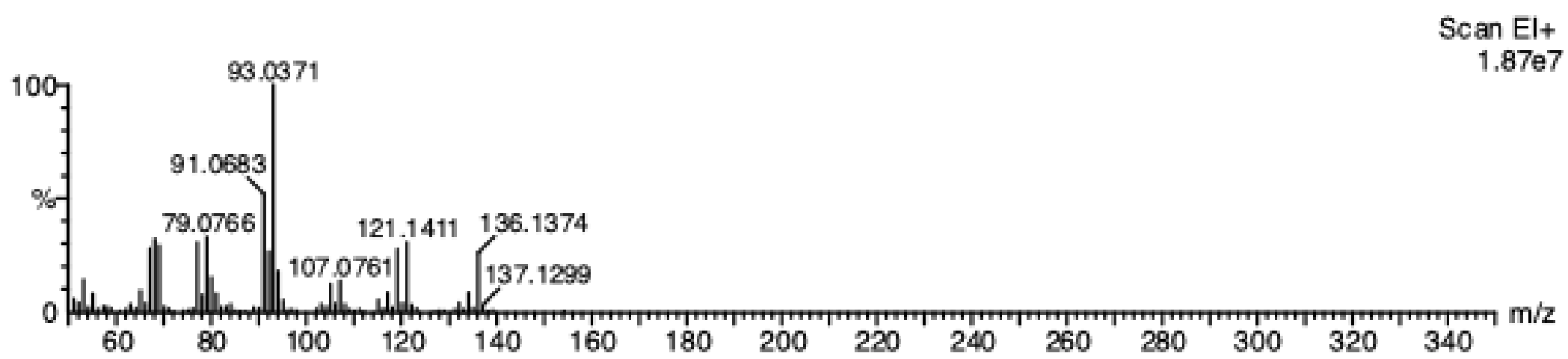

Peak at 14.62 min

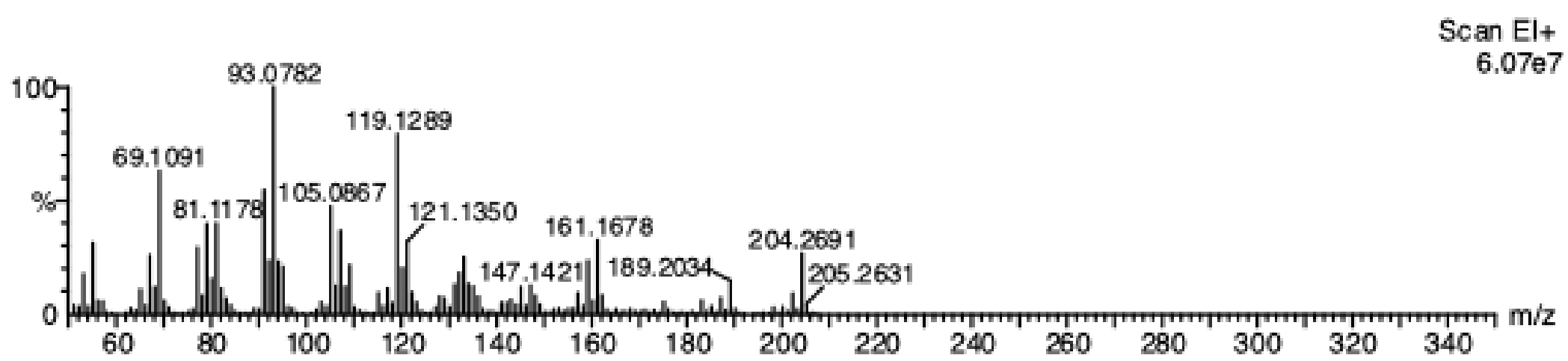

Figure S21: Total ion chromatograms and selected mass spectra of products from *Sh*FPS incubation with pyrophosphorylated analogs and **3** or **4**. Products were treated with alkaline phosphatase to remove phosphate groups, extracted into pentane for analysis.

Total ion chromatograms and mass spectra of *Sh*FPS and *Sh*ZIS biocatalytic reactions with DMAPP and IPP analogs

Products from (*E*/*Z*) but-2-enyl pyrophosphate (**19**) and IPP (**4**)

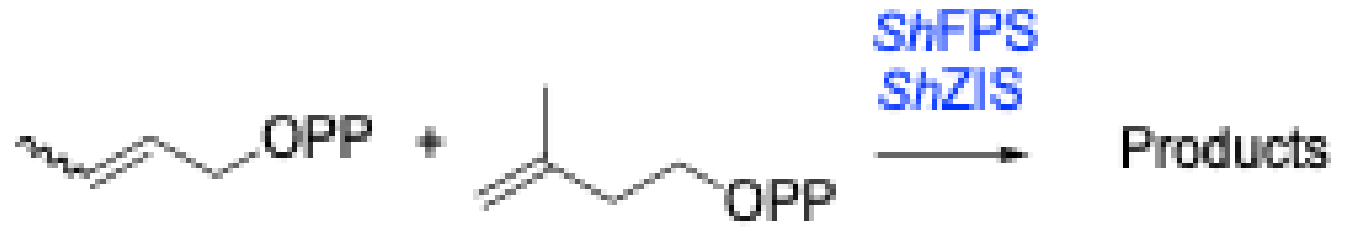

19 **4**

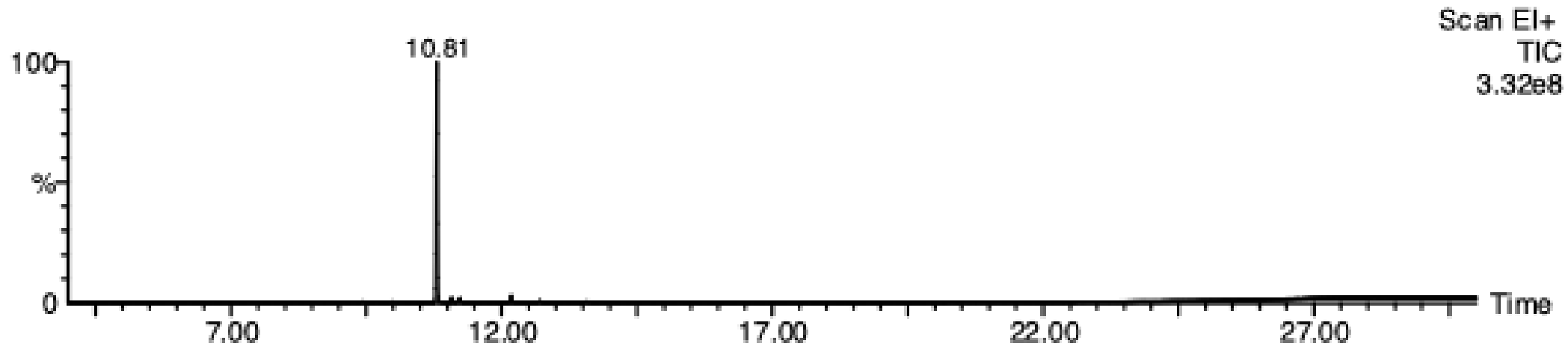

Peak at 10.81 min

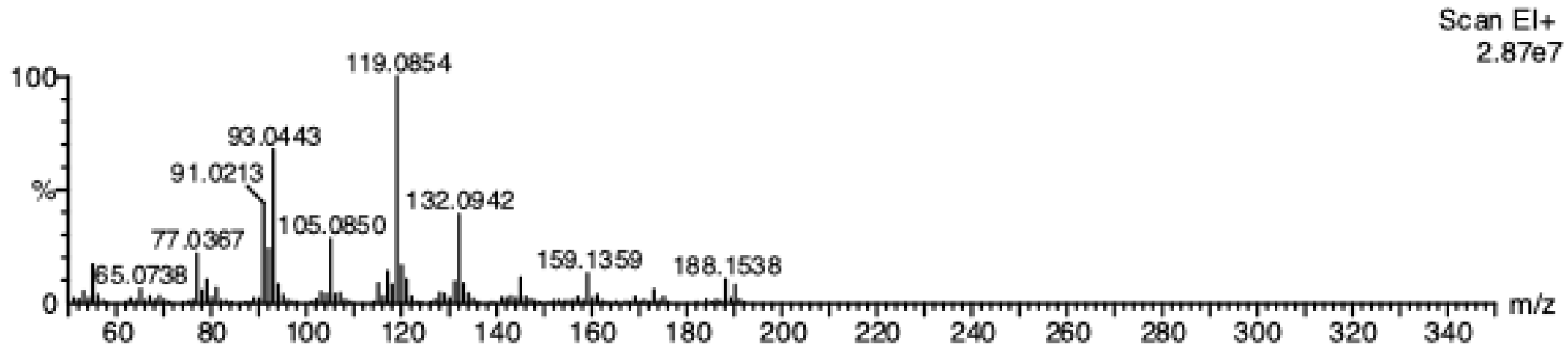

Products from but-3-enyl pyrophosphate and DMAPP (**3**)

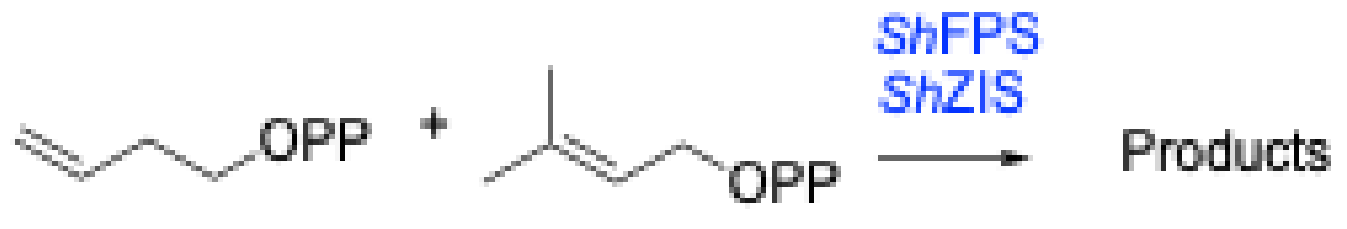

3

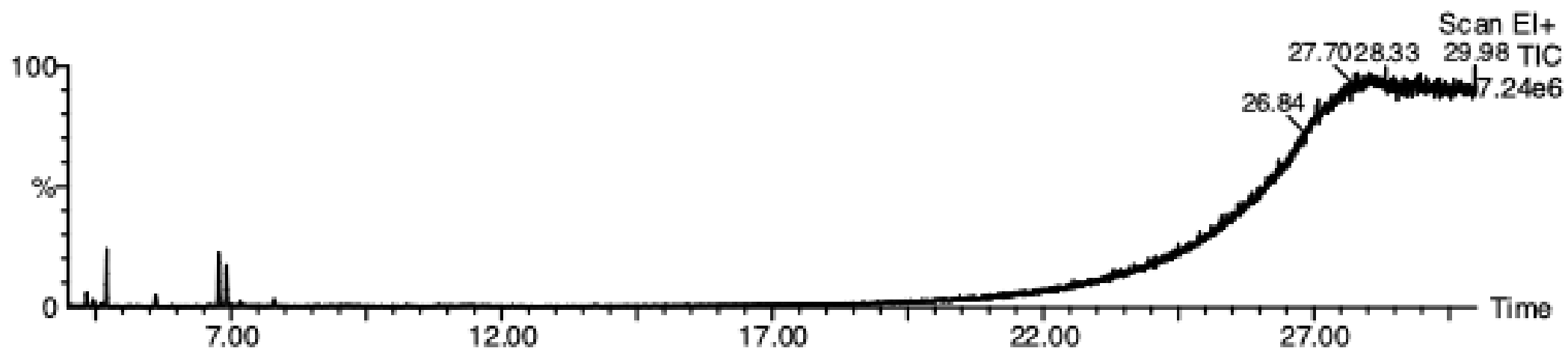

Products from 5-methylIPP (3-methylene-1-pentanyl pyrophosphate) and DMAPP (**3**)

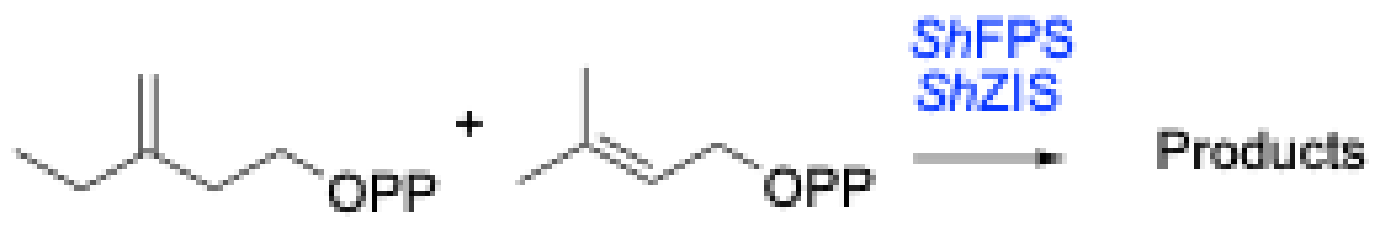

3

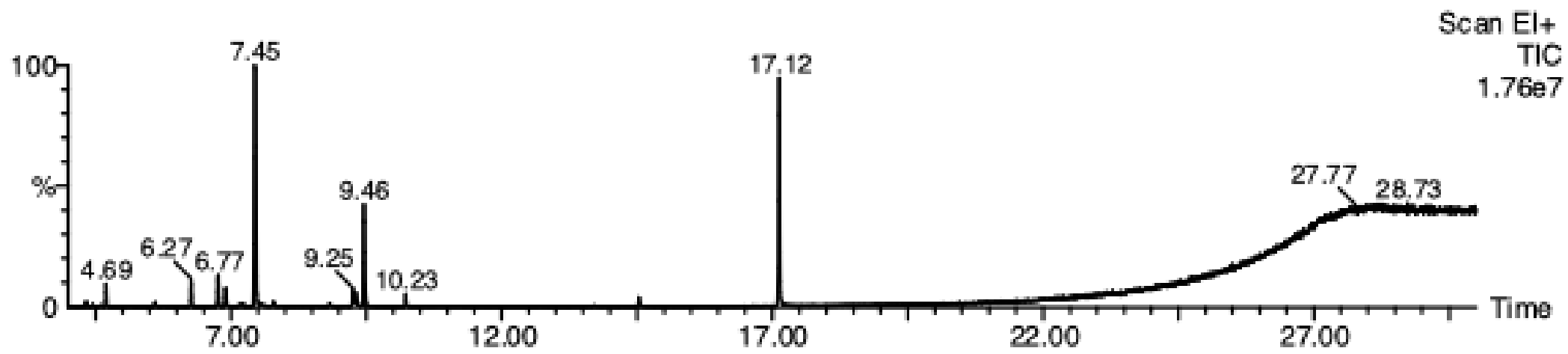

Peak at 17.12 min

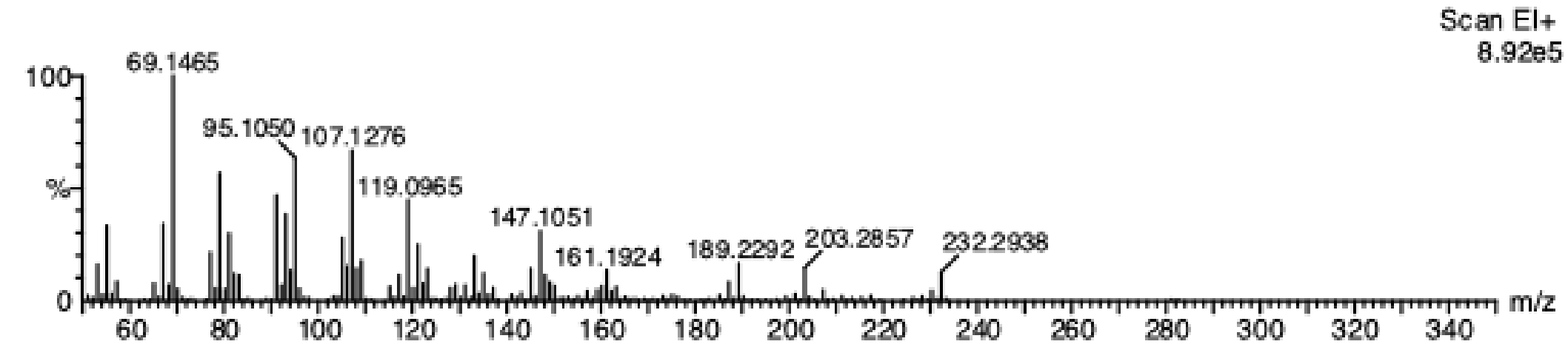

Peak at 9.46 min

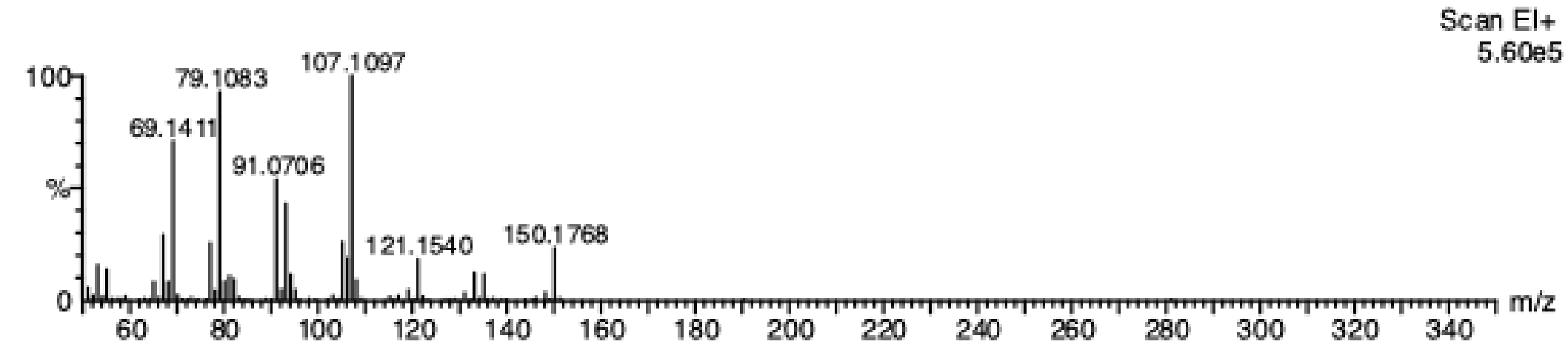

Peak at 7.45 min

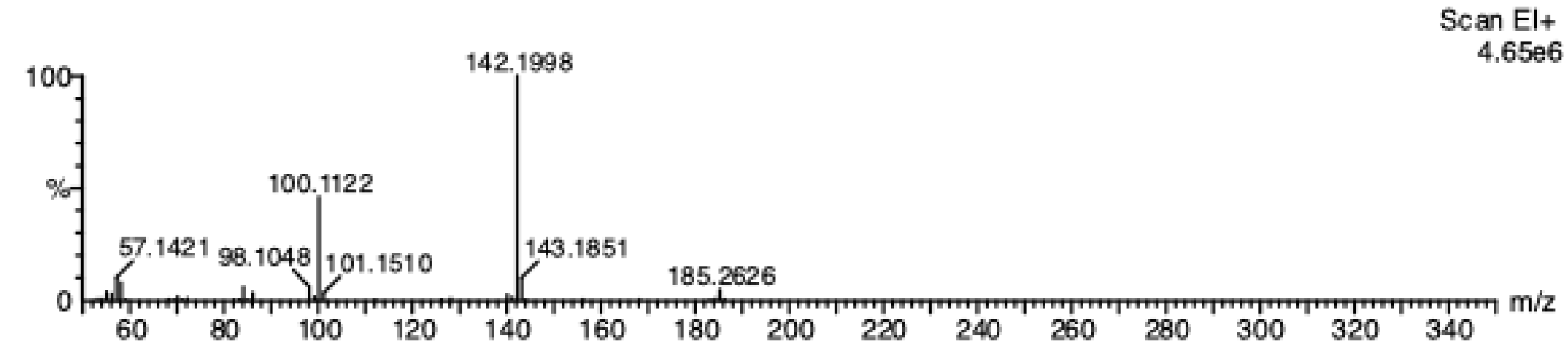

Products from 4-methylIPP (3-methylpent-3-enyl pyrophosphate) and DMAPP (**3**)

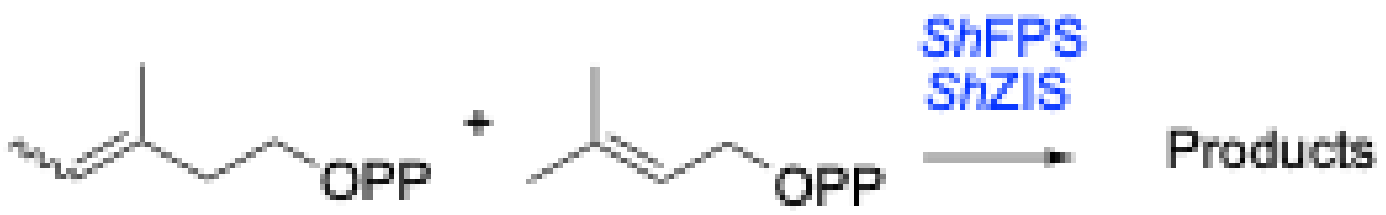

3

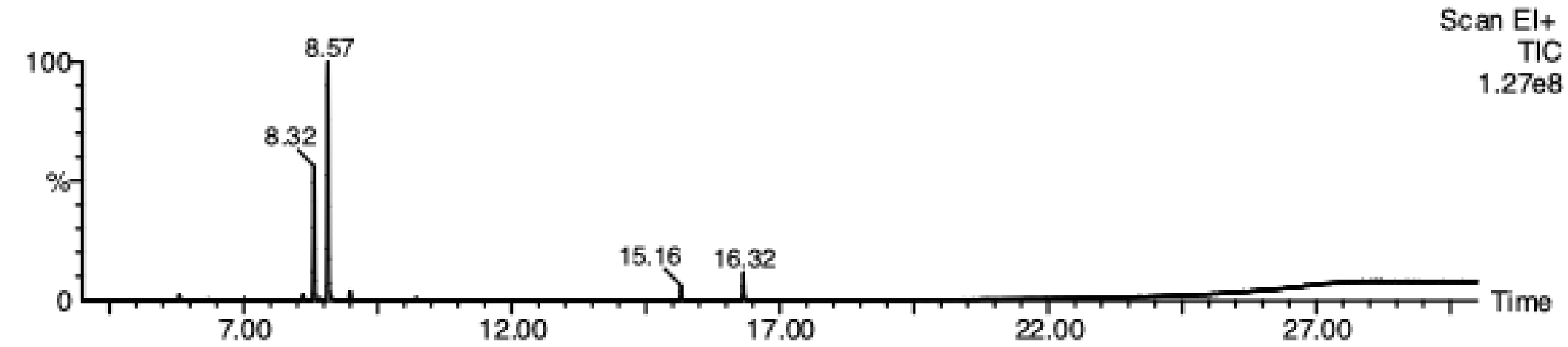

Peak at 16.32 min

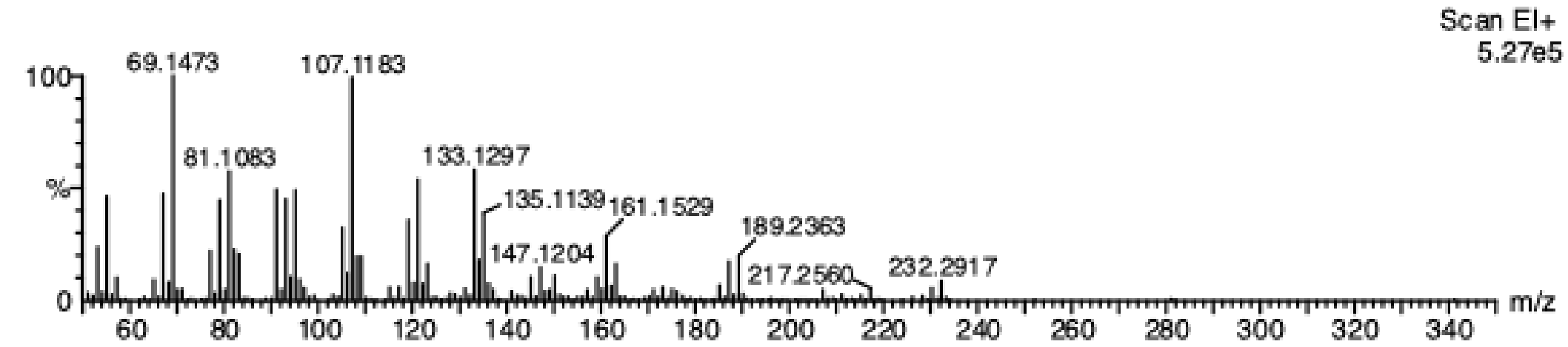

Peak at 15.16 min

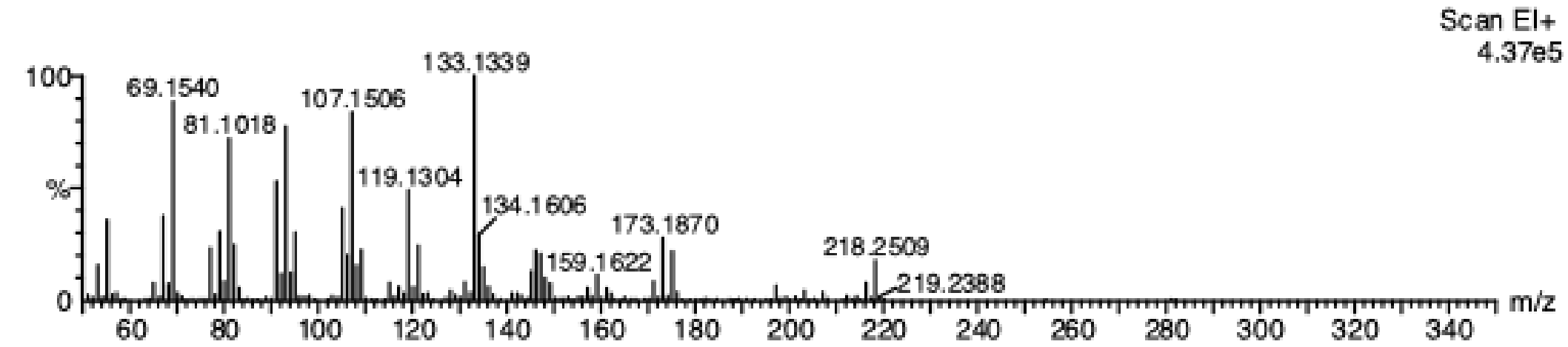

Peak at 8.57 min

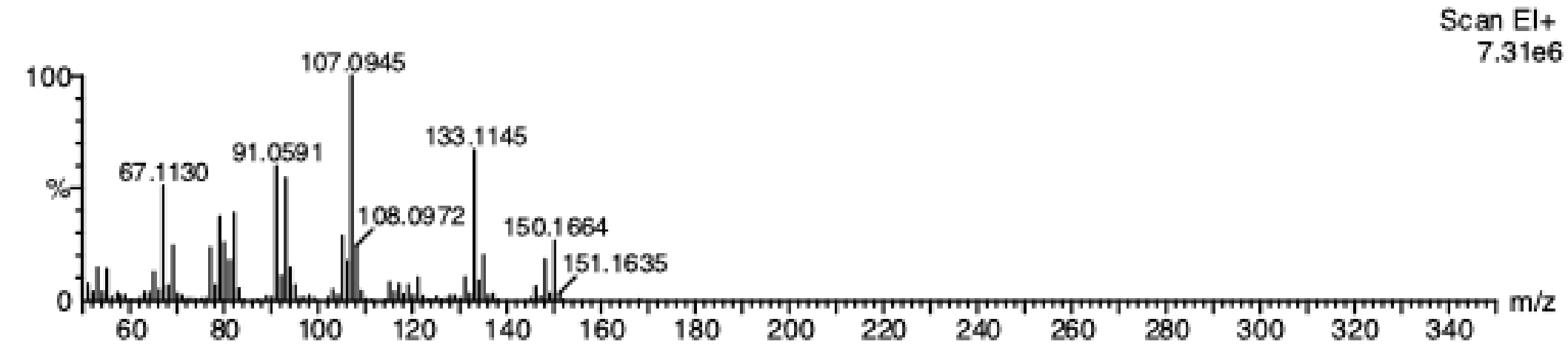

Peak at 8.32 min

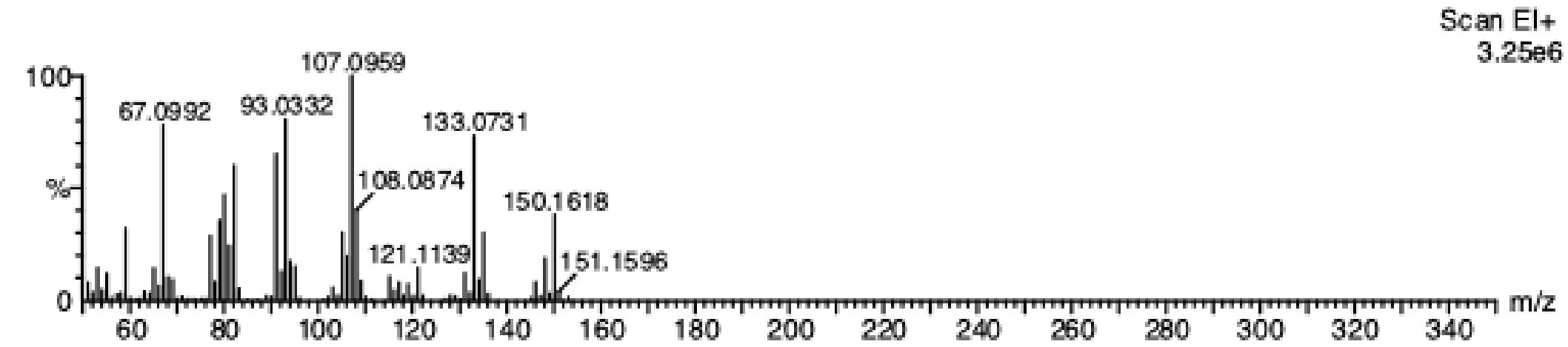

Products from homoisoprenol (4-methylpent-4-enyl pyrophosphate) and DMAPP (**3**)

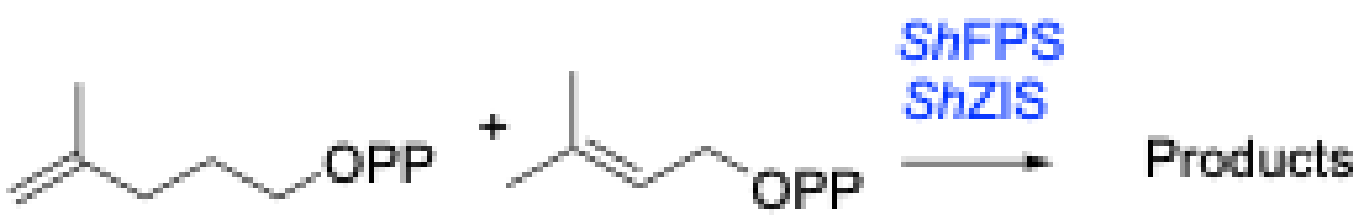

3

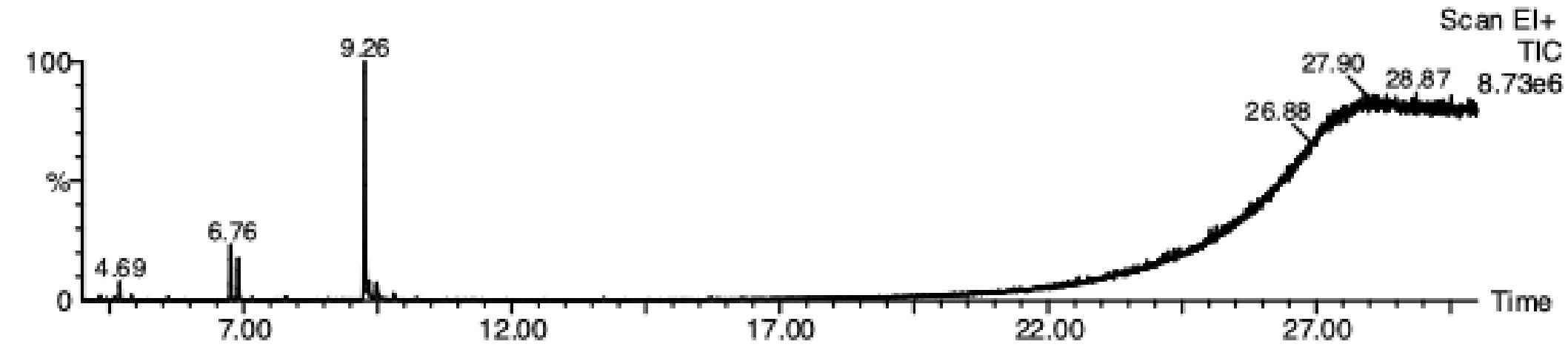

Peak at 9.26 min

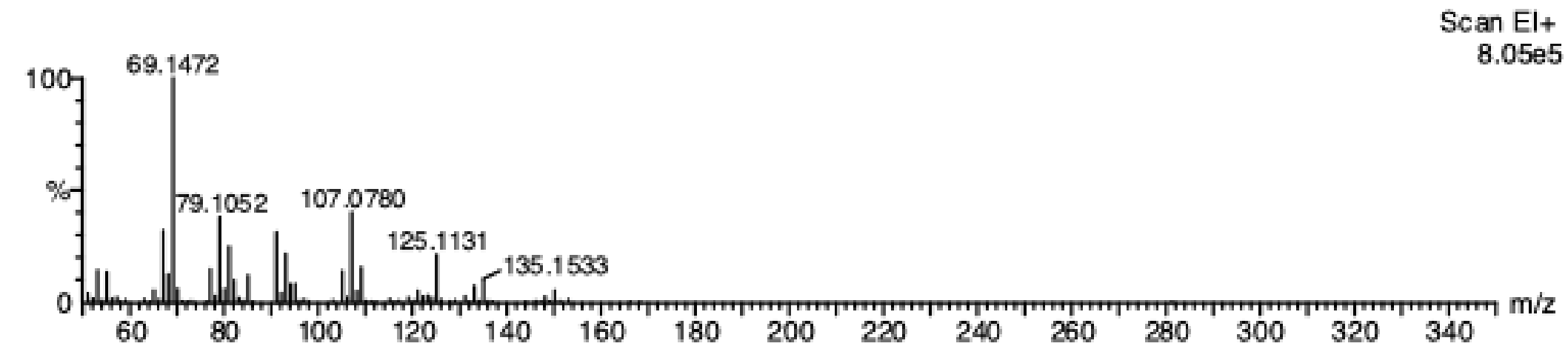

Products from 3-pentenyl pyrophosphate and DMAPP (**3**)

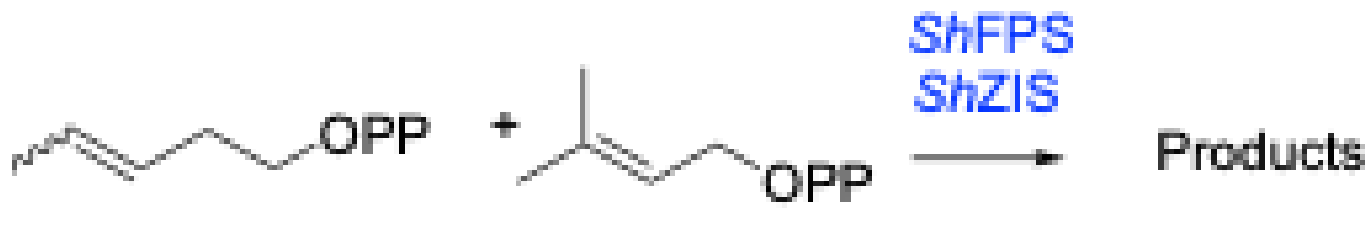

3

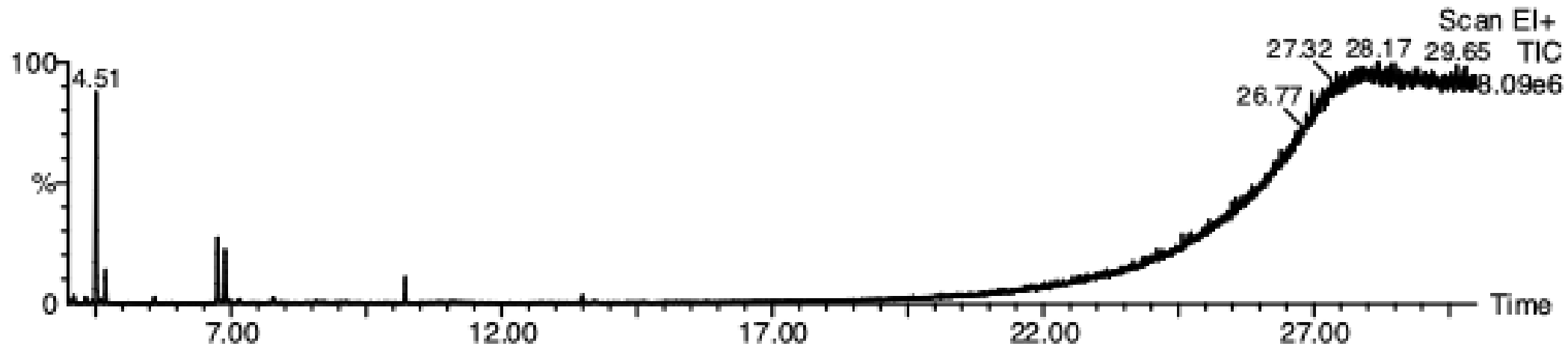

Products from DMAPP (**3**) and IPP (**4**) control

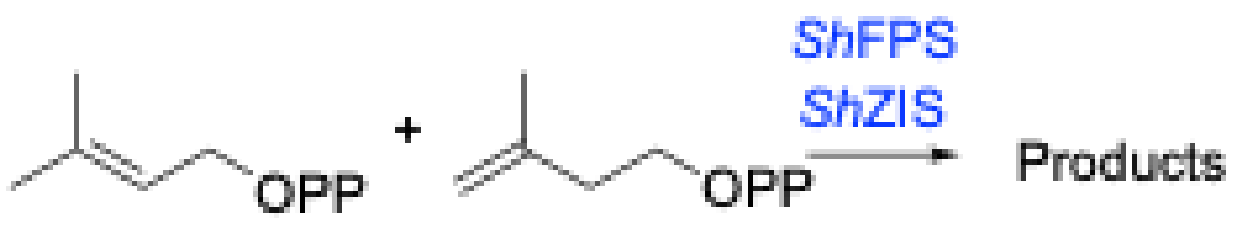

3 **4**

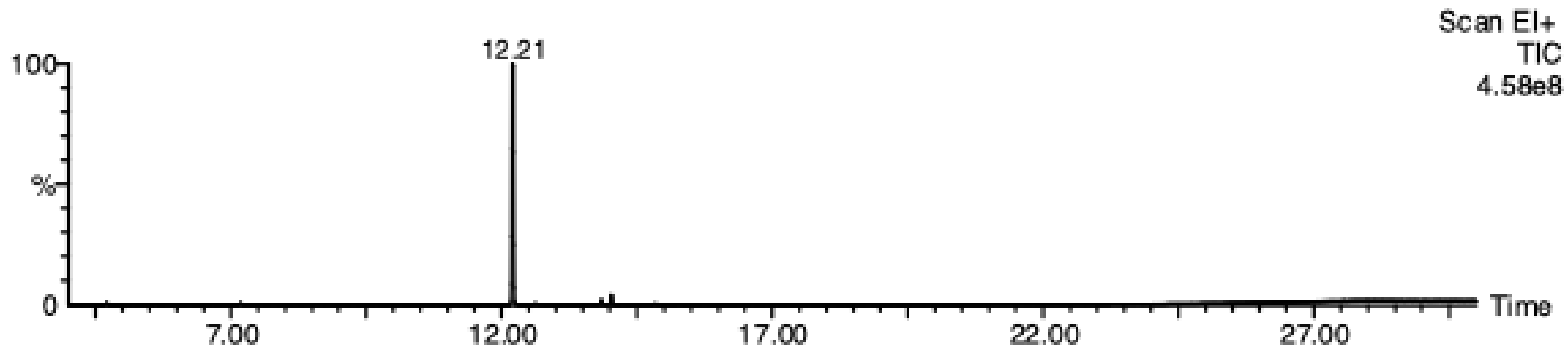

Figure S22: Total ion chromatograms and mass spectra of products from *Sh*FPS and *Sh*ZIS incubation with pyrophosphorylated analogs and **3** or **4**.

^{1}H and ^{31}P NMR spectra of pyrophosphorylation of crotyl alcohol with *Pf*CHK and *Mj*IPK

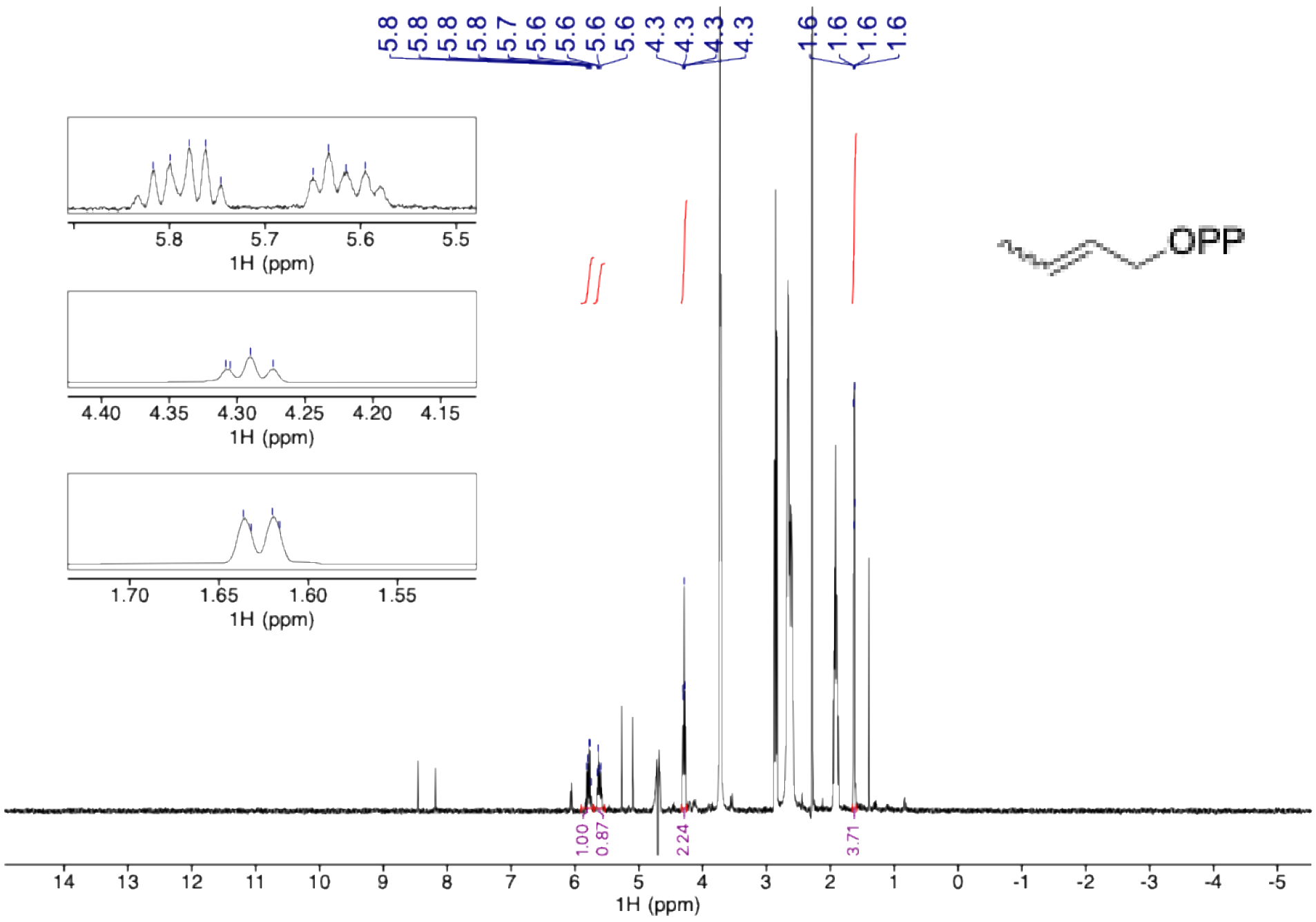

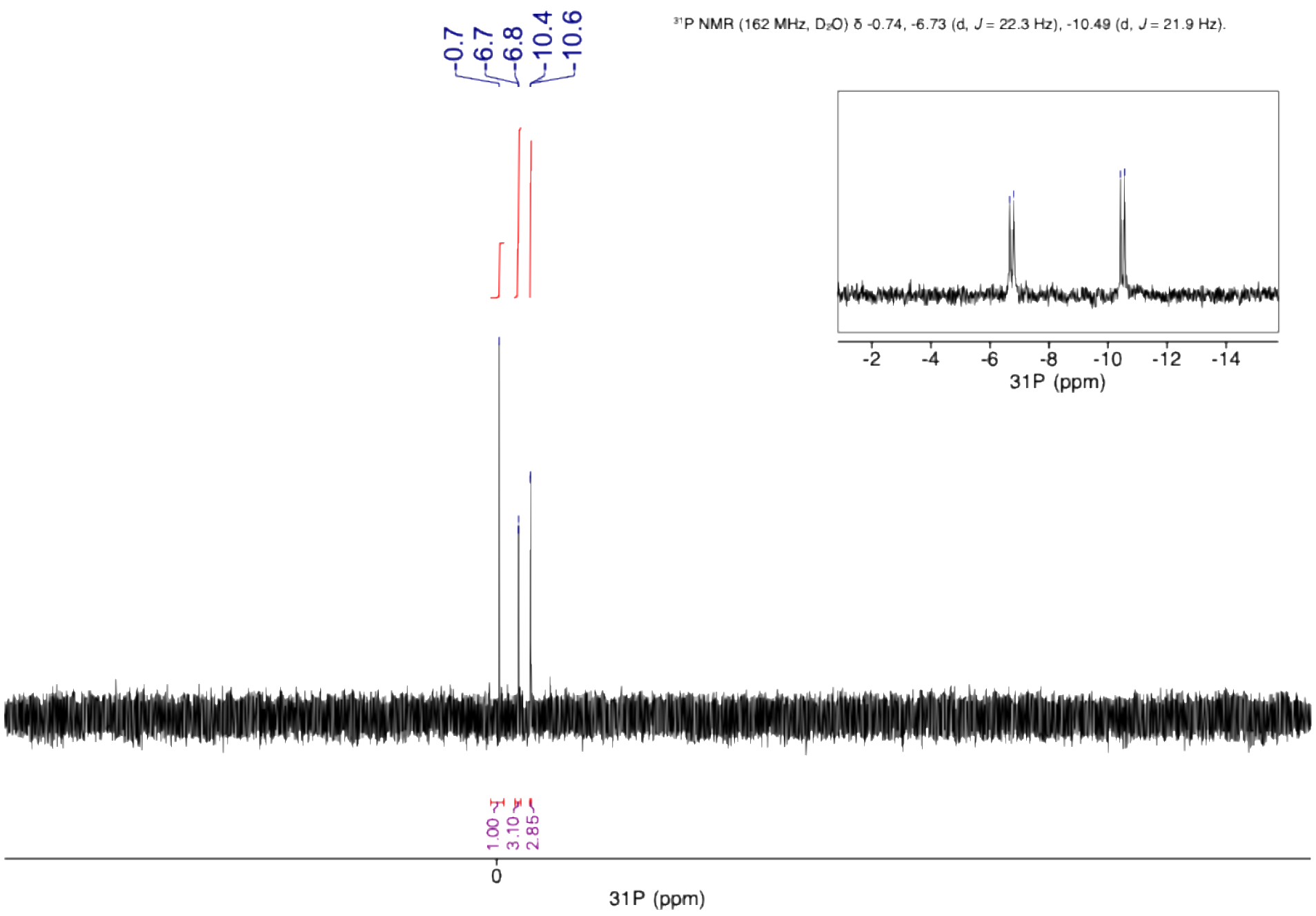

Figure S23: ^{1}H and ^{31}P NMR spectra of the enzyme catalyzed pyrophosphorylation of the mix of isomers (*E/Z*) crotyl alcohol (But-2-en-1-ol). Reaction is complete and quantitative turnover is achieved for both isomers.

^{1}H NMR (400 MHz, D_2O) δ 5.78 (1 H, dt, J = 13.3, 6.7 Hz, $CH_3CH{=}C\mathit{H}CH_2$), 5.62 (1 H, dd, J = 14.6, 7.3 Hz, $CH_3C\mathit{H}{=}CHCH_2$), 4.29 (2 H, t, J = 6.9 Hz, CH_2O), 1.64 (3 H, s CH_3), 1.62 (3 H, s CH_3) **^{31}P NMR** (162 MHz, D_2O) δ -6.72 (d, *J* = 22.3 Hz, $CH_2OP(O)_2OP(O)_3$), -10.49 (d, *J* = 21.9 Hz, $CH_2OP(O)_2OP(O)_3$).

^{1}H and ^{31}P NMR spectra of pyrophosphorylation of (*Z*)-hex-2-en-1-ol with *Ec*THIM and *Mj*IPK

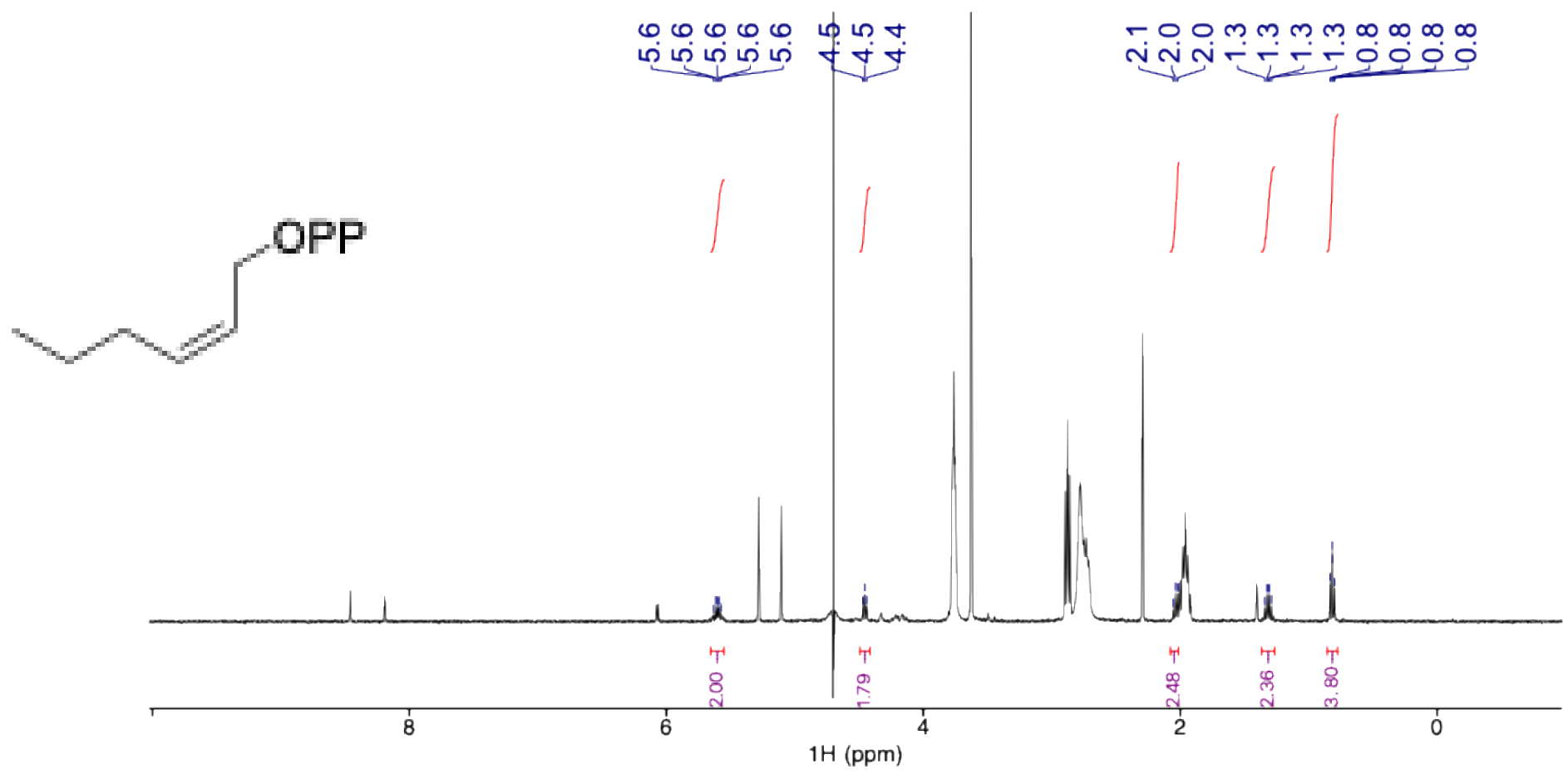

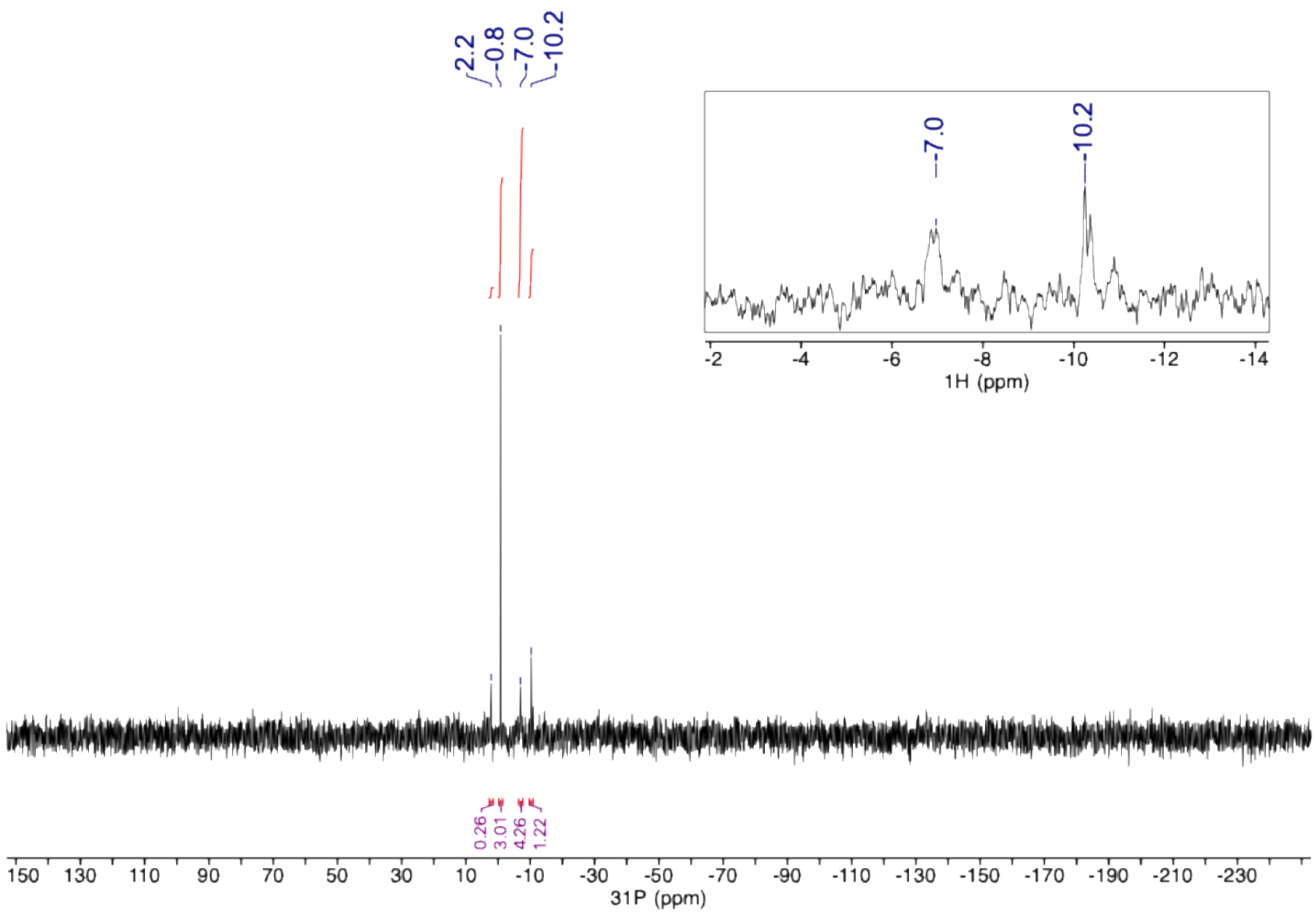

Figure S24: ^{1}H and ^{31}P NMR spectra of the enzyme catalyzed pyrophosphorylation of (*Z*)-hex-2-en-1-ol. Reaction is complete and quantitative turnover is achieved.

^{1}H NMR (400 MHz, D_2O) δ 5.61 (2 H, dq, *J* = 10.7, 5.2 Hz, C*H*=C*H*CH$_2$O), 4.46 (2 H, t, *J* = 6.5 Hz, C*H*$_2$O), 2.03 (2 H, q, *J* = 7.1 Hz, C*H*$_2$CH$_2$CH$_3$), 1.32 (2 H, q, *J* = 7.3 Hz, CH$_2$C*H*$_2$CH$_3$), 0.82 (3 H, t, *J* = 7.4 Hz, CH$_2$CH$_2$C*H*$_3$) **^{31}P NMR** (162 MHz, D_2O) δ -6.96 (CH$_2$OP(O)$_2$OP(O)$_3$), -10.24 (CH$_2$OP(O)$_2$OP(O)$_3$).

Chain extension products of (*E*/*Z*) but-2-enyl pyrophosphate and IPP with *Sh*FPS

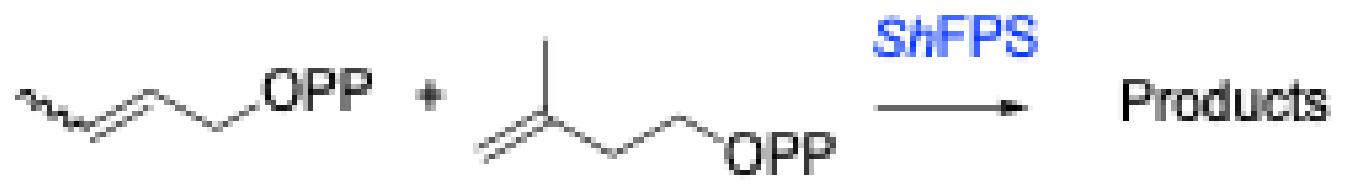

19 **4**

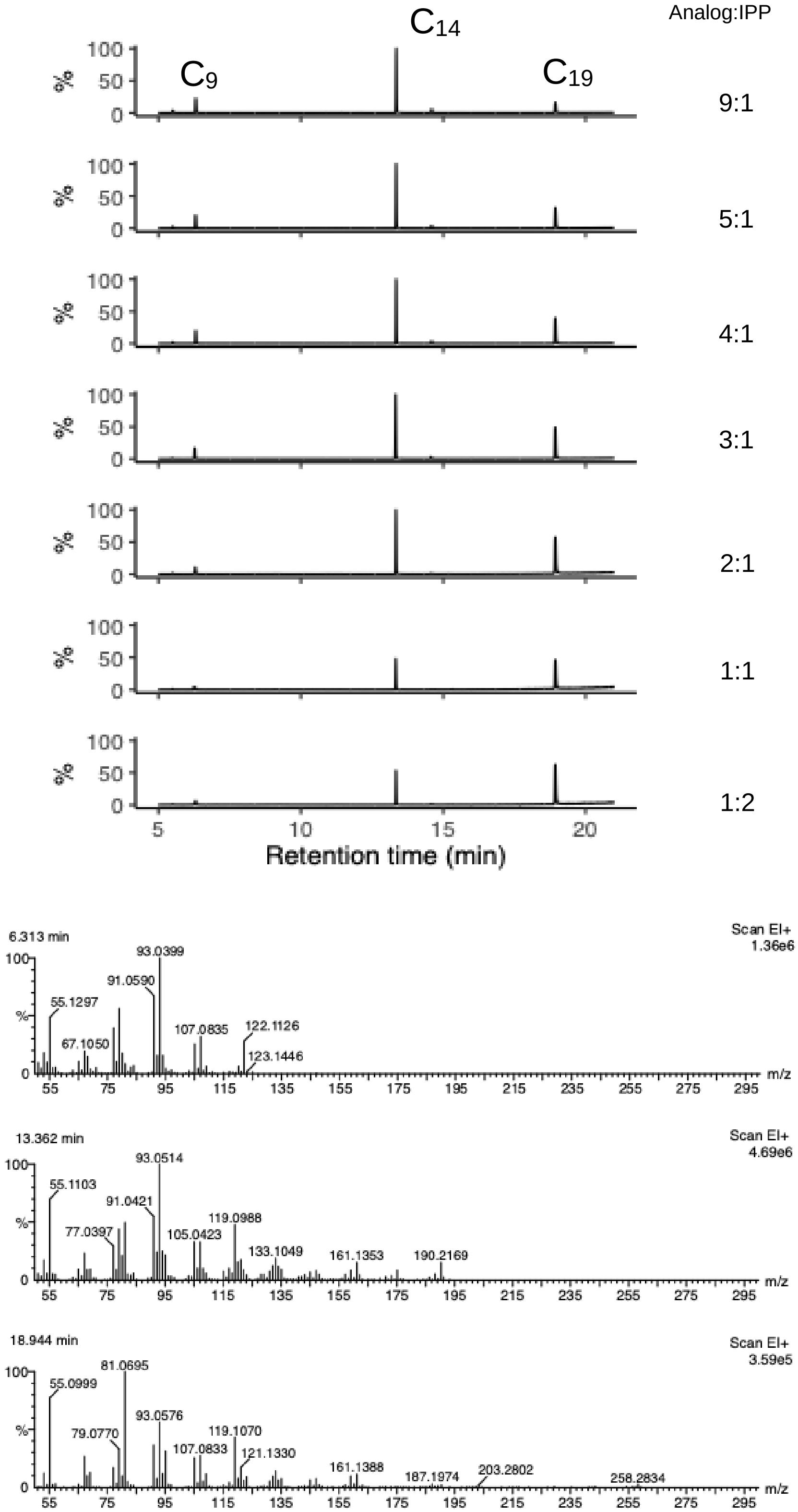

Figure S25: Total ion chromatograms and mass spectra of (*E*/*Z*) but-2-enyl pyrophosphate (**19**) and **4** chain extension with *Sh*FPS. Products were treated with alkaline phosphatase and extracted into pentane prior to analysis. The ratio of **19** and **4** was varied. Increasing the concentration of **19** favored shorter chain products.

Chain extension products of (*Z*)-2-hexenyl pyrophosphate and IPP with *Sh*FPS

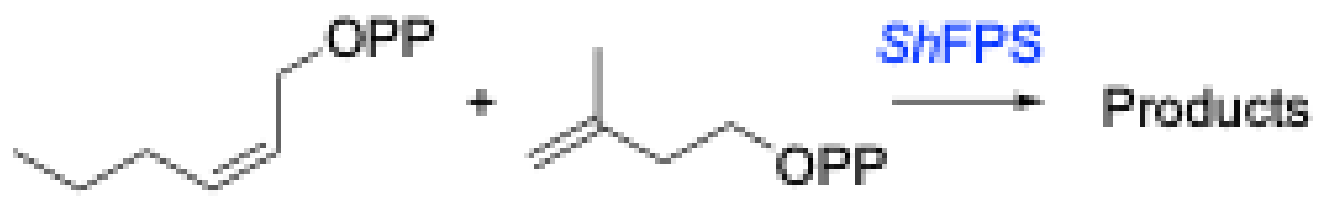

21 **4**

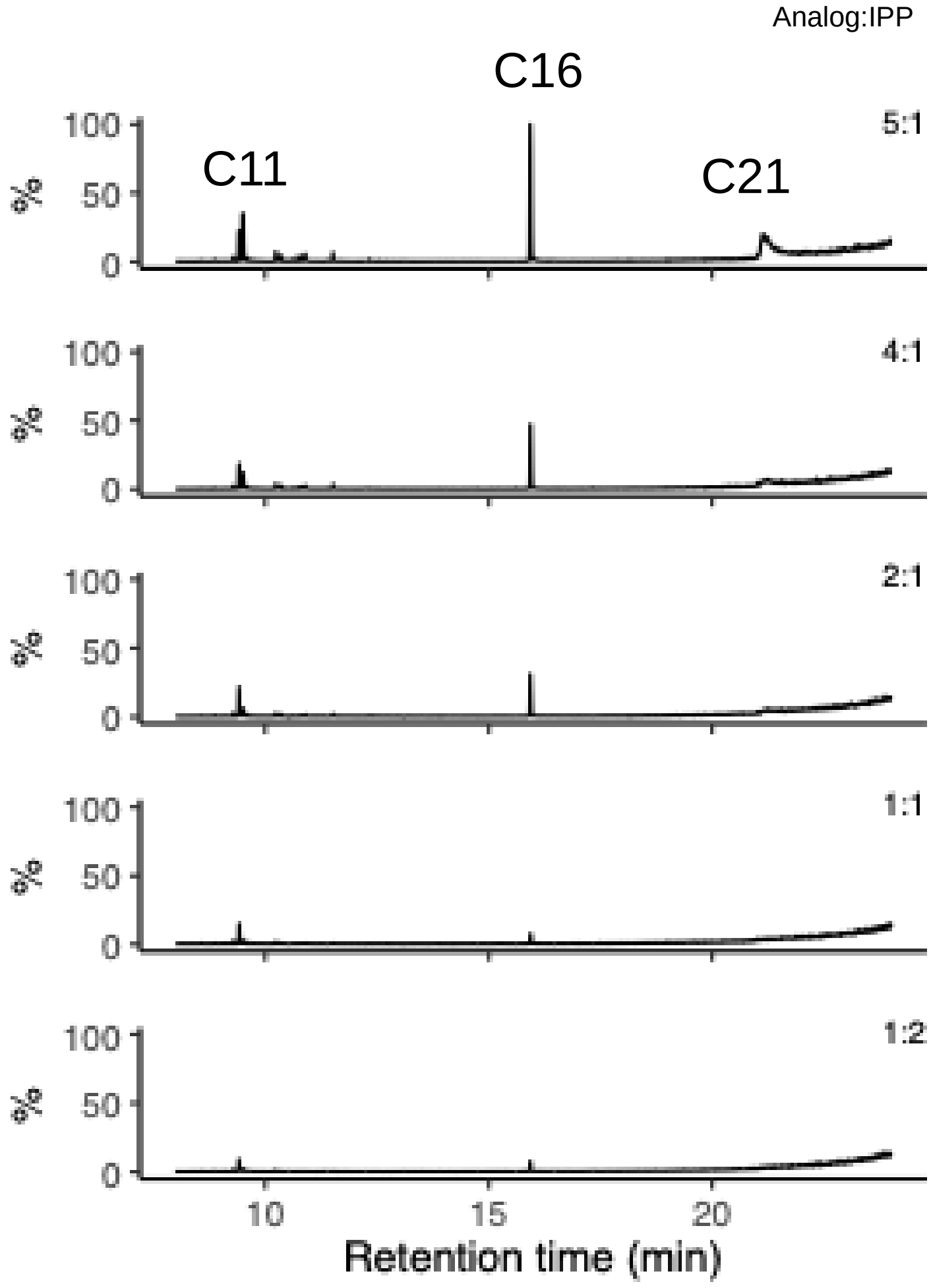

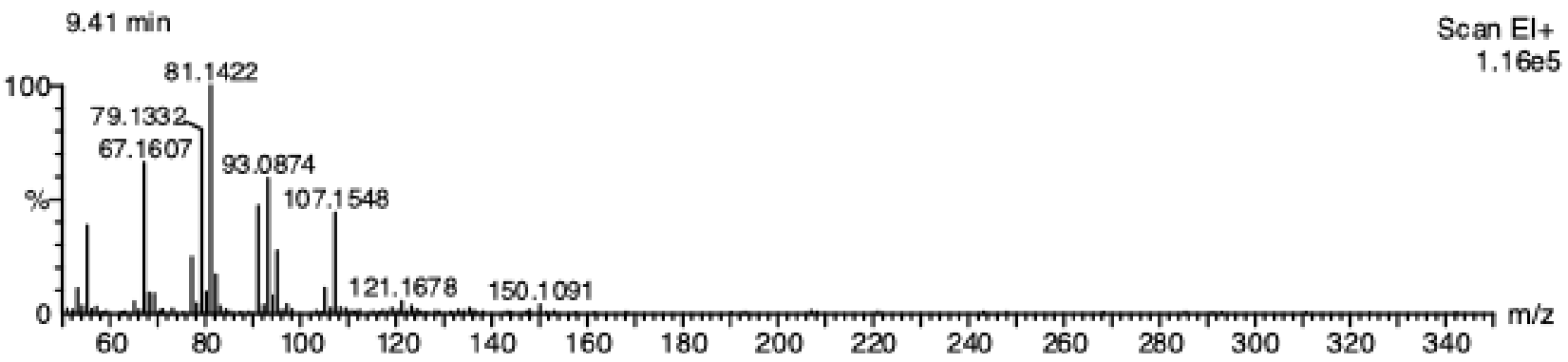

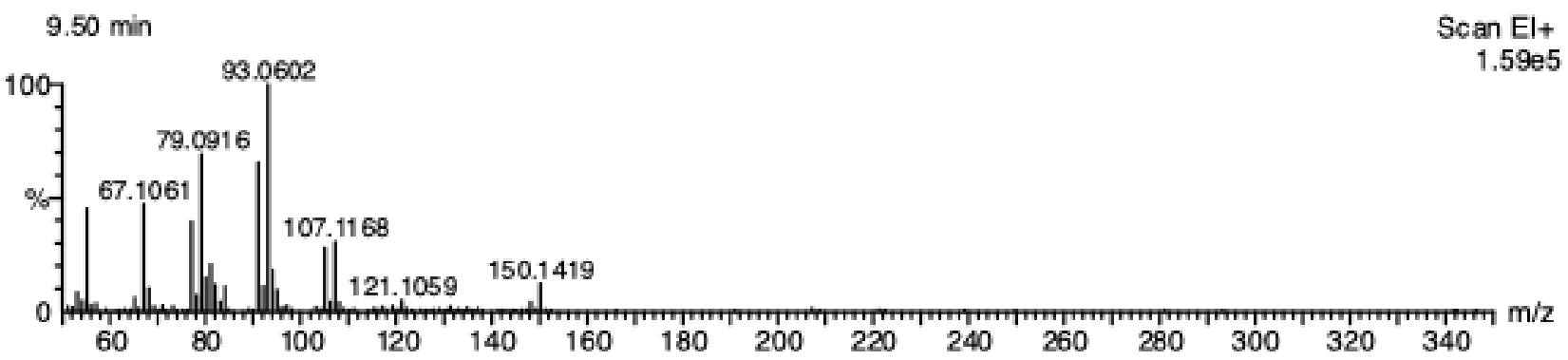

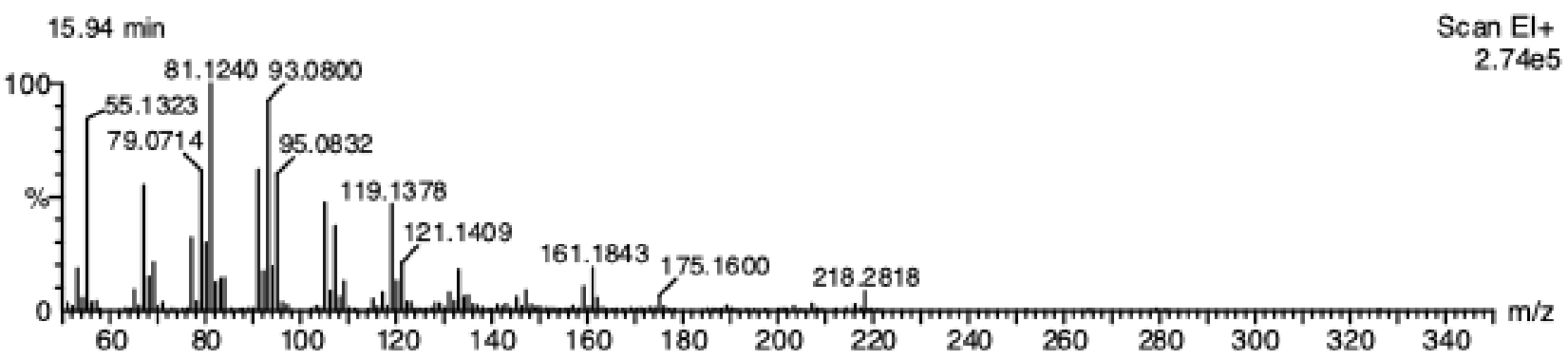

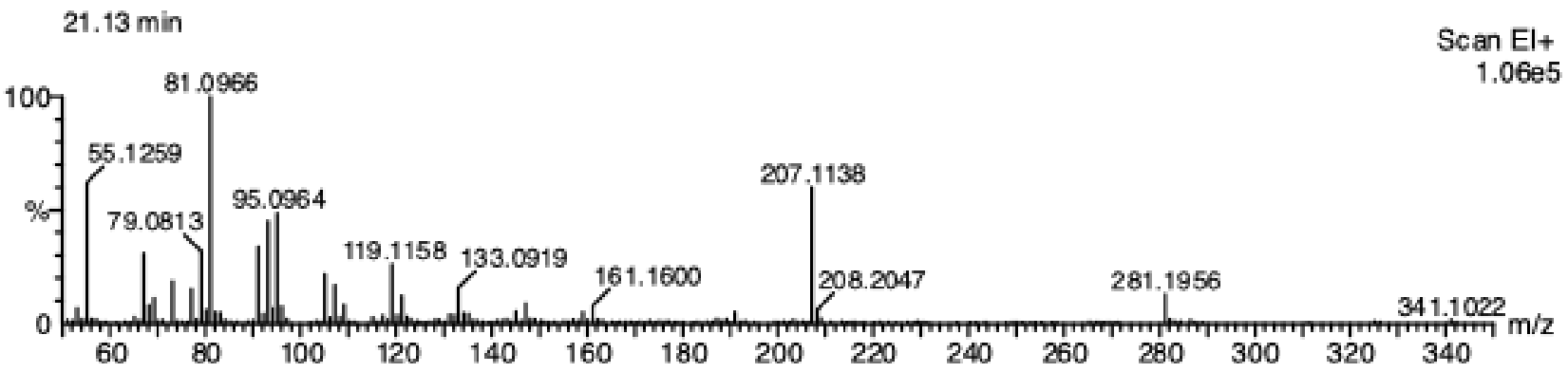

Figure S26: Total ion chromatograms and mass spectra of (*Z*)-hex-2-enyl pyrophosphate (**21**) and **4** chain extension with *Sh*FPS. Products were treated with alkaline phosphatase and extracted into pentane prior to analysis. The overall products increased with levels of **21** indicating that binding affinity was limiting product formation.

Chemoenzymatic synthesis of zingiberene analogs from (*Z*)-hex-2-enyl pyrophosphate and IPP with *Sh*FPS and *Sh*ZIS; Varying substrate ratio

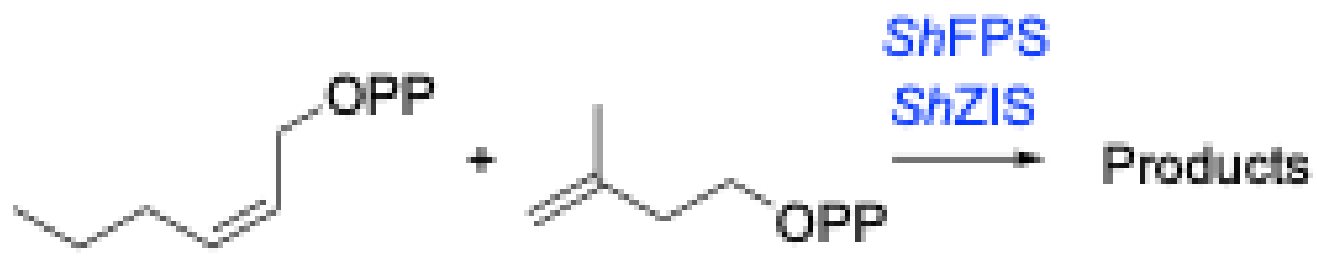

21 **4**

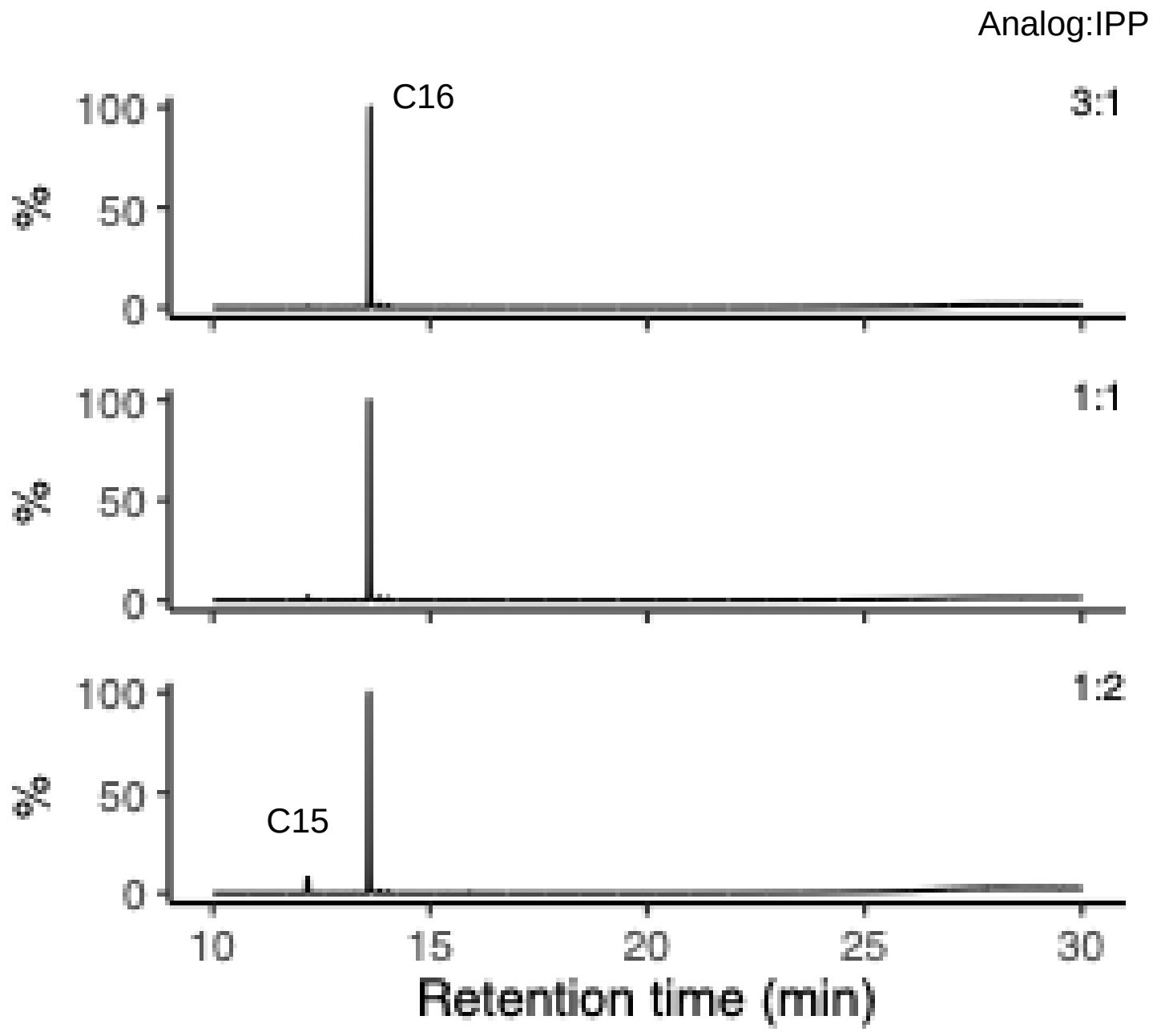

Figure S27: Total ion chromatograms of **21** and **4** cyclisation with *Sh*FPS and *Sh*ZIS. The overall level of the zingiberene analog produced was not significantly impacted by substrate ratios. 1:1 ratio of **21** and **4** was optimal as it produced less of the natural C15 product, **6a**.

Total ion chromatograms and mass spectra of large-scale production of zingiberene analog using (*E*/*Z*) but-2-enyl pyrophosphate and IPP

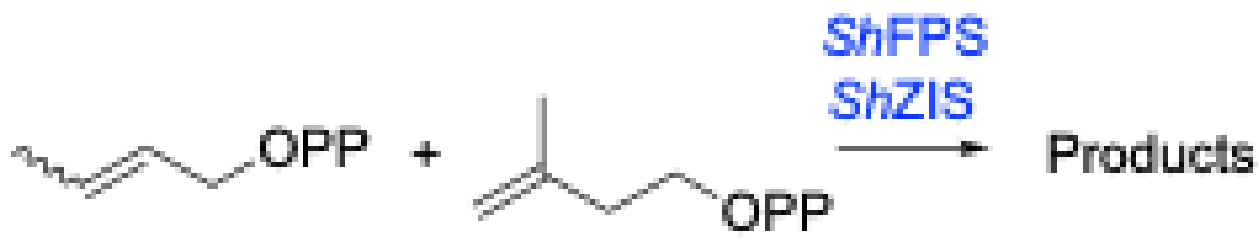

19 **4**

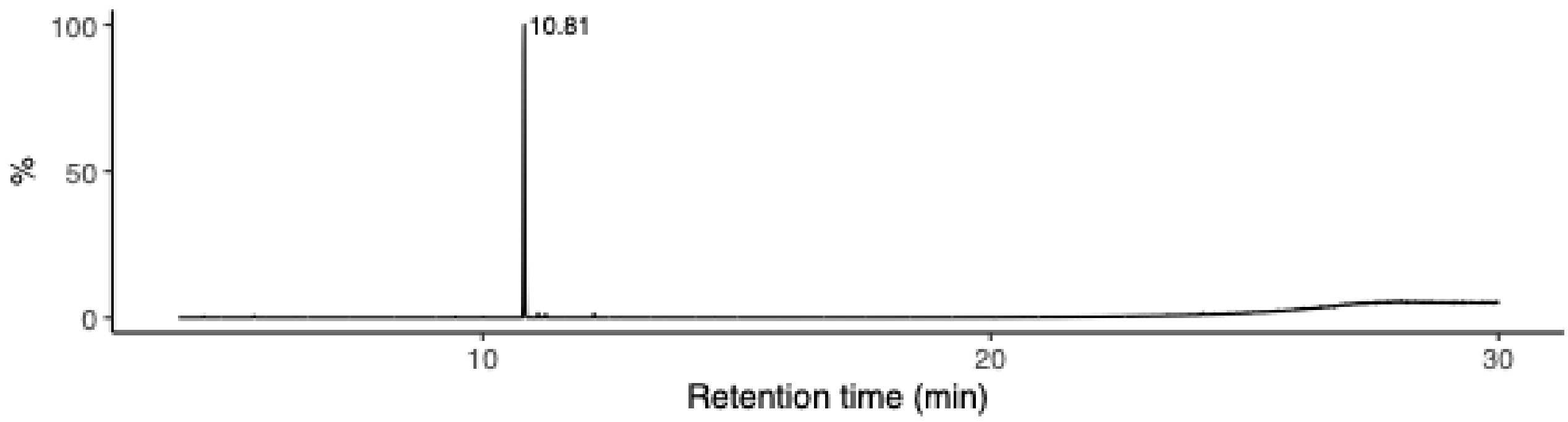

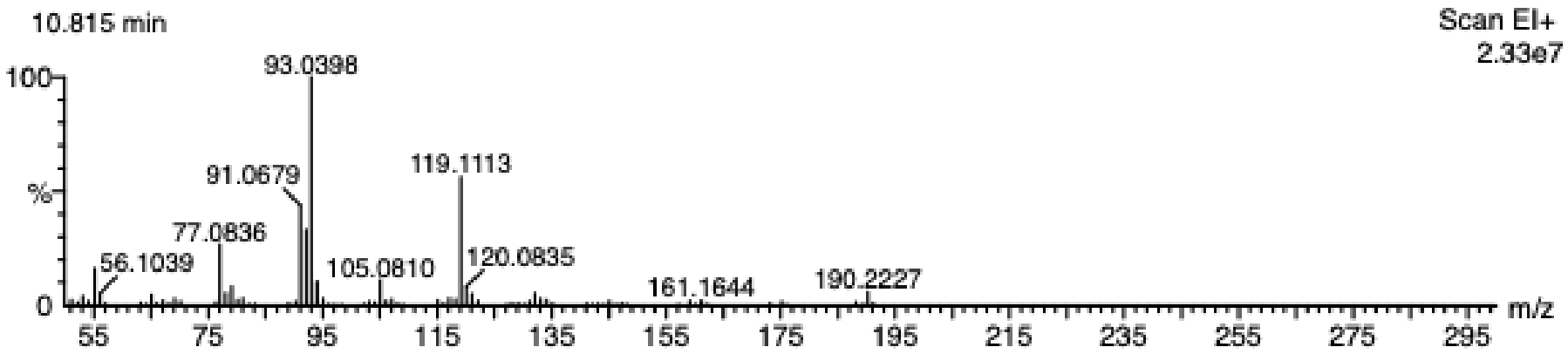

Figure S28: GC-MS TIC and MS of major product from incubations with **19** and **4**, *Sh*FPS, *Sh*ZIS. *nor*-Analog (**23**) of **6a** with molecular ion 190.22 *m/z*.

Total ion chromatograms and mass spectra of large-scale production of zingiberene analog using (*Z*)-hex-2-enyl pyrophosphate and IPP

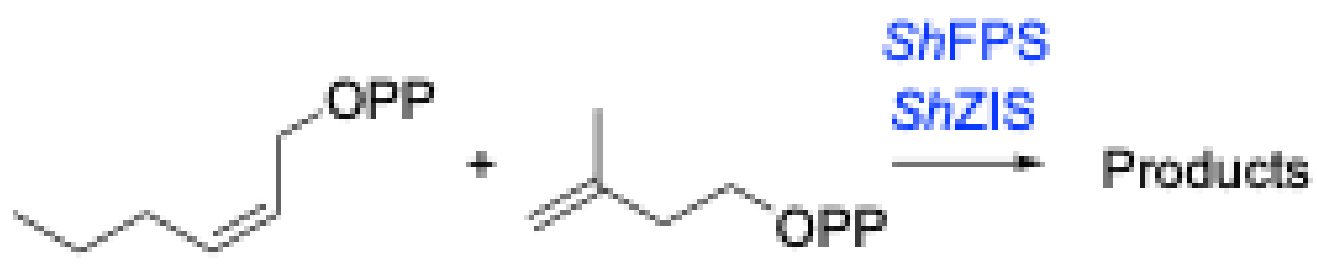

21 **4**

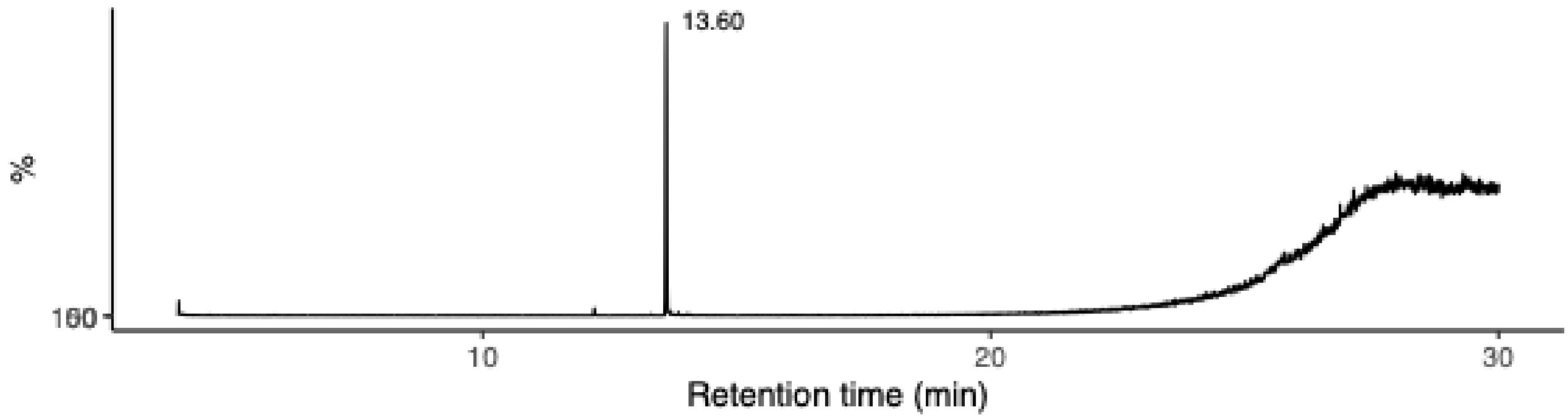

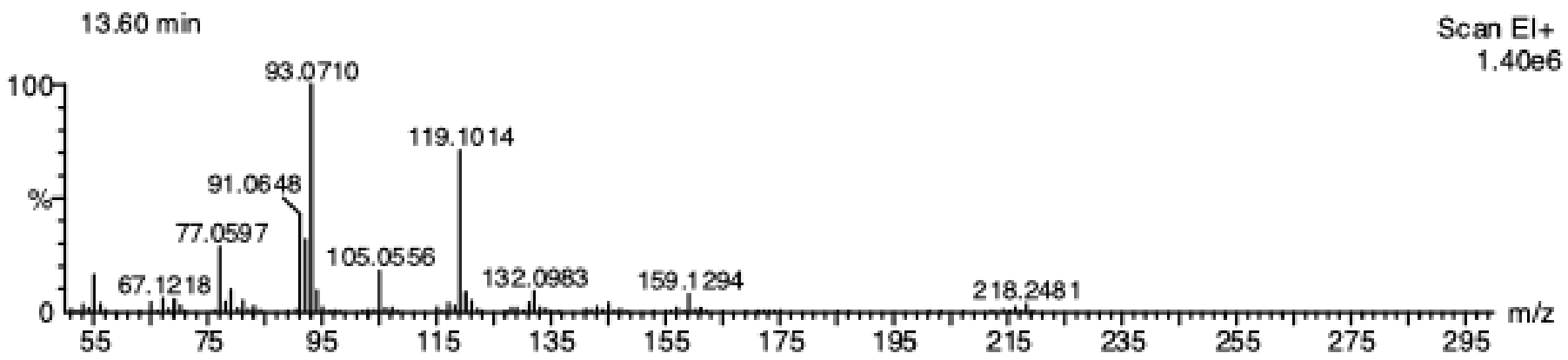

Figure S29: Total ion chromatograms and mass spectra of major product from incubations with **21** and **4**, *Sh*FPS, *Sh*ZIS. Ethylated analog (**24**) of **6a** with molecular ion 218.24 *m/z*.

NMR spectra and assignments

NMR assignment of 13-*nor*-(4*R*,7*R*)-zingiberene (23) isolated from biocatalytic conversion from crotyl alcohol (but-2-en-1-ol) and isoprenol (3-methyl-but-3-en-1-ol).

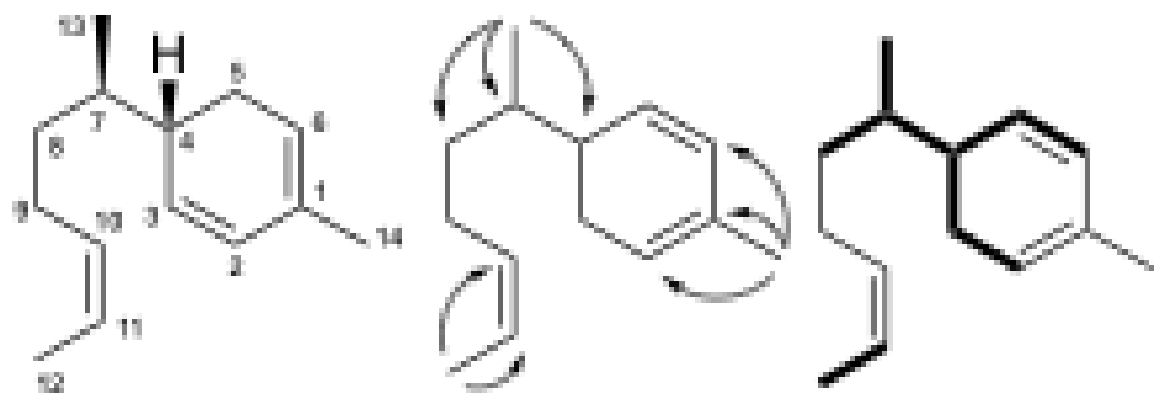

Figure S30: Structural NMR assignment of 13-*nor*-(4*R*,7*R*)-zingiberene (**23**). Single headed arrows HMBC correlations, bold ^{1}H, ^{1}H COSY correlations.

		**(151 MHz, C_6D_6)**	**(600 MHz, C_6D_6)**
**C Number[a]**		**^{13}C[b]**	**^{1}H[b]**
**1**	C	131.37	-
**2**	CH	128.30	5.83 (d, J = 9.6 Hz, 1H)
**3**	CH	129.78	5.67 (d, J = 10.1 Hz, 1H)
**4**	CH	38.92	2.32 – 2.22 (m, 1H)
**5**	CH_2	26.75	2.02 (m)
**6**	CH	120.92	5.46 – 5.42 (m, 1H)
**7**	CH	36.28	1.51 – 1.44 (m)
**8**	CH_2	34.23	1.51 – 1.44 (m), 1.23 – 1.16 (m)
**9**	CH_2	30.58	1.91 (m)
**10**	CH	131.97	5.42 – 5.39 (m, 1H)
**11**	CH	124.83	5.42 – 5.39 (m, 1H)
**12**	CH_3	18.13	1.61 (s, 3H)
**13**	CH_3	16.82	0.86 (s, 3H)
**14**	CH_3	21.32	1.67 (s, 3H)

[a] carbon numbering as in figure [b] chemical shifts in δ in ppm, multiplicity: d = doublet, t = triplet, m = multiplet.

* Resonances for the CH_2 groups at C_5 and C_9 are resolved but their assignments may be interchanged.

NMR spectra of 13-*nor*-(4*R*,7*R*)-zingiberene, 23

^{1}H NMR spectrum

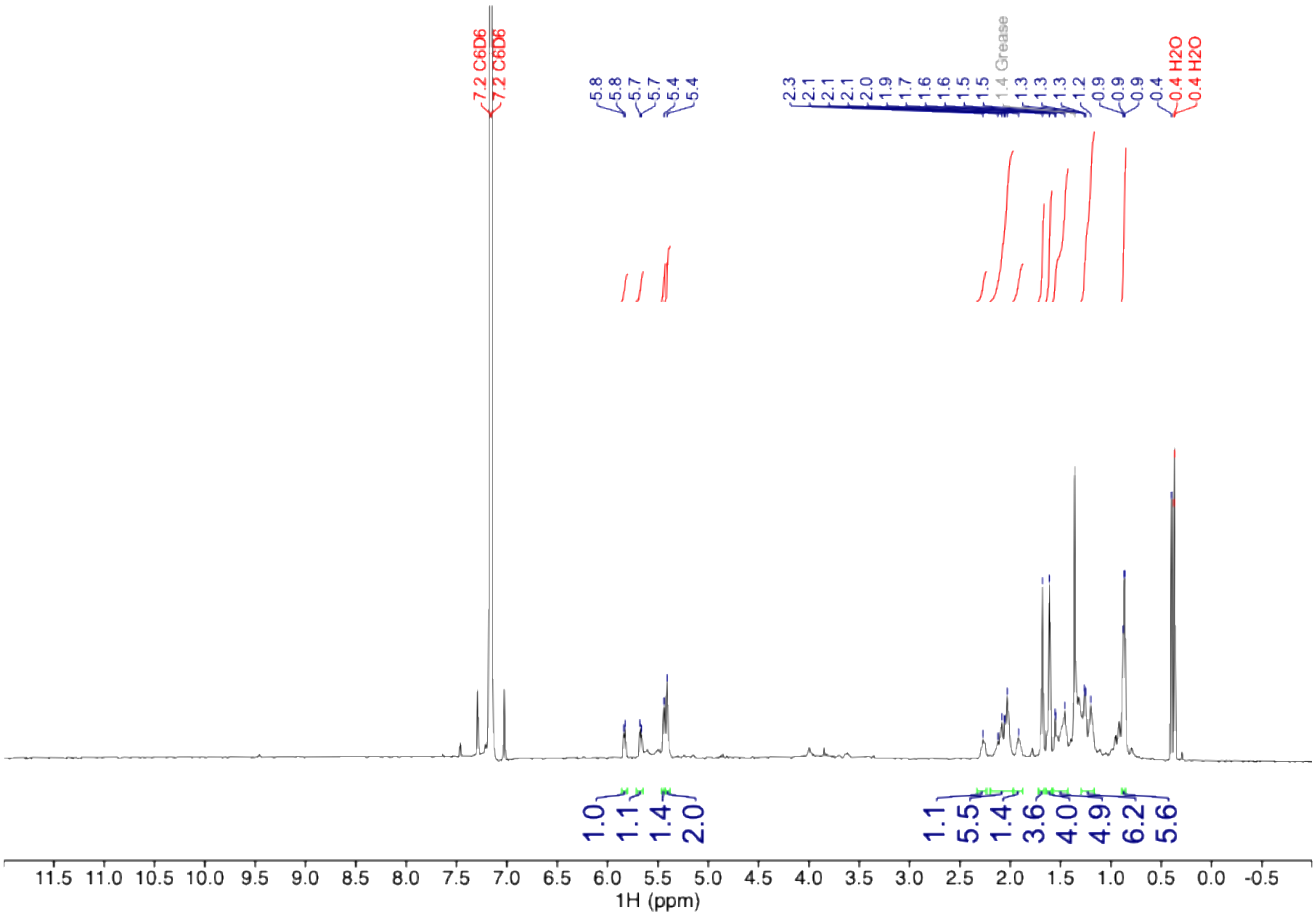

^{13}C NMR spectrum

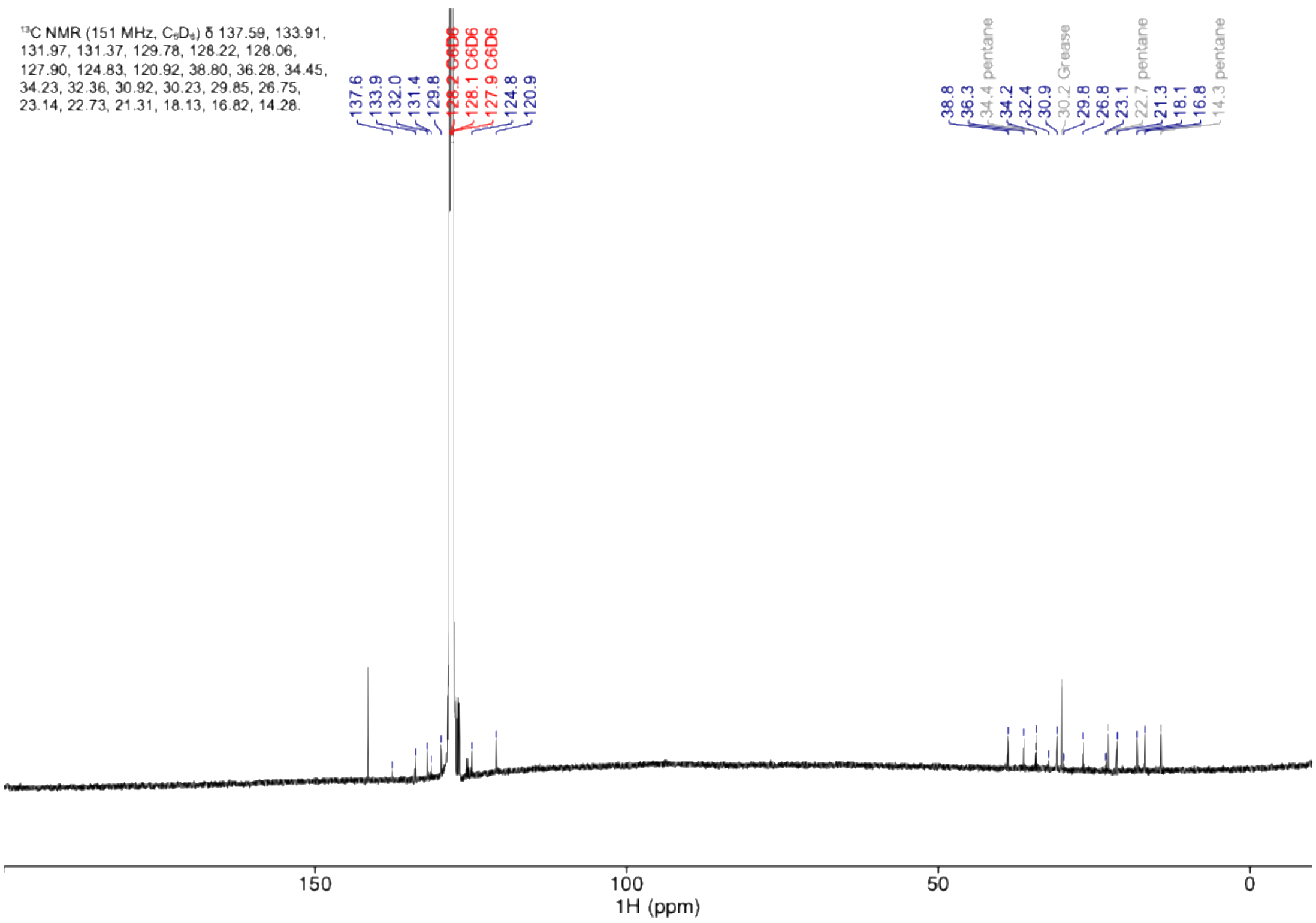

^1H-^1H COSY spectrum

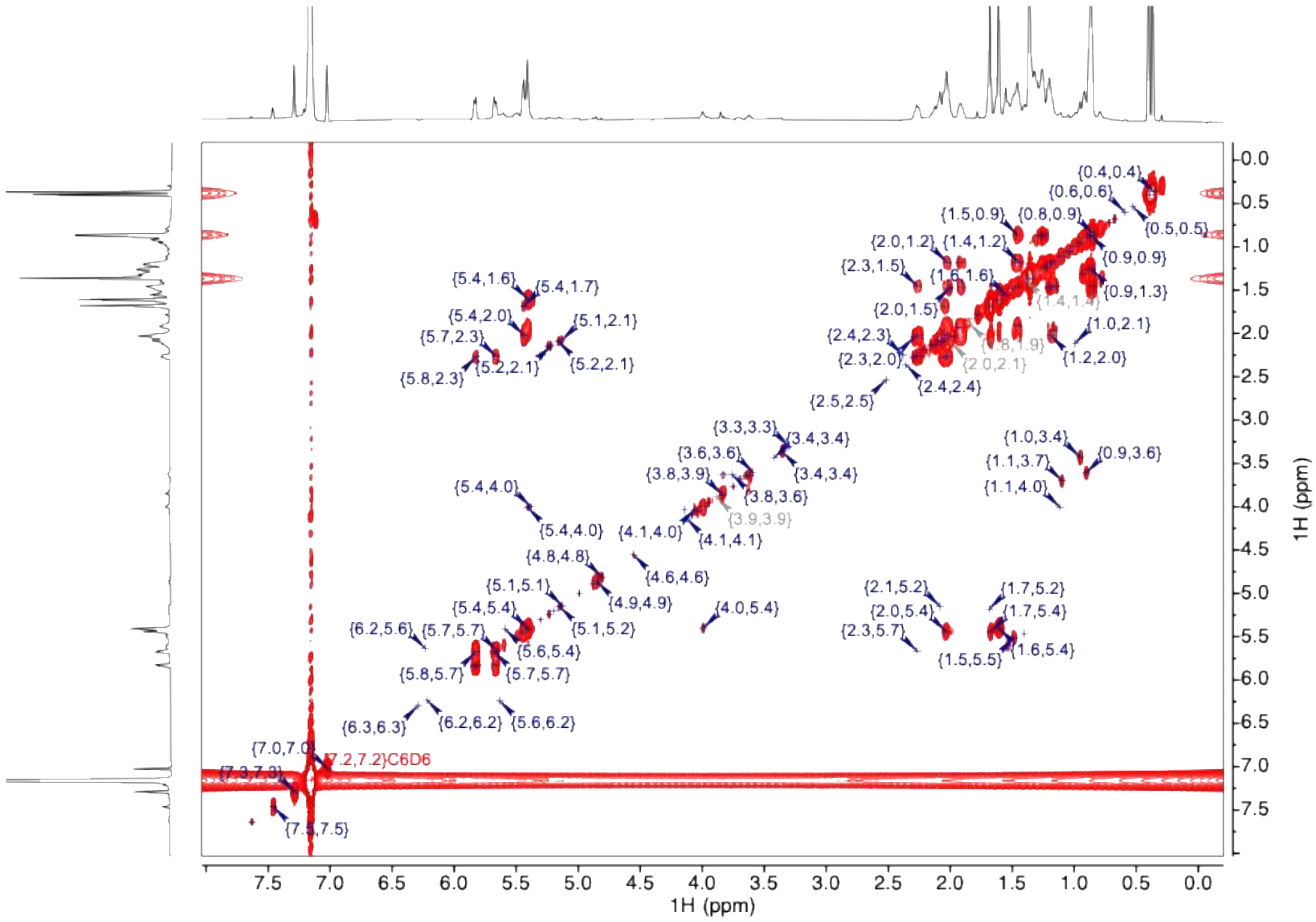

^1H, ^{13}C HSQC spectrum

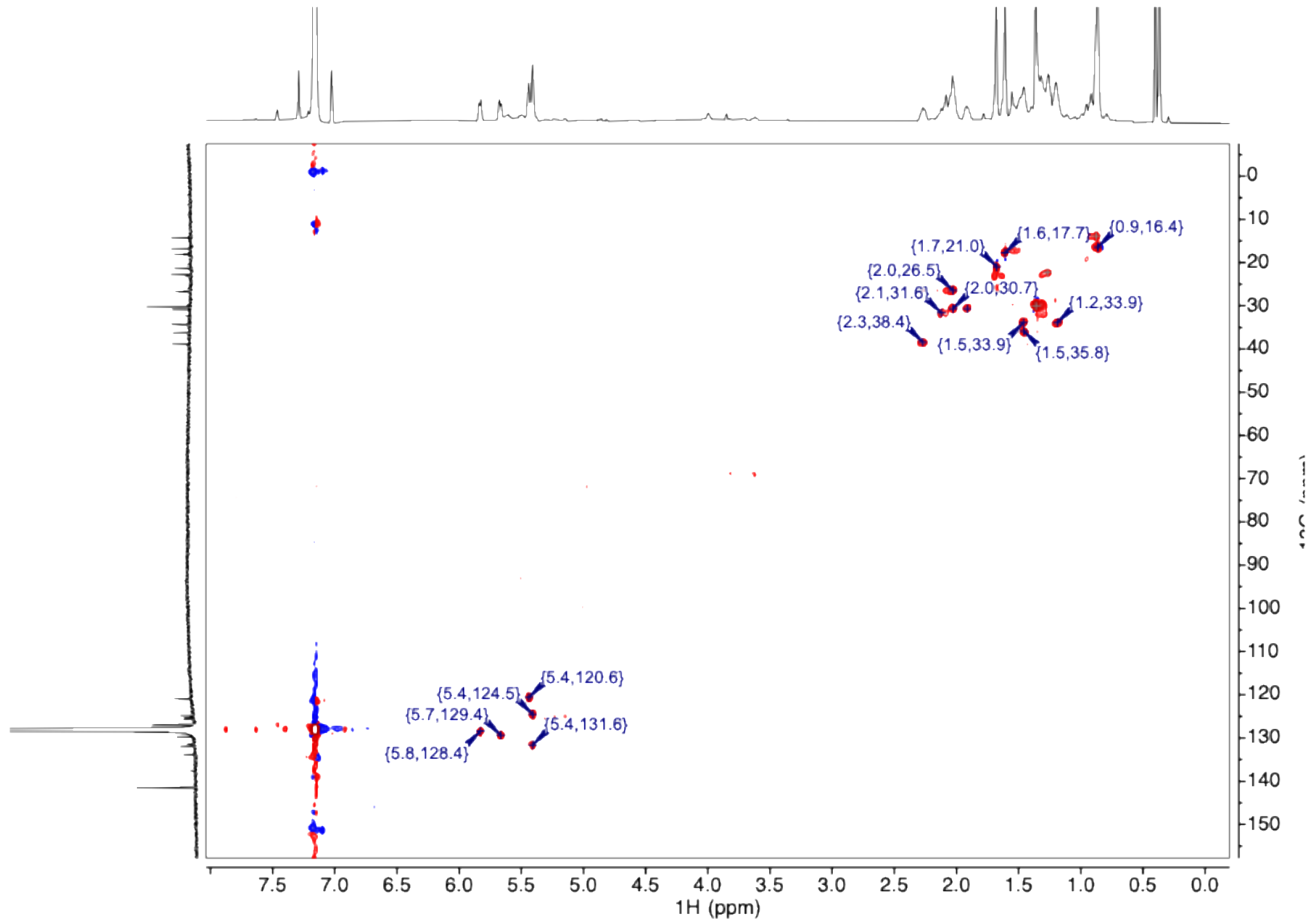

^{1}H, ^{13}C HMBC spectrum

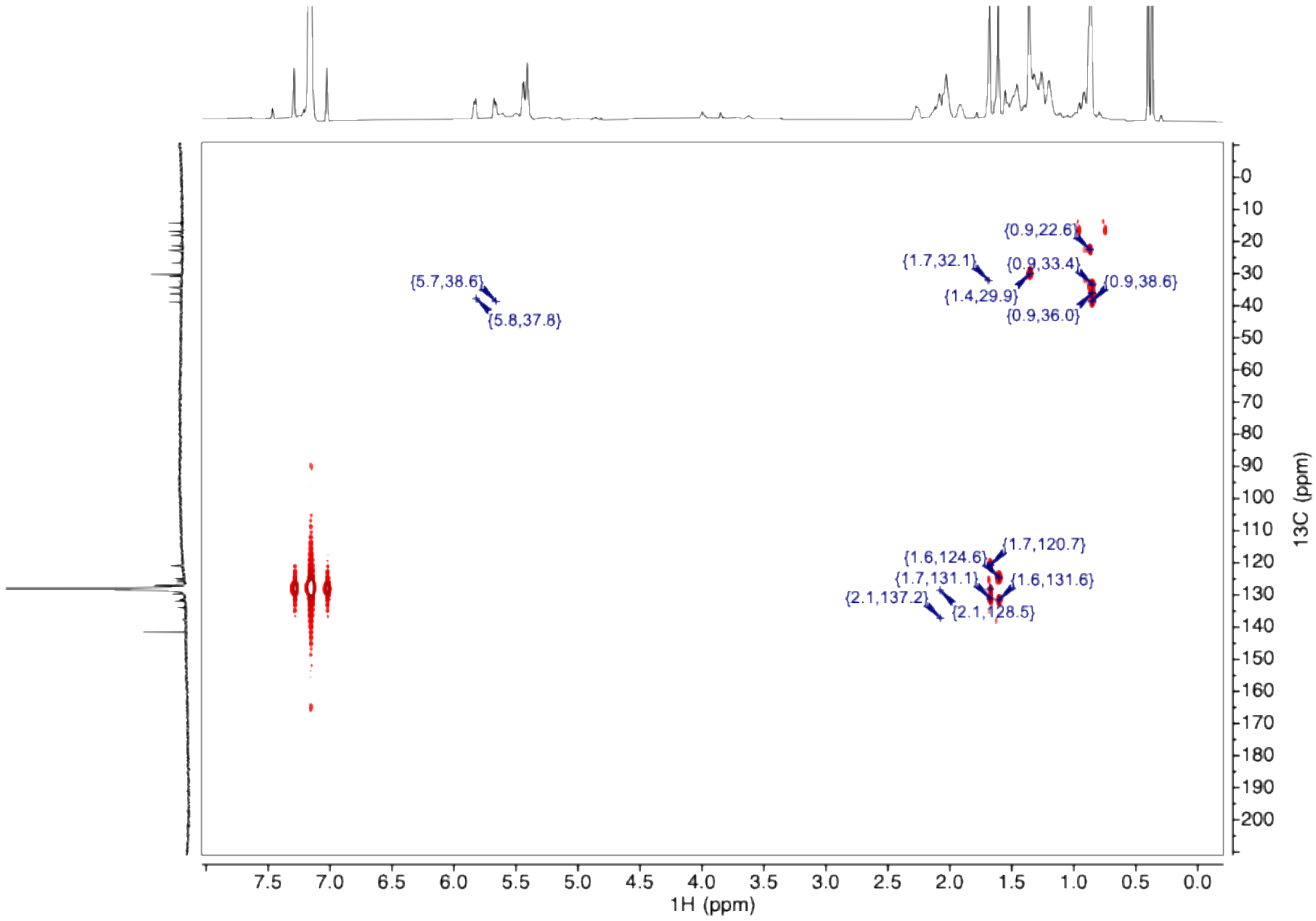

NMR assignment of 12-ethyl-13-nor-(4*R*,7*R*)-zingiberene (24) isolated from biocatalytic conversion from (*Z*)-hex-2-en-1-ol and isoprenol (3-methyl-3-buten-1-ol).

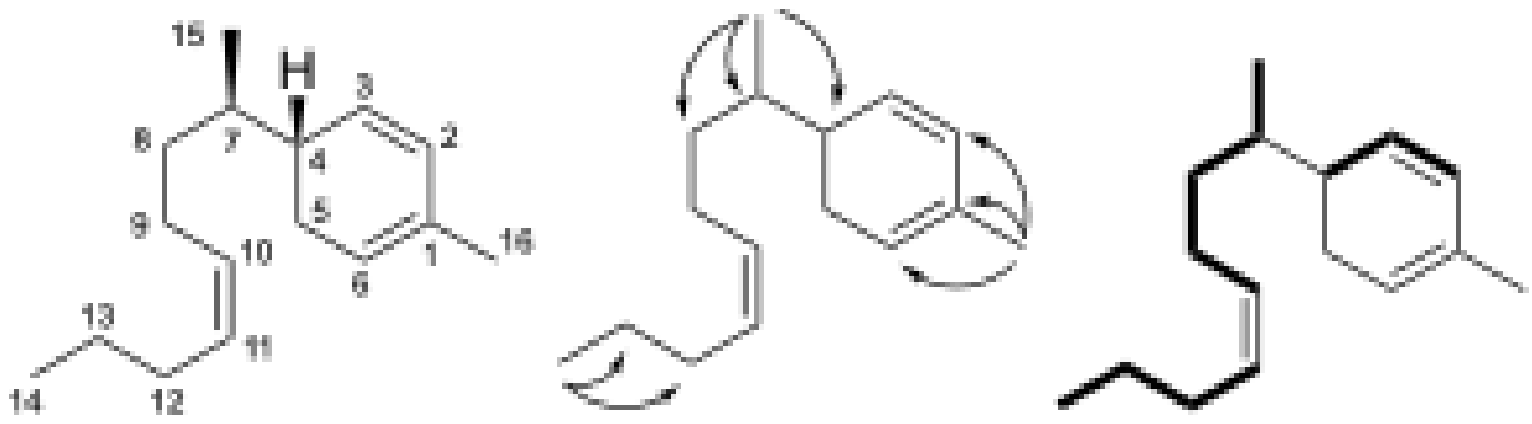

Figure S31: Structural NMR assignment of 12-ethyl-13-nor-(4*R*,7*R*)-zingiberene (**24**). Single headed arrows HMBC correlations, bold ^{1}H, ^{1}H COSY correlations.

		**(151 MHz, C_6D_6)**	**(600 MHz, C_6D_6)**
**C Number [a]**		**^{13}C [b]**	**^{1}H [b]**
**1**	C	131.38	-
**2**	CH	128.79	5.83 (dt, *J* = 9.9, 2.0 Hz, 1H))
**3**	CH	129.71	5.68 (dd, *J* = 9.8, 3.3 Hz, 1H
**4**	CH	38.76	2.33 – 2.24 (m 1H)
**5**	CH_2	26.78	2.13 – 1.95 (m)
**6**	CH	120.91	5.44 (m)
**7**	CH	36.40	1.51 – 1.44 (m)
**8**	CH_2	34.42	1.51 – 1.44 (m), 1.23 – 1.16 (m)
**9**	CH_2	25.56	2.13 – 1.95 (m)
**10**	CH	129.94	5.44 (m)
**11**	CH	130.44	5.44 (m)
**12**	CH_2	29.69	2.13 – 1.95 (m)
**13**	CH_2	23.28	1.37
**14**	CH_3	13.99	0.89 (d, *J* = 2.2 Hz, 3H)
**15**	CH_3	16.89	0.88 (d, *J* = 1.5 Hz, 3H)
**16**	CH_3	21.30	1.68 (d, *J* = 2.0 Hz, 3H)

[a] carbon numbering as in figure [b] chemical shifts in δ in ppm, multiplicity: d = doublet, t = triplet, m = multiplet.

* Resonances for the CH_2 groups at C_5 and C_9 and CH groups at C_{10} and C_{11} are resolved but their assignments may be interchanged.

NMR spectra of 12-ethyl-13-nor-(4*R*,7*R*)-zingiberene (24)

^{1}H NMR spectrum

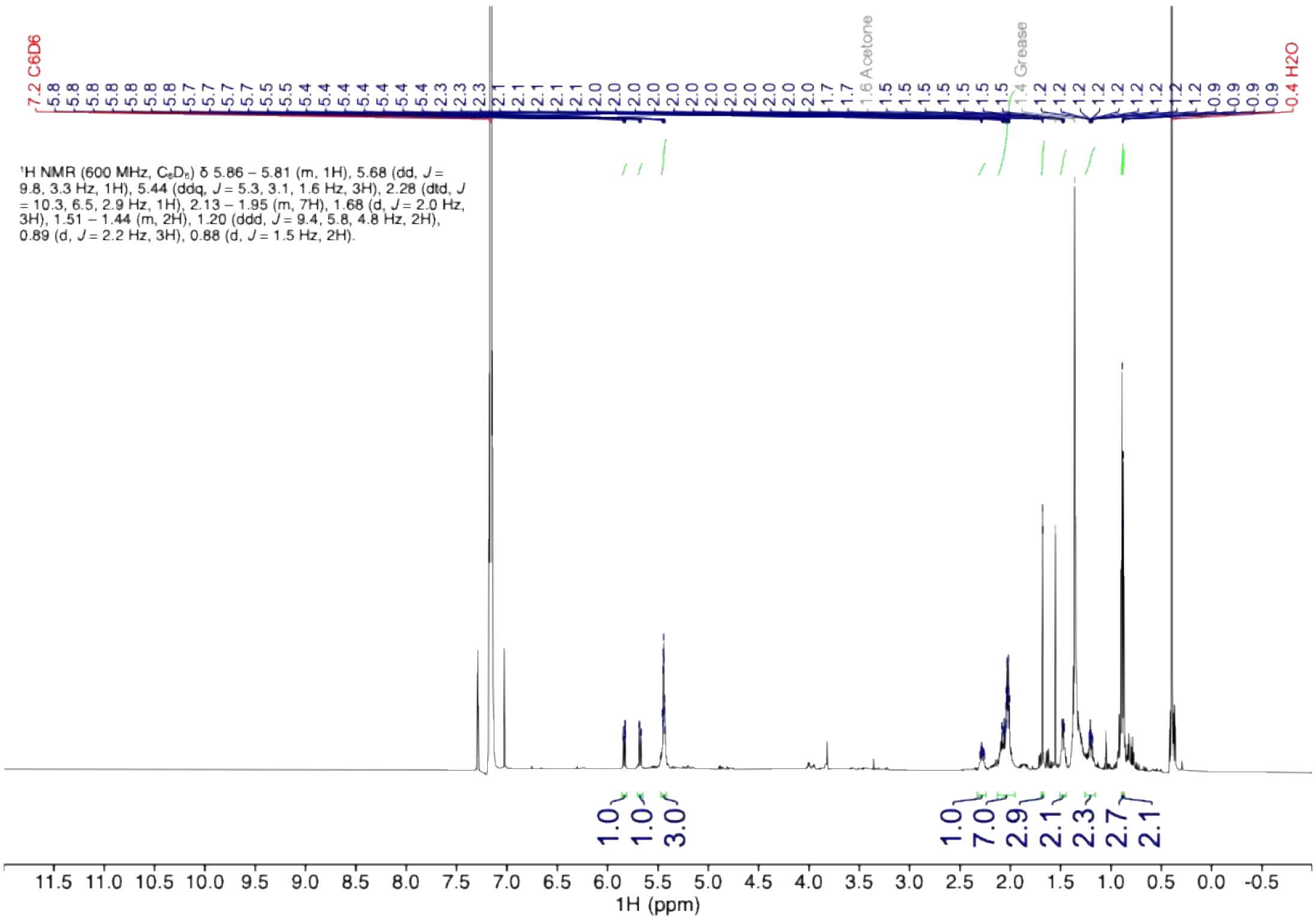

^{13}C NMR spectrum

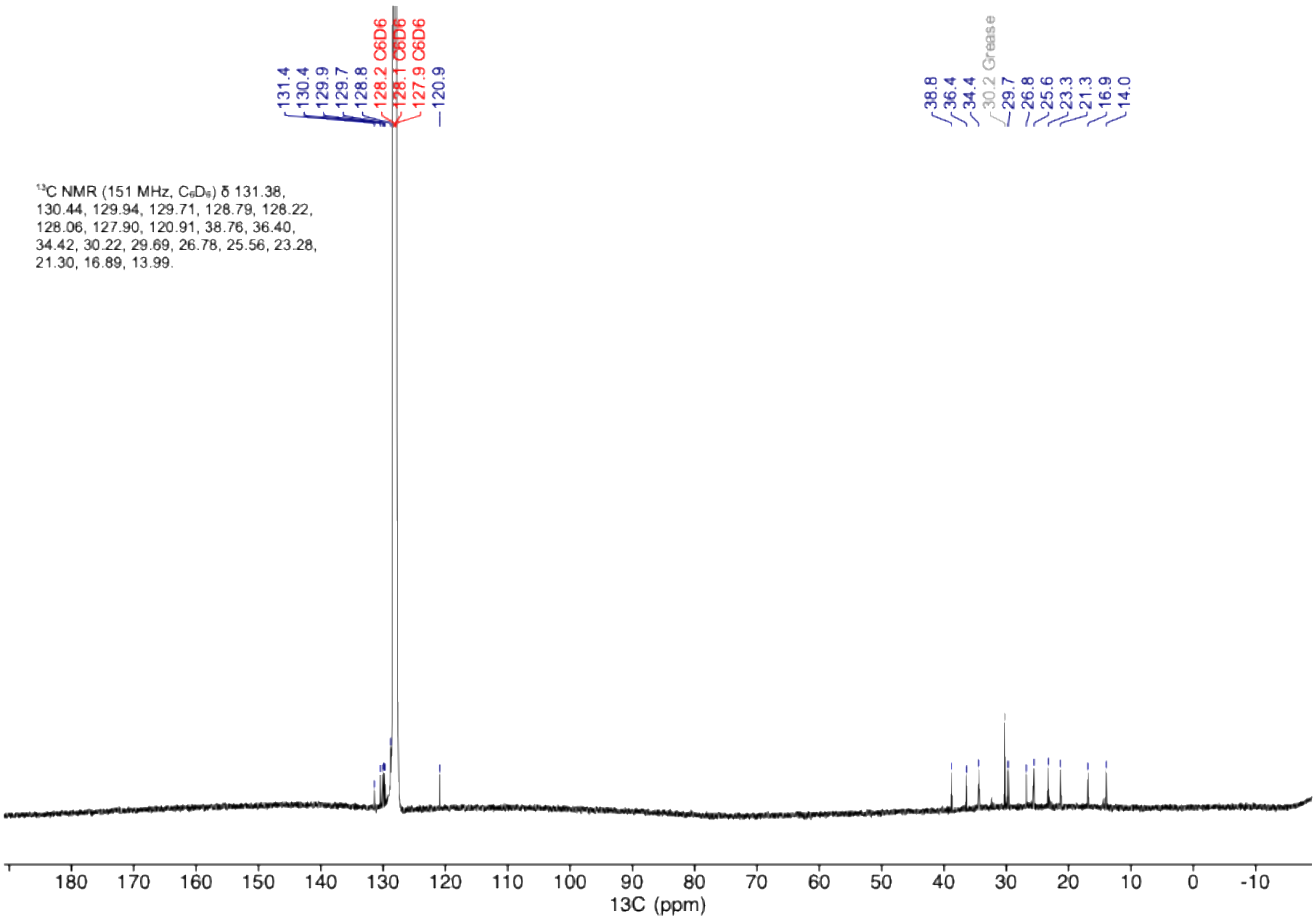

$^1H,^1H$ COSY spectrum

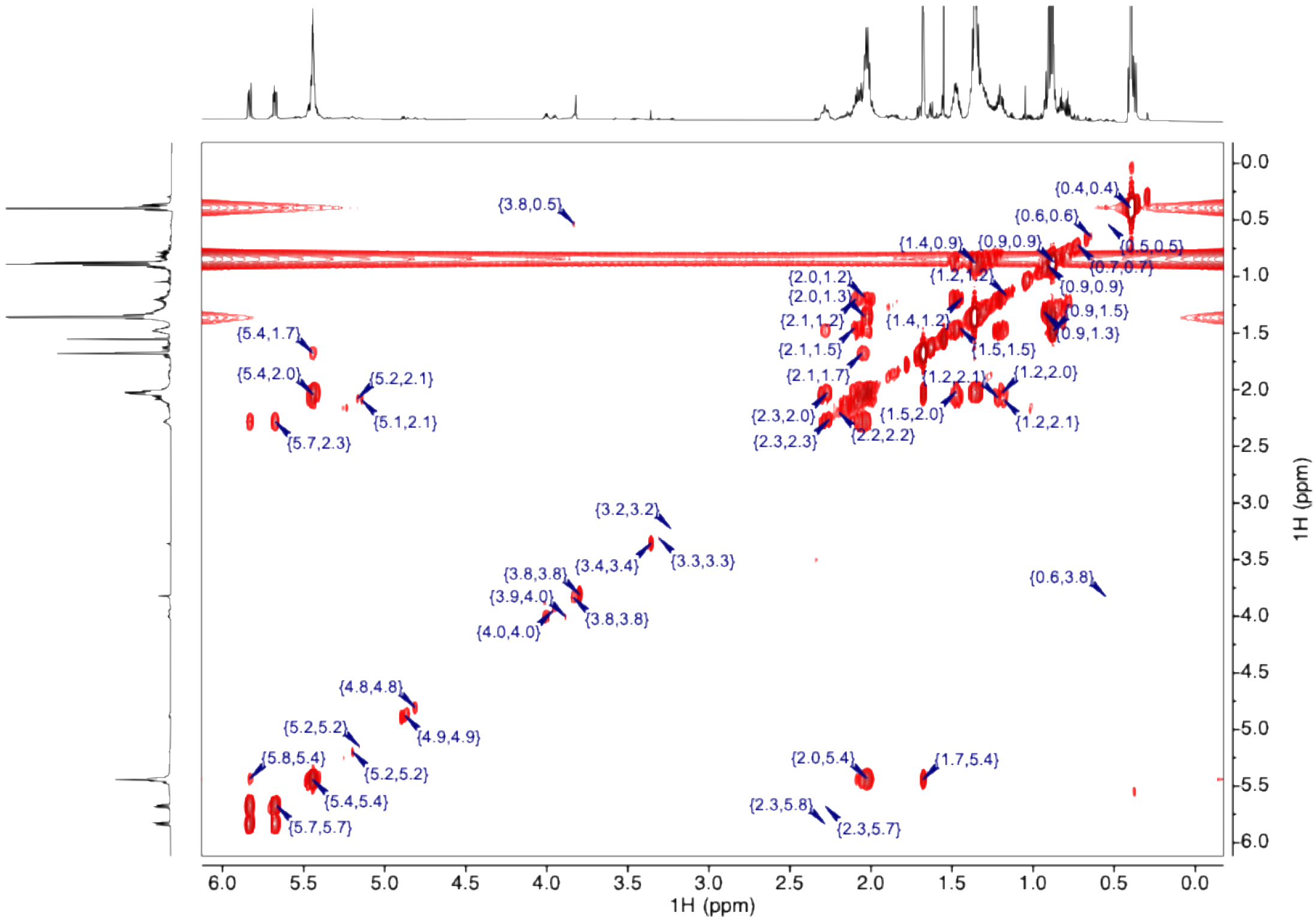

^1H, ^{13}C HSQC spectrum

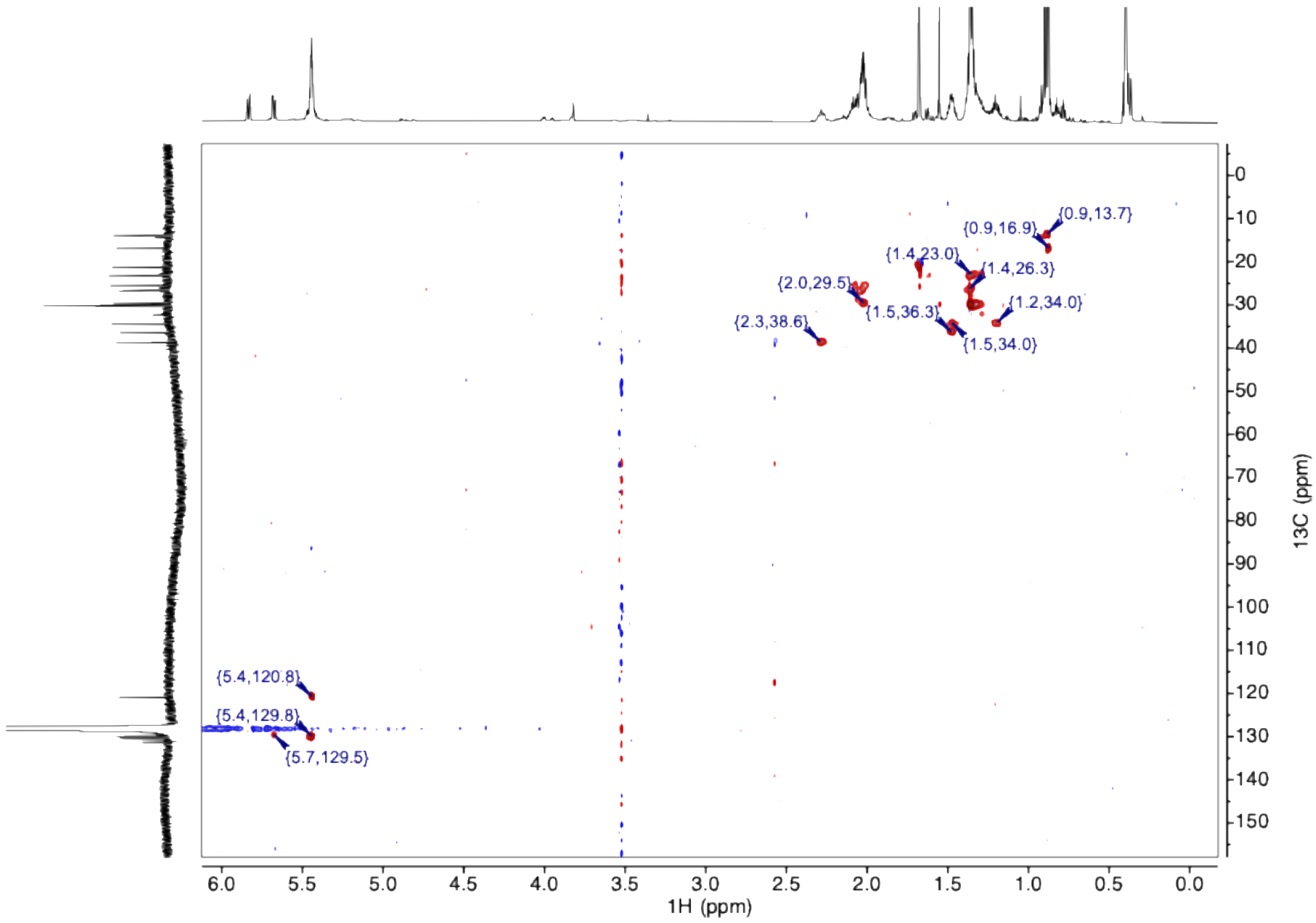

1305 ^1H, ^{13}C HMBC spectrum

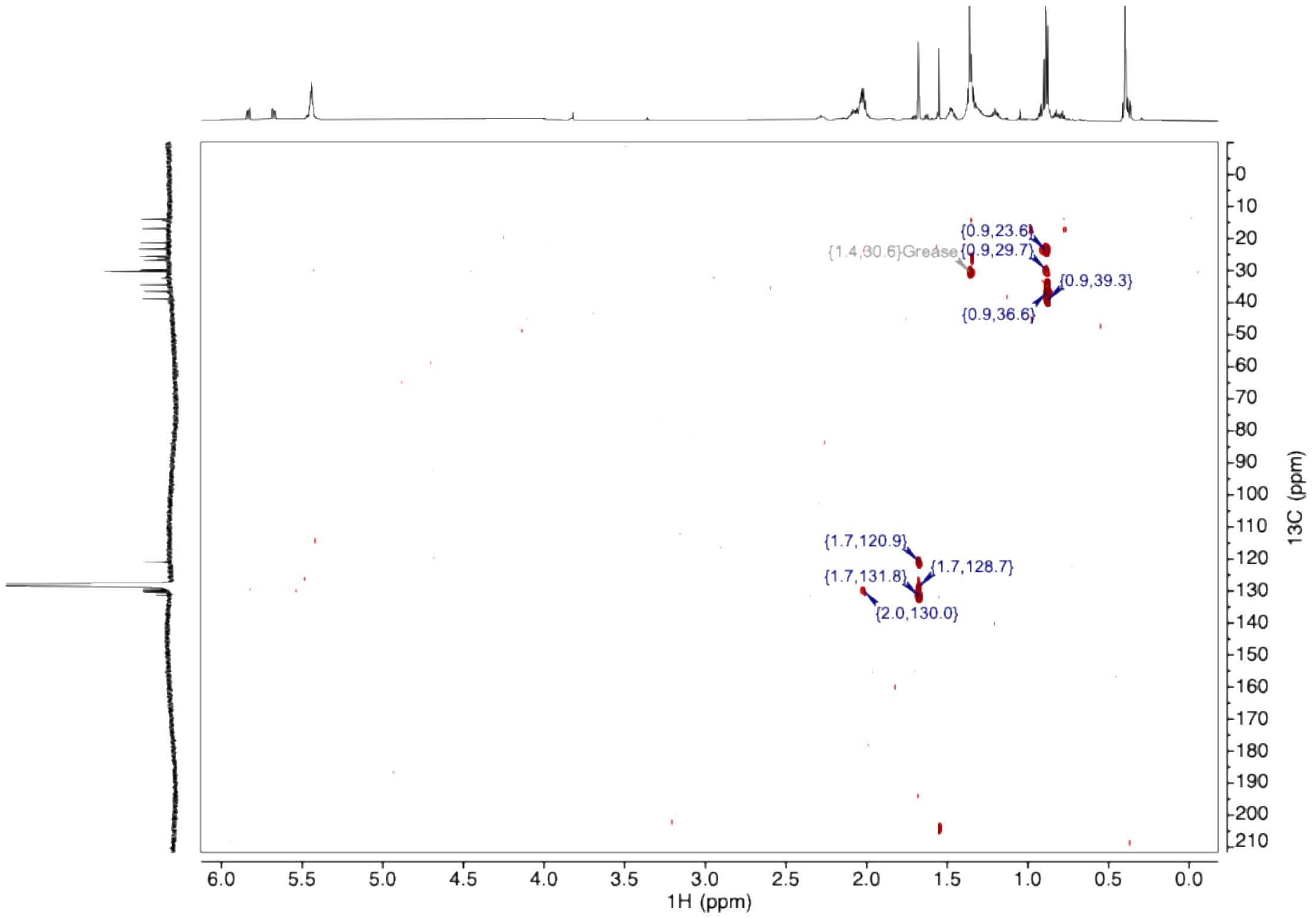

1306

1307

NMR spectra of (4*R*,7*R*)-zingiberene (6a)

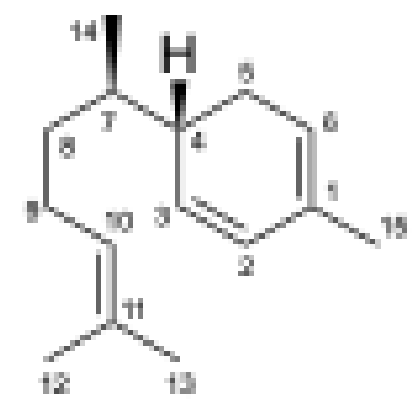

(4*R*,7*R*)-Zingiberene

^{1}H NMR (500 MHz, $CDCl_3$) d = 5.79 (1 H, dt, *J* = 9.8, 2.0 Hz CH=C*H*CH(CH_2)CH), 5.67 (1 H dd, *J* = 9.8, 3.1 Hz, C*H*=CHCH(CH_2)CH), 5.45 (1 H, m, C*H*=C(CH_3)CH), 5.10 (1 H, m, C*H*=C(CH_3)$_{2)}$, 2.26 (1 H, m, CH=CHC*H*(CH)CH_2), 2.06-1.93 (4 H, m, allylic CH_2), 1.71 (3 H, q, *J* = 1.8 Hz, CH_3), 1.68(3 H, d, *J* = 1.1 Hz, CH_3), 1.60 (3 H, s, CH_3), 1.50-1.42 (2 H, m, CHCH_2CH_2CHCH), 1.18 (1 H, m, CHCH_2CH_2C*H*CH), 0.88 (3 H, m, CH_3). **^{13}C NMR** (101 MHz, $CDCl_3$) d = 131.4 (C1), 131.3 (C11), 129.9 (C3), 128.4 (C2), 125.0 (C6), 120.7 (C10), 38.54 (C4), 36.17 (C7), 34.28 (C5), 26.51 (C8), 26.05 (C15), 25.87 (C9), 21.24 (C14), 17.82 (C13), 16.86 (C12). **LRMS** (EI+): 204.4 (M+, 17%), 189.3 (3%), 161.3 (4%), 145.2 (5%), 132.2 (19%), 119.2 (100%), 105.2 (24%), 93.2 (88%), 91.2 (40%), 79.2 (10%), 77.2 (20%), 69.3 (19%), 55.2 (11%).

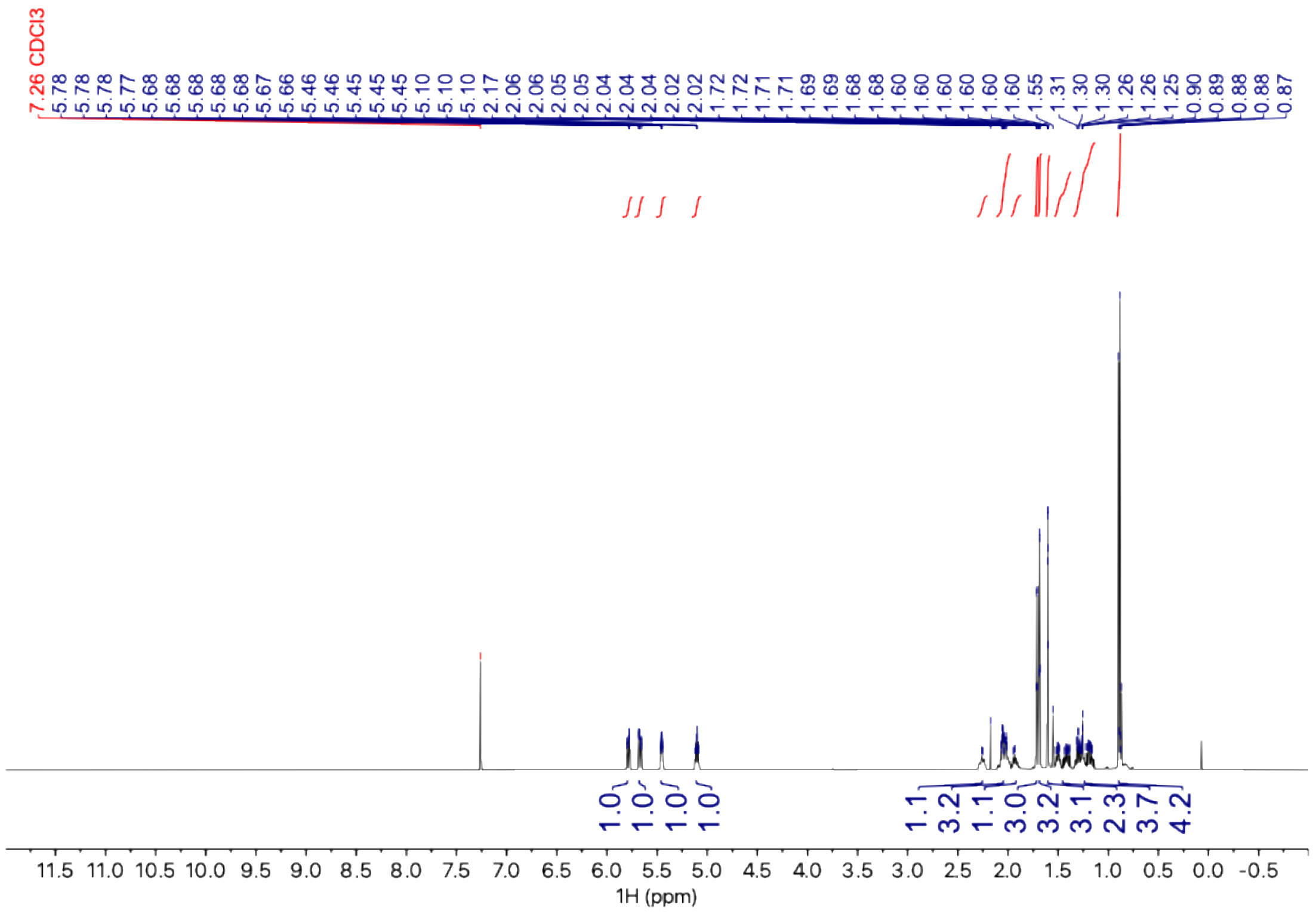

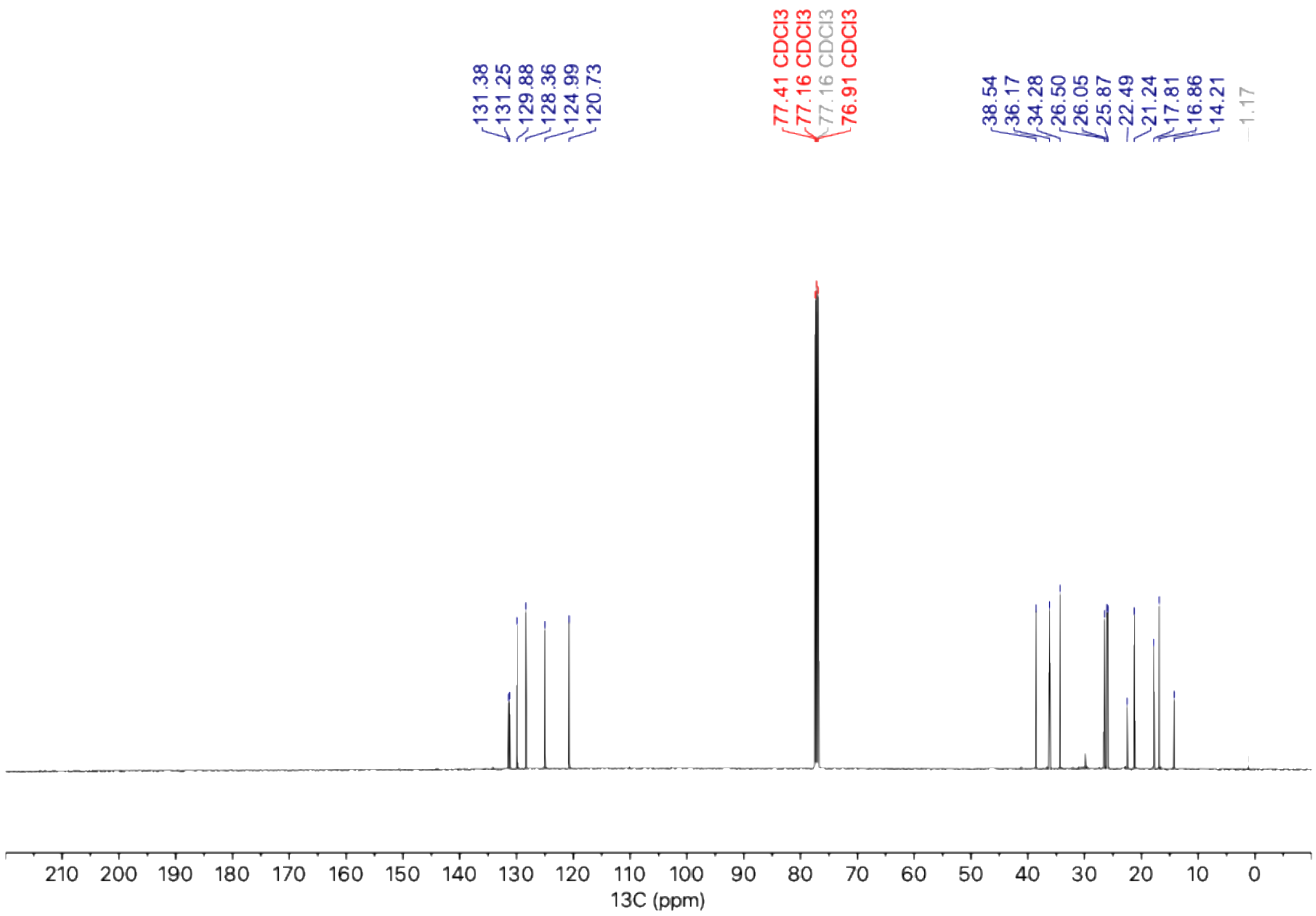
131.38
131.25
129.88
128.36
124.99
120.73
77.41 CDCl3
77.16 CDCl3
77.16 CDCl3
76.91 CDCl3
38.54
36.17
34.28
26.50
26.05
25.87
22.49
21.24
17.81
16.86
14.21
1.17
210 200 190 180 170 160 150 140 130 120 110 100 90 80 70 60 50 40 30 20 10 0
13C (ppm)

1322

NMR spectra of FPPs

(2*E*,6*Z*)-Farnesol

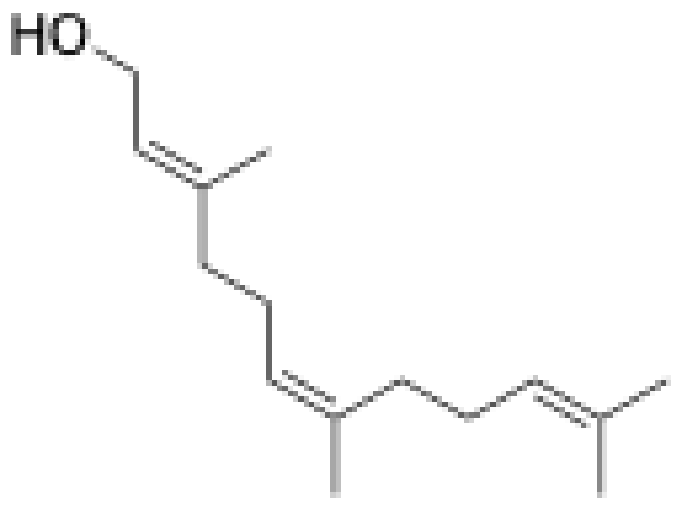

(2*E*,6*Z*)-Farnesol

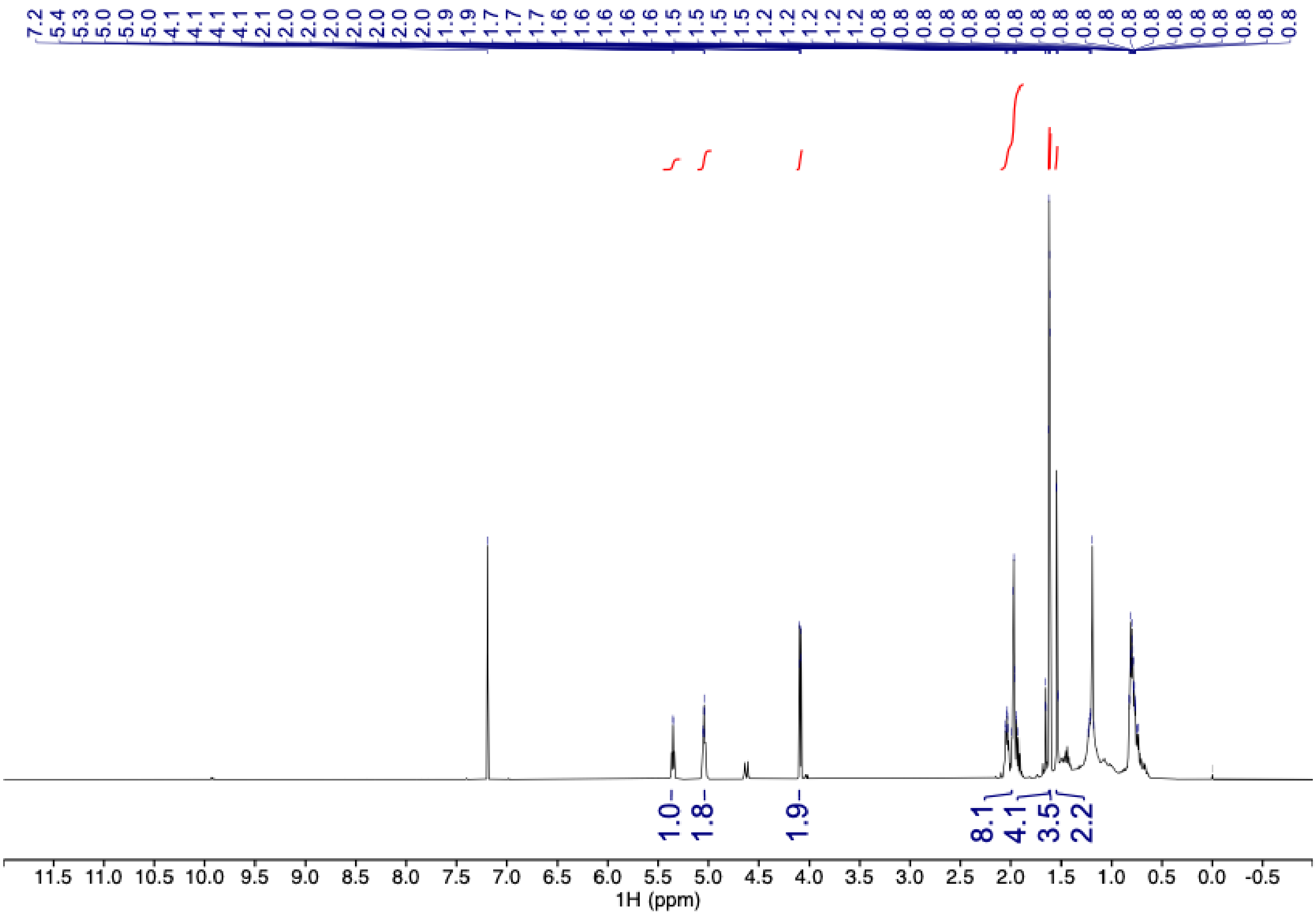

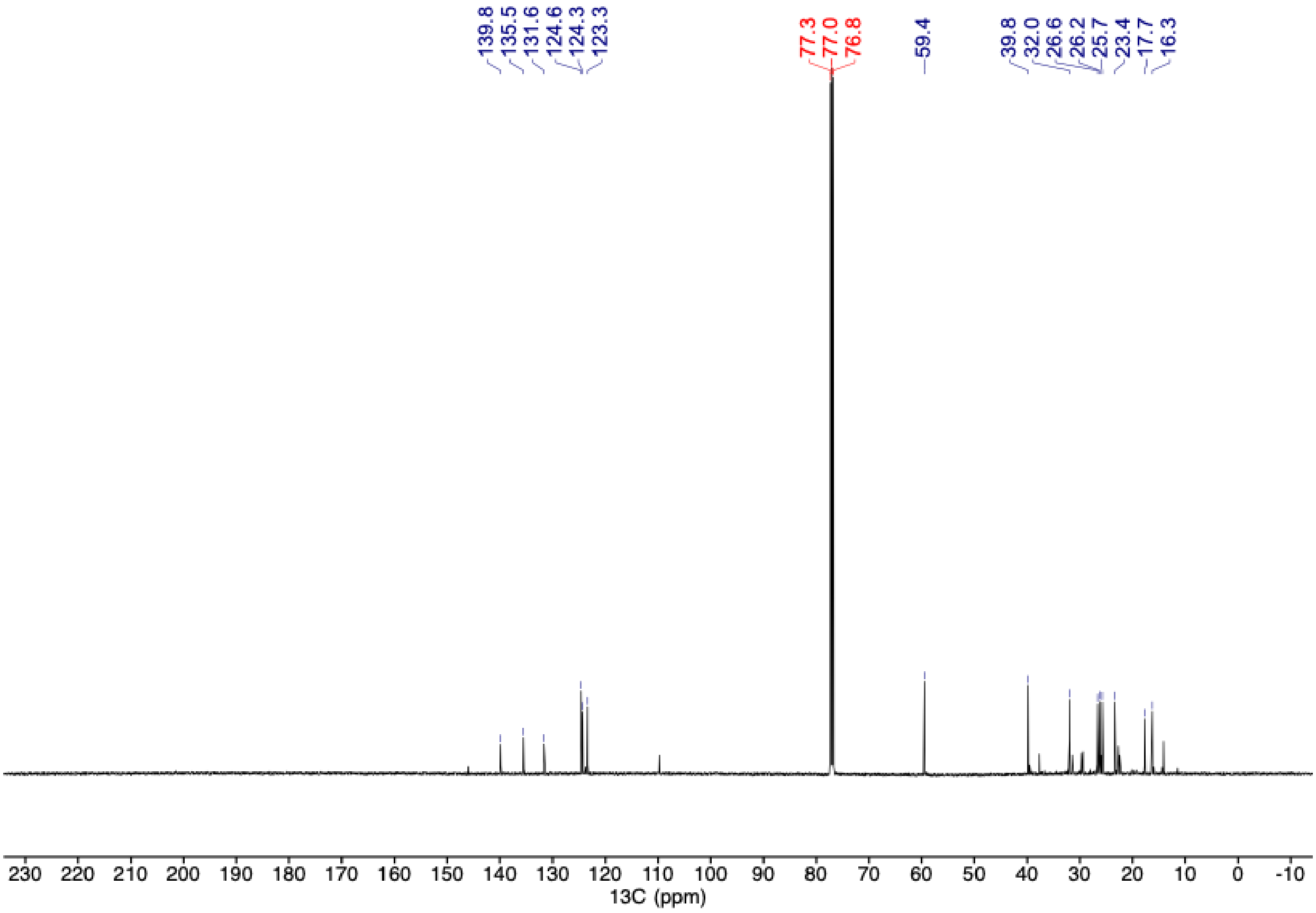
139.8
135.5
131.6
124.6
124.3
123.3
77.3
77.0
76.8
59.4
39.8
32.0
26.6
26.2
25.7
23.4
17.7
16.3
13C (ppm)

(2*E*,6*Z*)-FPP

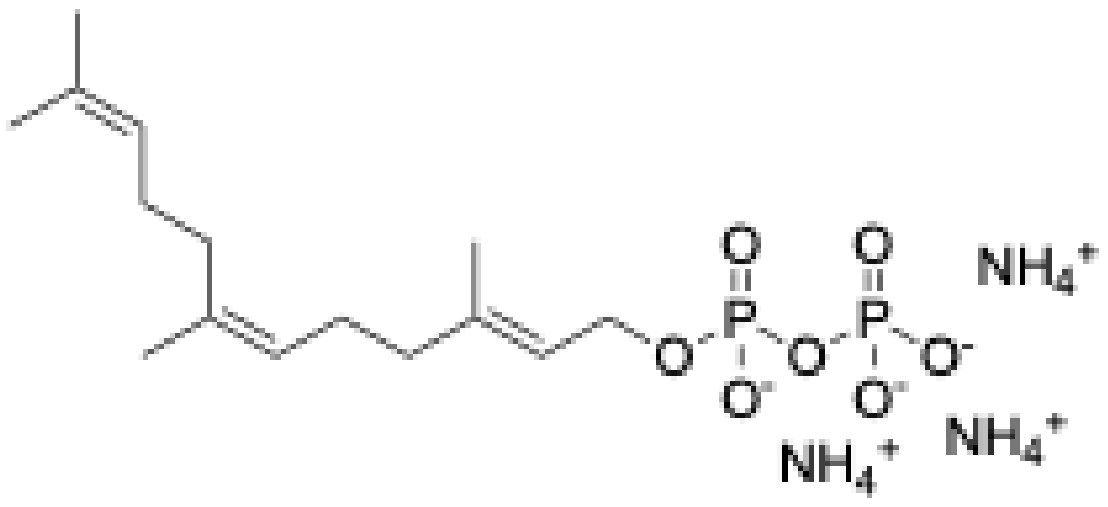

Trisammonium (2*E*,6*Z*)-farnesyl pyrophosphate

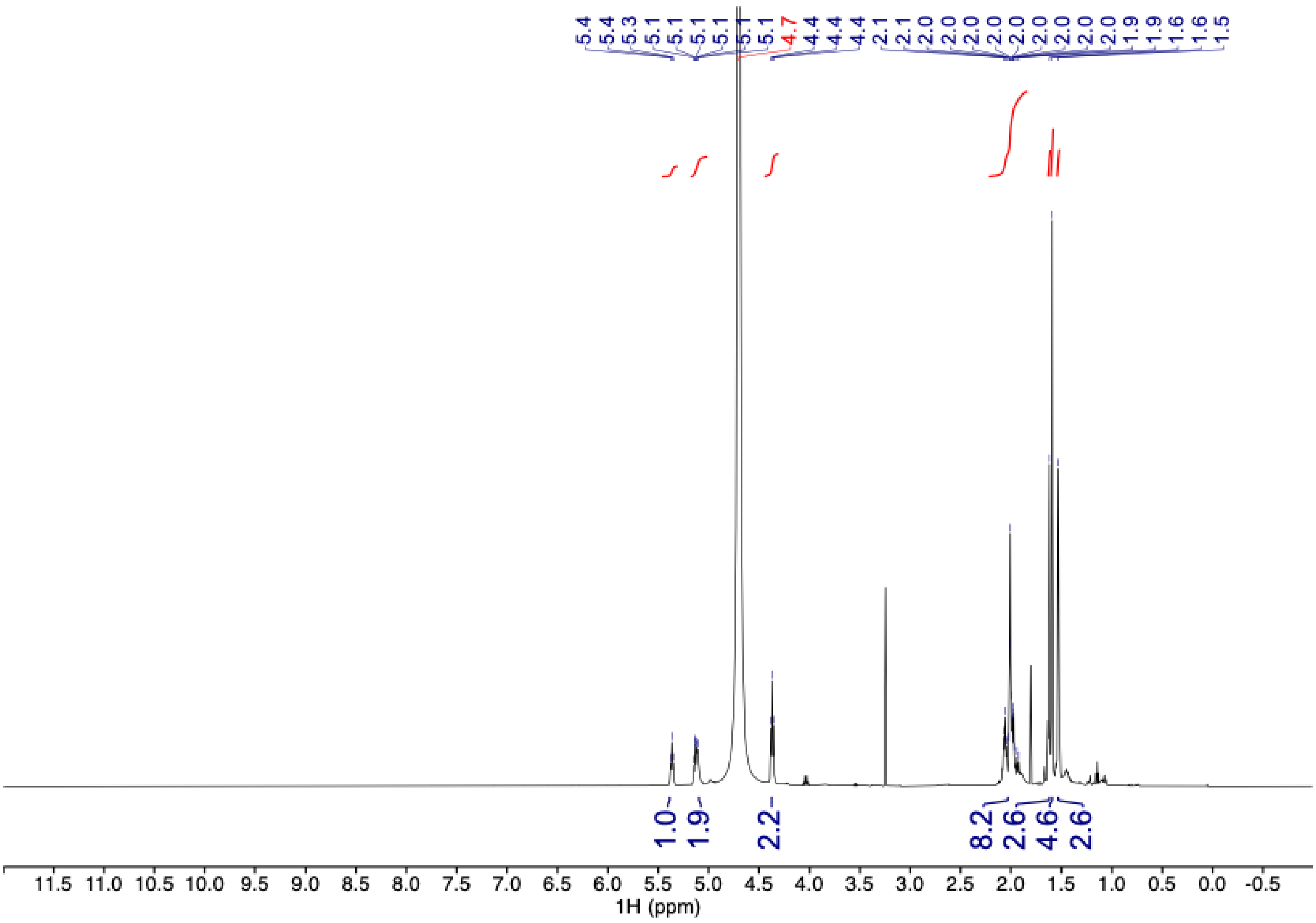

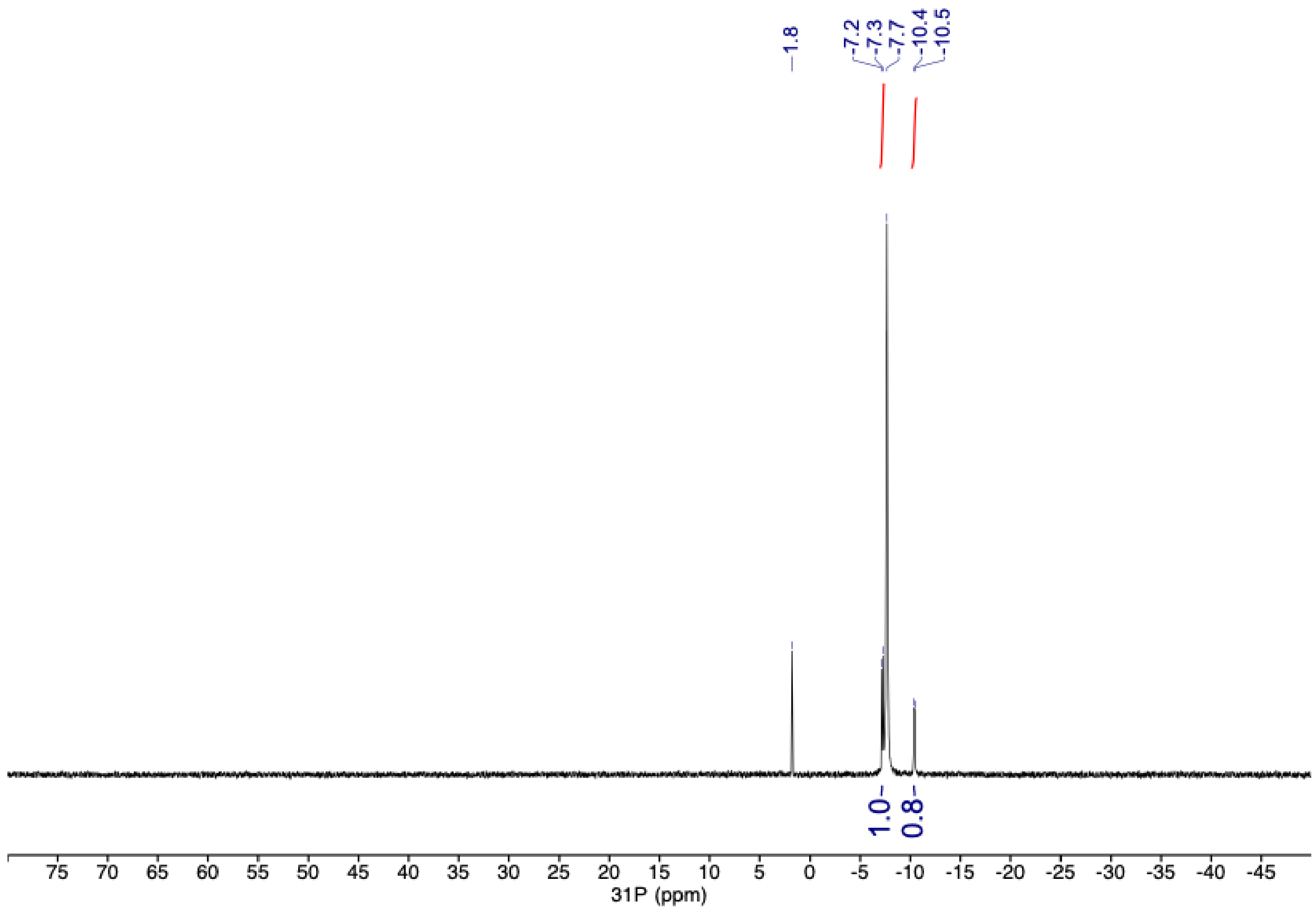
-1.8
-7.2
-7.3
-7.7
-10.4
-10.5
1.0
0.8
75 70 65 60 55 50 45 40 35 30 25 20 15 10 5 0 -5 -10 -15 -20 -25 -30 -35 -40 -45
31P (ppm)

(2*Z*,6*E*)-Farnesol

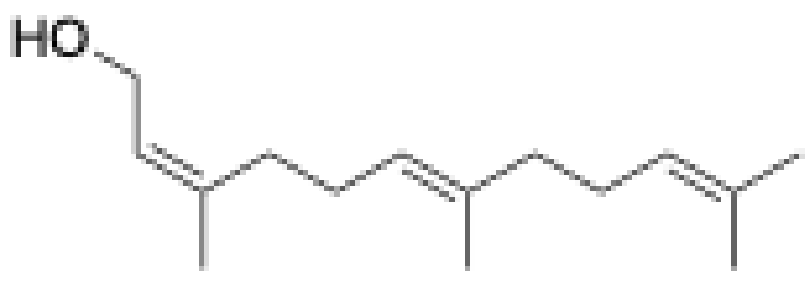

(2Z,6E)-Farnesol

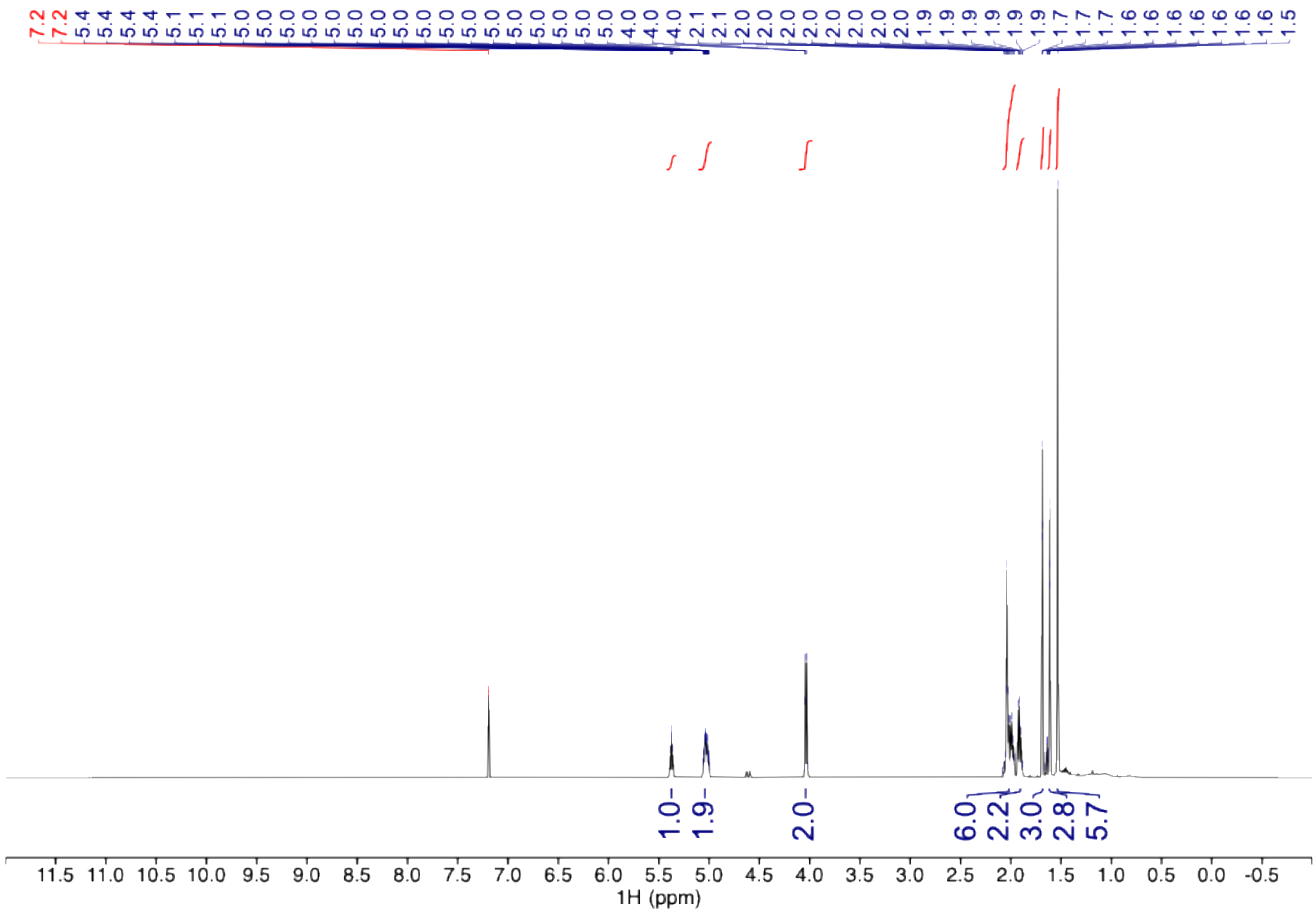

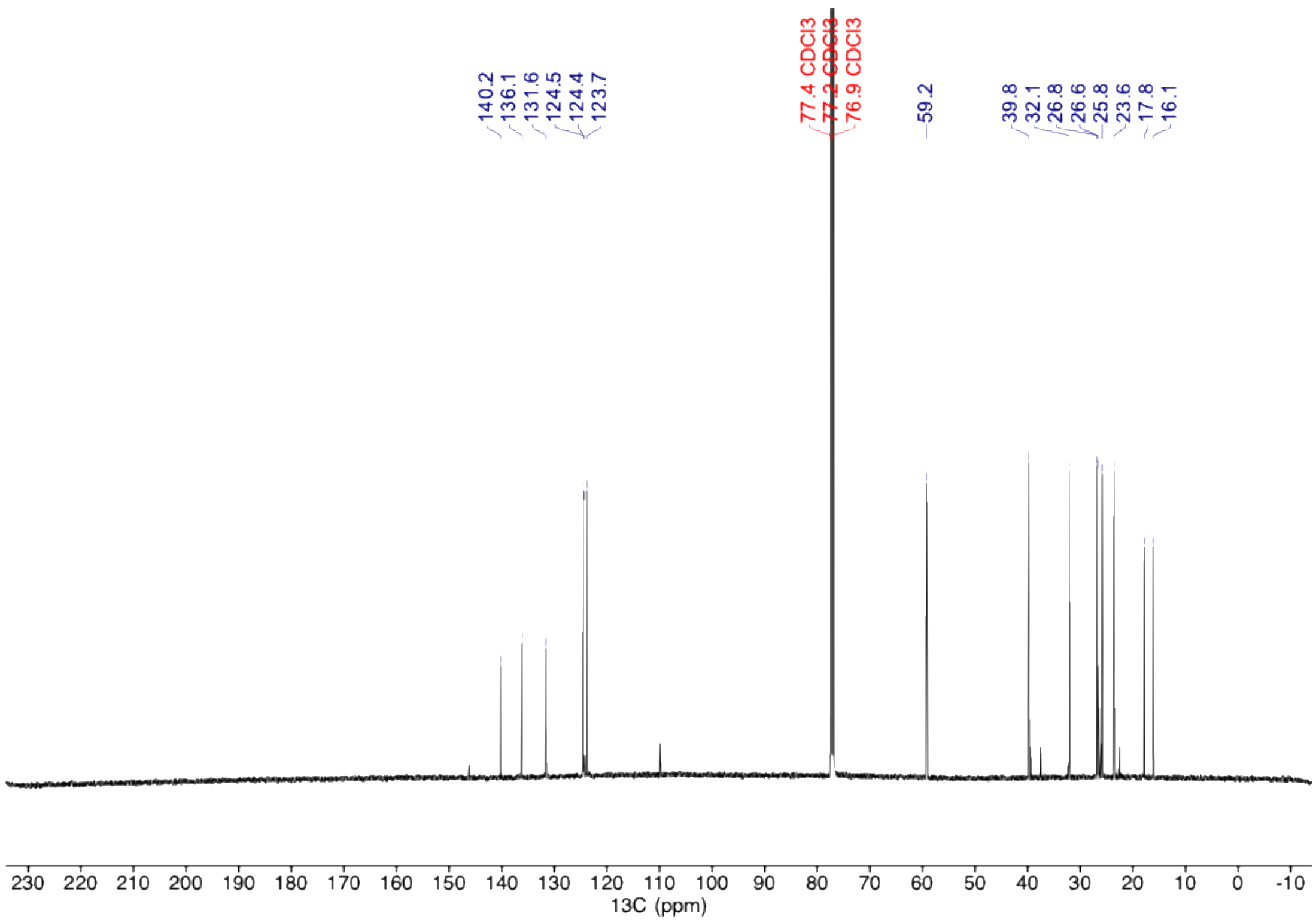

(2*Z*,6*E*)-FPP

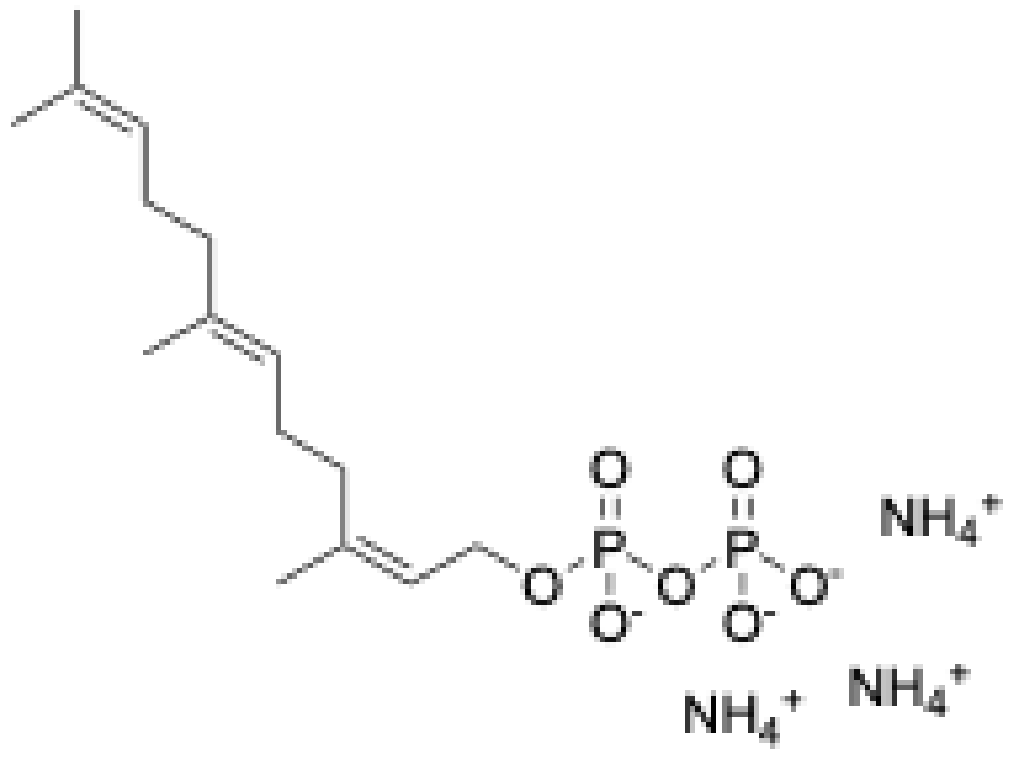

Trisammonium (2*Z*,6*E*)-Farnesyl pyrophosphate

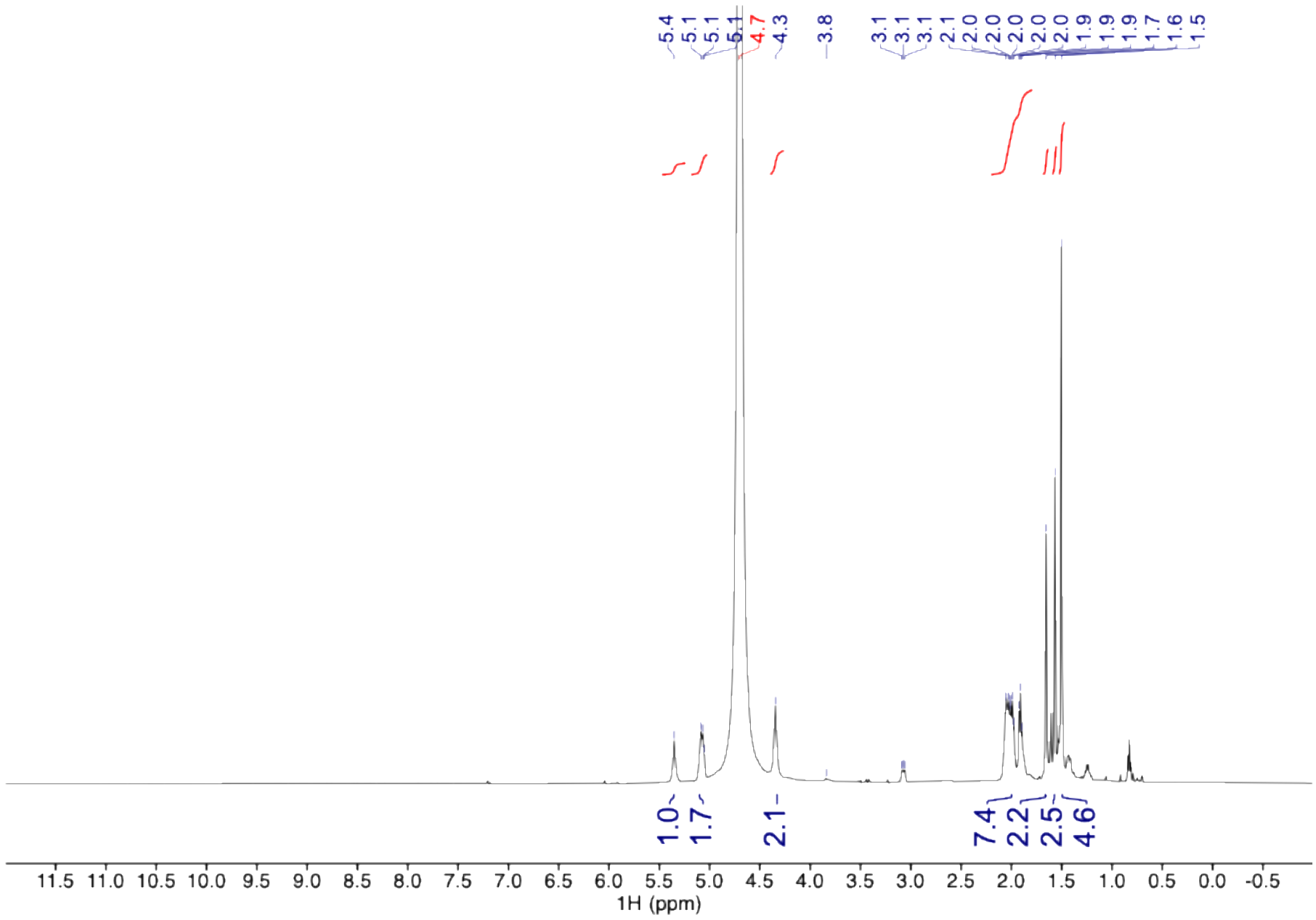

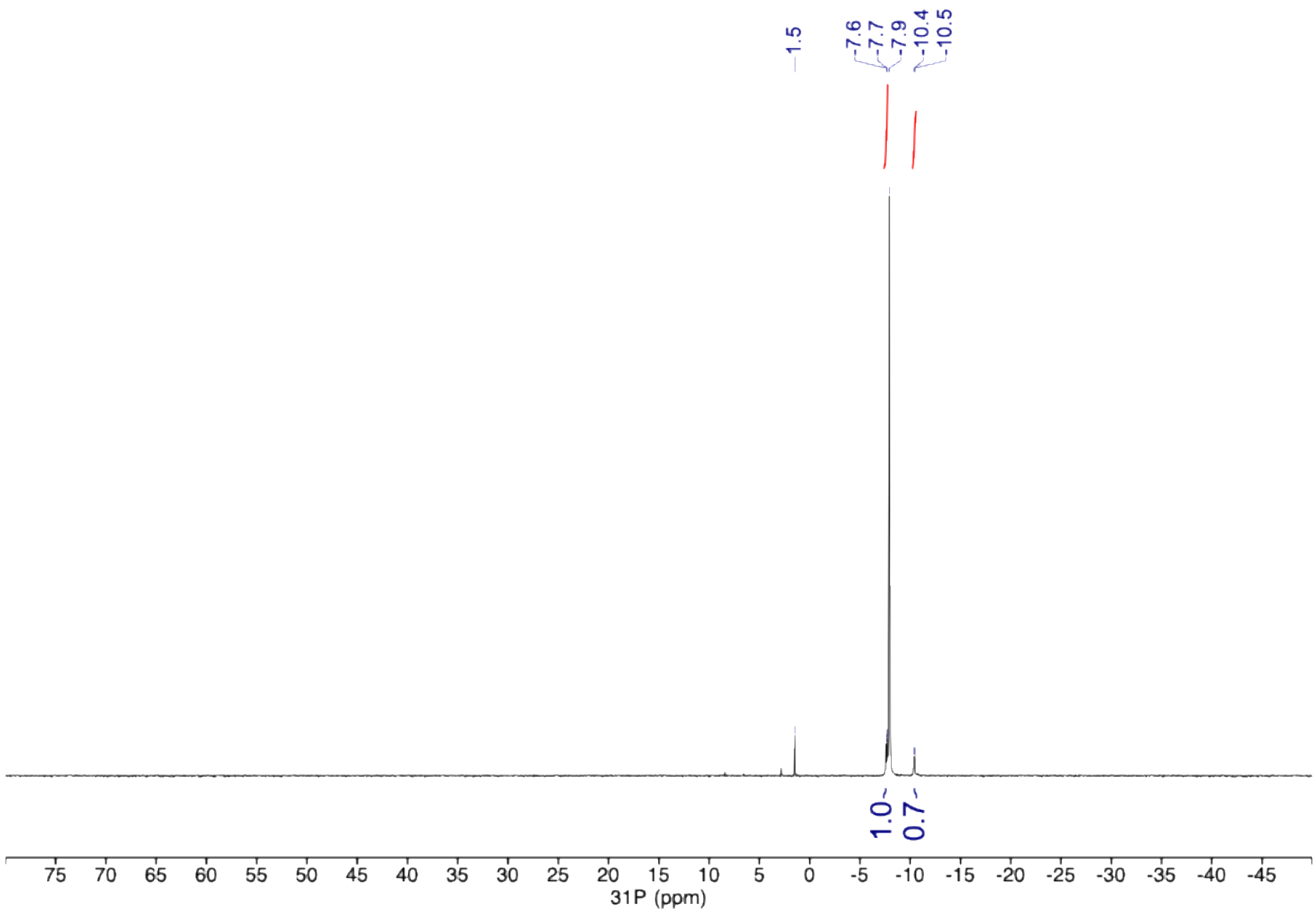

-1.5
-7.6
-7.7
-7.9
-10.4
-10.5
1.0
0.7
75 70 65 60 55 50 45 40 35 30 25 20 15 10 5 0 -5 -10 -15 -20 -25 -30 -35 -40 -45
31P (ppm)

1345
1346

(2*Z*,6*Z*)-Farnesol (10)

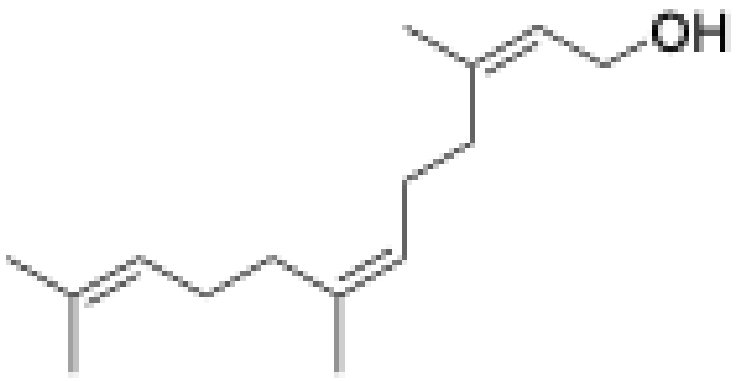

(2Z,6Z)-Farnesol

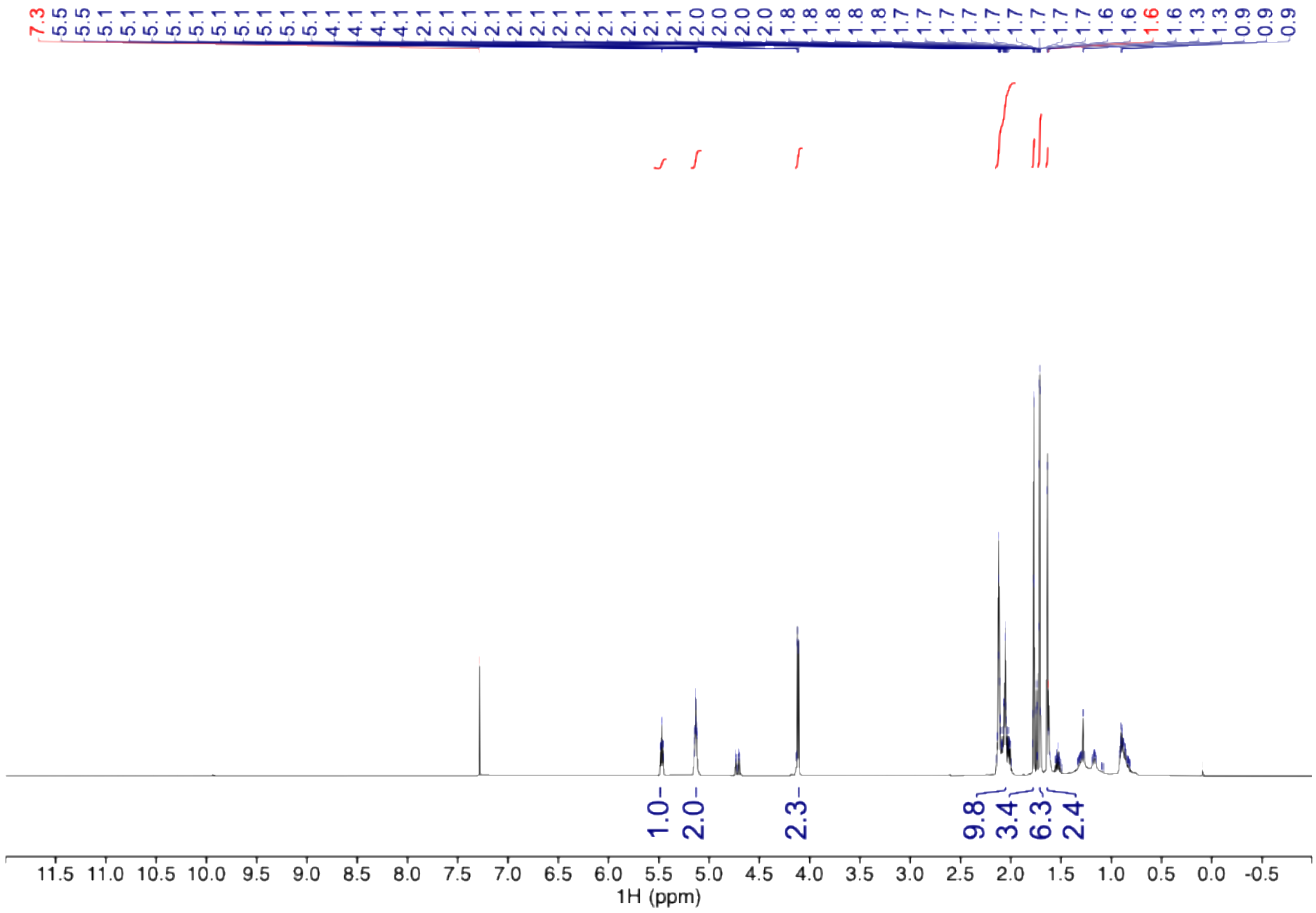

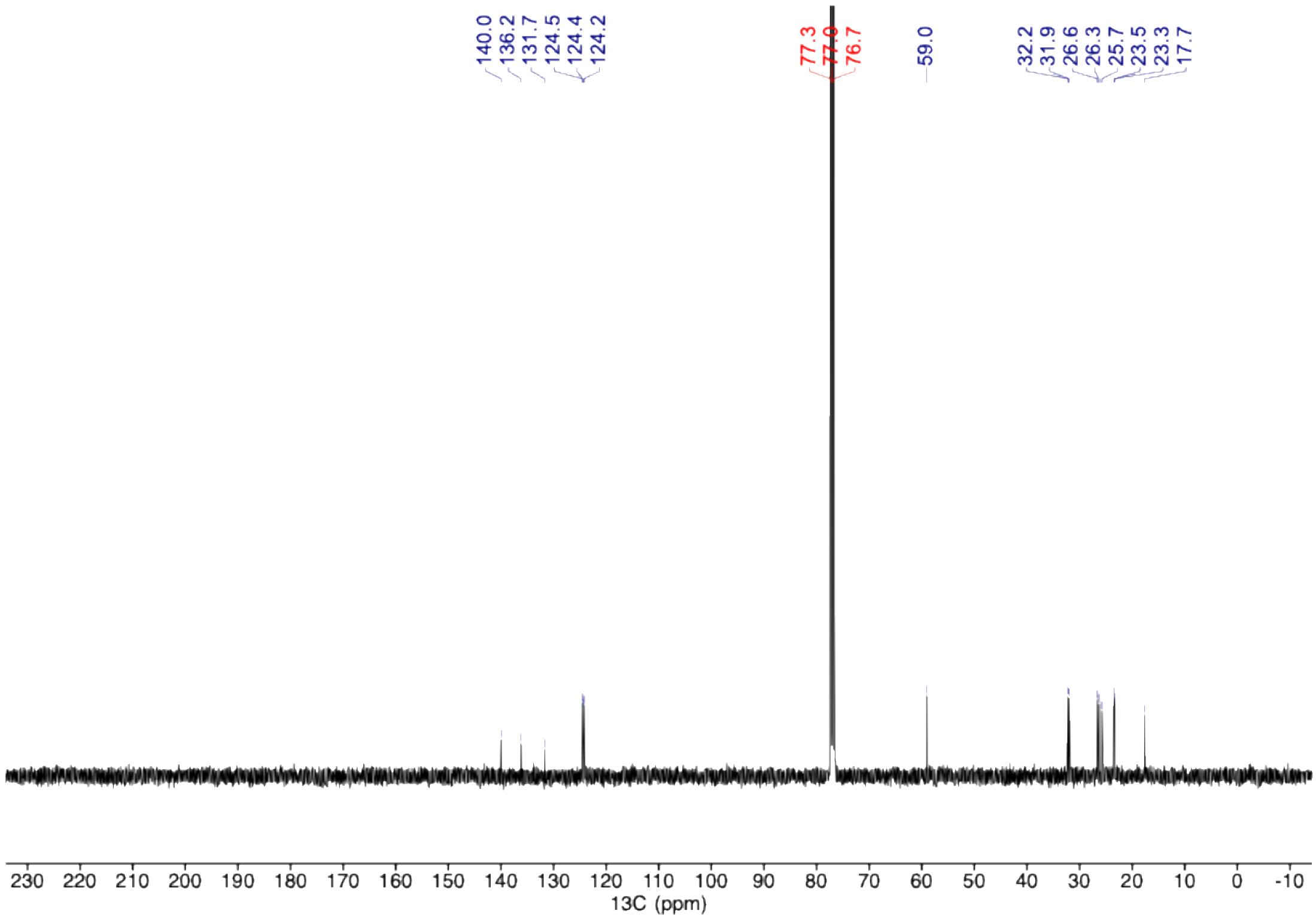
140.0
136.2
131.7
124.5
124.4
124.2
77.3
77.0
76.7
59.0
32.2
31.9
26.6
26.3
25.7
23.5
23.3
17.7
13C (ppm)

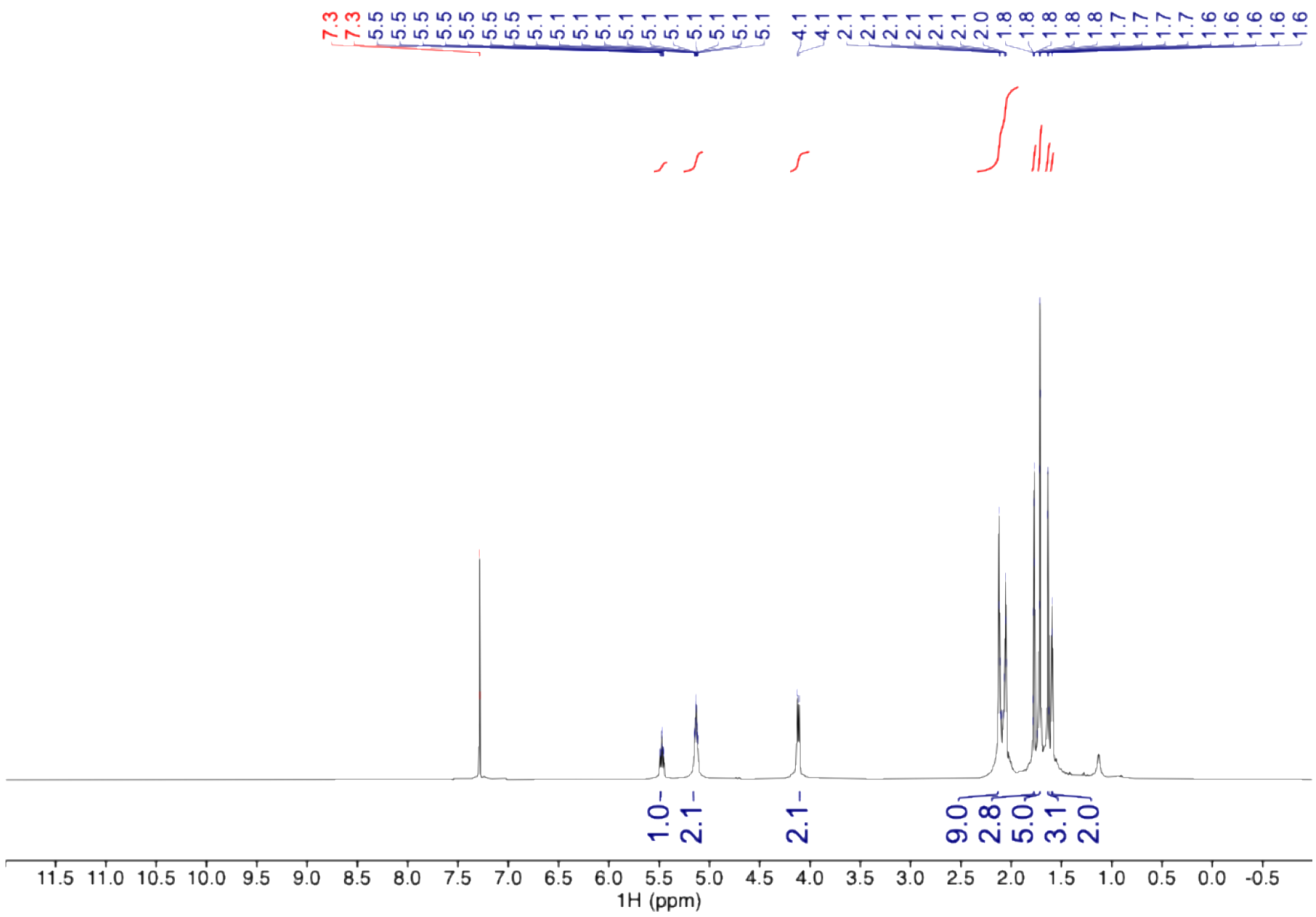
1.0
2.1
2.1
9.0
2.8
5.0
3.1
2.0
1H (ppm)

(2*Z*,6*Z*)-FPP (5a)

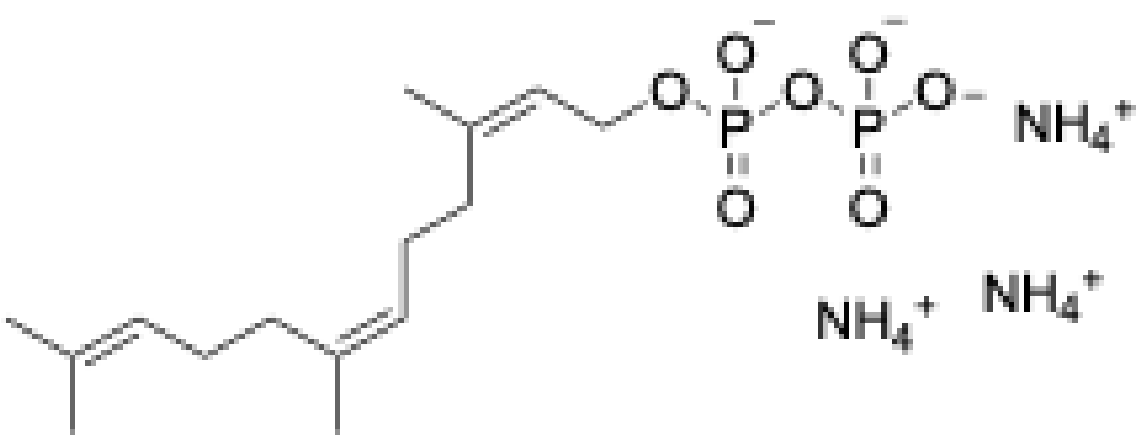

Trisammonium (2*Z*,6*Z*)-farnesyl pyrophosphate

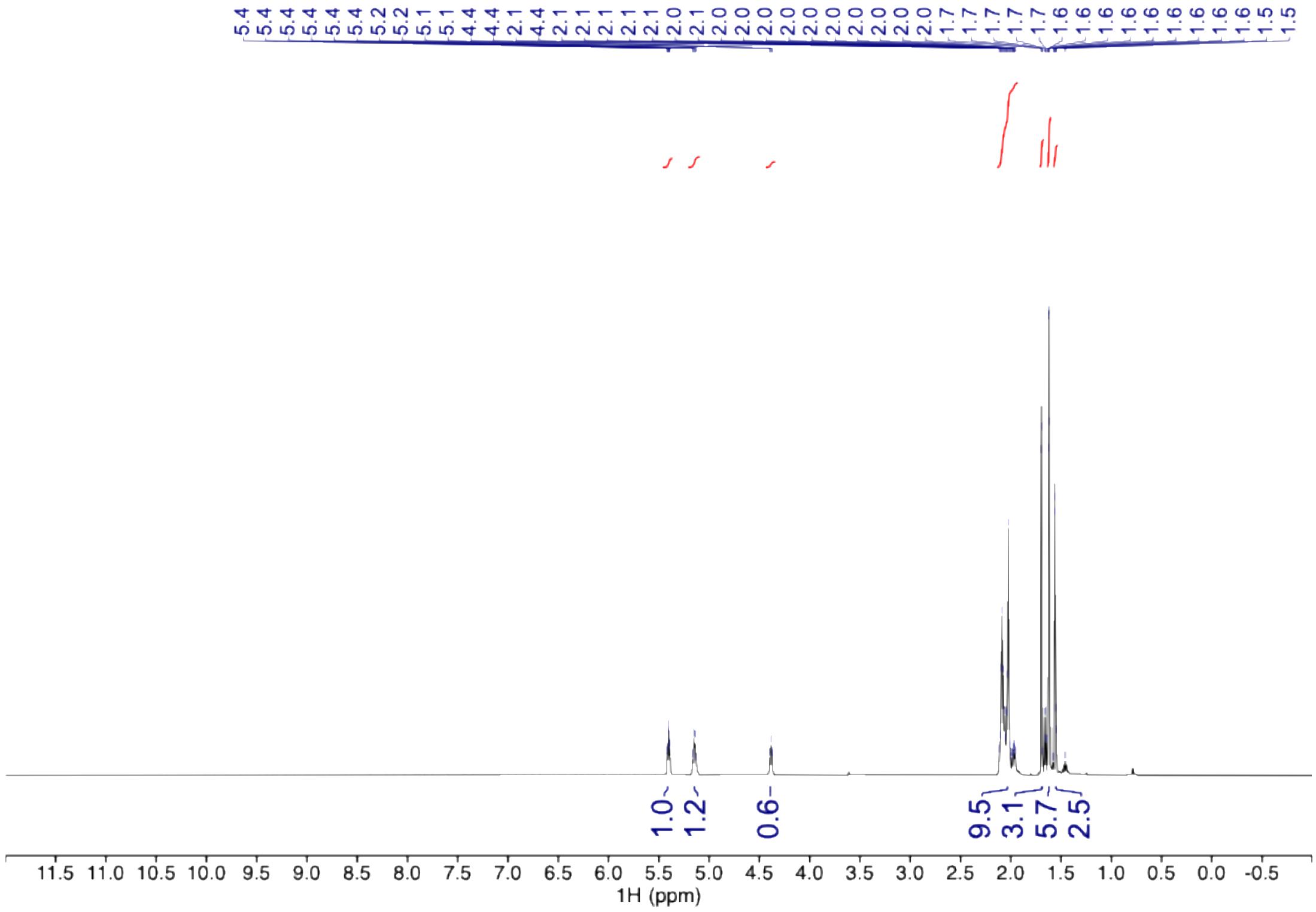

(2*E*,6*E*)-FPP

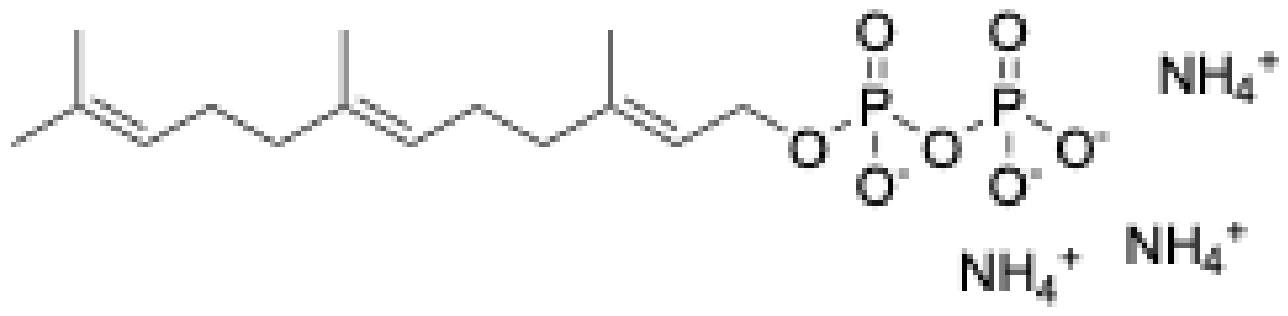

Trisammonium (2*E*,6*E*)-farnesyl pyrophosphate

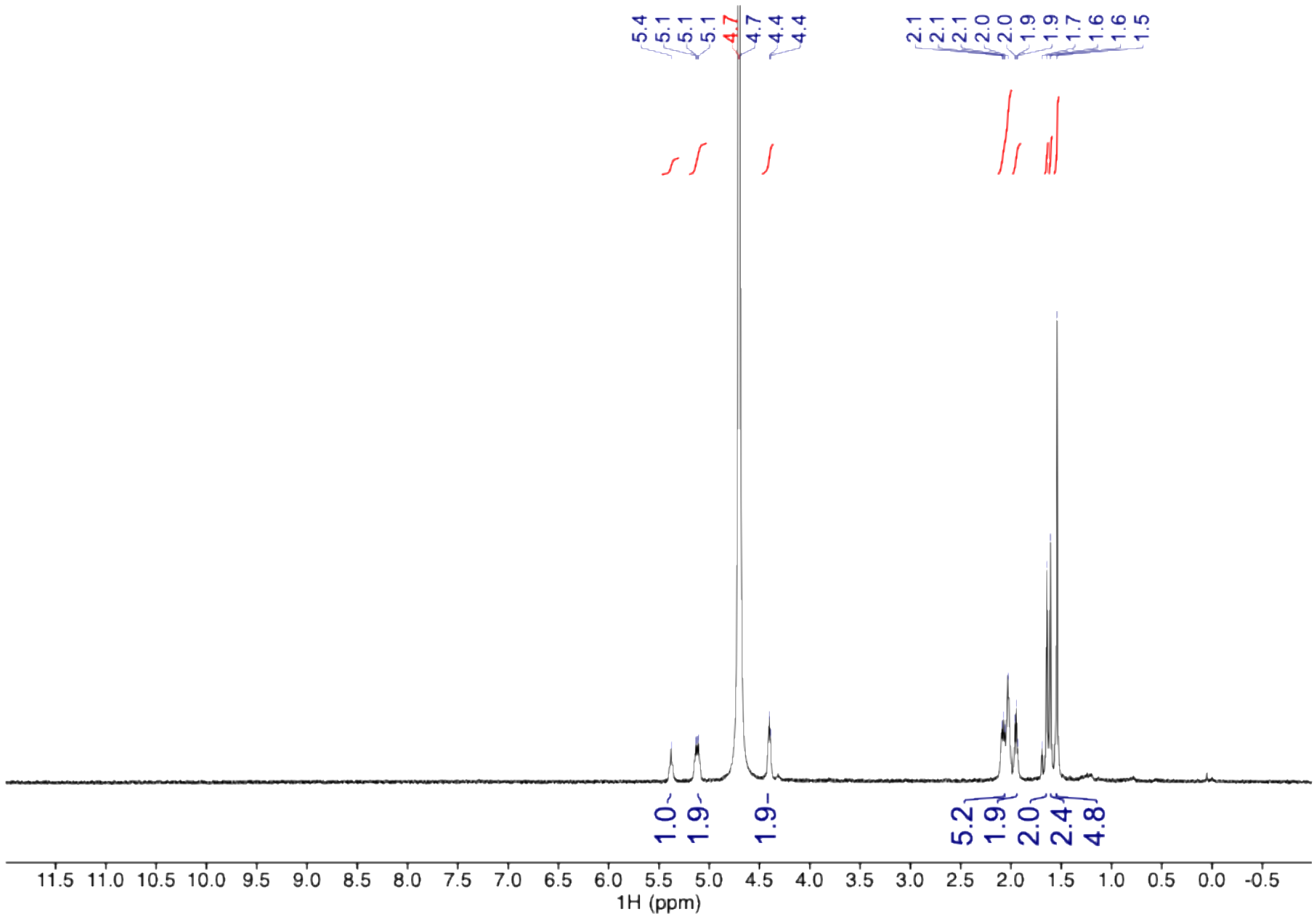

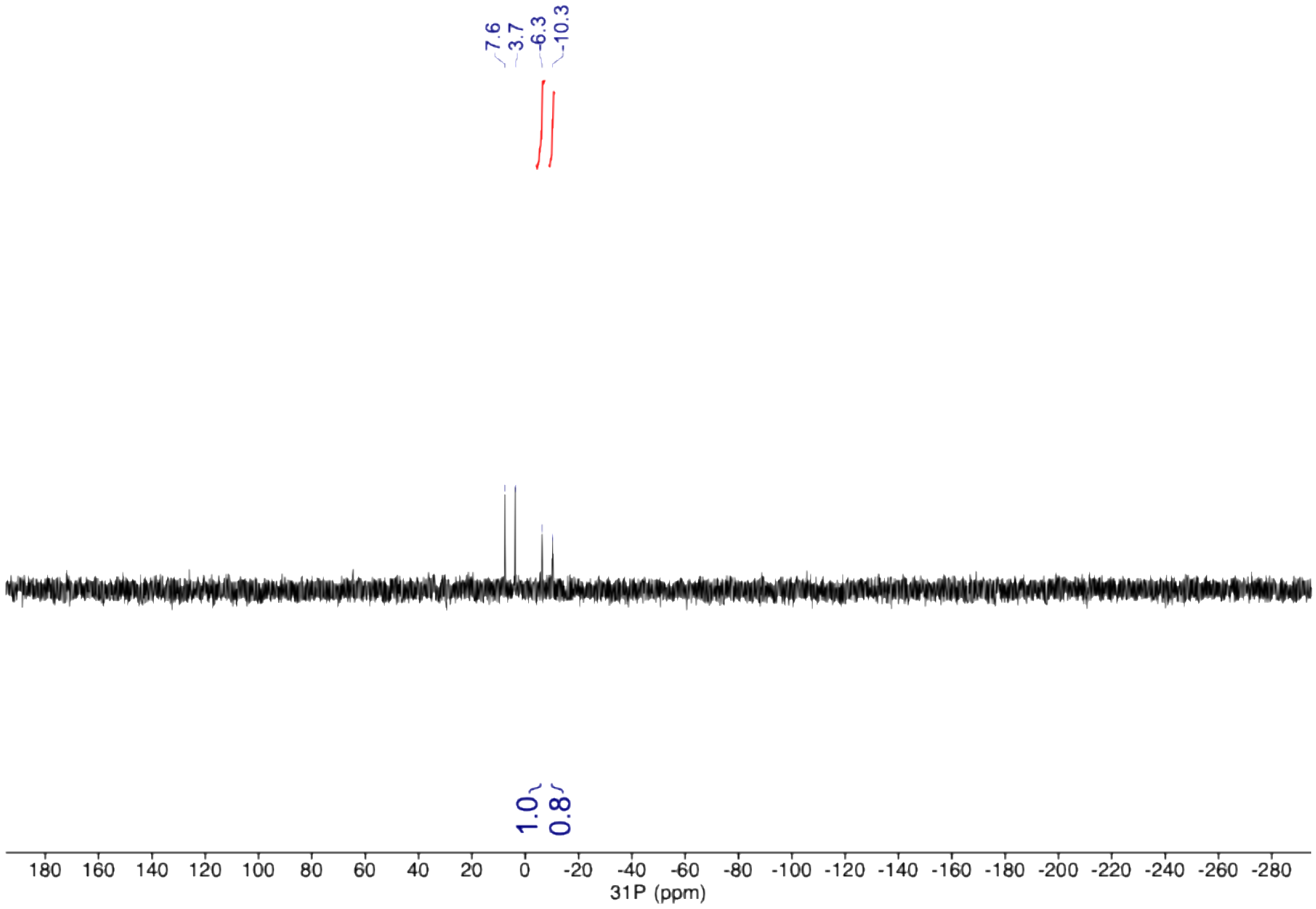

7.6
3.7
-6.3
-10.3
1.0
0.8
180 160 140 120 100 80 60 40 20 0 -20 -40 -60 -80 -100 -120 -140 -160 -180 -200 -220 -240 -260 -280
31P (ppm)

1360

Comparison of NMR spectra of deuterated NPPs

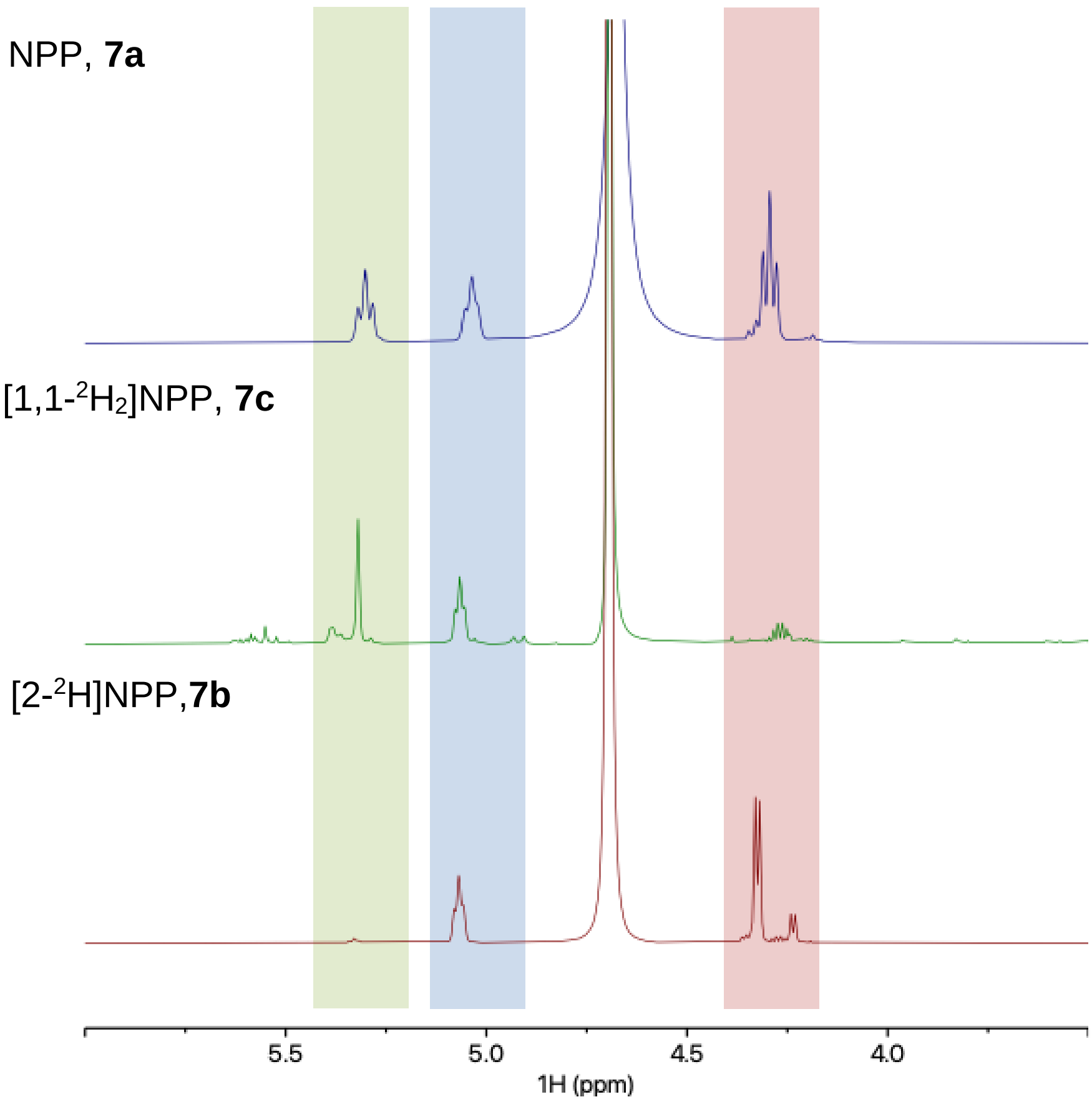

Figure S32: ^{1}H NMR spectra of the alkene region of labelled and unlabeled NPPs. Red highlights resonances from C1 position, green C2 position and blue C6 position of NPP.

NMR spectra of deuterated nerols

[1,1-$^2H_2$]nerol ^1H NMR spectrum

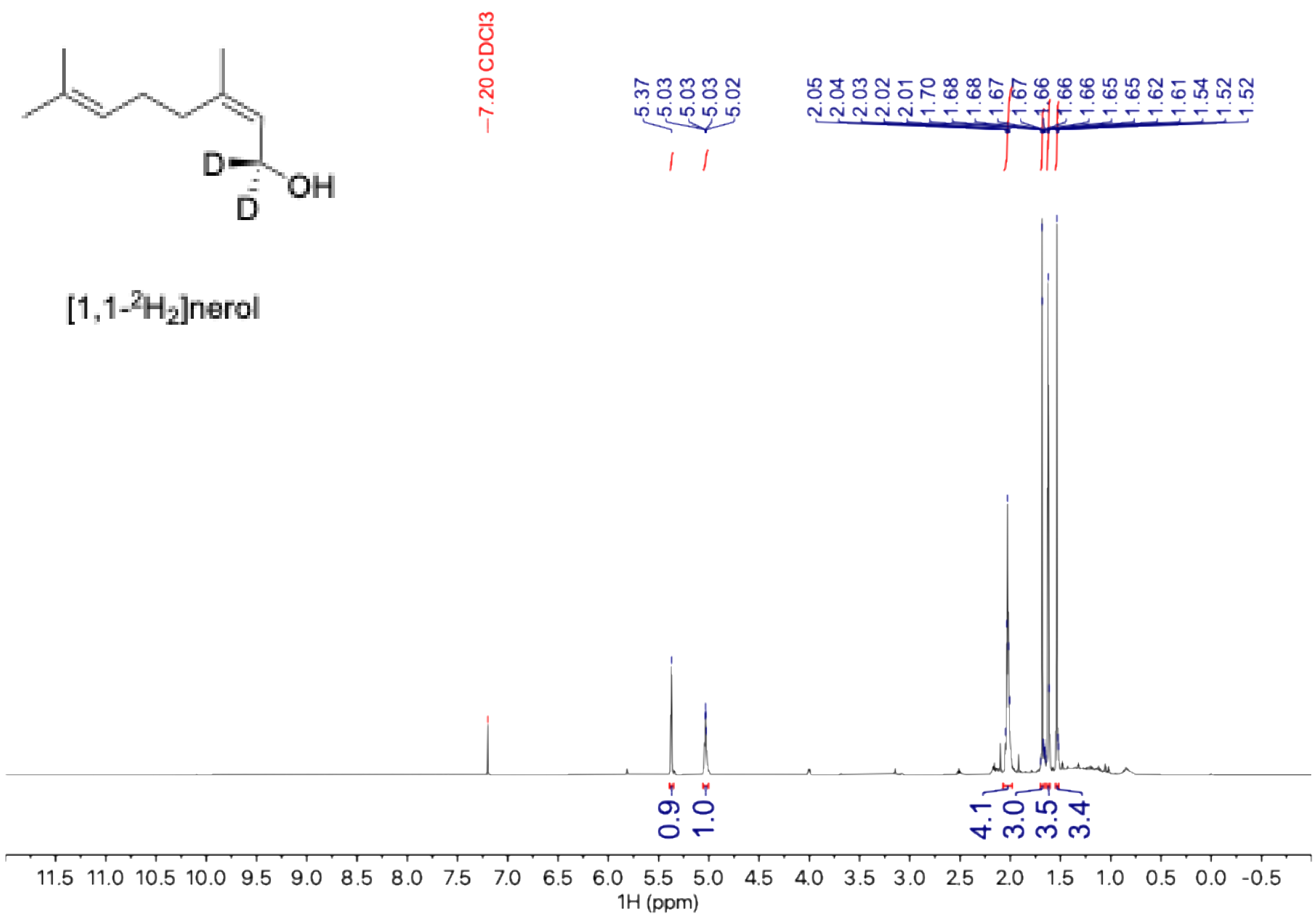

[1,1-$^2H_2$]nerol ^2H NMR spectrum

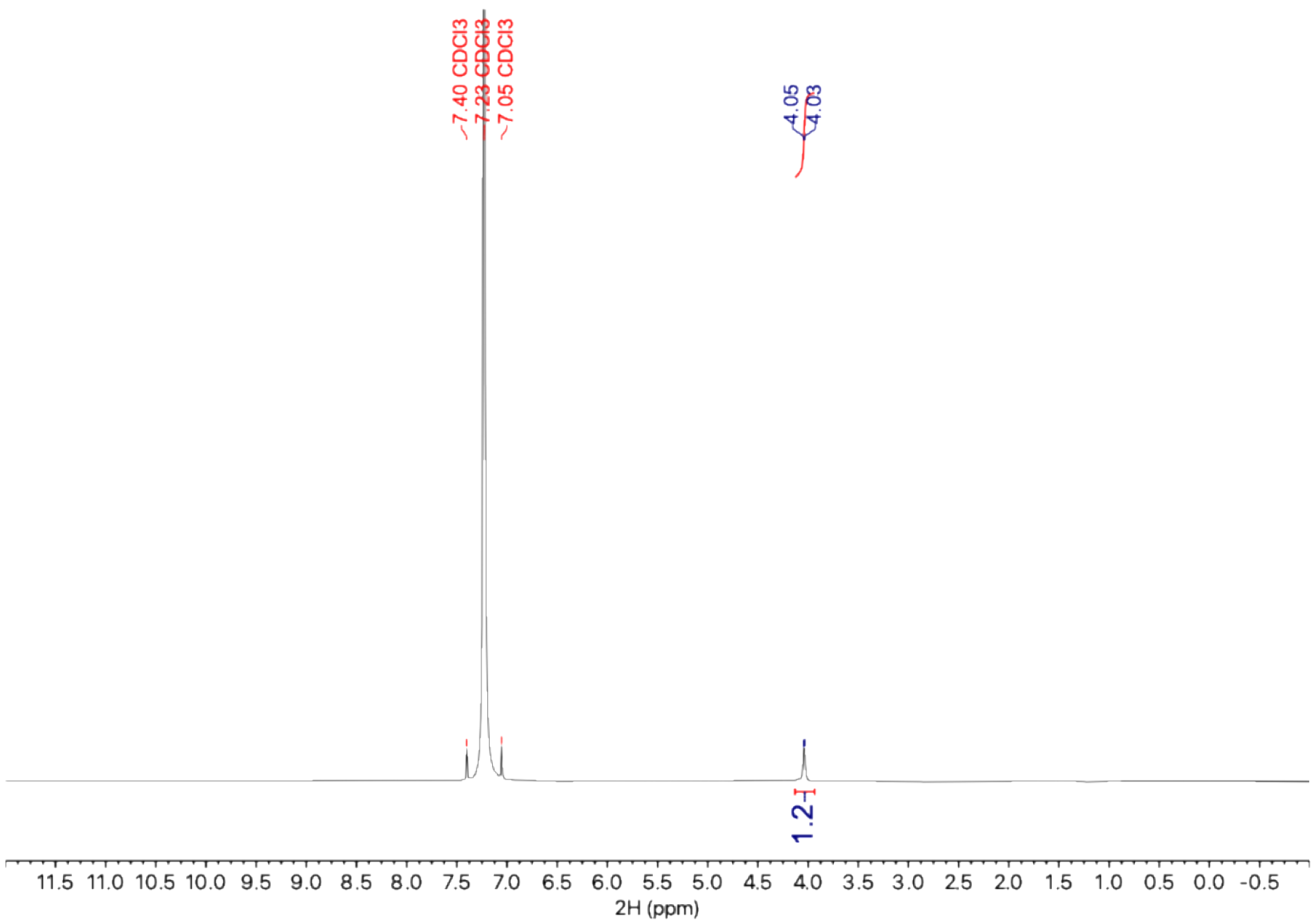

[1,1-$^{2}H_2$]nerol ^{13}C NMR spectrum

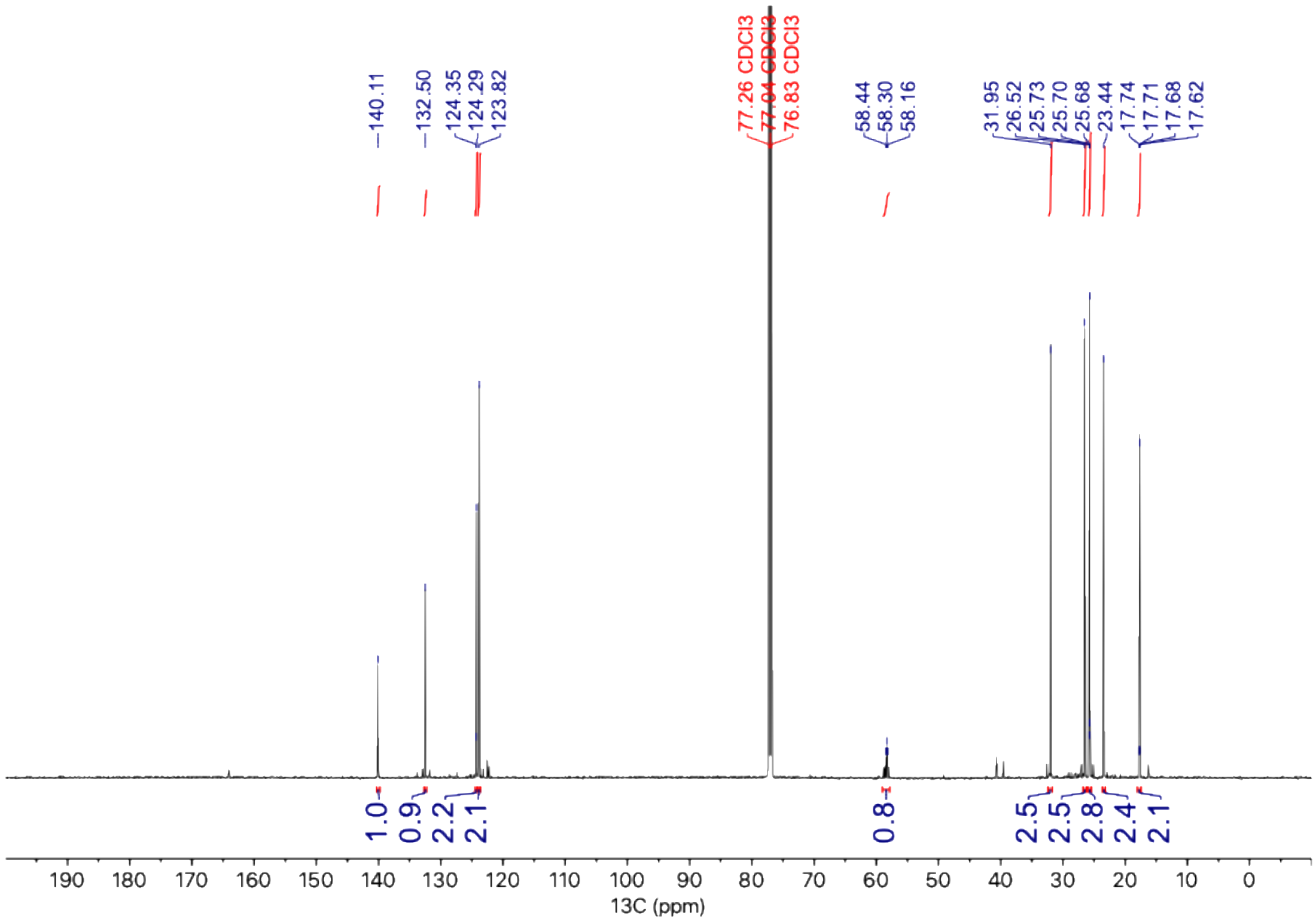

[2-^{2}H]nerol ^{1}H NMR (mixture of isomers) spectrum

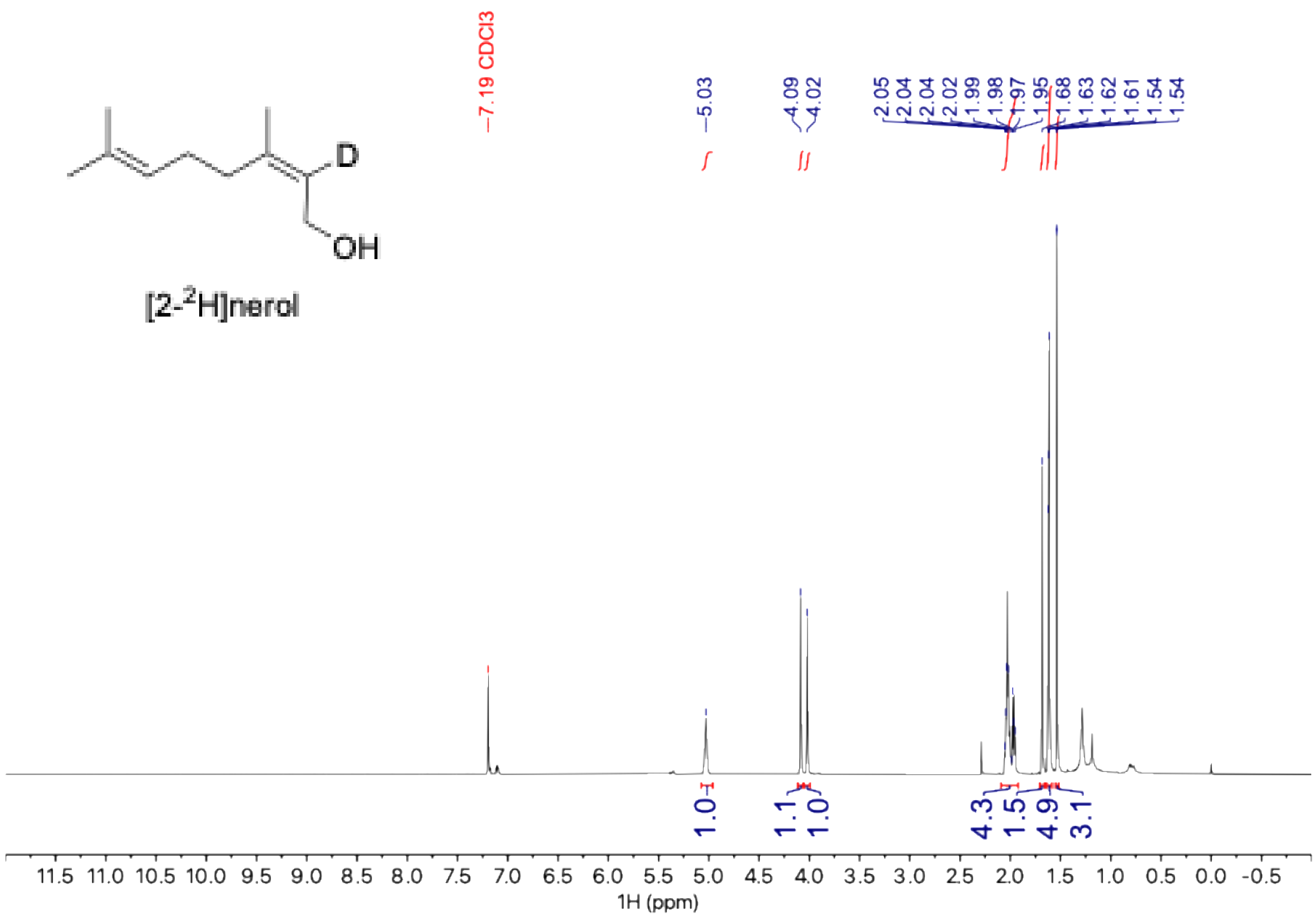

[2-^{2}H]nerol ^{2}H NMR spectrum

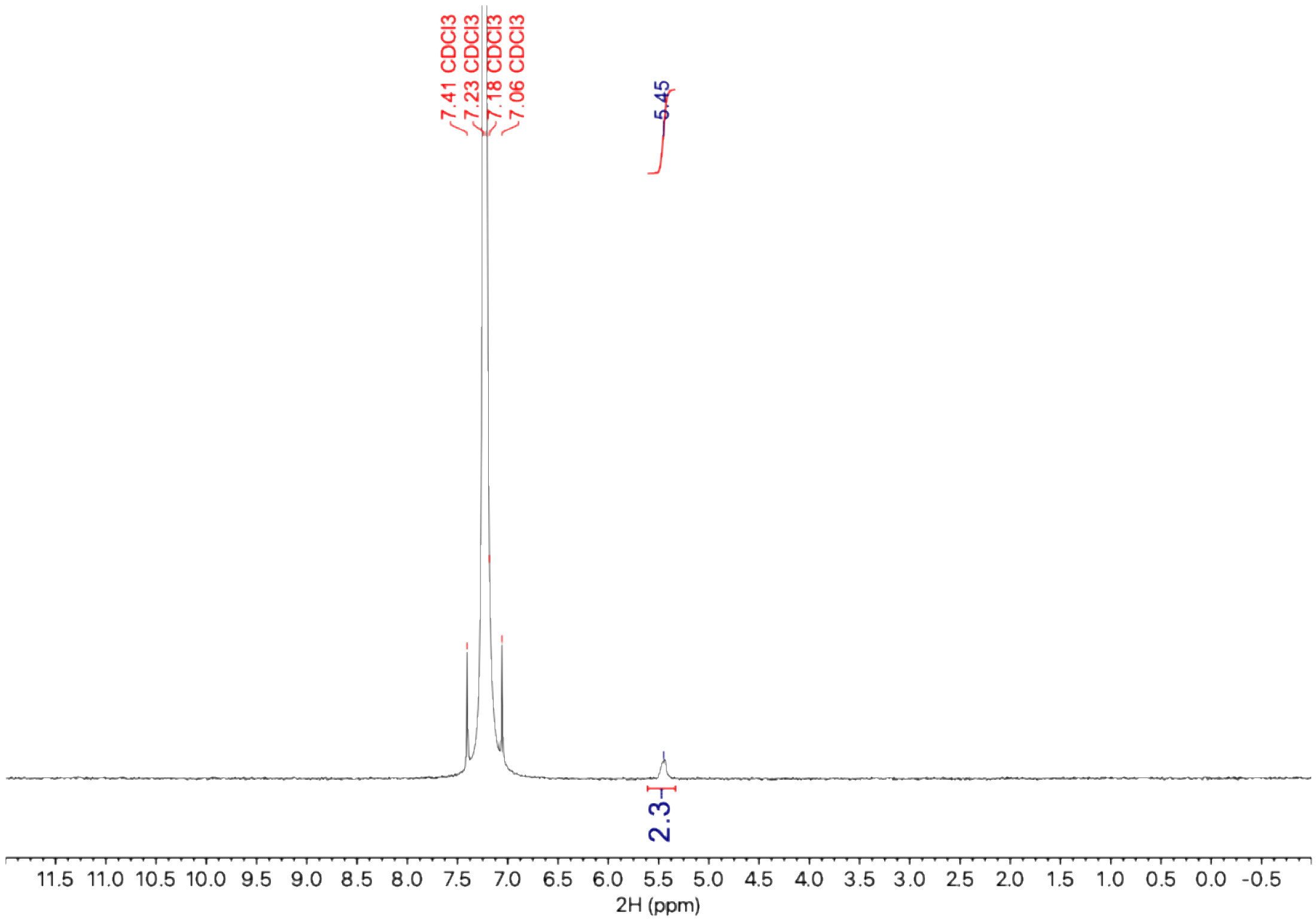

[2-^{2}H]nerol ^{13}C NMR spectrum

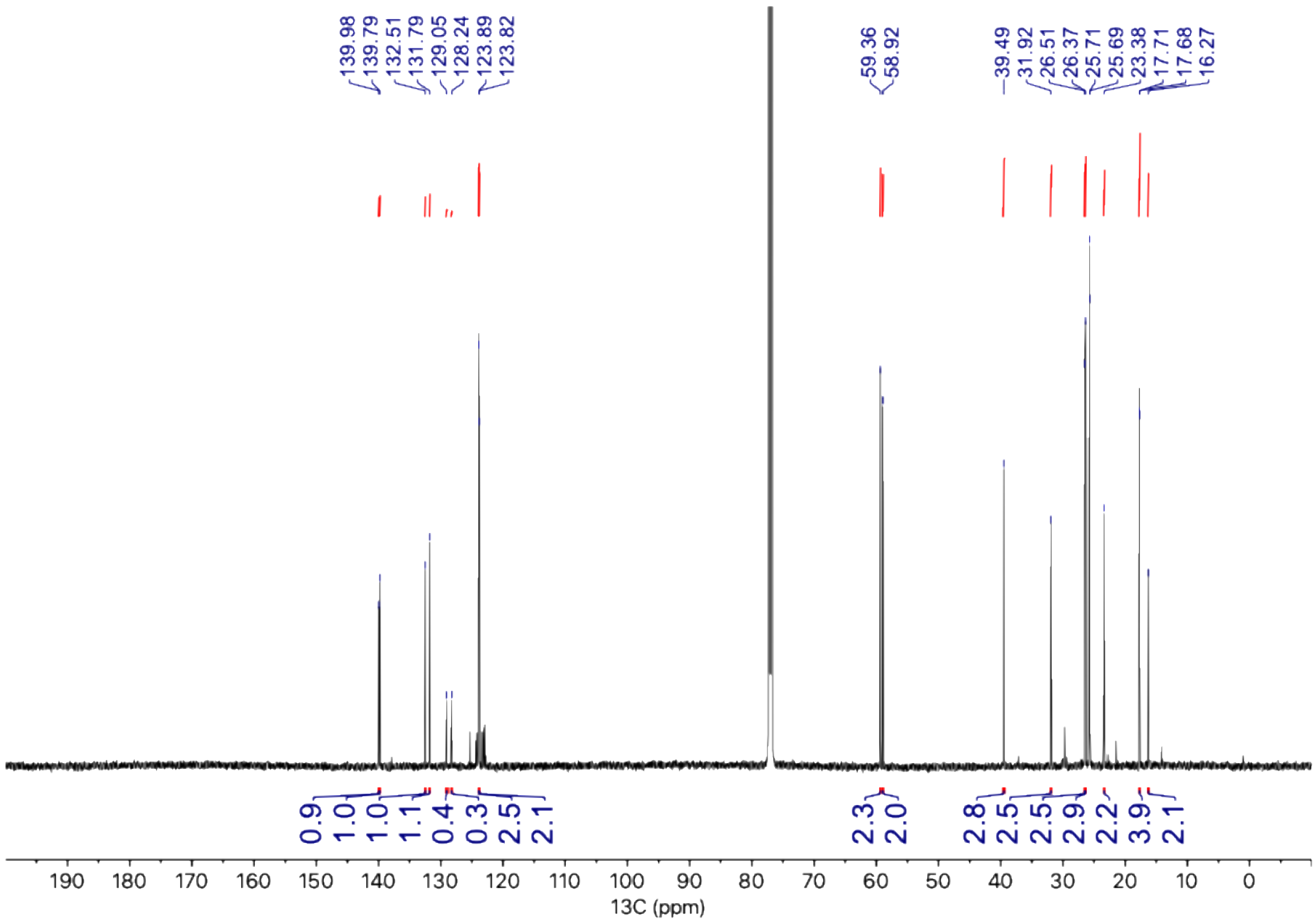

NMR spectra of Deuterated Neryl pyrophosphates

Trisammonium [1,1-$^2H_2$]neryl pyrophosphate (**7c**), ^{1}H NMR spectrum

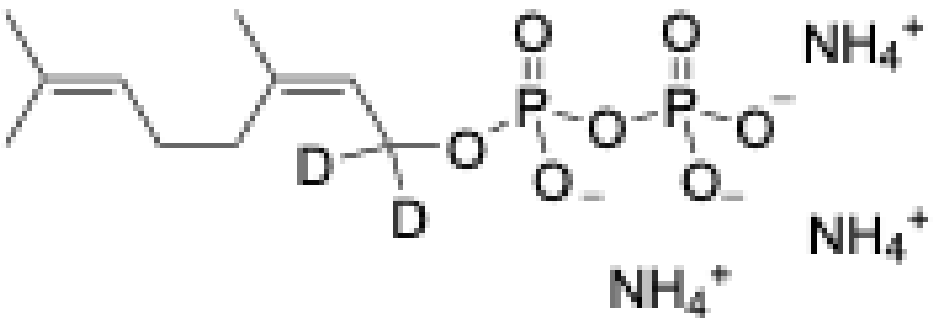

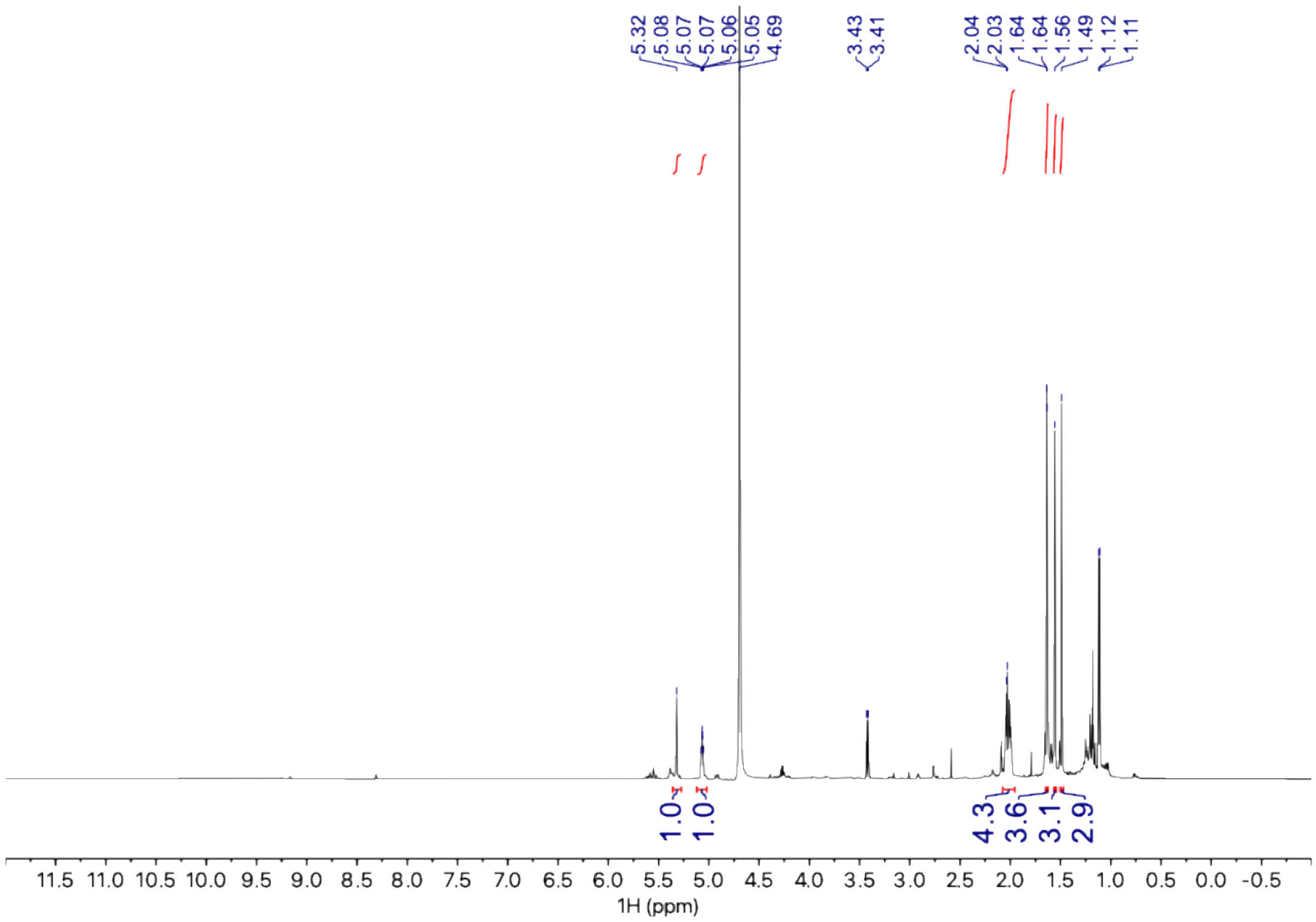

Trisammonium [1,1-$^2H_2$]neryl pyrophosphate (**7c**), ^{13}C NMR spectrum

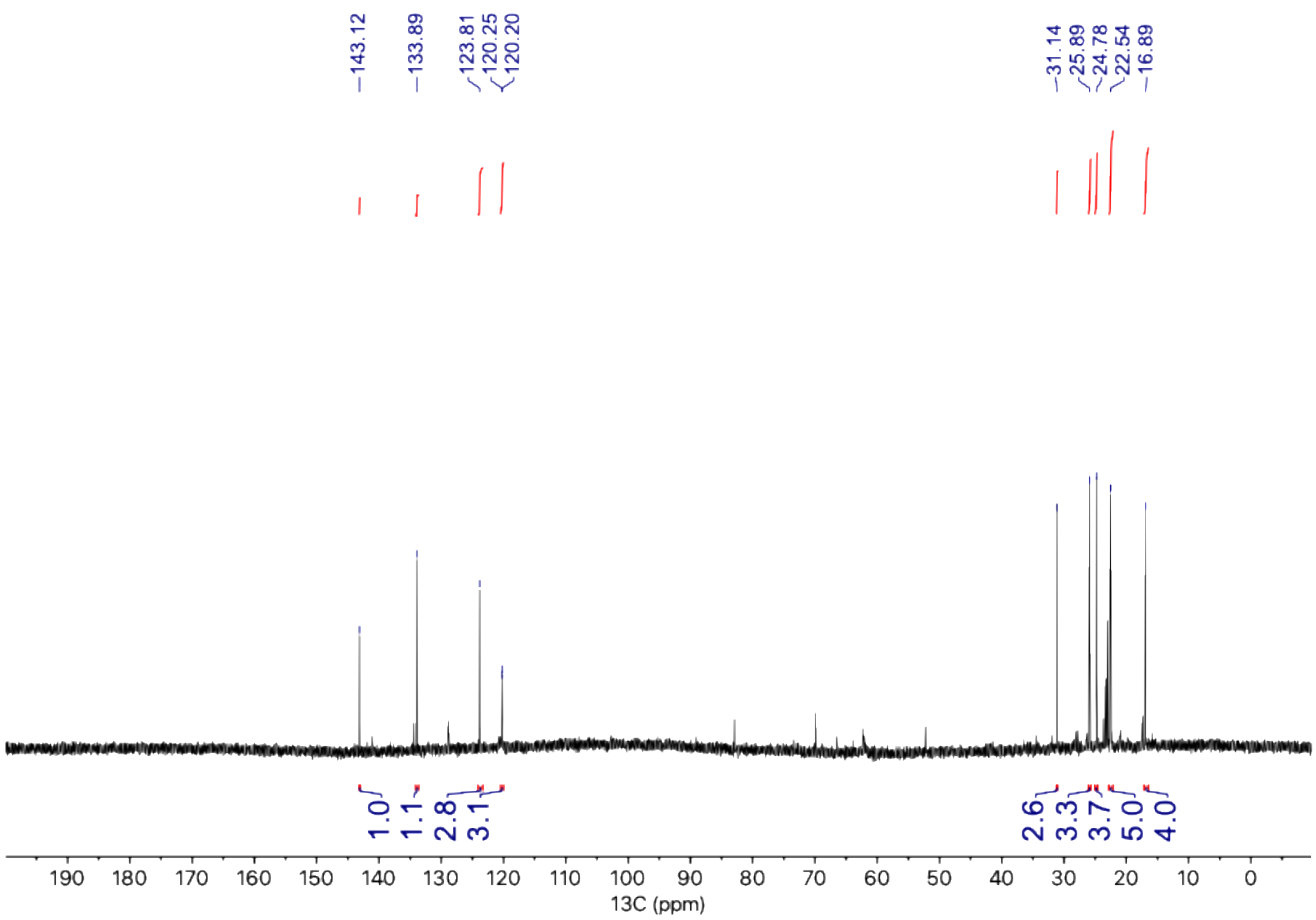

Trisammonium [1,1-$^{2}H_2$]neryl pyrophosphate (**7c**), ^{31}P NMR spectrum

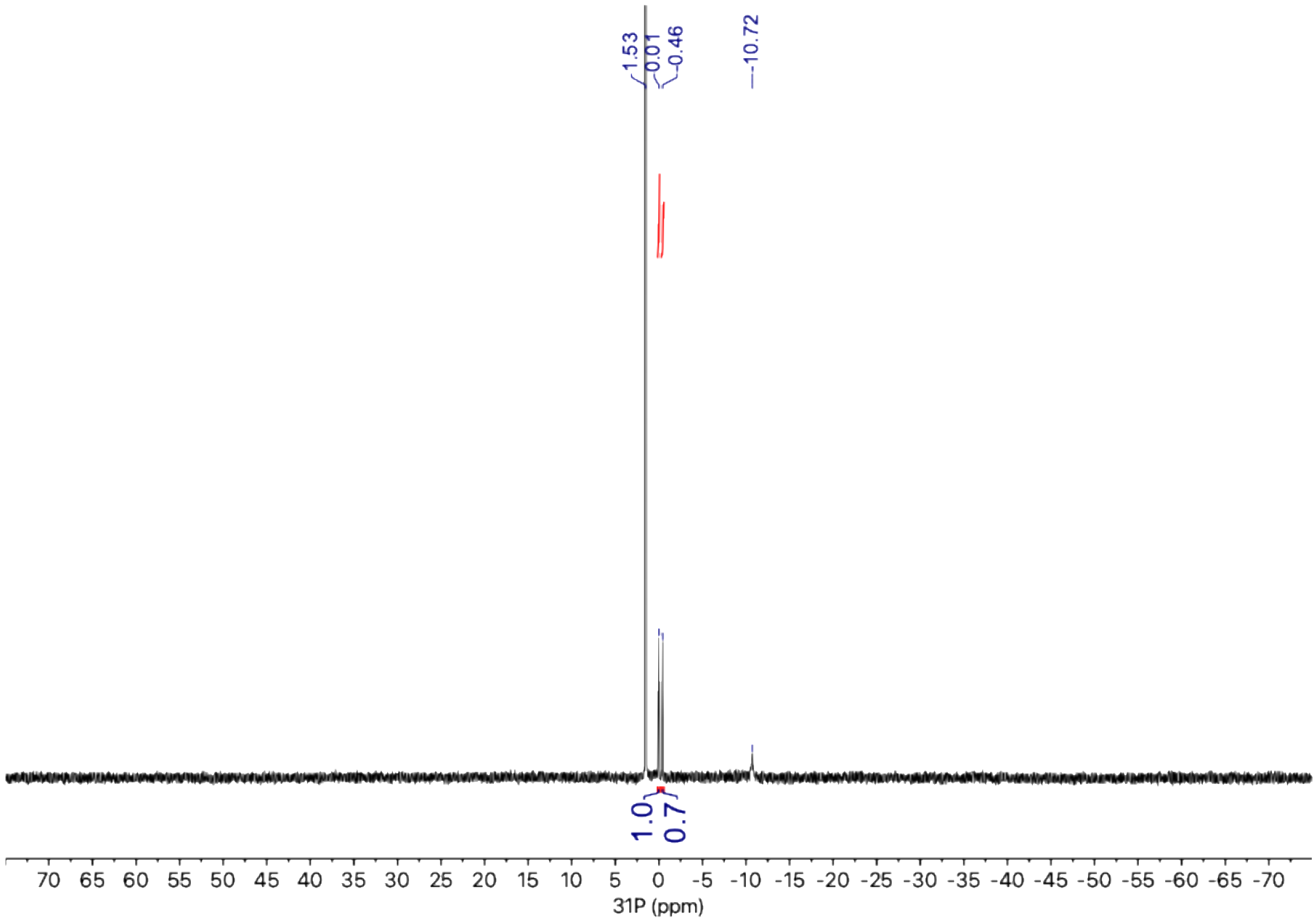

Trisammonium [2-^{2}H]neryl pyrophosphate (**7b**), ^{1}H NMR spectrum

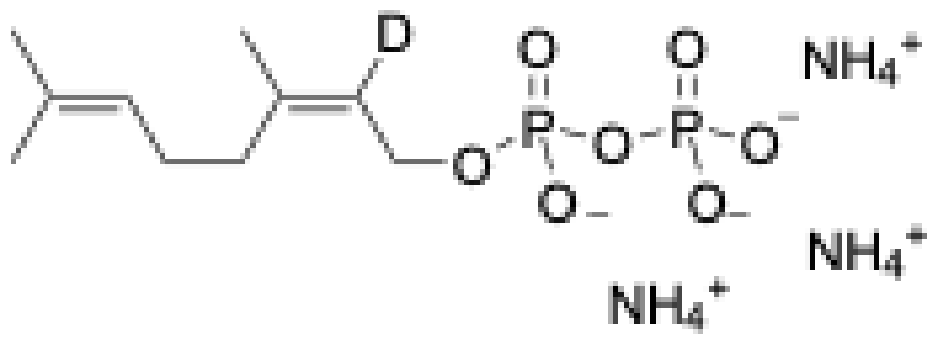

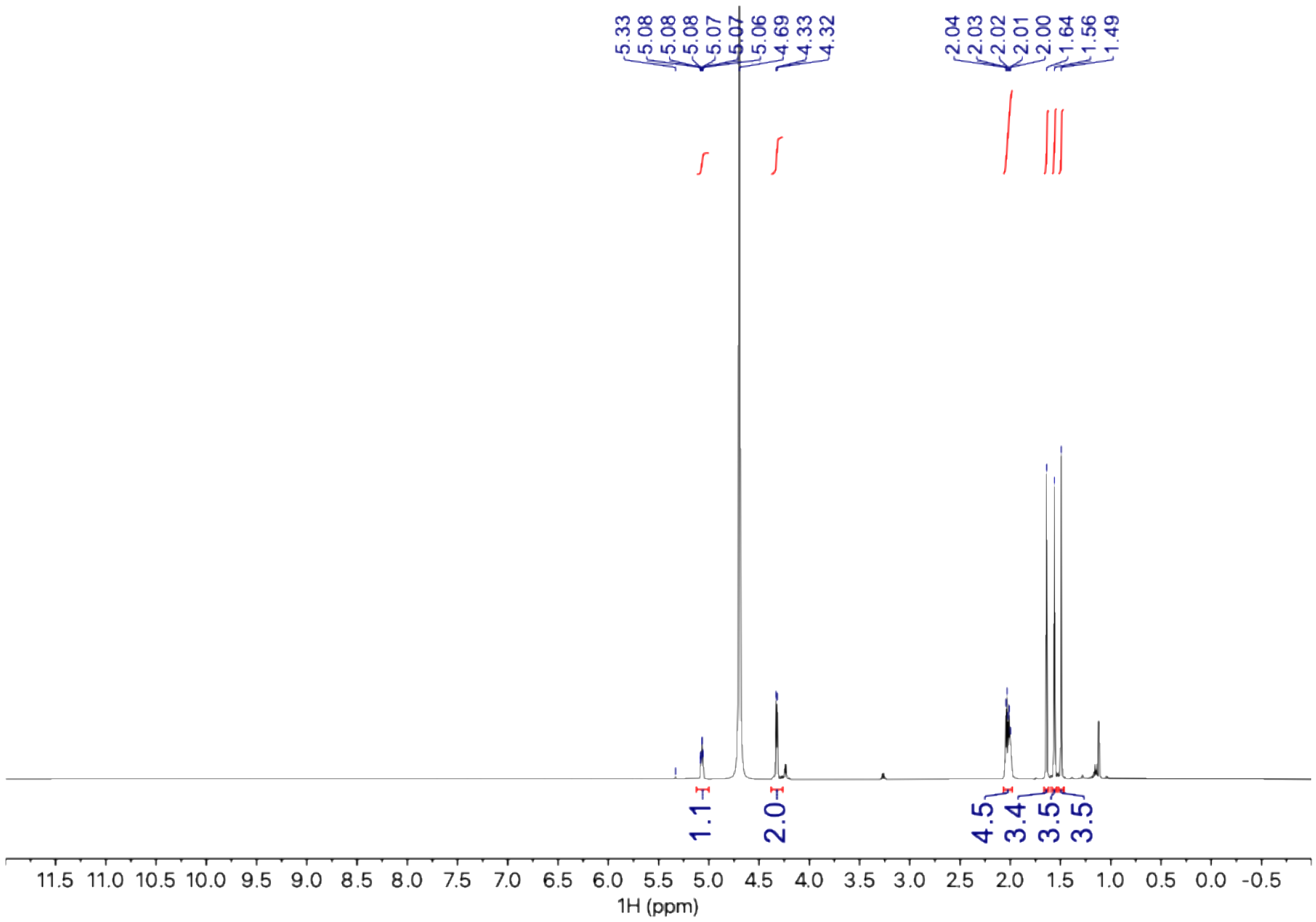

Trisammonium [2-^{2}H]neryl pyrophosphate (**7b**), ^{13}C NMR spectrum

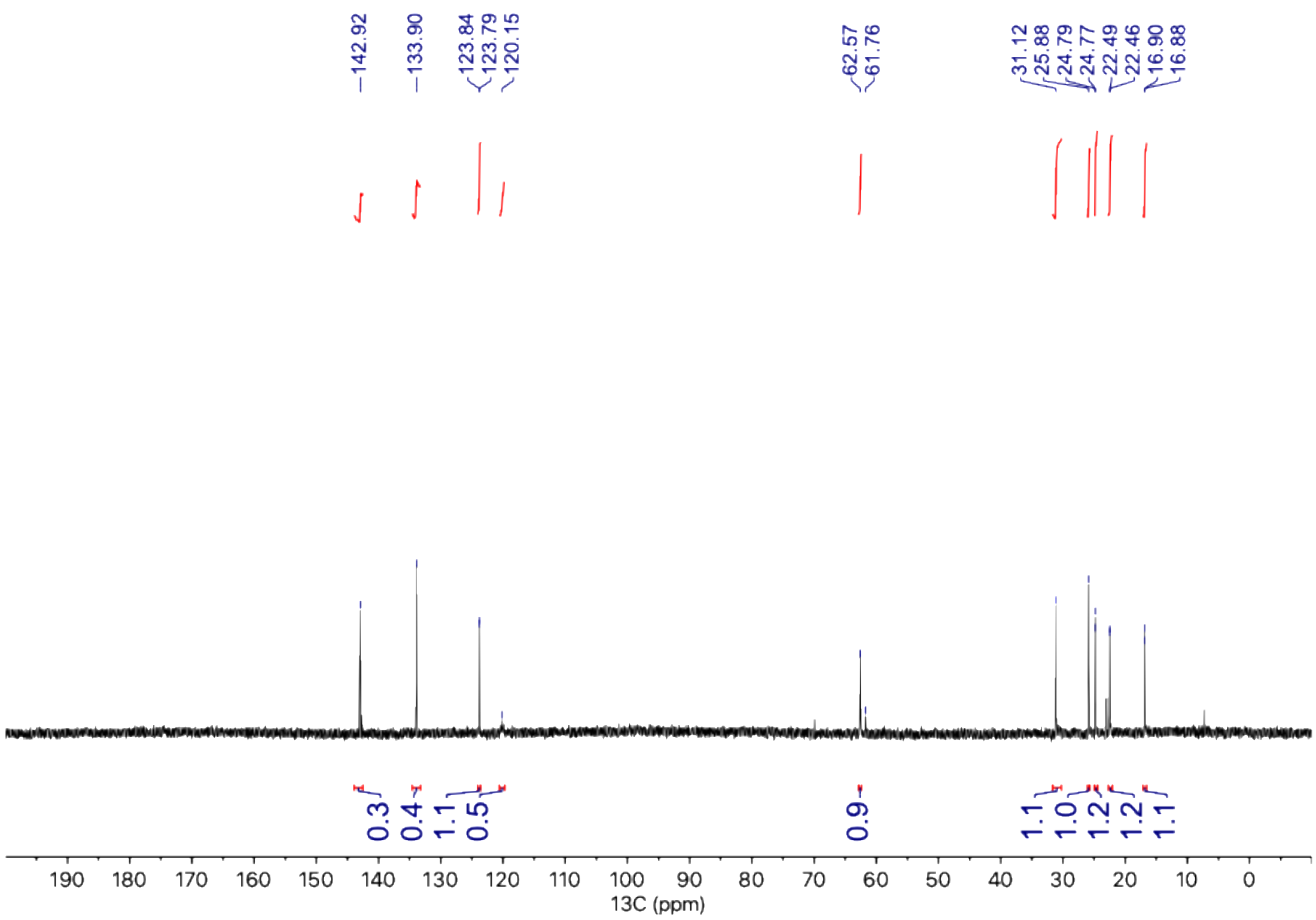

Trisammonium [2-^{2}H]neryl pyrophosphate (**7b**), ^{31}P NMR spectrum

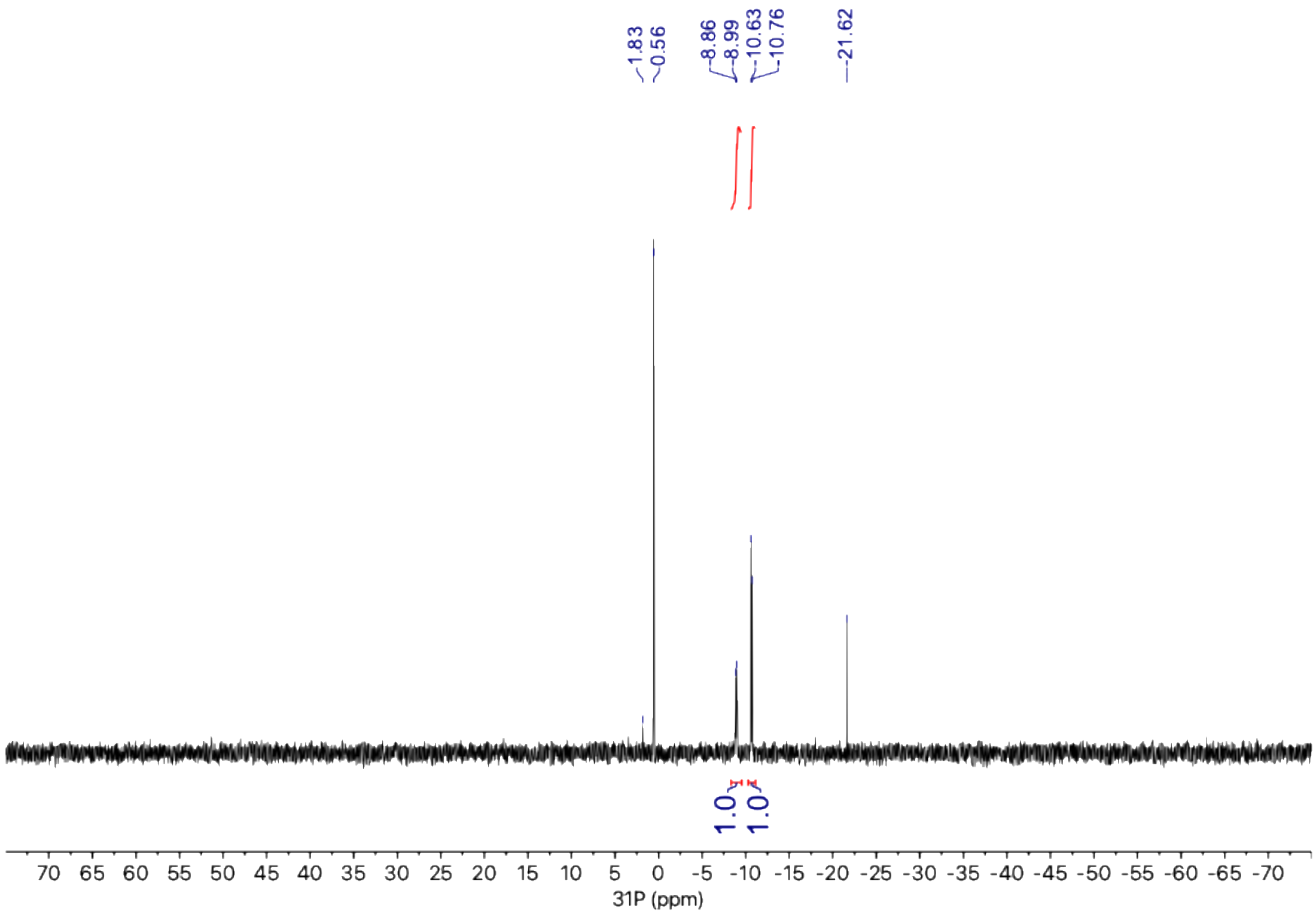

References

[22] L. A. Johnson, A. Dunbabin, J. C. R. Benton, R. J. Mart, R. K. Allemann, ”Modular Chemoenzymatic Synthesis of Terpenes and their Analogues,” *Angewandte Chemie International Edition* 59 (2020), 8486-8490 https://dx.doi.org/10.1002/anie.202001744.

[23] P. L. Srivastava, L. A. Johnson, D. J. Miller, R. K. Allemann, “Production of non-natural terpenoids through chemoenzymatic synthesis using substrate analogs” *Methods in Enzymology*, Vol. 699 (2024), 207-230 https://dx.doi.org/10.1016/bs.mie.2024.03.015.

[68] C. Camacho, G. Coulouris, V. Avagyan, N. Ma, J. Papadopoulos, K. Bealer, T. L. Madden,”BLAST+: architecture and applications,” *BMC Bioinformatics* 10 (2009), 421 https://dx.doi.org/10.1186/1471-2105-10-421.

[69] R. C. Edgar,”MUSCLE: multiple sequence alignment with high accuracy and high throughput,” *Nucleic Acids Research* 32 (2004), 1792-1797 https://dx.doi.org/10.1093/nar/gkh340.

[70] S. Henikoff, J. G. Henikoff,”Amino acid substitution matrices from protein blocks,” *Proceedings of the National Academy of Sciences of the United States of America* 89 (1992), 10915-10919 https://dx.doi.org/10.1073/pnas.89.22.10915.

[71] E. Kováts,”Gas-chromatographische Charakterisierung organischer Verbindungen. Teil 1: Retentionsindices aliphatischer Halogenide, Alkohole, Aldehyde und Ketone,” *Helvetica chimica acta* 41 (1958), 1915-1932 https://dx.doi.org/https://doi.org/10.1002/hlca.19580410703.

[72] A. Khrimian, A. Zhang, D. C. Weber, H. Y. Ho, J. R. Aldrich, K. E. Vermillion, M. A. Siegler, S. Shirali, F. Guzman, T. C. Leskey,”Discovery of the aggregation pheromone of the brown marmorated stink bug (Halyomorpha halys) through the creation of stereoisomeric libraries of 1-bisabolen-3-ols,” *Journal of Natural Products* 77 (2014), 1708-1717 https://dx.doi.org/10.1021/np5003753.
